# Aryl (β,β′,β″-Trifluoro)-*tert*-butyl: A Candidate Motif for the Discovery of Bioactives

**DOI:** 10.1021/acs.orglett.3c02236

**Published:** 2023-09-08

**Authors:** Luca S. Dobson, Qingzhi Zhang, Benjamin A. McKay, Oluwayinka Oke, Chukwuemeka Isanbor, Mohd Faheem Khan, Bruno A. Piscelli, David B. Cordes, Rodrigo A. Cormanich, Cormac D. Murphy, David O’Hagan

**Affiliations:** † School of Chemistry, University of St Andrews, North Haugh, St Andrews KY16 9ST, U.K.; ‡ Chemistry Department, University of Lagos, Akoka, Lagos 101245, Nigeria; § School of Biomolecular and Biomedical Science, University College Dublin, Belfield, Dublin 4, Ireland; ∥ Chemistry Institute, University of Campinas, Monteiro Lobato Street, Campinas, Sao Paulo 13083-862, Brazil

## Abstract

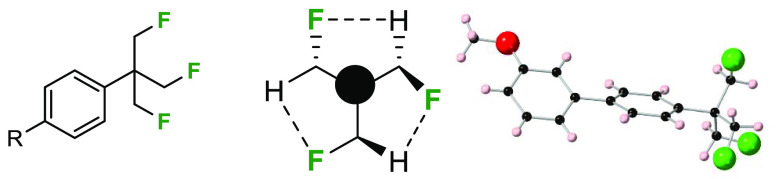

The (β,β′,β″-trifluoro)-*tert*-butyl (TFTB) group has received very little attention in the literature. This work presents a direct synthesis of this group and explores its properties. The TFTB group arises when the methyl groups of a *tert*-butyl moiety are exchanged for fluoromethyl groups. Sequential fluoromethylations result in a decrease of Log *P* (increasing hydrophilicity), ultimately by 1.7 Log *P* units in the TFTB group relative to that of *tert*-butyl benzene itself. A focus is placed on synthetic transformations, conformational analysis, and metabolism of the TFTB group in the context of presenting a favorable profile as a motif for the discovery of bioactives.

The selective fluorination of aromatic rings has a tendency to increase their lipophilicity. This phenomenon has been widely articulated for aryl–F or aryl–CF_3_ fluorinations, and it is an important concept in medicinal chemistry.^1^ However, the opposite effect is found when aliphatics are selectively fluorinated, and this concept is perhaps less widely embedded in the culture of the discovery of bioactives.^1,2^ This concept has been explored across a range of structural motifs, and we have explored the phenomenon in the context of partially fluorinated cyclohexanes.^3^ In this Letter, a focus is placed on selective fluorination of the aryl *tert*-butyl group. The *tert*-butyl group is a ubiquitous motif in organic chemistry; however, its high lipophilicity mitigates against its wide utility in medicinal chemistry.^4^ For example, only a handful of the top 200 selling drugs of 2021 contain a *tert*-butyl group. Examples are ventolin (salbutamol),^5^ ivacaftor formulations,^6^ bupropion formulations (e.g., Wellbutrin),^7^ and timolol formulations (e.g., Combigan).^8^ The lipophilicity of the *tert*-butyl group exposes any drug candidate to the pharmacokinetic challenges associated with increasing Log *P* (low solubility, membrane and albumin association, increased metabolism, etc.).^9^ The *tert*-butyl group is represented more widely in agrochemical products where higher lipophilicities are tolerated to a greater extent (e.g., chromafenozide (Matric), isouron, and tebufloquin).^10^ Recognizing that fluorinations of aliphatic motifs can decrease Log *P*, it became an objective to explore selective fluorinations of the *tert*-butyl group, specifically replacing the methyl substituents with fluoromethyl groups, anticipating Log *P* reductions. Only two papers have been reported^11,12^ in the literature regarding this (β,β′,β″-trifluoro)-*tert*-butyl substituent (TFTB), and these are confined exclusively to amine **5** as a building block; the most recent report^11^ was over two decades ago. These papers outline different synthetic approaches to amine **5**, as summarized in Scheme 1.

**Scheme 1:**
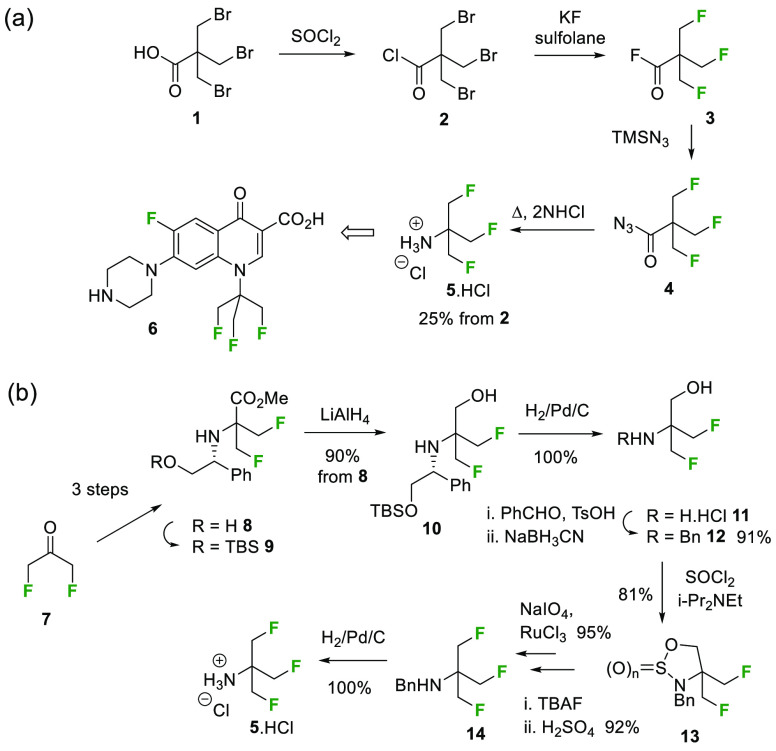
Previous Routes to the TFTB Motif in Amine **5**(12,11)

In the first route,^12^ β,β′,β″-tribromopivalic acid **1** was converted to the corresponding trifluoroacyl fluoride **3** to enable a Curtius rearrangement to amine **5**. The free base was then progressed to fluoroquinolone **6**, as one of a range of variants exploring antibiotic structure–activity relationships. A more convoluted route to amine **5** was reported a decade later.^11^ Although not an obvious improvement, it avoided “the distillation of labile fluorinated pivaloyl fluorides and isocyanates”. Amine **5** is the only TFTB building block reported so far, and no particular properties of the TFTB motif were described. We report a synthesis of the aryl-TFTB motif for the first time. A conformational analysis is explored, and Log *P* comparisons are made, measured progressively from the *tert*-butyl group through mono-, di-, and tri- fluoromethyl substituents. Given the current concern regarding persistent organofluorine compounds,^13^ metabolism of the aryl-TFTB substituent is also explored.

A direct synthetic approach to the TFTB motif was envisaged through the formal deoxyfluorination of an appropriate precursor triol such as **16**, and the route that developed is summarized in Scheme 2. Intermediate triol **16** was prepared via a Cannizzaro-type reaction on phenylacetaldehyde **15** as previously reported,^14^ and then the triol was activated to tritosylate **17**. These reactions proved straightforward, and **17** could be readily purified by recrystallization.

**Scheme 2:**
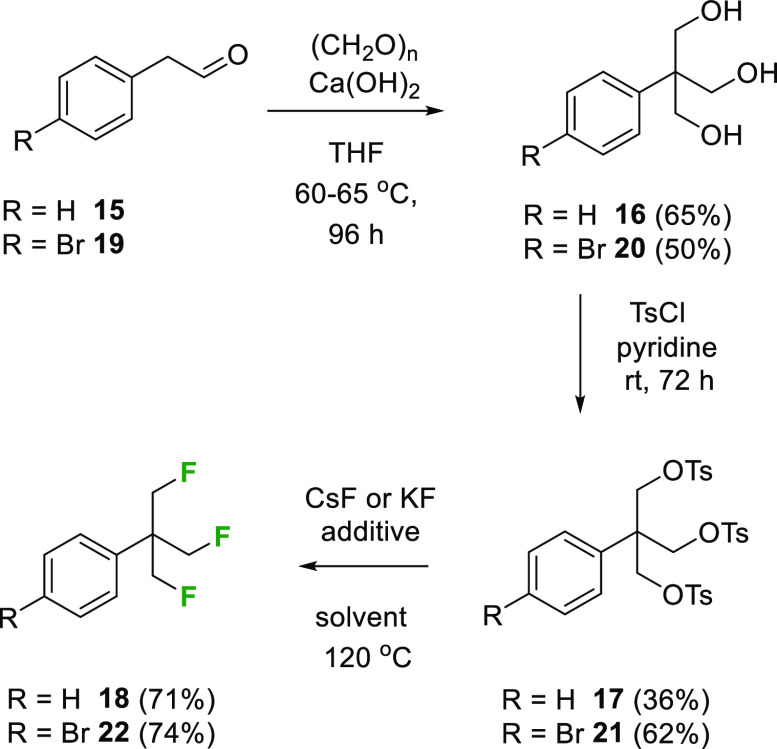
Synthesis of (β,β′,β″-Trifluoro)-*tert*-butyl Benzenes **18** and **22**

Optimization of the trifluorination reaction of **17** to **18** was explored with both cesium (CsF) and potassium (KF) fluorides and with polar aprotic solvents (DMF and DMSO), as summarized in Table 1. KF was not suitable even with 18-crown-6, and in the event the most efficient transformations were achieved with CsF in DMSO at 120 °C, which showed a modest improvement over DMF. TBAF addition improved the DMF reaction; however, there was no particular advantage in TBAF addition to DMSO. Lower temperatures led to more sluggish reactions.

**Table 1:** Development of the Fluorination of Tritosylate **17** to Generate **18**^a^

reaction	fluoride	solvent	additive	conv.
A	KF	DMF	18-crown-6	1%
B	KF	DMSO	18-crown-6	<1%
C	CsF	DMF	none	89%
D	CsF	DMSO	none	98%
E	CsF	DMSO	20% TBAF	99%
F	CsF	DMF	20% TBAF	99%
G	CsF	DMF	18-crown-6	89%
H	CsF	DMSO	18-crown-6	95%

a Reactions were conducted at 120 °C.

The route was adapted too to the *para*-bromoaryl substitution such that acetaldehyde **19** was progressed to aryl bromide **22**, which was explored for cross-coupling reactions^15,16^ to generate products **23**–**35**, as summarized in Scheme 3.

**Scheme 3:**
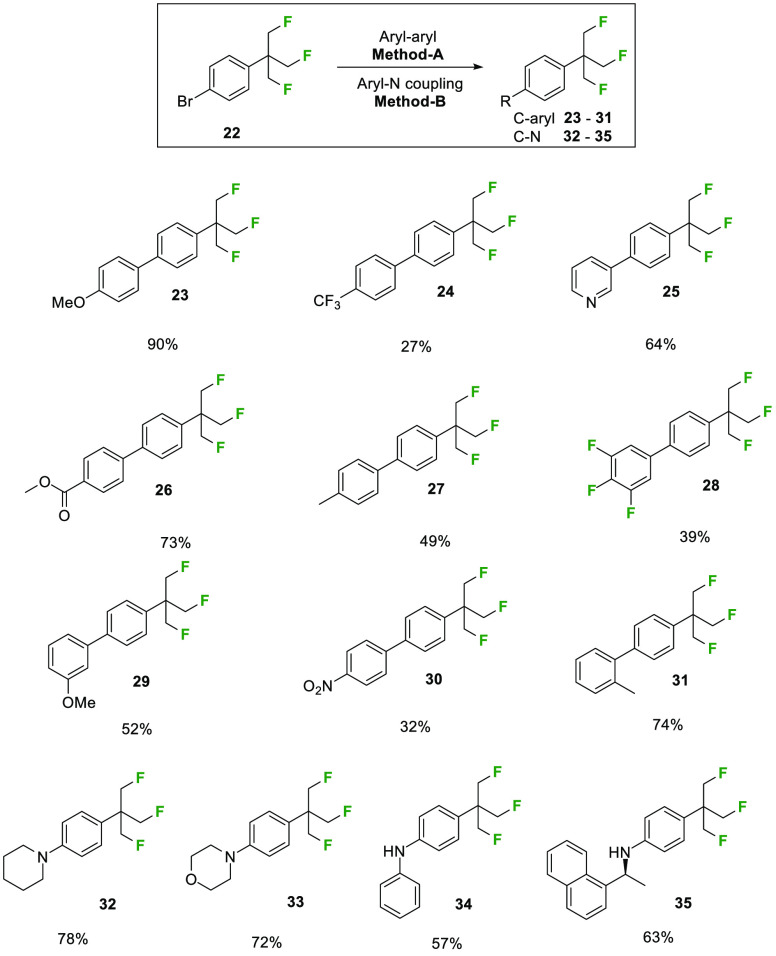
Cross-Coupling Reactions with **22** Method A: Pd(PPh_3_)_4_, K_2_CO_3_, THF/H_2_O (3:1), 80 °C, 18 h. Method B: Pd(OAc)_2_, Xantphos, Cs_2_CO_3_, 1,4-dioxane, 100 °C, 18 h.

An X-ray structure of Suzuki product **29** was determined, as illustrated in Figure 1. This gave the first insight into the preferred conformation of the aryl-TFTB motif.

**Figure 1:**
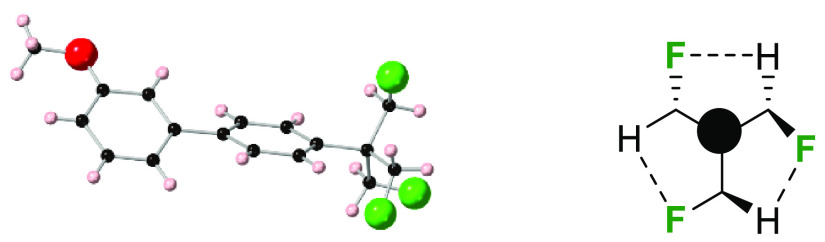
X-ray structure of **29** showing a propeller arrangement of the aryl-TFTB motif and a graphic illustrating attractive CH···FC interactions.

The structure indicates a preference for each of the C–F bonds to lie approximately on an *xyz*-axis relative to each other in a propeller arrangement, essentially with each fluorine atom orienting away from each other, and is consistent with electrostatic repulsions between the fluorines. Notably, each of the C–F bonds also lies approximately parallel (C–F···H–C ∼ 11°) to a C–H bond on a neighboring fluoromethyl group. These interactions are accommodated because the hydrogens of the C–H bonds are polarized by the electronegativity of their geminal fluorine such that there is the potential for electrostatic attraction between these electropositive hydrogens and the fluorines on an adjacent fluoromethyl group.

The conformational space for the aryl-TFTB substituent was explored using a Grimme’s iterative workflow approach with static metadynamics simulations as implemented in CREST software^17^ for the parent compound **18**. The global minimum was used to explore the energetic minima and maxima along the full rotation coordinate of one C–CH_2_F bond, and each stationary point was optimized at the M06-2X/def2-TZVP theory level.^18^ Thermal corrections were obtained from frequency calculations at standard temperatures and pressures and also at the M06-2X/def2-TZVP level.

The rotational energy profile in Figure 2a illustrates that there are three eclipsing barriers, the highest of which has Δ*G*^*‡*^ = 6.5 kcal mol^–1^. The TFTB motif conformation from the global minimum **C** is similar to that observed from the X-ray-derived crystal structure of **29**, showing good agreement between experimental and calculated C–F···HC contact distances (Figure 2b).

**Figure 2:**
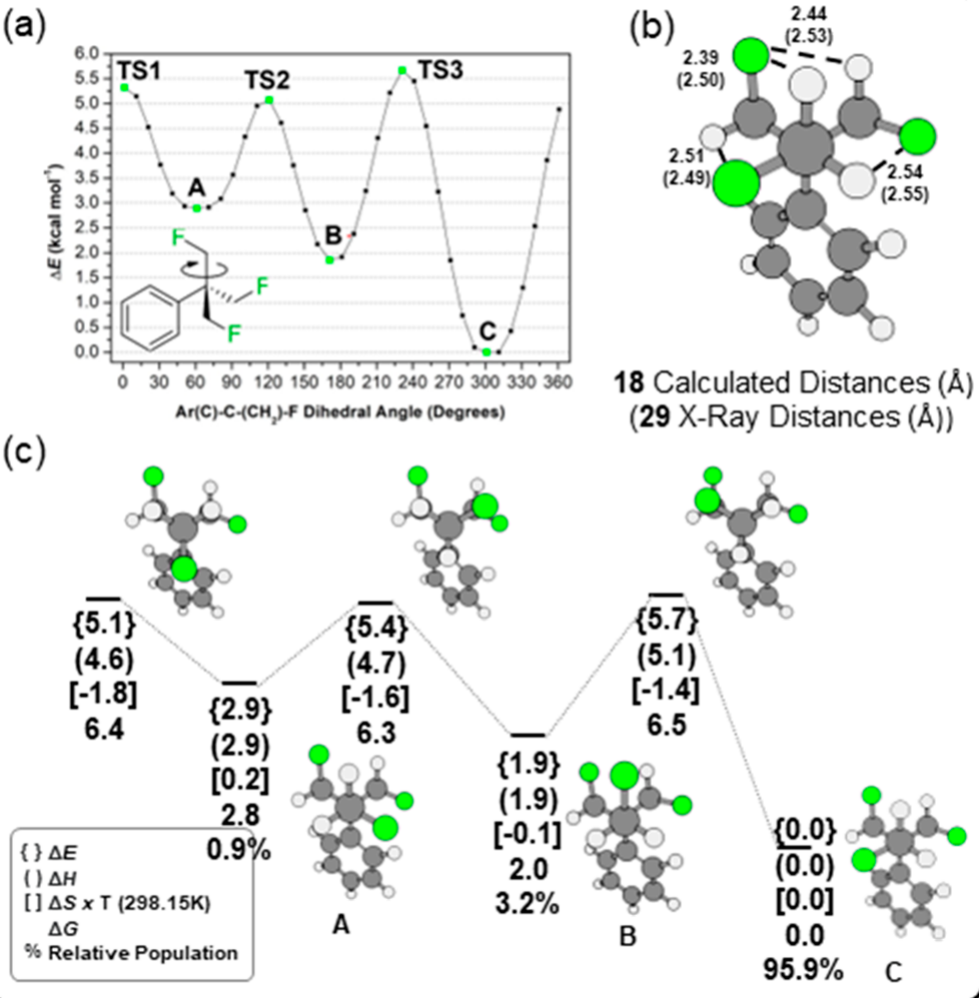
(a) Calculated rotational energy profile of **18** rotating around one of the C–CH_2_F bonds. (b) Comparison of C–F•••H–C contact distances (Å) between the calculated structure for **18** and the X-ray derived structure of the analogue **29**. (c) Thermodynamic parameters calculated for minima and maxima of the rotational energy profile in panel a in kcal mol^–1^; minima are labeled from **A** to **C** in decreasing relative energies. Calculations were carried out at the M06-2X/def2-TZVP theoretical level.^17,18^

The parallel alignment of C–F and C–H bonds in **C** results in a total of four CF···HC electrostatic contacts, each accounting for −8.9 kcal mol^–1^ of electrostatic stabilization as calculated by the classic Coulomb equation using NPA-derived atomic charges from NBO analysis.^19^ Upon rotation of the C–CH_2_F bond, repulsive contacts from the alignment of C–F bonds start to emerge in conformers **A** and **B** and lead to electrostatically destabilizing CF···FC interactions of +10.0 kcal mol^–1^ in each contact.

Non-covalent interaction analysis (NCI)^20^ also reveals the attractive and repulsive nature of the CF···HC and CF···FC interactions, respectively, as illustrated in Figure 3. In the CF···HC contact, the region characterized by sin(λ_2_)ρ ∼ −0.015 au indicates the presence of an attractive interaction between F and H atoms, which are weaker in conformer **A** than in **C**, as indicated by the higher values of the reduced density gradient (*s*) for the CF···HC contact in **A**. On the other hand, the CF···FC contact is characterized by a region of sin(λ_2_)ρ ∼ +0.025 au, which indicates the presence of repulsive interactions between F atoms. In conformer **A**, the reduced density gradient approaches zero for the CF···FC contact, indicating stronger repulsive interactions compared to those in conformer **C**. Overall, NCI analysis is in accordance with NBO and reinforces the importance of the electrostatic CF···HC and CF···FC interactions in determining the conformational equilibria between **A**, **B**, and **C**.

**Figure 3:**
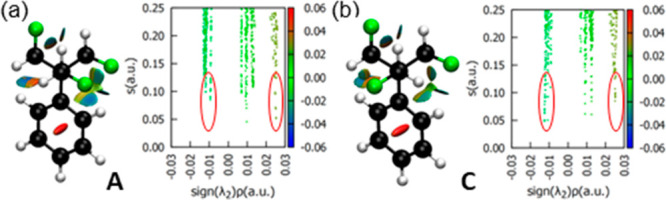
NCI iso-surfaces plotted using a reduced density gradient (*s*) of 0.5 au and a blue–green–red color scale of −0.020 < sin(λ_2_)ρ < +0.020 au (left) and *s vs* sin(λ_2_)ρ graphs (right) obtained from the M06-2X/def2-TZVP electron density for (a) conformer **A** and (b) conformer **C**.

Given the calculated relative Gibbs free energies between the minima **A-C** it is estimated that conformer **C** will dominate (96%) while conformers **A** (1%) and **B** (3%) will be minor contributors. The tendency toward a clearly preferred conformer reflects favorably on the potential of the TFTB substituent in the discovery of bioactives.

It was of interest too to explore the effects of fluorination on Log *P*. To this end. Log *P* values were evaluated experimentally for phenyl derivative **18** by reverse-phase HPLC (MeCN/water, C_18_ column)^3^ and compared with those of the corresponding aryl *tert*-butyls **36** and **37** with two and one fluoromethyl groups, respectively, and also relative to *tert*-butyl benzene **38**. The outcomes are summarized in Figure 4.

**Figure 4:**
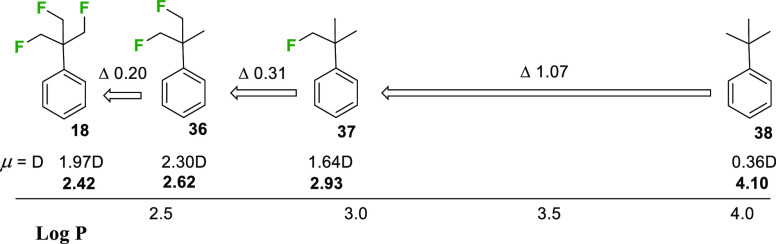
Log *P* and molecular dipole values for progressively fluorinated *tert*-butylbenzenes determined by reverse-phase HPLC.^3^

The replacement of one methyl of *tert*-butylbenzene **38** for a fluoromethyl group in **37** results in significant decrease of Log *P*, in this case by an order of magnitude (ΔLog *P* = 1.07), as illustrated in Figure 4. Log *P* reductions in changing −CH_3_ to −CH_2_F in molecular matched pairs are well-known,^21^ and there is good evidence that the effect is supported significantly by an increase in overall molecular dipole moment.^21b^ Exchange of the second and third methyl groups for fluoromethyls in **36** and **18**, respectively, continues the trend toward increasing hydrophilicity (lower Log *P*), although notably there is no longer a consistent increase in the molecular dipole moment. The difluoro analogue **36** is more polar again (μ = 2.3 D calculated for its minimum energy conformation; see Figure S10); however, the aryl-TFTB **18** becomes less polar as the individual C–F dipoles cancel each other, and there are compensating electrostatic interactions between the coaligned C–F and C–H bonds as discussed above. Nonetheless, **18** has the lowest Log *P* value and is the most hydrophilic of the series, presumably because there are six geminal and polarized hydrogens that can make electrostatic interactions with water. Solubility was not addressed comprehensively here; however, the TFTB-biphenyl ether **23** was more soluble (11 mg mL^–1^) in water than its corresponding *tert*-butyl analog (6 mg mL^–1^) [see SI].

There is a growing concern regarding persistent fluorochemicals,^13^ and it is becoming increasingly important that any new motifs that contain fluorine should be able to metabolize.^22^ To this end we have explored the metabolism of both *tert*-butylbenzene **38** as a control and then **18** in cultures of *Cunninghamella elegans*, a fungus that has been used to model human metabolism, as it is rich in P-450 activity.^23^ Aliquots of **38** and **18** were subject to incubations with *C. elegans* under previously established protocols (see the SI). After up to three days of incubation, the fungal culture supernatants were extracted into ethyl acetate. *tert*-Butylbenzene **38** was completely metabolized after three days. Aryl-TFTB **18** was more slowly, but significantly, metabolized (∼60%), and the organofluorine metabolite profile was assessed by ^19^F NMR and GC-MS. The major metabolite was determined to be alcohol **41**, the identity of which was confirmed by independent synthesis (see the SI). Alcohol **41** could clearly arise after P-450 hydroxylation of a fluoromethyl group to generate **39** and then collapse to aldehyde **40**, with HF elimination followed by biocatalytic reduction, as illustrated in Scheme 4. Consistent with this hypothesis, a metabolite with the mass of aldehyde **40** was identified as a minor metabolite by GC-MS (Figure S3). This study indicates that the TFTB motif is amenable to metabolism and should not present a persistence concern.

**Scheme 4:**
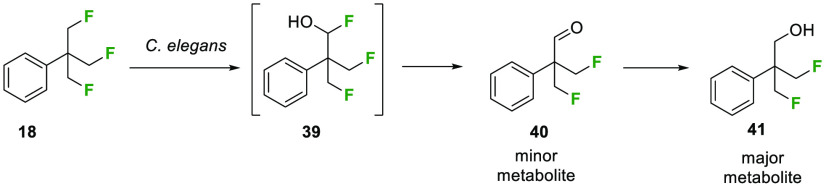
Metabolism of **18** in *C. elegans* Generated Alcohol **41** as the Major Metabolite

Finally, a synthesis of an aryl-TFTB analog **49** of the *tert*-butyl containing pesticide pyridaben **50**(24) was demonstrated and is illustrated in Scheme 5. Pyridaben is among the most widely used acaricides globally.^24b^ One objective in developing this route was to establish a protocol to benzyl bromide **47**, as this offers an intermediate for the more general introduction of the aryl-TFTB motif. Several approaches were explored for the benzylic bromination of **46**, and the most efficient conditions used the method previously described by Golding *et al*.^25^ Benzyl bromide **47** was then combined with thiol **48** to generate **49**.

**Scheme 5:**
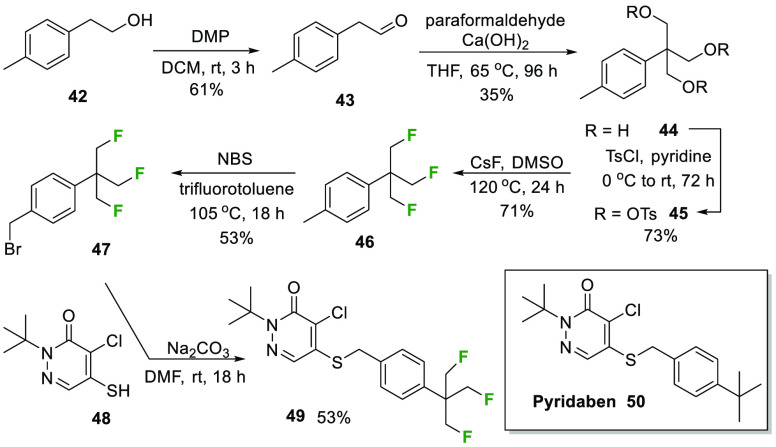
Synthesis of the (β,β′,β″-Trifluoro)-*tert*-butyl Pyridaben Analog **49** of Pyridaben **50**

In conclusion, we present an amenable route to the aryl (β,β′,β″-trifluoro)-*tert*-butyl (TFTB) motif and explore Pd-cross coupling reactions on bromoaryl derivatives. An analogue **49** of the pesticide pyridaben **50** is prepared to exemplify an additional approach to incorporating TFTB through benzyl bromide **47**. X-ray structure analysis and DFT computation indicate that the aryl *tert*-(β,β′,β″-trifluoro)butyl (TFTB) substituent is found to have a favored conformation, which is dictated by electrostatic repulsion between the fluorines and also stabilized by compensating electrostatic interactions between polarised C−F and C−H bonds hydrogen. The progressive switch of methyl for fluoromethyl groups in going from *tert*-butylbenzene **38** to the analogous TFTB benzene **18** resulted in over an order of magnitude reduction in Log *P*. In addition, it is demonstrated that the aryl-TFTB group is significantly metabolized in cultures of *C. elegans* and will not be a persistent organofluorine. Collectively, these aspects should be attractive for the exploration of the aryl-TFTB motif more widely in the discovery of bioactives.

## Data Availability

The data underlying this study are available in the published article and its Supporting Information.

## Supporting Information Available

Synthesis, computational methods, biotransformations and details of X-ray structure analysis (PDF)
