# Supplementary material for: Aryl (β,β′,β″-Trifluoro)-tert-butyl: A Candidate Motif for the Discovery of Bioactives
Source: Org Lett. 2023 Sep 8;25(37):6802–7. doi: 10.1021/acs.orglett.3c02236 (PMC10521027; doi:10.1021/acs.orglett.3c02236)
Supplement: Supplementary file 1 — ol3c02236_si_001.pdf [file ol3c02236_si_001.pdf]

Supporting Information

Aryl *tert*-(β,β',β'' trifluoro)butyl; a candidate motif for bioactives discovery

Luca S. Dobson,[a] Qingzhi Zhang,[a] Benjamin A. McKay,[a] Oluwayinka Oke, [a],[b] Chukwuemeka Isanbor,[b] Mohd Faheem Khan,[c] Bruno A. Piscelli,[d] David B. Cordes,[a] Rodrigo A. Cormanich,[d] Cormac D. Murphy[c] and David O'Hagan*[a]

[a] *L. Dobson, Dr Qing Zhang, B. A. McKay, O. Oke, Dr. D. B. Cordes, Prof Dr D. O'Hagan*
School of Chemistry, University of St Andrews, North Haugh, St Andrews, KY16 9ST, UK.
E-mail: do1@st-andrews.ac.uk
[b] *O. Oke, Prof. Dr C Isanbor.*
University of Lagos, Chemistry Department, Akoka, Lagos, 101245, Nigeria.
[c] *University College Dublin, School of Biomolecular and Biomedical Science, Belfield, Dublin 4, Ireland.*
[d] *University of Campinas, Chemistry Institute, Monteiro Lobato Street, Campinas, Sao Paulo, Brazil - 13083-862.*

Contents

Synthetic protocols Page 2 – 19

Biotransformation with *Cunninghamella elegans* DSM1908 Page 20 – 22

Solubility measurement Page 23

Crystallographic Data for compound 29 Page 24 - 25

Computational details for conformational analysis of compound 18 Page 26 – 33

Images of NMR spectra for synthesised compounds Page 34 – 111

Synthetic protocols

General

NMR Spectra were recorded on Bruker AVIII 500, AVIII-HD 500 or AVII 400 spectrometers. NMR analyses were carried out at room temperature in indicated deuterated solvents unless otherwise noted. Chemical shift data are reported as δ in units of ppm relative to respective deuterated NMR solvent. Coupling constant J was reported in Hz. ^{19}F NMR spectra were recorded relative to CCl_3F ($\delta F = 0.00$ ppm). Structural assignments were made with additional information from gCOSY, gHSQC, and gHMBC experiments in conjunction with ^{1}H, ^{13}C, and ^{19}F NMR data.

All reactions were carried out under an argon atmosphere with standard Schlenk techniques unless otherwise specified. The reaction glassware was flame dried or oven dried and cooled under vacuum. Commercially available chemicals were purchased from Acros, Alfa Aeser, Fisher Scientific, Fluorochem, Sigma Aldrich, Strem Chemicals, and TCI (UK) and used as received unless otherwise stated. Melting points were recorded on an Electrothermal 9100 melting point apparatus and measurements of samples were taken directly. IR were recorded on a Shimadzu IR-Affinity-1S spectrometer with a diamond ATR attachment. Spectra were recorded of either thin films or solids, with characteristic absorption wavenumbers (ν max) reported in cm^{-1}. Optical rotation was performed using a Perkin Elmer Model 341 polarimeter, and values were recorded at 20°C and 589 nm using a 1 dm cell; $[\alpha]^{20}_{D}$ values are reported in units of 10^{-1} $deg.cm^2.g^{-1}$.

DCM and THF were dried and deoxygenated using an MBraun SPS-800 solvent system. *Concentrated under reduced pressure* refers to the use of rotary evaporator with membrane pump at 30-50 mbar. Analytical thin-layer chromatography was carried out on aluminium backed Merck TLC silica gel 60 F254 plates. These plates were visualised using UV light at 254 nm wavelength or dyed by potassium permanganate followed by air dryer heating. Flash column chromatography was performed using Merck Geduran silica gel 60 Å (250-400 mesh), using either manual glass columns or a Biotage Selekt 2 automatic chromatography system eluting with solvents as reported. High resolution mass spectra were recorded on a Thermo Scientific Exactive orbitrap mass spectrometer by the University of St Andrews, UK.

2-(Hydroxymethyl)-2-phenylpropane-1,3-diol (16)[1]

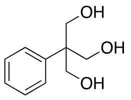

Phenylacetaldehyde (5.0 mL, 8.57 mmol) and calcium hydroxide (12.7 g, 42.9 mmol) were added to a suspension of paraformaldehyde (5.15 g, 171.5 mmol) in THF (65 mL). The reaction mixture was stirred at 60-65 ^{o}C for four days. After cooling to room temperature, the reaction mixture was filtered through celite and the celite was rinsed with DCM (50 mL). The filtrate was concentrated under reduced pressure to give the crude product. The crude product was purified by column chromatography over silica gel (1:1 hexane/ethyl acetate to 100% ethyl acetate) to give the desired product as a white solid (1.02 g, 65.3%). ^{1}H NMR (400 MHz, d^{6}-DMSO) δ 7.39-7.43 (2H, m, $C\underline{H}C$), 7.23-7.28 (2H, m, $C\underline{H}CHC$), 7.12-7.17 (1H, m, $C\underline{H}CHCHC$), 4.41 (3H, t, J 5.2 Hz, $O\underline{H}$), 3.71 (6H, d, J 5.2 Hz, CH_2).

2-Phenyl-2-((tosyloxy)methyl)propane-1,3-diyl bis(4-methylbenzenesulfonate) (17)[1]

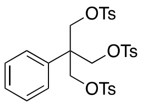

p-Toluenesulfonyl chloride (11.0 g, 57.8 mmol) was added slowly to a solution of 2-(hydroxymethyl)-2-phenylpropane-1,3-diol (2.23 g, 12.3 mmol) in pyridine (25.3 mL) at 0 ^{o}C and the reaction was warmed to room temperature and stirred for three days. On completion, the reaction mixture was concentrated under reduced pressure. The resulting residue was dissolved in DCM (250 mL) and the suspension was filtered. The filtrate was washed with water (250 mL). The organic phase was separated, and the aqueous phase was extracted with DCM (3 x 75 mL). The combined organic phases were washed with brine (125 mL), dried over magnesium sulfate, filtered, and concentrated under vacuum to give the crude product. The crude product was suspended in ethanol (37 mL) and the suspension was stirred vigorously and heated to 92 ^{o}C. The suspension was filtered to give the desired product as a white solid (2.81 g, 35.5%). ^{1}H NMR (500 MHz, $CDCl_3$) δ 7.62-7.66 (6H, m, ArH), 7.31-7.34 (6H, m, ArH), 7.15-7.22 (3H, m, ArH), 6.93-6.98 (2H, m, ArH), 4.20 (6H, s, CH_2), 2.48 (9H, s, CH_3).

(1,3-Difluoro-2-(fluoromethyl)propan-2-yl)benzene (18)

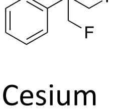

Cesium fluoride (8.48 g, 55.8 mmol) was added to a solution of 2-phenyl-2-((tosyloxy)methyl)propane-1,3-diyl bis(4-methylbenzenesulfonate) (2.00 g, 3.10 mmol) in DMSO (68 mL) and the reaction was heated to 120 ^{o}C and stirred for 24 h. On completion, the reaction mixture was diluted with water and the aqueous layer was extracted with diethyl ether (3x). The combined organic layer was washed with water, dried over magnesium sulfate, filtered and concentrated under vacuum to give the crude product. The crude product was

purified by column chromatography over silica gel (hexane/ethyl acetate, gradient from 0 to 10% ethyl acetate) to give the desired product as a yellow oil (417.8 mg, 71.4%). IR ν_{max}/cm^{-1} 2970 (C-H), 2922 (C-H), 2361, 1717, 1028 (C-F), 1005 (C-F), 698. ^{1}H NMR (400 MHz, $CDCl_3$) δ 7.38-7.44 (4H, m, ArH), 7.31-7.37 (1H, m, ArH), 4.81 (6H, dt, J 46.9, 1.5 Hz, CH_2F). ^{13}C NMR (101 MHz, $CDCl_3$) δ 136.3 (<u>C</u>CCH$_2$), 129.1, 128.2, 127.0, 83.3 (3C, dt, J 176.2, 5.9 Hz, CH_2F), 48.9 (apparent q, J 17.1 Hz, <u>C</u>CH$_2$F). ^{19}F NMR (376 MHz, $CDCl_3$), δ -230.9 (3F, t, J 46.9 Hz, CH_2F). HRMS (EI): calculated for $C_{10}H_{11}F_3$, 188.0807; found 188.0807.

2-(4-Bromophenyl)acetaldehyde (19)[2]

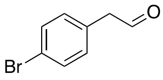

Dess-Martin Periodinane (12.6 g, 30.3 mmol) was added to a stirred solution of 2-(4-bromophenyl)ethanol (3.86 mL, 27.5 mmol) in DCM (275 mL) and the reaction was stirred for 3 hours. The reaction was quenched by addition of sat. aq. sodium thiosulfate and sat. aq. sodium hydrogen carbonate. The aqueous layer was extracted with DCM (3x). The combined organic layers were washed with $NaHCO_3$ and brine, dried over sodium sulfate, filtered and concentrated to give the crude product. The crude product was dissolved in 9:1 hexane/ethyl acetate solution and filtered. The filtrate was concentrated under reduced pressure to give the desired product as a colourless liquid (4.53 g, 75.1%). ^{1}H NMR (400 MHz, $CDCl_3$) δ 9.74 (1H, t, J 1.9 Hz, CHO), 7.47-7.52 (2H, m, ArH), 7.09 (2H, d, J 8.9 Hz, CHCBr), 3.69 (2H, d, J 1.9 Hz, CH_2).

2-(4-Bromophenyl)-2-(hydroxymethyl)propane-1,3-diol (20)[3]

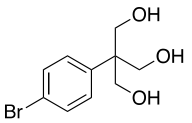

2-(4-Bromophenyl)acetaldehyde (4.53 g, 22.8 mmol) and calcium hydroxide (6.75 g, 94.0 mmol) were added to a suspension of paraformaldehyde (2.73 g, 91.0 mmol) in THF (35 mL). The reaction mixture was stirred at 60-65 ^{o}C for four days. After cooling to room temperature, the reaction mixture was filtered through celite and the celite was rinsed with DCM (50 mL). The filtrate was concentrated under reduced pressure to give the crude product. The crude product was purified by column chromatography over silica gel (1:1 hexane/ethyl acetate gradient to 100% ethyl acetate) to give the desired product as a white solid (2.99 g, 50.2%). m.p. 117-119 ^{o}C. IR ν_{max}/cm^{-1} 2868 (C-H), 1767 (C-H), 1580, 1454 (O-H), 1361 (O-H), 1132 (C-O), 1042 (C-O), 775, 750 (C-Br). ^{1}H NMR (400 MHz, d^{6}-DMSO) δ 7.42-7.46 (2H, m, ArH), 7.35-7.39 (2H, m, ArH), 4.46 (3H, t, J 5.2 Hz, OH), 3.67 (6H, d, J 5.2 Hz, CH_2). HRMS (ESI) calculated for $C_{10}H_{13}O_3Br^{79}Na^+$: 282.9940; found 282.9938.

2-(4-Bromophenyl)-2-((tosyloxy)methyl)propane-1,3-diyl bis(4-methylbenzenesulfonate) (21)

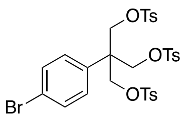

p-Toluenesulfonyl chloride (9.47 g, 49.7 mmol) was added slowly to a solution of 2-(4-bromophenyl)-2-(hydroxymethyl)propane-1,3-diol (2.99 g, 10.6 mmol) in pyridine (21.9 mL) at 0 °C and the reaction was warmed to room temperature and stirred for three days. On completion, the reaction mixture was concentrated under reduced pressure. The resulting residue was dissolved in DCM (220 mL) and the suspension was filtered. The filtrate was washed with water (220 mL). The organic phase was separated, and the aqueous phase was extracted with DCM (3 x 65 mL). The combined organic phases were washed with brine (110 mL), dried over magnesium sulfate, filtered and concentrated under vacuum to give the crude product. The crude product was suspended in ethanol (30 mL) and the suspension was stirred vigorously and heated to 92 °C. The suspension was filtered to give the desired product as a white solid (4.78 g, 62.3%). m.p. 134-136 °C. IR ν_{max}/cm^{-1} 1356 (S=O), 1171 (C-O), 989, 968, 820, 667, 548. ^{1}H NMR (400 MHz, $CDCl_3$) δ 7.59-7.62 (6H, m, ArH), 7.30-7.33 (6H, m, ArH), 7.22-7.25 (2H, m, ArH), 6.76-6.79 (2H, m, ArH), 4.15 (6H, s, CH_2O), 2.47 (9H, s, CH_3). ^{13}C NMR (126 MHz, $CDCl_3$) δ 145.6, 133.9, 131.9, 131.6, 130.1, 127.93, 127.88, 122.4, 68.5 (CH_2O), 46.0 ($\underline{C}CH_2O$), 21.8 (Ar$\underline{C}H_3$). HRMS (ESI$^+$) calculated for $C_{31}H_{31}O_9Br^{79}S_3Na^+$: 745.0182; found 745.0199.

1-Bromo-4-(1,3-difluoro-2-(fluoromethyl)propan-2-yl)benzene (22)

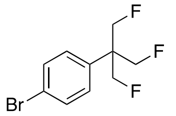

Cesium fluoride (1.89 g, 12.4 mmol) was added to a solution of 2-phenyl-2-((tosyloxy)methyl)propane-1,3-diyl bis(4-methylbenzenesulfonate) (500 mg, 0.691 mmol) in DMSO (15 mL) and the reaction was heated to 120 °C and stirred for 24 h. On completion, the reaction mixture was diluted with water and the aqueous layer was extracted with diethyl ether (3x). The combined organic layer was washed with water, dried over magnesium sulfate, filtered and concentrated under vacuum to give the crude product. The crude product was purified by column chromatography over silica gel (hexane/ethyl acetate, gradient from 0 to 10% ethyl acetate) to give the desired product as a yellow oil (136.2 mg, 73.8%). IR ν_{max}/cm^{-1} 2972, 2916 (C-H), 1497, 1072 (C-F), 1028 (C-F), 1003 (C-F), 818 (C-Br). ^{1}H NMR (400 MHz, $CDCl_3$) δ 7.51-7.56 (2H, m, ArH), 7.28-7.32 (2H, m, ArH), 4.77 (6H, dt, J 46.9, 1.6 Hz, CH_2F). ^{13}C NMR (126 MHz, $CDCl_3$) δ 135.4, 132.2, 128.8, 122.4, 82.9 (3C, dt, J 177.1, 5.9 Hz, CH_2F), 41.1 ($\underline{C}CH_2F$). ^{19}F NMR (376 MHz, $CDCl_3$) δ -231.0 (3F, t, J 47.2 Hz, CH_2F). HRMS (EI) calculated for $C_{10}H_{10}Br^{79}F_3^+$: 265.9913; found 265.9914.

General procedure for Suzuki cross-coupling reactions (General Method A)

Tetrakis(triphenylphosphine)palladium(0) (5 mol%) was added to a solution of 1-bromo-4-(1,3-difluoro-2-(fluoromethyl)propan-2-yl)benzene (1 eq.), boronic acid (1.2 eq.) and potassium carbonate (2 eq.) in 3:1 THF/water solution (0.2 M) and the reaction was heated to reflux and stirred for 18 h. Subsequently, the reaction was cooled to room temperature and diluted with water. The aqueous layer was extracted with DCM (3x). The combined organic layers were washed with brine, dried over sodium sulfate, filtered and concentrated under vacuum to give the crude product. The crude was purified by column chromatography over silica gel (hexane/DCM 7:3).

4-(1,3-Difluoro-2-(fluoromethyl)propan-2-yl)-4'-methyl-1,1'-biphenyl (23)

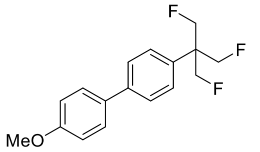

This compound was prepared according to General Procedure A. The compound was isolated as a white solid (31.9 mg, 90.3%). m.p. 118-120 °C. IR ν_{max}/cm^{-1} 2920, 2853 (C-H), 1604, 1501, 1253, 1186, 1028, 1003, 821, 810. ^{1}H NMR (500 MHz, $CDCl_3$) δ 7.57-7.61 (2H, m, ArH), 7.51-7.54 (2H, m, ArH), 7.45-7.48 (2H, m, ArH), 6.97-7.00 (2H, m, ArH), 4.84 (6H, dt, J 47.0, 1.5 Hz, CH_2), 3.86 (3H, s, CH_3). ^{13}C NMR (126 MHz, $CDCl_3$) δ 140.5, 134.4, 132.8, 128.1, 127.3, 127.2, 114.3, 83.2 (3C, dt, J 176.7, 5.9 Hz, $\underline{C}H_2F$), 55.4 (apparent q, J 16.5 Hz, $\underline{C}CH_2F$), 29.7 (CH_3). ^{19}F NMR (377 MHz, $CDCl_3$) δ -230.8 (3F, t, J 47.0 Hz, CH_2F). HRMS (ESI) calculated for $C_{17}H_{17}OF_3Na^+$: 317.1129; found 317.1125.

Methyl 4'-(1,3-difluoro-2-(fluoromethyl)propan-2-yl)-[1,1'-biphenyl]-4-carboxylate (24)

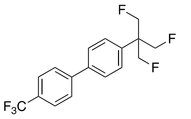

This compound was prepared according to General Procedure A. The compound was isolated as a white solid (12.3 mg, 27.4%). m.p. 72-74 °C. IR ν_{max}/cm^{-1} 2961, 2916 (C-H), 1616 (C=C), 1325 (C-F), 1165, 1138, 1126, 1109, 1066, 1016, 1004, 822. ^{1}H NMR (500 MHz, $CDCl_3$) δ 7.66-7.73 (4H, m, ArH), 7.62-7.66 (2H, m, ArH), 7.51-7.56 (2H, m, ArH), 4.85 (6H, dt, J 46.9, 1.5 Hz, CH_2). ^{13}C NMR (126 MHz, $CDCl_3$) δ 143.8, 139.4, 129.6 (q, J 32.6 Hz, CF_3), 127.8, 127.6, 127.4, 125.8 (q, J 3.7 Hz, $\underline{C}CF_3$), 83.0 (3C, dt, J 176.2, 5.9 Hz, $\underline{C}H_2F$), 48.8 (q, J 16.8 Hz, $\underline{C}CH_2F$). ^{19}F NMR (376 MHz, $CDCl_3$) δ -62.4 (3F, s, CF_3), -230.9 (3F, t, J 47.0 Hz, CF_2H). HRMS (ESI$^+$) calculated for $C_{17}H_{14}F_6Na^+$: 355.0897; found 355.0892.

3-(4-(1,3-Difluoro-2-(fluoromethyl)propan-2-yl)phenyl)pyridine (25)

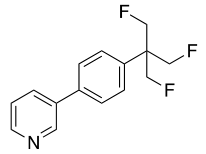

This compound was prepared according to General Procedure A. The compound was isolated as a white solid (43.1 mg, 64.4%). m.p. 95-97 °C. IR ν_{max}/cm^{-1} 2972, 2911, 1474, 1437, 1186, 1119, 1026, 1001, 802, 719, 711. ^{1}H NMR (500 MHz, $CDCl_3$) δ 8.85 (1H, m, ArH), 8.61 (1H, m, ArH), 7.87 (1H, dt, J 7.9, 2.0 Hz, ArH), 7.63 (2H, dt, J 8.7, 2.2 Hz, ArH), 7.55 (2H, dt, J 8.3, 2.1 Hz, ArH), 7.36-7.41 (1H, m, ArH), 4.85 (6H, dt, J 46.9, 1.5 Hz, CH_2F). ^{13}C NMR (126 MHz, $CDCl_3$) δ 148.9, 148.4, 137.7, 136.3, 136.0, 134.5, 132.3, 132.2, 127.82, 127.76, 123.8, 83.2 (3C, dt, J 176.6, 5.9 Hz, CH_2F). $^{19}F\{^{1}H\}$ NMR (470 MHz, $CDCl_3$) δ -231.0 (3F, s, CH_2F). HRMS (ESI$^+$) calculated for $C_{15}H_{14}F_3NNa^+$: 288.0976; found 288.0971.

4-(1,3-Difluoro-2-(fluoromethyl)propan-2-yl)-4'-methyl-1,1'-biphenyl (26)

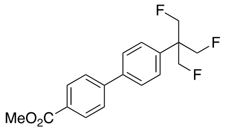

This compound was prepared according to General Procedure A. The compound was isolated as a white solid (39.3 mg, 73.0%). m.p. 92-94 °C. IR ν_{max}/cm^{-1} 2956, 2922, 2853 (C-H), 1714 (C=O), 1436 (C-H), 1280s (C-F), 1101 (C-O). ^{1}H NMR (500 MHz, $CDCl_3$) δ 8.10-8.13 (2H, m, ArH), 7.64-7.68 (4H, m, ArH), 7.51-7.54 (2H, m, ArH), 4.84 (6H, dt, J 46.9, 1.6 Hz, CH_2), 3.94 (3H, s, CH_3). ^{13}C NMR (126 MHz, $CDCl_3$) δ 166.9 (C=O), 144.7, 139.7, 136.1, 130.2, 129.2, 127.7, 127.5, 127.0, 83.0 (dt, J 176.2, 5.4 Hz, CH_2F), 52.2 (CH_3), 48.7 (apparent q, J 16.9 Hz, $\underline{C}CH_2F$). $^{19}F\{^{1}H\}$ NMR (376 MHz, $CDCl_3$) δ -230.9 (3F, s, CH_2F). HRMS (ESI) calculated for $C_{18}H_{18}F_3O_2^+$: 323.1259; found 323.1250.

4-(1,3-Difluoro-2-(fluoromethyl)propan-2-yl)-4'-methyl-1,1'-biphenyl (27)

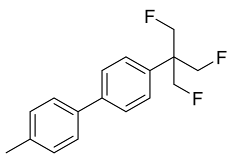

This compound was prepared according to General Procedure A. Tetrakis(triphenylphosphine)palladium(0) (14.6 mg, 5 mol%) was added to a solution of 1-bromo-4-(1,3-difluoro-2-(fluoromethyl)propan-2-yl)benzene (67.4 mg, 0.252 mmol), 4-tolylboronic acid (41.1 mg, 0.302 mmol) and potassium carbonate (69.7 mg, 0.504 mmol) in 3:1 THF/water solution (1.25 mL) and the reaction was heated to reflux and stirred for 18 h. Subsequently, the reaction was cooled to room temperature and diluted with water. The aqueous layer was extracted with DCM (3x). The combined organic layers were washed with

brine, dried over sodium sulfate, filtered and concentrated under vacuum to give the crude product. The crude was purified by column chromatography over silica gel (hexane/DCM 7:3). The compound was isolated as a white solid (34.6 mg, 49.3%). m.p. 98-100 °C. IR ν_{max}/cm^{-1} 2972, 2918 (C-H), 1501 (C=C), 1028, 991, 810. ^1H NMR (500 MHz, $CDCl_3$) δ 7.60-7.64 (2H, m, ArH), 7.46-7.53 (4H, m, ArH), 7.25-7.29 (2H, m, ArH), 4.85 (6H, dt, J 47.0, 1.5 Hz, CH_2), 2.41 (3H, s, CH_3). ^{13}C NMR (126 MHz, $CDCl_3$) δ 140.8, 137.4, 134.8, 129.6, 127.4, 127.3, 126.9, 83.1 (3C, dt, J 176.8, 5.8 Hz, $\underline{C}H_2F$), 48.6 (q, J 17.1 Hz, $\underline{C}CH_2$), 21.2 (CH_3). $^{19}F\{^1H\}$ NMR (376 MHz, $CDCl_3$) δ -230.8 (3F, s, CH_2F). HRMS (ESI) calculated for $C_{17}H_{17}F_3Na^+$: 301.1175; found 301.1169.

4'-(1,3-Difluoro-2-(fluoromethyl)propan-2-yl)-3,4,5-trifluoro-1,1'-biphenyl (28)

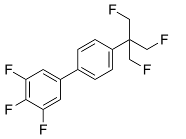

This compound was prepared according to General Procedure A. The compound was isolated as a yellow oil (10.1 mg, 39.2%). ^1H NMR (400 MHz, $CDCl_3$) δ 7.49-7.56 (4H, m, ArH), 7.15-7.21 (2H, m, ArH), 4.83 (6H, dt, J 46.8, 1.5 Hz, CH_2F). $^{19}F\{^1H\}$ NMR (376 MHz, $CDCl_3$) δ -231.0 (3F, s, CH_2F), -162.1 (1F, t, J 20.2 Hz, $C\underline{F}$), -133.8 (2F, d, J 20.2 Hz, $CHC\underline{F}$). HRMS (ESI^+) calculated for $C_{16}H_{12}F_6Na^+$: 341.0741; found 341.0735.

4'-(1,3-Difluoro-2-(fluoromethyl)propan-2-yl)-3-methoxy-1,1'-biphenyl (29)

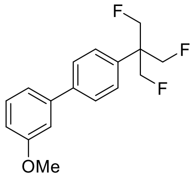

This compound was prepared according to General Procedure A. The compound was isolated as a white solid (22.8 mg, 51.6%). m.p. 70-72 °C. IR ν_{max}/cm^{-1} 2955 (C-H), 1172 (C-F), 1024 (C-F), 999, 787. ^1H NMR (500 MHz, $CDCl_3$) δ 7.61-7.65 (2H, m, ArH), 7.48-7.53 (2H, m, ArH), 7.34-7.40 (1H, m, ArH), 7.16-7.20 (1H, m, ArH), 7.11-7.14 (1H, m, ArH), 6.90-6.94 (1H, m, ArH), 4.85 (6H, dt, J 46.8, 1.6 Hz, CH_2), 3.87 (3H, s, CH_3). ^{13}C NMR (126 MHz, $CDCl_3$) δ 160.1, 141.9, 140.9, 135.4, 130.0, 127.8, 127.4, 119.7, 113.0, 83.2 (3C, dt, 176.2, 5.9 Hz, $\underline{C}H_2F$), 55.4, 48.8 (q, J 17.3 Hz, $\underline{C}CH_2F$). $^{19}F\{^1H\}$ NMR (376 MHz, $CDCl_3$) δ -230.8 (3F, s, CH_2F). HRMS (ESI) calculated for $C_{17}H_{17}F_3ONa^+$: 317.1129; found 317.1124.

4-(1,3-Difluoro-2-(fluoromethyl)propan-2-yl)-4'-nitro-1,1'-biphenyl (30)

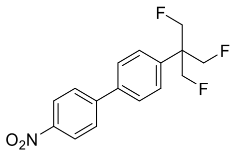

This compound was prepared according to General Procedure A. The compound was isolated as a white solid (16.4 mg, 32.4%). m.p. 114-116 °C. IR ν_{max}/cm^{-1} 2972 (C-H), 2920 (C-H), 1514 (N-O), 1341 (N-O), 1018 (C-N), 825 (C=C). ^1H NMR (400 MHz, $CDCl_3$) δ 8.29-8.33 (2H, m, ArH),

7.72-7.76 (2H, m, ArH), 7.65-7.69 (2H, m, ArH), 7.54-7.59 (2H, m, ArH), 4.85 (6H, dt, J 46.8, 1.5 Hz, CH_2F). ^{13}C NMR (126 MHz, $CDCl_3$) δ 147.4, 146.8, 138.6, 137.2, 128.5, 127.9, 124.5, 124.3, 83.1 (3C, dt, J 176.7, 5.9 Hz, CH_2F), 49.0 (apparent q, J 16.7 Hz, $\underline{C}CH_2F$). $^{19}F\{^1H\}$ NMR (376 MHz, $CDCl_3$) δ -231.0 (3F, s, CH_2F). HRMS (ESI^+) calculated for $C_{16}H_{14}F_3NO_2Na^+$: 332.0874; found 332.0869.

4'-(1,3-Difluoro-2-(fluoromethyl)propan-2-yl)-2-methyl-1,1'-biphenyl (31)

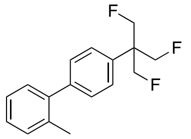

This compound was prepared according to General Procedure A. The compound was isolated as a white solid (33.6 mg, 73.6%). m.p. 66-68 °C. IR ν_{max}/cm^{-1} 2970 (C-H), 2901 (C-H), 2361, 1485, 1015 (C-F), 997, 982, 633. ^1H NMR (500 MHz, $CDCl_3$) δ 7.44-7.48 (2H, m, ArH), 7.35-7.39 (2H, m, ArH), 7.20-7.30 (4H, m, ArH), 4.86 (6H, dt, J 46.9, 1.5 Hz, CH_2), 2.28 (3H, s, CH_3). ^{13}C NMR (126 MHz, $CDCl_3$) δ 141.8, 141.2, 135.5, 134.7, 130.5, 129.8, 127.6, 126.7, 126.0, 83.3 (3C, dt, J 176.7, 5.9 Hz, CH_2F), 48.8 (apparent q, J 16.8 Hz, $\underline{C}CH_2F$), 20.6 (CH_3). $^{19}F\{^1H\}$ NMR (376 MHz, $CDCl_3$) δ -230.8 (3F, s, CH_2F). HRMS (ESI) calculated for $C_{17}H_{17}F_3Na^+$: 301.1180; found 301.1175.

General procedure for Buchwald-Hartwig cross-coupling reactions (General Method B)

1-bromo-4-(1,3-difluoro-2-(fluoromethyl)propan-2-yl)benzene (1.0 eq.), amine (1.2 eq.), palladium(II) acetate (10 mol%), Xantphos (10 mol%) and cesium carbonate (2.0 eq.) were dissolved in 1,4-dioxane (0.187 M) under nitrogen. The reaction was heated to 100 °C and stirred overnight. On completion, the reaction was cooled to room temperature and the mixture was filtered through celite, eluting with ethyl acetate. The filtrate was washed with saturated aqueous ammonium chloride and brine, dried over magnesium sulfate, filtered and concentrated under vacuum to give the crude product. The crude was purified by column chromatography over silica gel (hexane/ethyl acetate, gradient 0 to 100% ethyl acetate).

1-(4-(1,3-Difluoro-2-(fluoromethyl)propan-2-yl)phenyl)piperidine (32)

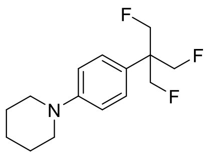

This compound was prepared according to General Procedure B. 1-bromo-4-(1,3-difluoro-2-(fluoromethyl)propan-2-yl)benzene (50.0 mg, 0.187 mmol), piperidine (22.2 μL, 0.225 mmol), palladium(II) acetate (4.20 mg, 10 mol%), Xantphos (10.8 mg, 10 mol%) and cesium carbonate (121.9 mg, 0.374 mmol) were dissolved in 1,4-dioxane (1.0 mL) under nitrogen. The reaction was heated to 100 °C and stirred overnight. On completion, the reaction was cooled to room temperature and the mixture was filtered through celite, eluting with ethyl acetate. The filtrate was washed with saturated aqueous ammonium chloride and brine, dried over

magnesium sulfate, filtered and concentrated under vacuum to give the crude product. The crude was purified by column chromatography over silica gel (hexane/ethyl acetate, gradient 0 to 100% ethyl acetate). The compound was isolated as a yellow oil (39.7 mg, 78.2%). IR ν_{max}/cm^{-1} 2931 (C-H), 1612, 1520, 1240 (C-N), 1024 (C-F), 993, 819. ^{1}H NMR (500 MHz, $CDCl_3$) δ 7.25-7.30 (2H, m, ArH), 6.91-6.96 (2H, m, ArH), 4.76 (6H, dt, J 47.0, 1.5 Hz, CH_2F), 3.15-3.19 (4H, m, $C\underline{H_2}N$), 1.67-1.73 (4H, m, $C\underline{H_2}CH_2N$), 1.55-1.61 (2H, m, $C\underline{H_2}CH_2CH_2N$). ^{13}C NMR (126 MHz, $CDCl_3$) δ 151.5, 127.6, 125.8, 116.4, 83.5 (3C, dt, J 176.2, 5.9 Hz, $\underline{C}H_2F$), 50.2, 48.1 (q, J 17.3 Hz, $\underline{C}CH_2F$), 25.8, 24.4. ^{19}F{^{1}H} NMR (376 MHz, $CDCl_3$) δ -230.5 (3F, s, CH_2F). HRMS (EI) calculated for $C_{15}H_{20}F_3N$: 271.1542; found 271.1550.

4-(4-(1,3-Difluoro-2-(fluoromethyl)propan-2-yl)phenyl)morpholine (33)

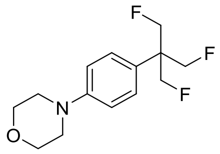

This compound was prepared according to General Procedure B. The compound was isolated as a yellow oil (36.1 mg, 71.7%). IR ν_{max}/cm^{-1} 1519, 1236 (C-N), 1119 (C-O), 1022 (C-F), 1011 (C-O), 993, 932. ^{1}H NMR (500 MHz, $CDCl_3$) δ 7.30-7.34 (2H, m, ArH), 6.90-6.95 (2H, m, ArH), 4.77 (6H, dt, J 47.0, 1.5 Hz, CH_2F), 3.83-3.88 (4H, m, OCH_2), 3.15-3.20 (4H, m, NCH_2). ^{13}C NMR (126 MHz, $CDCl_3$) δ 127.9, 115.7, 83.4 (3C, dt, J 176.2, 5.6 Hz, CH_2F), 67.0, 49.0. ^{19}F{^{1}H} NMR (376 MHz, $CDCl_3$) δ -230.6 (3F, s, CH_2F). HRMS (EI) calculated for $C_{14}H_{18}F_3NO$: 273.1335; found 273.1339.

4-(1,3-Difluoro-2-(fluoromethyl)propan-2-yl)-N-phenylaniline (34)

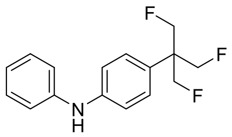

This compound was prepared according to General Procedure B. The compound was isolated as a yellow oil (21.0 mg, 56.9%). IR ν_{max}/cm^{-1} 2361, 1595 (N-H), 1520, 1497, 1316 (C-N), 1016 (C-N), 995, 750. ^{1}H NMR (500 MHz, $CDCl_3$) δ 7.28-7.32 (4H, m, ArH), 7.06-7.12 (4H, m, ArH), 6.97 (1H, tt, J 7.3, 1.1 Hz, ArH), 5.75 (1H, s, NH), 4.78 (6H, dt, J 47.0, 1.5 Hz, CH_2F). ^{13}C NMR (126 MHz, Acetone-d^6) δ 130.0, 128.7, 121.3, 118.5, 117.7, 84.1 (3C, dt, J 173.9, 5.4 Hz, CH_2F), 41.3 ($\underline{C}CH_2F$). ^{19}F{^{1}H} NMR (376 MHz, $CDCl_3$) δ -230.5 (3F, s, CH_2F). HRMS (EI) calculated for $C_{16}H_{16}F_3N$: 279.1229; found 279.1238.

(*R*)-4-(1,3-difluoro-2-(fluoromethyl)propan-2-yl)-N-(1-(naphthalen-1-yl)ethyl)aniline (35)

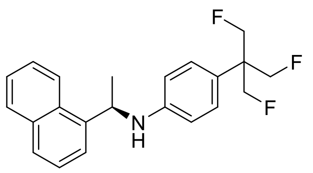

This compound was prepared according to General Procedure B. The compound was isolated as a yellow oil (38.0 mg, 62.5%). IR v_{max}/cm^{-1} 2968, 2361 (C-H), 1614, 1512, 1508, 1026 (C-F), 999, 799, 775. ^{1}H NMR (500 MHz, $CDCl_3$) δ 8.15 (1H, d, J 8.2 Hz, ArH), 7.90-7.93 (1H, m, ArH), 7.76 (1H, d, J 8.3 Hz, ArH), 7.64 (1H, d, J 7.3 Hz, ArH), 7.50-7.59 (2H, m, ArH), 7.42 (1H, dd, J 8.0, 7.3 Hz, ArH), 7.07-7.11 (2H, m, ArH), 6.46-6.52 (2H, m, ArH), 5.27 (1H, q, J 6.7 Hz, C<u>H</u>CH_3), 4.69 (6H, dt, J 47.1, 1.5 Hz, CH_2F), 1.66 (3H, d, 6.7 Hz, CH_3). ^{13}C NMR (126 MHz, $CDCl_3$) δ 146.8, 139.8, 134.3, 130.7, 129.3, 127.8, 127.7, 126.3, 126.0, 125.7, 124.2, 122.6, 122.4, 113.5, 83.5 (3C, dt, J 176.1, 6.0 Hz, <u>C</u>H_2F), 49.6 (N<u>C</u>CH3), 48.7 (<u>C</u>CH_2F), 23.7 (CH_3). $^{19}F\{^{1}H\}$ NMR (376 MHz, $CDCl_3$) δ -230.4 (3F, s, CH_2F). HRMS (ESI^+) calculated for $C_{22}H_{22}F_3NNa^+$: 380.1602; found 380.1597. $[\alpha]^{20}_{D}$ = +25.5° (c = 0.0167, $CHCl_3$).

2-(p-Tolyl)acetaldehyde (43)[4]

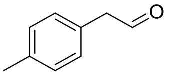

Dess-Martin Periodinane (8.56 g, 20.2 mmol) was added to a stirred solution of 2-(p-tolyl)ethan-1-ol (2.53 mL, 18.4 mmol) in DCM (184 mL) and the reaction was stirred for 3 hours. The reaction was quenched by addition of sat. aq. sodium thiosulfate and sat. aq. sodium hydrogen carbonate. The aqueous layer was extracted with DCM (3x). The combined organic layers were washed with $NaHCO_3$ and brine, dried over sodium sulfate, filtered and concentrated to give the crude product. The crude product was dissolved in 9:1 hexane/ethyl acetate solution and filtered. The filtrate was concentrated under reduced pressure to give the desired product as a colourless liquid (1.55 g, 62.8%). ^{1}H NMR (400 MHz, $CDCl_3$) δ 9.73 (1H, t, J 2.4 Hz, CHO), 7.18 (2H, d, J 7.9 Hz, ArH), 7.12 (2H, d, J 7.9 Hz, ArH), 3.65 (2H, d, J 2.4 Hz, CH_2), 2.35 (3H, s, CH_3).

2-(Hydroxymethyl)-2-(p-tolyl)propane-1,3-diol (44)

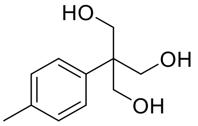

2-(p-Tolyl)acetaldehyde (1.72 g, 12.8 mmol) and calcium hydroxide (3.80 g, 51.3 mmol) were added to a suspension of paraformaldehyde (1.54 g, 51.3 mmol) in THF (19.2 mL). The reaction mixture was stirred at 60-65 °C for four days. After cooling to room temperature, the reaction mixture was filtered through celite and the celite was rinsed with DCM (50 mL). The filtrate was concentrated under reduced pressure to give the crude product. The crude product was purified by column chromatography over silica gel (1:1 hexane/ethyl acetate gradient to 100% ethyl acetate) to give the desired product as a white solid (1.47 g, 58.3%). IR v_{max}/cm^{-1} 3333 (O-H), 2920 (C-H), 2878 (C-H), 1516, 1115 (C-O), 1065 (C-O), 1013, 812. ^{1}H NMR (500 MHz, d^6-DMSO) δ 7.27-7.30 (2H, m, ArH), 7.04-7.08 (2H, m, ArH), 4.38 (6H, t, J 5.2 Hz, CH_2), 3.68 (3H, t, J 5.2 Hz, OH), 3.35 (3H, s, CH_3). ^{13}C NMR (126 MHz, d^6-DMSO) δ 139.9,

134.3, 128.2, 127.5, 63.5 (CH_2), 48.7 ($\underline{C}CH_2$), 20.6 (CH_3). HRMS (EI) calculated for $C_{11}H_{16}O_3$: 196.1094; found 196.1095.

2-(p-Tolyl)-2-((tosyloxy)methyl)propane-1,3-diyl bis(4-methylbenzenesulfonate) (45)

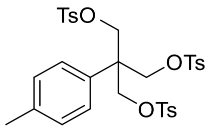

p-Toluenesulfonyl chloride (4.43 g, 23.2 mmol) was added slowly to a solution of 2-(hydroxymethyl)-2-(p-tolyl)propane-1,3-diol (1.29 g, 4.94 mmol) in pyridine (10.2 mL) at 0 °C and the reaction was warmed to room temperature and stirred for three days. On completion, the reaction mixture was concentrated under reduced pressure. The resulting residue was dissolved in DCM (100 mL) and the suspension was filtered. The filtrate was washed with water (100 mL). The organic phase was separated, and the aqueous phase was extracted with DCM (3 x 30 mL). The combined organic phases were washed with brine (50 mL), dried over magnesium sulfate, filtered and concentrated under vacuum to give the crude product. The crude product was suspended in ethanol and the suspension was stirred vigorously and heated to 92 °C. The suspension was filtered to give the desired product as a white solid (2.12 g, 65.1%). m.p. 136-138 °C. IR ν_{max}/cm^{-1} 2988 (C-H), 2901 (C-H), 2361, 1362 (S=O), 1173 (C-O), 1076 (C-O), 1067 (C-O), 970, 827, 802. ^1H NMR (500 MHz, $CDCl_3$) δ 7.60-7.64 (6H, m, ArH), 7.29-7.33 (6H, m, ArH), 6.95-6.98 (2H, m, ArH), 6.79-6.83 (2H, m, ArH), 4.16 (6H, s, CH_2O), 2.46 (9H, s, CH_3), 2.29 (3H, s, CH_3). ^{13}C NMR (126 MHz, $CDCl_3$) δ 145.4, 138.0, 131.9, 131.8, 130.1, 129.6, 128.1, 126.2, 69.1 (CH_2O), 45.9 ($\underline{C}CH_2$), 21.9 ($SO_2Ar\underline{C}H_3$), 21.1 ($Ar\underline{C}H_3$). HRMS (ESI^+) calculated for $C_{32}H_{34}O_9S_3Na^+$: 681.1263; found 681.1257.

1-(1,3-Difluoro-2-(fluoromethyl)propan-2-yl)-4-methylbenzene (46)

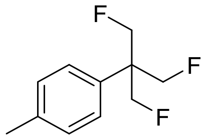

Cesium fluoride (1.02 g, 6.70 mmol) was added to a solution of 2-(p-tolyl)-2-((tosyloxy)methyl)propane-1,3-diyl bis(4-methylbenzenesulfonate) (245.2 mg, 0.372 mmol) in DMSO (8.15 mL) and the reaction was heated to 120 °C and stirred for 24 h. On completion, the reaction mixture was diluted with water and the aqueous layer was extracted with diethyl ether (3x). The combined organic layer was washed with water, dried over magnesium sulfate, filtered and concentrated under vacuum to give the crude product. The crude product was purified by column chromatography over silica gel (hexane/ethyl acetate, gradient from 0 to 10% ethyl acetate) to give the desired product as a yellow oil (53.0 mg, 70.5%). IR ν_{max}/cm^{-1} 2970 (C-H), 2924 (C-H), 2361, 1518, 1030 (C-F), 1003 (C-F), 814. ^1H NMR (500 MHz, $CDCl_3$) δ 7.30 (2H, d, J 8.1 Hz, ArH), 7.22 (2H, d, J 7.9 Hz, ArH), 4.79 (6H, dt, J 47.0, 1.6 Hz, CH_2F), 2.35 (3H, s, CH_3). ^{13}C NMR (126 MHz, $CDCl_3$) δ 138.0, 133.2, 129.8, 126.8, 83.3 (3C, dt, J 176.6, 5.8 Hz, CH_2F), 21.1 (CH_3). $^{19}F\{^1H\}$ NMR (376 MHz, $CDCl_3$) δ -230.8 (3F, s, CH_2F). HRMS (ESI) calculated for $C_{11}H_{13}F_3Na^+$: 225.0867; found 225.0862.

1-(Bromomethyl)-4-(1,3-difluoro-2-(fluoromethyl)propan-2-yl)benzene (47)

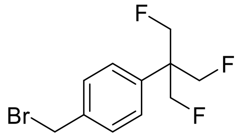

1-(1,3-difluoro-2-(fluoromethyl)propan-2-yl)-4-methylbenzene (38.8 mg, 0.192 mmol) was added to a solution of N-bromosuccinimide (61.5 mg, 0.0384 mmol) in trifluorotoluene (1.0 mL). The reaction was heated to reflux and stirred for 18 hours. On completion, the reaction was cooled to room temperature, hexane was added to the mixture and the mixture was filtered. The solvent was removed under reduced pressure to give the crude product. The crude product was purified by column chromatography over silica gel (hexane/ethyl acetate) to give the purified product as a yellow oil (28.7 mg, 53.2%). IR ν_{max}/cm^{-1} 2972 (C-H), 1024 (C-F), 1001 (C-F), 642 (C-Br), 624 (C-Br). ^{1}H NMR (400 MHz, $CDCl_3$) δ 7.38-7.45 (4H, m, ArH), 4.79 (6H, (dq, J 46.9, 1.6 Hz, CH_2F), 4.48 (2H, s, CH_2Br). ^{13}C NMR (126 MHz, $CDCl_3$) δ 137.7, 136.6, 129.6, 127.5, 83.1 (3C, dt, J 175.9, 6.7 Hz, CH_2F), 40.2, 32.7 (CH_2Br). $^{19}F\{^{1}H\}$ NMR (470 MHz, $CDCl_3$) δ -231.0 (3F, s, CH_2F). HRMS (EI) calculated for $C_{11}H_{12}Br^{79}F_3$: 280.0069; found 280.0060.

2-(Tert-butyl)-4,5-dichloropyridazin-3(2H)-one[5]

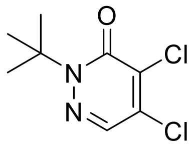

Tert-butylhydrazine hydrochloride (6.23 g, 50.0 mmol) was added to a solution of sodium hydroxide (1.90 g, 47.5 mmol) dissolved in a mixture of toluene (180 mL) and water (20 mL) at room temperature. Mucochloric acid (8.45 g, 50.0 mmol) was added in batches and stirred for 30 minutes. Acetic acid (2.72 mL, 47.5 mmol) was added dropwise and the reaction was heated for 45-50 ^{o}C and stirred for 18 hours. The solution was allowed to cool to room temperature and then diluted with water (80 mL). The organic layer was cooled to 0 ^{o}C and washed with 30% sodium hydroxide solution, concentrated hydrochloric acid solution and water. The organic layer was dried over magnesium sulfate, filtered and concentrated under vacuum to give the crude product as a red solid. The crude product was used without further purification (7.21 g, 68.1%). ^{1}H NMR (400 MHz, $CDCl_3$) δ 7.73 (1H, s, NCH), 1.64 (9H, s, CH_3).

2-(Tert-butyl)-4-chloro-5-mercapto-4,5-dihydropyridazin-3(2H)-one (48)[6]

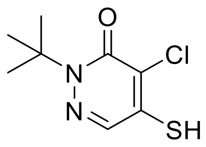

Sodium hydrosulfide hydrate (3.63 g, 64.8 mmol) was added to a solution of 2-(tert-butyl)-4,5-dichloropyridazin-3(2H)-one (7.54 g, 34.1 mmol) in ethanol (30 mL) in batches at 0 ^{o}C and the reaction mixture was stirred at room temperature for 2 hours. The mixture was poured

into ice water and acidified with concentrated hydrochloric acid (23.4 mL). The yellow solid was collected, washed with ice water, dried and used without further purification (5.43 g, 72.1%). ^{1}H NMR (400 MHz, $CDCl_3$) δ 7.57 (1H, s, NCH), 4.00 (1H, s, SH), 1.63 (9H, s, CH_3).

2-(Tert-butyl)-4-chloro-5-((4-(1,3-difluoro-2-(fluoromethyl)propan-2-yl)benzyl)thio)pyridazin-3(2H)-one (49)

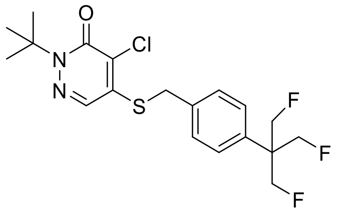

1-(Bromomethyl)-4-(1,3-difluoro-2-(fluoromethyl)propan-2-yl)benzene (17.4 mg, 0.0619 mmol) and sodium carbonate (9.84 mg, 0.0929 mmol) were added to a solution of 2-(tert-butyl)-4-chloro-5-mercaptopyridazin-3(2H)-one (13.5 mg, 0.0619 mmol) in DMF (0.25 mL) and the reaction mixture was stirred for 18 hours at room temperature. The reaction mixture was extracted with ethyl acetate (3x). The combined organic layers were washed with water, dried over magnesium sulfate, filtered and concentrated under vacuum to give the crude product. The crude product was purified by column chromatography over silica gel (hexane/ethyl acetate, gradient 0 to 10% ethyl acetate) to give the purified product as an orange solid (13.9 mg, 53.3%). m.p. 106-108 °C. IR v_{max}/cm^{-1} 2984 (C-H), 2361, 1653 (C=O), 1558 (C=C), 1506. ^{1}H NMR (400 MHz, $CDCl_3$) δ 7.60 (1H, s, CHN), 7.41-7.47 (4H, m, ArH), 4.79 (6H, dt, J 46.8, 1.5 Hz, CH_2F), 4.26 (2H, s, SCH_2), 1.63 (9H, s, CH_3). ^{13}C NMR (101 MHz, $CDCl_3$) δ 131.9, 131.1, 129.4, 127.9, 83.1 (3C, dt, J 176.4, 5.2 Hz, CH_2F), 35.4, 29.9, 27.8. ^{19}F{^{1}H} NMR (377 MHz, $CDCl_3$) δ -231.0 (3F, s, CH_2F). HRMS (ESI) calculated for $C_{19}H_{22}ON_2Cl^{35}F_3SNa^+$: 441.0986; found 441.0991.

Synthesis of reference substrate 41 for the *Cunninghamella elegans* biotransformation

Synthesis route to reference metabolite 41

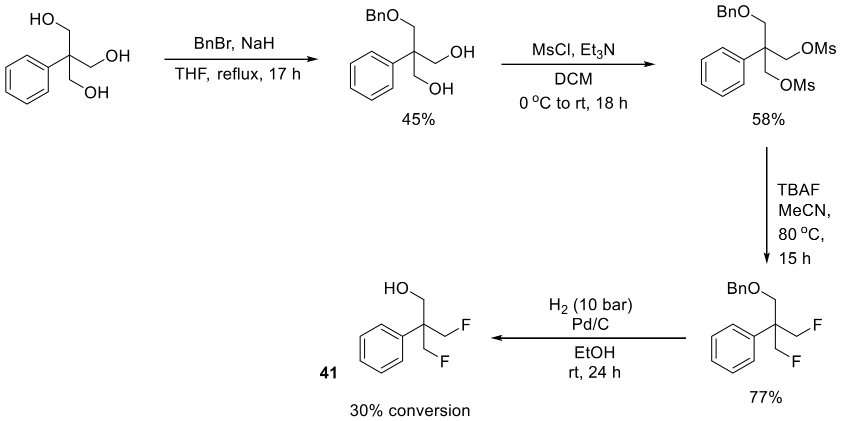

2-((Benzyloxy)methyl)-2-phenylpropane-1,3-diol

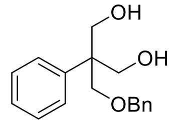

A solution of 2-hydroxymethyl-2-phenylpropane (100 mg, 0.547 mmol) in THF (0.2 mL) was added dropwise to a solution of sodium hydride (60% dispersion in mineral oil, 22.0 mg, 0.549 mmol) in THF (0.6 mL). The reaction mixture was refluxed for 1 hour, then benzyl bromide (71.8 µL, 0.604 mmol) was added and refluxing was continued for 16 hours. The reaction mixture was cooled to room temperature and water (0.1 mL) was added, followed by the removal of THF under reduced pressure. The residue was extracted with diethyl ether (3 x 2 mL), washed with water, brine and dried over magnesium sulfate. The solvent was removed under reduced pressure to give the crude product. The crude product was purified by column chromatography over silica gel (hexane/ethyl acetate, gradient 0 to 100% ethyl acetate) to give the desired product as a colourless oil (122.2 mg, 44.9%). ^{1}H NMR (400 MHz, d^{6}-Acetone) δ 7.48-7.53 (2H, m, ArH), 7.24-7.35 (7H, m, ArH), 7.16-7.21 (1H, m, ArH), 4.53 (2H, s, $C\underline{H_2}Ph$), 3.99 (2H, qd, J 10.9, 3.7 Hz, $C\underline{H_2}OH$), 3.92 (2H, s, $C\underline{H_2}OCH_2Ph$), 3.71-3.79 (2H, m, $C\underline{H_2}OCH_2Ph$). ^{13}C NMR (101 MHz, d^{6}-Acetone) δ 143.0, 139.7, 129.0, 128.7, 128.4, 128.2, 128.1, 126.8, 73.9 ($\underline{C}H_2Ph$), 73.5 ($\underline{C}H_2OCH_2Ph$), 66.2 ($C\underline{H_2}OH$), 49.8 ($\underline{C}CH_2O$). HRMS (ESI) calculated for $C_{17}H_{20}O_3Na^+$: 295.1310; found 295.1305.

2-((Benzyloxy)methyl)-2-phenylpropane-1,3-diyl dimethanesulfonate

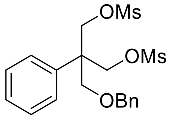

Triethylamine (84.5 µL, 0.606 mmol) was added to a solution of 1-(1,3-difluoro-2-(fluoromethyl)propan-2-yl)-4-methylbenzene (66.1 mg, 0.243 mmol) in dichloromethane (1.33 mL) at 0 °C. Methanesulfonyl chloride (47.0 µL, 0.606 mmol) was added and the reaction was warmed to room temperature. On completion, the reaction was quenched by the addition of saturated ammonium chloride solution (1.33 mL) and the aqueous layer was extracted with dichloromethane (3 x 7.2 mL). The combined organic layers were washed with brine (7.2 mL), dried over magnesium sulfate, filtered and concentrated under vacuum to give the crude product. The crude product was purified by column chromatography over silica gel (hexane/ethyl acetate, gradient 0 to 100% ethyl acetate) to give the purified product as a yellow oil (60.4 mg, 58.0%). IR ν_{max}/cm^{-1} 2988 (C-H), 2901 (C-H), 2361, 1352 (S=O), 1171 (S=O), 951, 822, 698. ^{1}H NMR (400 MHz, $CDCl_3$) δ 7.29-7.41 (8H, m, ArH), 7.23-7.28 (2H, m, ArH), 4.58 (4H, dd, J 11.1, 10.0 Hz, OCH_2S), 4.53 (2H, s, $O\underline{C}H_2Ar$), 3.82 (2H, s, $\underline{C}H_2OCH_2Ar$), 2.85 (6H, s, SCH_3). ^{13}C NMR (126 MHz, $CDCl_3$) δ 137.4, 137.3, 129.0, 128.7, 128.1, 127.9, 126.7, 73.8 ($O\underline{C}H_2Ar$), 70.4 ($\underline{C}H_2OCH_2Ar$), 69.9 ($\underline{C}H_2OS$), 47.0 ($\underline{C}CH_2O$), 37.3 (SCH_3). HRMS (ESI) calculated for $C_{19}H_{24}O_7S_2Na^+$: 451.0861; found 451.0856.

(1-(Benzyloxy)-3-fluoro-2-(fluoromethyl)propan-2-yl)benzene

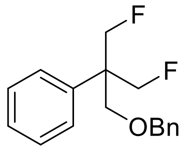

Tert-butylammonium fluoride (1.0M in THF, 0.311 mL, 0.311 mmol) was added to a solution of 2-((benzyloxy)methyl)-2-phenylpropane-1,3-diyl dimethanesulfonate (33.4 mg, 0.0778 mmol) in acetonitrile (0.519 mL) and the reaction was stirred under reflux for 15 hours. The reaction was then cooled to room temperature and water (4.32 mL) was added. The mixture was extracted with ethyl acetate (3 x 3.24 mL). The combined organic layers were dried over magnesium sulfate, filtered and concentrated under vacuum to give the crude product. The crude product was purified by column chromatography over silica gel (hexane/ethyl acetate, gradient 0 to 100% ethyl acetate) to give the purified product as a colourless oil (16.6 mg, 77.3%). ^{1}H NMR (400 MHz, $CDCl_3$) δ 7.23-7.44 (10H, m, ArH), 4.87 (2H, qd, J 9.3, 1.5 Hz, CH_2F), 4.75 (2H, qd, J 9.3, 1.5 Hz, CH_2F), 4.52 (2H, s, O<u>C</u>H_2Ar), 3.80 (2H, t, J 1.6 Hz, C<u>C</u>H_2O). ^{13}C NMR (126 MHz, $CDCl_3$) δ 138.0, 128.7, 128.5, 127.9, 127.7, 127.6, 127.1, 114.7, 84.3 (2C, dd, J 175.3, 5.4 Hz, <u>C</u>H_2F), 73.7 (O<u>C</u>H_2Ar), 72.1 (d, J 8.4 Hz, <u>C</u>CH_2F), 70.6 (t, J 5.9 Hz, C<u>C</u>H_2O). ^{19}F NMR (470 MHz, $CDCl_3$) δ -230.2 (2F, t, J 47.1 Hz, CH_2F). HRMS (ESI) calculated for $C_{17}H_{18}F_2ONa^+$: 299.1218; found 299.1212.

3-Fluoro-2-(fluoromethyl)-2-phenylpropan-1-ol (41)

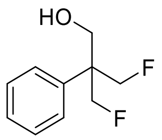

(1-(Benzyloxy)-3-fluoro-2-(fluoromethyl)propan-2-yl)benzene (8.0 mg, 0.0203 mmol) was dissolved in ethanol (0.25 mL) and 10% palladium on carbon (2.0 mg) was added. The reaction was placed in an autoclave which was filled with hydrogen gas (50 bar). On completion, the reaction mixture was filtered through celite and the solvent removed under reduced pressure. ^{1}H NMR (400 MHz, $CDCl_3$) δ 7.22-7.44 (5H, m, ArH), 4.85 (4H, dt, J 47.1, 1.3 Hz, CH_2F. ^{19}F NMR (376 MHz, $CDCl_3$) δ -230.7 (2F, t, J 47.1 Hz, CH_2F).

Synthesis of (monofluoromethyl)-*tert* butyl benzene (37)

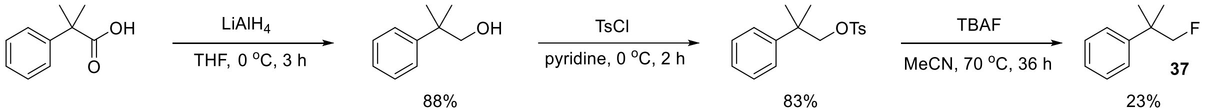

2-Methyl-2-phenylpropan-1-ol

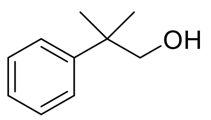

2-Methyl-2-phenylpropionic acid (897 mg, 5.46 mmol) was dissolved in THF (30 mL) under inert atmosphere. The reaction mixture was cooled to 0 °C using an ice-bath and $LiAlH_4$ in THF (2 M, 5.46 mL) was added dropwise. Upon addition, the reaction was stirred at ambient temperature for 20 min, then heated to 50 °C for 3 h. The reaction mixture was cooled to 0 °C and quenched with methanol (1 mL) and HCl (40 mL). The product was extracted with ethyl acetate (3 × 30 mL) dried over $MgSO_4$, and solvent removed under reduced pressure. The product was obtained as a white solid (723 mg, 88%). ^{1}H NMR (500 MHz, $CDCl_3$) δ 7.43-7.34 (4H, m, ArH), 7.24 (1H, tt, J = 7.1, 1.3 Hz, ArH), 3.59 (2H, s, $C\underline{H_2}OH$), 1.73 (1H, s, $CH_2O\underline{H}$), 1.35 (6H, s, $C\underline{H_3}$). ^{13}C NMR (126 MHz, $CDCl_3$) δ 146.5, 128.4, 126.3, 126.2, 73.1 (s, $\underline{C}H_2OH$), 40.1 (s, $\underline{C}HCH_2OH$), 25.4 (CH_3). HRMS (ESI) calculated for $C_{10}H_{14}ONa^+$, 173.0943; found, 173.0937.

2-Methyl-2-phenylpropyl 4-methylbenzenesulfonate

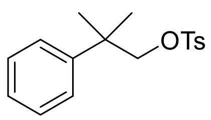

2-Methyl-2-phenylpropan-1-ol (0.321 g, 2.14 mmol) prepared above was dissolved in pyridine (6 mL) and cooled in an ice-bath to 0 °C. p-Toluenesulfonyl chloride (1.48 g, 7.75 mmol) was added portionwise with continuous stirring. The mixture was stirred for 2 h before being placed in the fridge overnight. The product was crashed out in water (100 mL) and filtered through celite with diethyl ether (50 mL) and ethyl acetate (60 mL). The solvent was removed under reduced pressure and the sample was dry loaded on silica. Column chromatography was carried out on the sample, eluting with ethyl acetate and hexane, gradient from 0% to 20% ethyl acetate to remove excess starting material. The solvent was removed under reduced pressure and the product obtained (0.542 g, 83%). ^{1}H NMR (500 MHz, $CDCl_3$) δ 7.64 (2H, d, J = 8.3 Hz, ArH), 7.29-7.18 (7H, m, ArH), 3.97 (2H, s, CH_2OTs), 2.44 (3H, s, $ArCH_3$), 1.33 (6H, s, CH_3). ^{13}C NMR (126 MHz, $CDCl_3$) δ 144.8, 144.7, 132.9, 129.9, 128.5, 128.0, 126.7, 126.0, 78.6 ($\underline{C}H_2OS$), 38.6 ($\underline{C}CH_2OS$), 25.4 ($C(\underline{C}H_3)_2$), 21.8 ($ArCH_3$). HRMS (ESI) calculated for $C_{17}H_{20}O_3SNa^+$, 327.1032; found, 327.1020.

(1-Fluoro-2-methylpropan-2-yl)benzene (37)

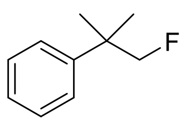

A sample of concentrated TBAF was prepared by removal under reduced pressure of the solvent from in THF (1 M, 8.9 mL). This TBAF was dissolved in MeCN (5 mL), and the tosylate prepared above (150 mg, 0.49 mmol) was added and heated to 70 °C in a sealed vial for 36 h. The solvent was removed under reduced pressure and extracted using diethyl ether (4 × 40 mL) from water (100 mL). The product was backwashed with water (30 mL) to remove any

excess MeCN. The organic layer was dried over $MgSO_4$, and the solvent removed under reduced pressure. The product was dry loaded on silica using pentane and purified using column chromatography. The column was carried out eluting with pentane, the solvent then removed under reduced pressure. The pure product was then obtained as a yellow oil (17.0 mg, 23%). ^{1}H NMR (500 MHz, $CDCl_3$) δ 7.42-7.33 (4H, m, ArH), 7.25 (1H, t, J 6.4 Hz, ArH), 4.38 (2H, d, J 47.9 Hz, CH_2F), 1.39 (6H, d, J 1.8 Hz, CH_3). ^{13}C NMR (126 MHz, $CDCl_3$) δ 145.5 (d, J 3.6 Hz, $\underline{C}CCH_2$), 128.5, 126.6, 126.2, 91.6 (d, J 177.2 Hz, $\underline{C}H_2F$), 39.4 (d, J 17.7 Hz, $\underline{C}CH_2F$), 24.9 (d, J 4.7 Hz, $\underline{C}H_3$). ^{19}F NMR (470 MHz, $CDCl_3$) δ -219.0 (1F, t, J 47.9 Hz, CH_2F). HRMS (EI) calculated for $C_{10}H_{13}F$, 152.0996; found, 152.0999.

Synthesis of (difluoromethyl)-*tert* butyl benzene (36)

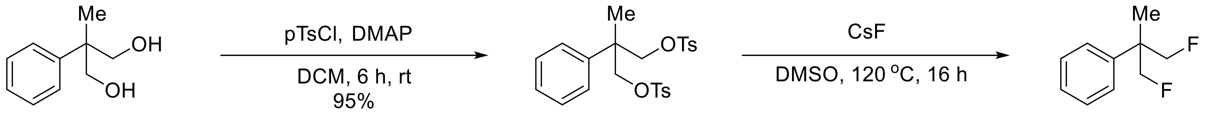

36

Synthesis of ditosylate[7]

A solution of p-toluenesulfonyl chloride (1.43 g, 7.5 mmol) in dichloromethane (6 ml) was added to a solution of 2-methyl-2-phenylpropane-1,3-diol (0.5 g, 3 mmol) and dimethylaminopyridine (1.1 g, 9.3 mmol) in dichloromethane (4 ml) at 0 °C over 30 min and then the reaction was left to stir for 6 h at ambient temperature. The reaction was then diluted with ethyl acetate (20 ml) and washed with water and brine, and the organics were dried over sodium sulfate, filtered and the solvent removed under vacuum. The crude product was purified by column chromatography over silica gel to give the purified product as a white solid (1.39 g, 2.85 mmol) in 95% yield. ^{1}H NMR (500 MHz, $CDCl_3$) δ 7.66 (4H, d, J 8.0 Hz, ArH), 7.31 (4H, d, J 7.9 Hz, ArH), 7.21-7.25 (3H, m, ArH), 7.06-7.13 (2H, m, ArH), 4.10 (4H, s, CH_2O), 2.45 (6H, s, $ArCH_3$), 1.31 (3H, s, CH_3). Data is in correspondence with the literature.[6]

Synthesis of 2-methyl-2-phenylpropane-1,3-difluoride (36)

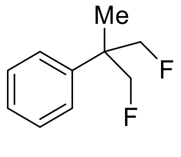

A solution of the ditosylate prepared above in DMSO was treated with CsF at 120 °C and the reaction was stirred for 16 h. On completion, water was added to the reaction and the aqueous phase was extracted with diethyl ether (3x). The combined organic layers were washed with water (3x), dried over magnesium sulfate, filtered and concentrated under reduced pressure to give the product as a yellow oil. ^{1}H NMR (500 MHz, $CDCl_3$) δ 7.73-7.29 (5H, m, ArH), 4.66 (2H, ddd, J 47.4, 9.1, 1.1 Hz, CH_2F), 4.55 (2H, ddd, J 47.4, 9.1, 1.6 Hz, CH_2F), 1.42 (3H, t, J 2.1 Hz, CH_3). ^{13}C NMR (126 MHz, $CDCl_3$) δ 140.6, 128.7, 127.3, 126.5, 86.2 (dd, J 176.9, 5.3 Hz, CH_2F), 43.9 (t, J 17.0 Hz, $\underline{C}CH_2F$), 18.7 (t, J 5.3 Hz, CH_3). ^{19}F NMR (470 MHz, $CDCl_3$) δ -224.2 (2F, t, J 47.3 Hz, CH_2F).

LogP Determinations

Compounds **18, 36, 37** and **38** were subject to reverse phase HPLC analysis to determine their logP values. The reference compounds used in this experiment were phenol and toluene. The column used was a Phenomenex Luna C18 100A (250 × 4.60 mm) 5μ, and was eluted with 60:40 MeCN:Water supplemented with 0.05% TFA at a flow rate of 1 mL/min. The samples were injected onto the column (10 μL of 0.5 mg/mL solution in MeCN) and the retention times used to determine Log P values as previously described.[7]

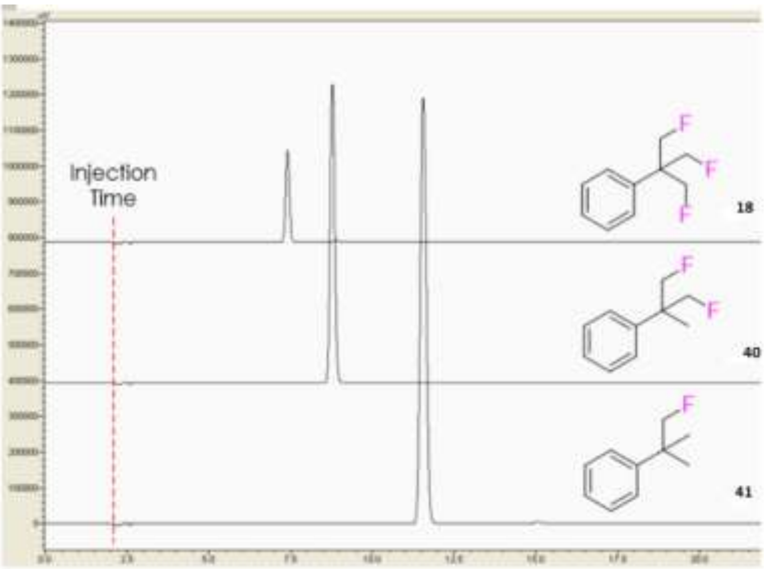

Figure S1 - A comparison of the reverse phase HPLC retention times for the three selectively fluorinated *tert*-butyl benzenes **18**, **36** and **37**.

Biotransformation with *Cunninghamella elegans* DSM1908[9]

Cunninghamella elegans DSM1908 was cultured using Sabouraud dextrose media (in broth, SDB, or in agar, SDA) as described in. The fungus was streaked on SDA plates and incubated at 28 °C for 5 days to grow the mycelia. The grown mycelium (with agar) was then blended with autoclaved water (100 mL) to prepare the fungal inoculum. To grow fungal cultures, autoclaved SDB (45 mL) was inoculated with the fungal suspension (5 mL), and the flasks were incubated at 28 °C, 150 rpm for 72 hours (3 days). The grown fungi were then used for the biotransformation of TBB (5 mg) by further incubation at 28 °C, 150 rpm for 72 hours. The aqueous supernatant was separated and extracted twice with 100 mL of ethyl acetate. The organic solvent was then removed under reduced pressure and the residue redissolved in 1 mL of ethyl acetate for ^{19}F NMR and GC-MS analysis. The ^{19}F NMR spectra were recorded on a Varian 400 MHz spectrometer, and the samples were prepared in 800 µL of methanol d4 by first evaporating ethyl acetate under a flow of N_2 gas. The biotransformation products were analysed using an Agilent 7890B N gas chromatograph equipped with a 5977A mass-selective detector and operated in the scanning mode. Samples (1 µL) were injected (in splitless setting) into an HP-5MS capillary column (30 m × 0.25 mm × 0.25 µm). The GC oven was set at an initial column temperature of 90 °C for 5 minutes and then raised to 300 °C at a rate of 10 °C/min.

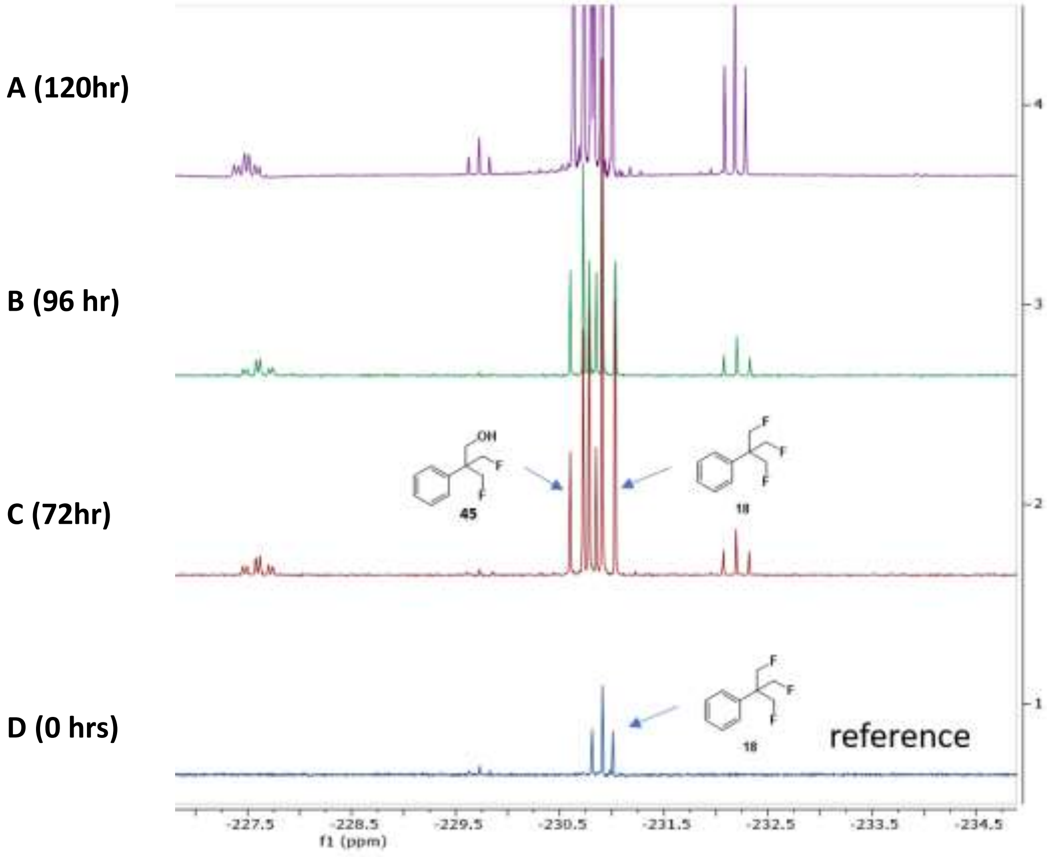

Figure S2. Time course ^{19}F-NMR (with proton coupling) spectra of supernatant extracts after incubating compound **18** with *C. ellegans*. Alcohol **41** was identified as the major metabolite by comparison to a synthetic sample of **41**. The identification of **41** was also supported by GC-MS (see Fig S3)

A

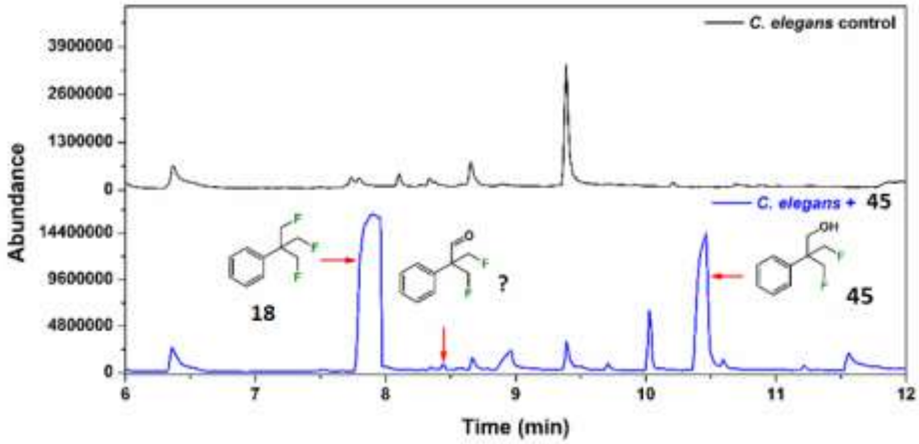

C. elegans control
C. elegans + 45
Abundance
18
?
45
Time (min)

B

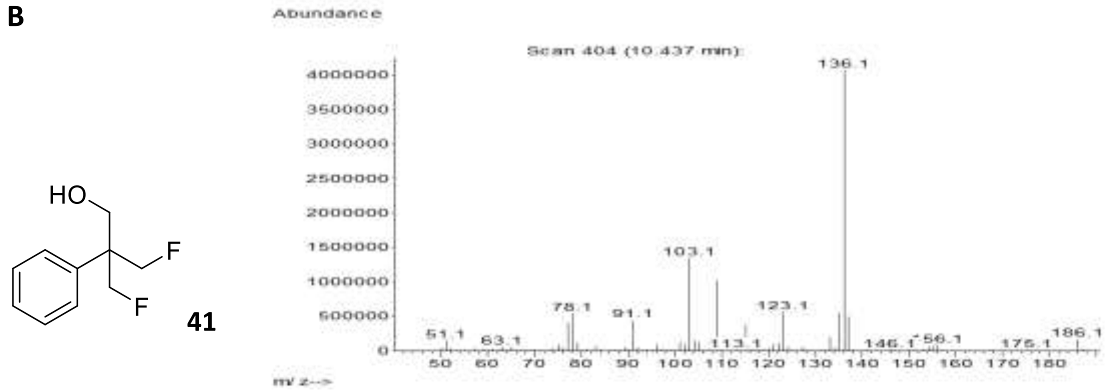

Abundance
Scan 404 (10.437 min):
136.1
103.1
123.1
78.1
91.1
186.1
HO
F
F
41
m/z-->

C

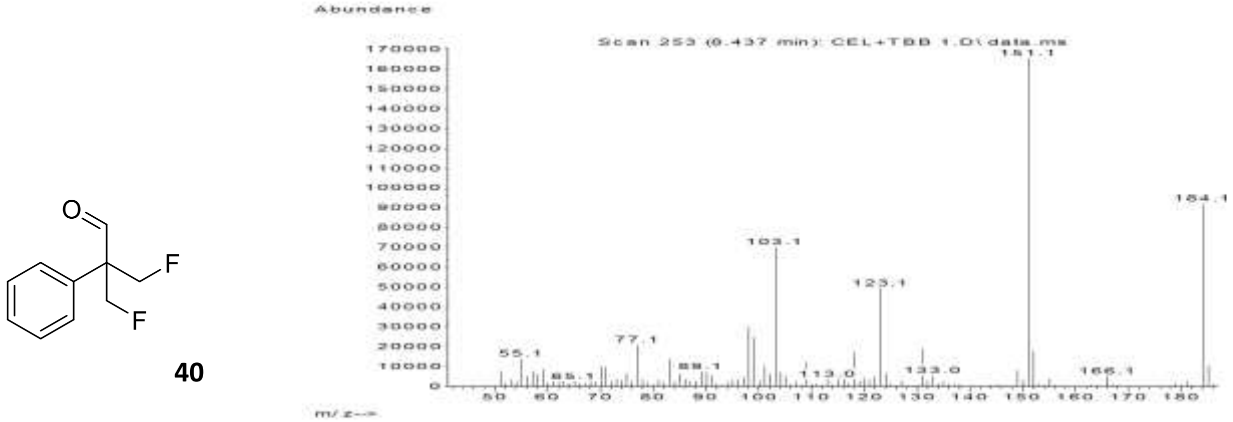

Abundance
151.1
184.1
103.1
123.1
77.1
55.1
O
F
F
40
m/z-->

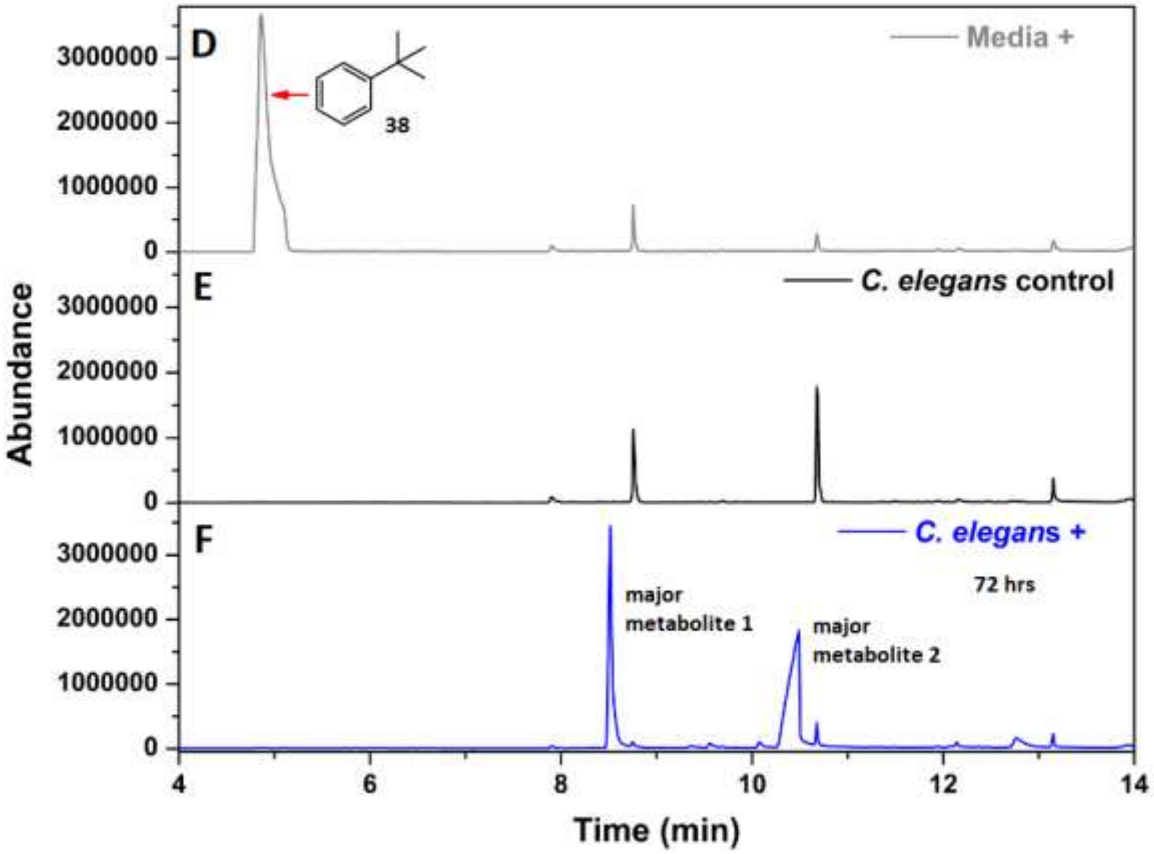

Figure S3: A Shows the GC-profile of the supernatant extract from *C. elegans* cultures incubated with **18** (lower GCMS trace) and without **18** (upper GC_MS trace) after 72 hrs. Substrate **18** was identified at 7.9 min and the major metabolite **41** at 10.4 min. **B** shows the Mass spectrum (GC-MS) of the major metabolite recorded at 10.4 mins, consistent with **41** (amu 186; 136 (-CH_2OH + F); 103 (136 – 33(CH_2F)). **C** shows the mass spectrum (GC-MS) identified after ~8.46 mins, consistent with **40** (amu 184; 153 (amu– 33(CH_2F)). **D** GC-trace of media plus *tert* butylbenzene **38** (control time zero). **E** GC-trace of *C. elegans* after 72 hrs (control). **F** GC-trace recorded after 72hrs incubation of **38** with *C. elegans*, showing complete conversion to two major metabolites.

References

1. Viguier, R.; Serratrice, G.; Dupraz, A.; Dupuy, C. *Eur. J. Inorg. Chem*., **2001**, *2001*,1789–1795.
2. Wu, R.; Ga, K., *Org. Biomol. Chem.,* **2021**, *19*, 4032-4036
3. Petersen, F., Lautenschläger, I., Schlimm, A., Flöser, B. M., Jacob, H., Amirbeigiarab, R., Rusch, T. R., Strunskus, T., Magnussen, O., Tuczek, F. *Dalton Trans.*, **2021**, *50*, 3, 1042-1052.
4. Wang, Z.; Xue, F.; Hayashi, T. *Angew. Chemie. Int. Ed.,* **2019**, *58*, 11054 – 11057.
5. Qin, J.; , Zhu, M.; Zhu, H.; Zhang, L.; Fu, Y.; Liu, J.; Wang, Z.; OuYang, G. *Phosphorus Sulfur Silicon Relat. Elem.,* **2020**, *195*, 592–599.
6. Liu, S.; Jin, S.; Wang, H.; Qi, Z.; Hu,X.; Qian, B.; Huang, H. *Tetrahedron Lett.,* **2021**, *68*, 15295.

7. M. Tudge, H. Mashima, C. Savarin, G. Humphrey, I. Davies, *Tetrahedron Letts.,* **2008**, *49*, 1041 – 1044.
8. Rodil, A.; Bosisio, S.; Ayoup, M. S.; Quinn, L.; Cordes, D. B.; Slawin, A. M. Z.; Murphy, C. D.; Michel, J.; O'Hagan, D. *Chem. Sci.,* **2018**, *9*, 3023 - 3028.
9. Khan, M.F.; Murphy, C. D. *Enz. Microb. Tech.,* **2022**, *161*, 110102.

Determination of solubility in water

In order to determine solubility in water an excess (~ 25.0 mg) of compound **23** and its *tert*-butyl non-fluorinated analogue were each dissolved in 1.5 mL of deionised water.

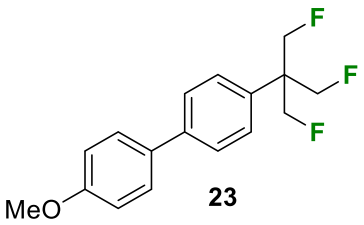

23

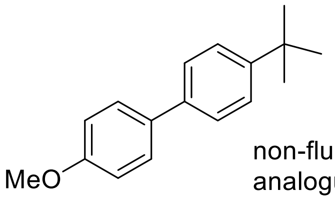

non-fluorinated analogue

The samples were boiled for 1 h, then cooled to room temperature. An exact aliquot (1.0 mL) of each was removed using a graduated syringe and filtered through cotton wool into a pre-weighed vial to remove any particulate solid. These samples were then freeze-dried. The resulting residues were weighed to yield the solubility values per ml for **23** (11mg/ml) and its *tert*-butyl non-fluorinated analogue (6 mgs/ml).

Crystallographic Data for compound 29

Colourless prismatic X-ray quality crystals of compound **29** were grown by diffusion of hexane into an ethyl acetate solution of the compound. X-ray diffraction data were collected at 173 K using a Rigaku MM-007HF High Brilliance RA generator/confocal optics with XtaLAB P200 diffractometer [Cu Kα radiation (λ = 1.54187 Å)]. Intensity data were collected using ω steps accumulating area detector images spanning at least a hemisphere of reciprocal space. Data were collected using CrystalClear[1] and processed (including correction for Lorentz, polarization and absorption) using CrysAlisPro.[2] The structure was solved by direct methods (SIR2011[3]) and refined by full-matrix least-squares against F^2 (SHELXL-2018/3[4]). Non-hydrogen atoms were refined anisotropically, and hydrogen atoms were refined using a riding model. All calculations were performed using the Olex2[5] interface. Selected crystallographic data are presented in Table S1, and a thermal ellipsoid plot is presented in Figure S4. CCDC 2270745 contains the supplementary crystallographic data for this paper. These data can be obtained free of charge from The Cambridge Crystallographic Data Centre via www.ccdc.cam.ac.uk/structures.

Table S1. Selected crystallographic data.

	**29**
formula	$C_{17}H_{17}F_3O$
fw	294.30
crystal description	Colourless prism
crystal size [mm^3]	0.06×0.04×0.02
space group	$P2_1/n$
a [Å]	8.92174(8)
b [Å]	9.48645(8)
c [Å]	16.96645(15)
β [°]	95.9870(9)
vol [Å]^3	1428.14(2)
Z	4
ρ (calc) [g/cm^3]	1.369
μ [mm^{-1}]	0.935
F(000)	616
reflections collected	16716
independent reflections (R_{int})	2929 (0.0151)
parameters, restraints	191, 0
GoF on F^2	1.056
R_1 [$I > 2\sigma(I)$]	0.0337
wR_2 (all data)	0.0930
largest diff. peak/hole [e/Å^3]	0.223, -0.196

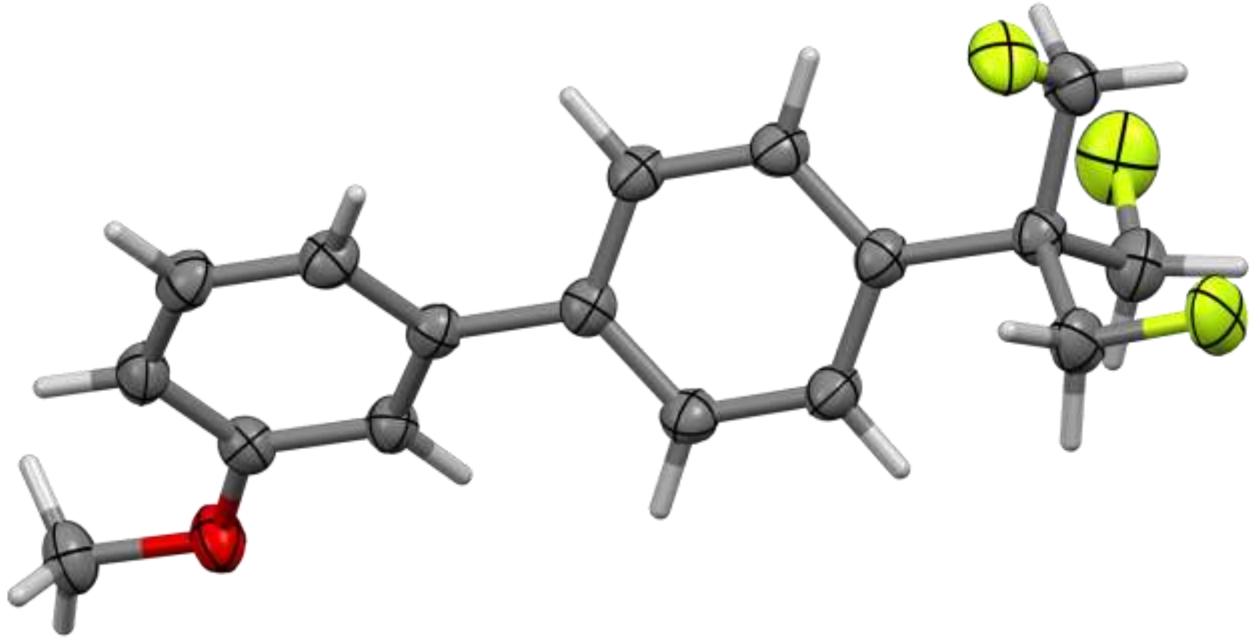

Figure S4: Thermal ellipsoid plot of the structure of **29**. Ellipsoids are drawn at the 50 % probability level

References for Crystallography

1. *CrystalClear-SM Expert* v2.1. Rigaku Americas, *The Woodlands, Texas, USA*, and Rigaku Corporation, *Tokyo, Japan*, 2015.
2. *CrysAlisPro* v1.171.41.93a. Rigaku Oxford Diffraction, Rigaku Corporation, *Oxford, U.K.*, 2020.
3. Burla, M. C.; Caliandro, R.; Camalli, M.; Carrozzini, B.; Cascarano, G. L.; Giacovazzo, C.; Mallamo, M.; Mazzone, A.; Polidori, G.; Spagna, R. SIR2011: a new package for crystal structure determination and refinement. *J. Appl. Crystallogr.* **2012**, *45*, 357-361.
4. Sheldrick, G. M. Crystal structure refinement with SHELXL. *Acta Crystallogr., Sect. C: Struct. Chem.* **2015**, *71*, 3-8.
5. Dolomanov, O. V.; Bourhis, L. J.; Gildea, R. J.; Howard, J. A. K.; Puschmann, H. OLEX2: a complete structure solution, refinement and analysis program. *J. Appl. Crystallogr*. **2009**, *42*, 339-341.

Computational details for conformational analysis of 18

The accessible conformers of compounds **18**, **36**, **37** and **38** were explored using the iterative workflow approach with static metadynamics simulations (--v4 keyword) as implemented in CREST software[1] at the semiempirical GFN2-xTB level of theory.[2] The resulting conformers were fully optimized with the hybrid Minnesota family functional M06-2X[3] and Alrichs' triple ζ def2-TZVP basis set[4] in Gaussian 16 rev C.01 program.[5] Frequency calculations confirmed all geometries as true energy minima, showing no imaginary frequencies. Molecular dipole moments were calculated for the global minima of **18**, **36**, **37** and **38** at the M06-2X/def2-TZVP level. The global minimum of **18** was used to explore the rotational energy profile (relaxed PES) around one C-CH_2F bond at the M06-2X/def2-TZVP theoretical level. Thermal corrections for each stationary point obtained from frequency calculations at standard temperature and pressure within the harmonic oscillator and rigid rotor models at the M06-2X/def2-TZVP theoretical level. No imaginary frequency was found for minima geometries, and a single imaginary frequency, corresponding to the C-CH_2F bond rotation, was found for the saddle points. NBO analysis (using the NBO 7.0 program[6] as implemented in Gaussian 16) was carried out at the M06-2X/def2-TZVP level of theory over optimised minima geometries, and NCI analysis[7] calculated over the electron densities obtained at the same theoretical level.

References for Computation

1. (a**)** Pracht, P.; F. Bohle, F.; Grimme, S. Automated Exploration of the Low-Energy Chemical Space with Fast Quantum Chemical Methods. *Phys. Chem. Chem. Phys*. **2020**, *22*, 7169-7192. (b) Grimme, S. Exploration of Chemical Compound, Conformer, and Reaction Space with Meta-Dynamics Simulations Based on Tight-Binding Quantum Chemical Calculations. *J. Chem. Theory. Comput.,* **2019**, *15*, 2847-2862.

2. Bannwarth C.; Ehlert, S.; Grimme, S. GFN2-xTB-An Accurate and Broadly Parametrized Self-Consistent Tight-Binding Quantum Chemical Method with Multipole Electrostatics and Density-Dependent Dispersion Contributions. *J. Chem. Theory Comput.,* **2019**, *15*, 1652–1671.

3. Zhao, Y.; D. Truhlar, D. G., The Mo6 Suite of Density Functionals for Main Group Thermochemistry, Thermochemical Kinetics, Noncovalent Interactions, Excited States, and Transition Elements: Two New Functionals and Systematic Testing of Four Mo6-class Functionals and 12 Other Functionals. *Theor. Chem. Acc.,* **2008**, *120*, 215–241.

4. Weigend, F.; Ahlrichs, R., Balanced Basis Sets of Split Valence, Triple Zeta Valence and Quadruple Zeta Valence Quality for H to Rn: Design and Assessment of Accuracy. *Phys. Chem. Chem. Phys*., **2005**, *7*, 3297-3305.

5. Gaussian 16 Revision C.01; Frisch, M. J.; Trucks, G. W.; Schlegel, H. B.; Scuseria, G. E.; Robb, M. A.; Cheeseman, J. R.; Scalmani, G.; Barone, V.; Petersson, G. A.; Nakatsuji, H.; Li, X.; Caricato, M.; Marenich, A. V; Bloino, J.; Janesko, B. G.; Gomperts, R.; Mennucci, B.; Hratchian, H. P.; Ortiz, J. V; Izmaylov, A. F.; Sonnenberg, J. L.; Williams-Young, D.; Ding, F.; Lipparini, F.; Egidi, F.; Goings, J.; Peng, B.; Petrone, A.; Henderson, T.; Ranasinghe, D.; Zakrzewski, V. G.; Gao, J.; Rega, N.; Zheng, G.; Liang, W.; Hada, M.; Ehara, M.; Toyota, K.;

Fukuda, R.; Hasegawa, J.; Ishida, M.; Nakajima, T.; Honda, Y.; Kitao, O.; Nakai, H.; Vreven, T.; Throssell, K.; Montgomery Jr., J. A.; Peralta, J. E.; Ogliaro, F.; Bearpark, M. J.; Heyd, J. J.; Brothers, E. N.; Kudin, K. N.; Staroverov, V. N.; Keith, T. A.; Kobayashi, R.; Normand, J.; Raghavachari, K.; Rendell, A. P.; Burant, J. C.; Iyengar, S. S.; Tomasi, J.; Cossi, M.; Millam, J. M.; Klene, M.; Adamo, C.; Cammi, R.; Ochterski, J. W.; Martin, R. L.; Morokuma, K.; Farkas, O.; Foresman, J. B.; Fox, D. J.; Inc., G. Gaussian 16 Revision C.01. *Wallingford CT*. Gaussian Inc., Walingford CT 2016.

6. Glendening, E. D.; Landis, C. R; Weinhold, F. NBO 7.0: New Vistas in Localized and Delocalized Chemical Bonding Theory. *J. Comput. Chem*., **2019**, *40*, 2234-2241. (b) Reed, A. E.; Weinstock, R. B; Weinhold, F., Natural Population Analysis. *J. Chem. Phys.,* **1985**, *83*, 735-746.

7. Johnson, E. R.; Keinan, S.; Mori-Sánchez, S.; Contreras-García, J.; Cohen, A. J.; Yang, W.T., Revealing Noncovalent Interactions. *J. Am. Chem. Soc.,* **2010**, *132*, 6498–6506.

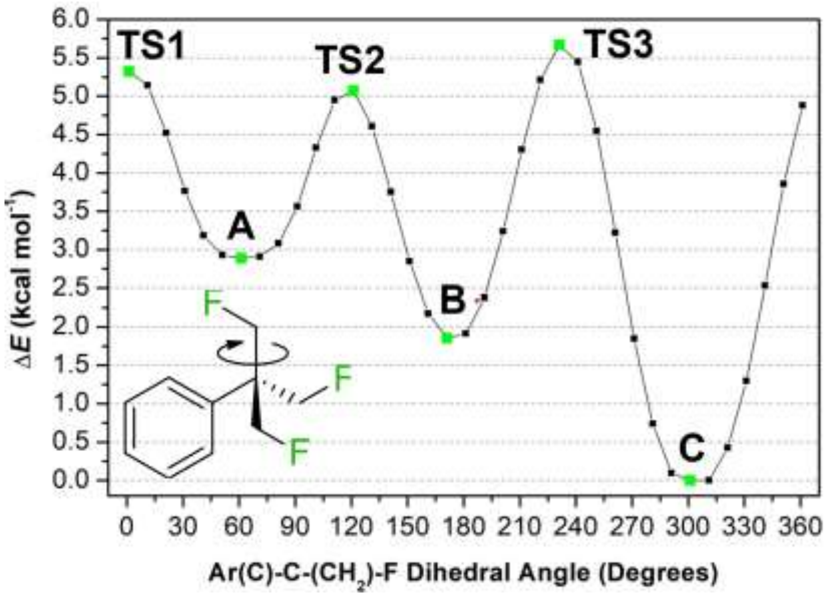

Figure S5. Calculated rotational energy profile of **18** rotating around one of the Ar(C)-C-CH_2-F bonds. Minima are labelled from **A** to **C** in decreasing relative energies. Calculations done at the M06-2X/def2-TZVP theoretical level.

Table S2. Thermodynamic parameters (in kcal mol^{-1}) calculated at standard temperature and pressure for the stationary points found in the relaxed PES for compound **18** obtained at the M06-2X/def2-TZVP.

Conformation	ΔE	ΔH	TΔS[a]	ΔG	Pop (%)[b]
**TS1**	5.1	4.6	-1.8	6.4	-
**A**	2.9	2.9	0.2	2.8	0.9
**TS2**	5.4	4.7	-1.6	6.3	-
**B**	1.9	1.9	-0.1	2.0	3.2
**TS3**	5.7	5.1	-1.4	6.5	-
**C**	0.0	0.0	0.0	0.0	95.9

[a] *Temperature of 298.15K.*

[b] *Calculated considering relative Gibbs free energies of conformers A-C.* k_b *= 0.001987 kcal/mol.K and T = 298.15K.*

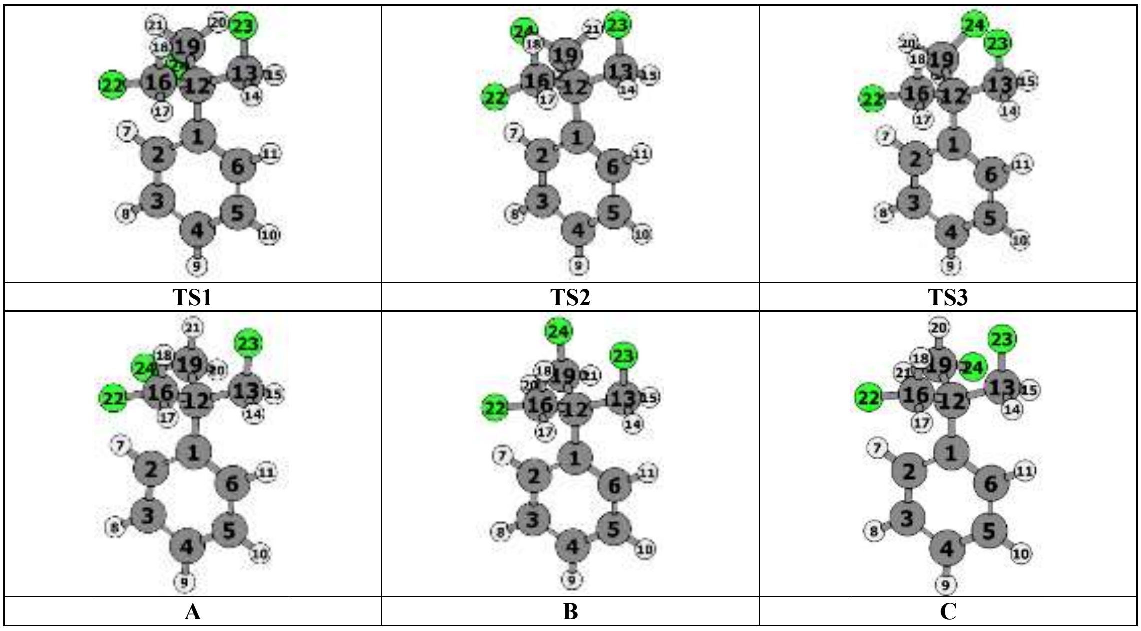

Figure S6. Conformation and atomic numeration for the stationary point geometries obtained in the calculated rotational energy profile of **18** rotating around one of the Ar(C)-C-CH_2-F bonds.

Table S3. NPA-derived atomic charges calculated at the M06-2X/def2-TZVP theoretical level for conformers A-C.

		NPA charge (au)		
**Atom**	**Number**	**Conformer A**	**Conformer B**	**Conformer C**
C	1	-0.02434	-0.01577	-0.01178
C	2	-0.19678	-0.21207	-0.20789
C	3	-0.20200	-0.20053	-0.19960
C	4	-0.20895	-0.20830	-0.21026
C	5	-0.20781	-0.20310	-0.20197
C	6	-0.22539	-0.22249	-0.21942
H	7	0.24492	0.23226	0.22458
H	8	0.21615	0.21701	0.21583
H	9	0.21517	0.21672	0.21579
H	10	0.21411	0.21578	0.21491
H	11	0.20568	0.21033	0.20872
C	12	-0.19677	-0.19997	-0.19804
C	13	0.06421	0.06501	0.05957
H	14	0.17563	0.16835	0.16907
H	15	0.17563	0.17166	0.18997
C	16	0.06586	0.06006	0.06468
H	17	0.16662	0.16885	0.17340
H	18	0.17822	0.19474	0.18127
C	19	0.06586	0.06986	0.06543
H	20	0.16662	0.17759	0.17813
H	21	0.17822	0.16719	0.17717
F	22	-0.35344	-0.36456	-0.36398
F	23	-0.36399	-0.35330	-0.36497
F	24	-0.35344	-0.35532	-0.36061

In addition to NBO analysis, Non-Covalent Interaction analysis (NCI) also reveals the attractive and repulsive nature of the CF···HC and CF···FC interactions, respectively, as illustrated in Figure S7. In the CF···HC contact, the region characterized by sign(λ_2)ρ ~-0.015 a.u. indicates the presence of an attractive interaction between F and H atoms, which are weaker in conformer **A** than in **C**, as indicated by the higher values of the reduced density

gradient (*s*) for the CF···HC contact in **A**. On the other hand, the CF··FC contact is characterized by a region of sign(λ_2)ρ ~+0.025 a.u., which indicates the presence of repulsive interactions between F atoms. In conformer **A**, the reduced density gradient approaches zero for the CF··FC contact, indicating stronger repulsive interactions compared to conformer **C**. Overall, NCI analysis is in accordance with NBO and reinforces the importance of the eletroctatic CF···HC and CF···FC interactions in determining the conformational equilibria between **A**, **B** and **C**.

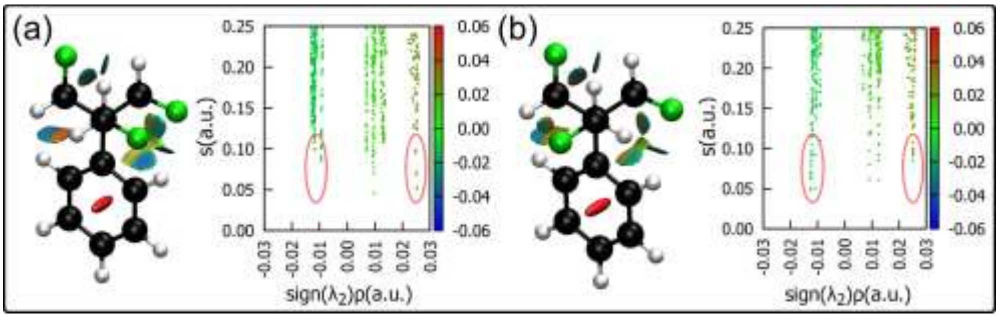

Figure S7. NCI iso-surfaces plotted using reduced density gradient (*s*) = 0.5 a.u. and blue-green-red colour scale ranging from -0.020 < sign(λ_2)ρ < +0.020 a.u. (left) and *s vs* sign(λ_2)ρ graphs (right) obtained from M06-2X/def2-TZVP electron density for **(a)** conformer **A** and **(b)** conformer **C**.

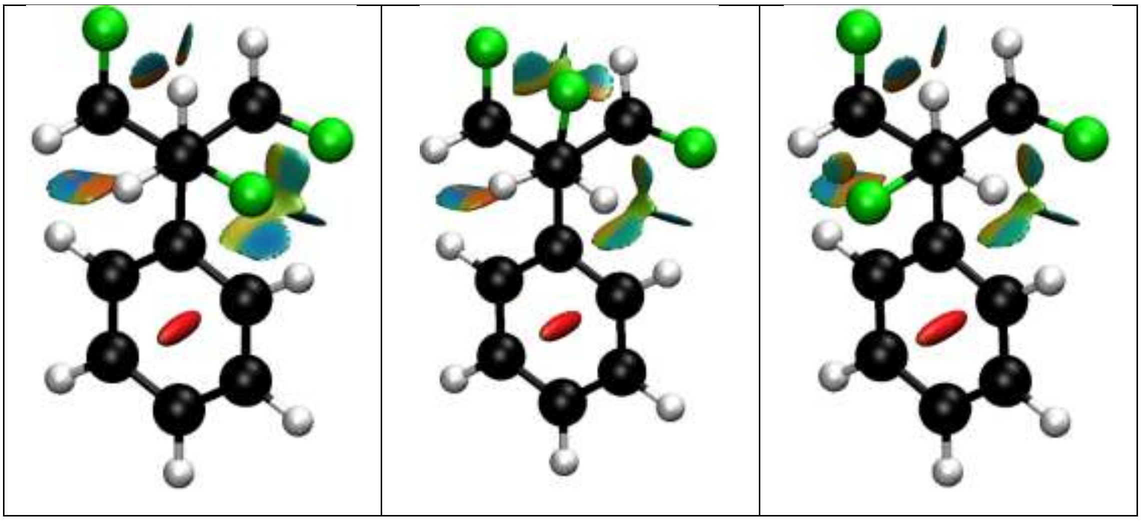

Figure S8. NCI iso-surfaces plotted using reduced density gradient (*s*) = 0.5 au and blue-green-red colour scale ranging from -0.020 < sign(λ_2)ρ < +0.020 au for conformers A-C (left to right).

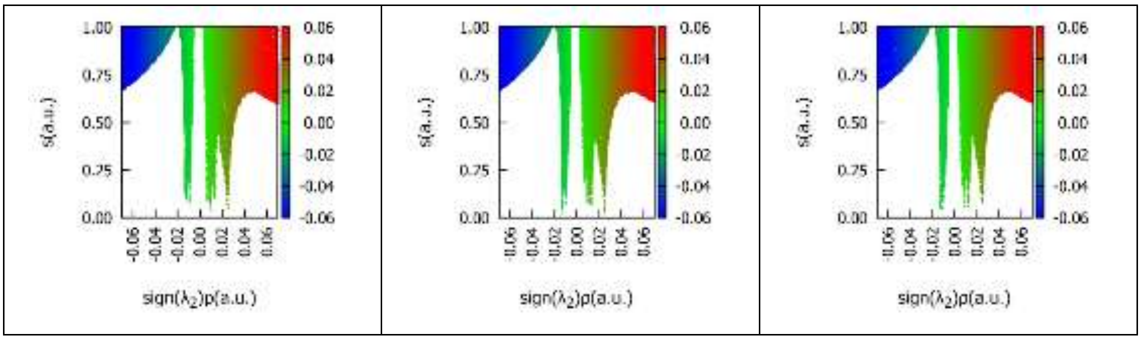

Figure S9. Graphs of *s vs* sign(λ_2)*ρ* obtained from M06-2X/def2-TZVP electron density for conformers A-C (left to right).

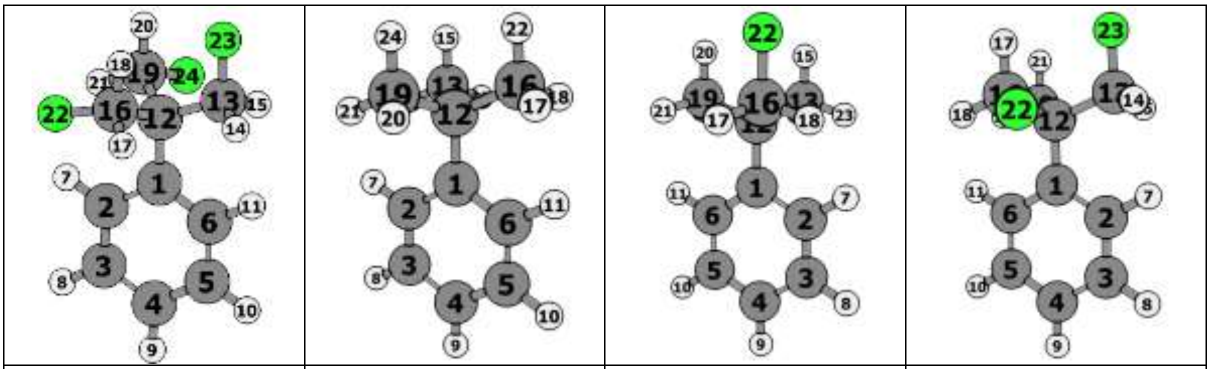

18	38	37	36
μ = 1.97 D	μ = 0.36 D	μ = 1.64 D	μ = 2.30 D

Figure S10. Calculated global minima geometries and molecular dipole moments obtained for compounds **18**, **36**, **37** and **38**.

Table S4. Cartesian coordinates, electronic energy and lowest harmonic vibrational frequency for TS1-TS3 and conformers A-C of compound **18** and the global minima of compounds **36**, **37** and **38**.

18 TS1<br>Energy (hartree) = -687.196220<br>Lowest harmonic vibrational frequency (cm^{-1}) = -105.49				18 A<br>Energy (hartree) = -687.200118<br>Lowest harmonic vibrational frequency (cm^{-1}) = 23.42			
C	-0.58467800	0.19397100	-0.02697400	C	-0.62702700	0.21365600	-0.00000200
C	-1.32724000	-0.98932100	-0.03635900	C	-1.33013400	-0.99471200	-0.00001200
C	-2.71096300	-0.95918400	-0.06682500	C	-2.71619900	-1.00850800	-0.00001800
C	-3.38867100	0.25296300	-0.08511900	C	-3.43465300	0.17872300	-0.00001000
C	-2.66626800	1.43314800	-0.07177600	C	-2.75155300	1.38308600	0.00000200
C	-1.27676000	1.40292900	-0.04315300	C	-1.36252200	1.39951000	0.00000600
H	-0.81605700	-1.94023500	-0.01208100	H	-0.78772300	-1.92709000	-0.00001300
H	-3.26347600	-1.88975700	-0.07154800	H	-3.23674700	-1.95742500	-0.00002900
H	-4.47038900	0.27432800	-0.10684600	H	-4.51672700	0.16408200	-0.00001300
H	-3.17823600	2.38660000	-0.08308800	H	-3.29537300	2.31884500	0.00001000
H	-0.75172700	2.34852000	-0.03389500	H	-0.87201100	2.36334100	0.00001000
C	0.93789600	0.13368300	-0.05190500	C	0.89880600	0.20650600	-0.00000300
C	1.54131700	1.53584700	-0.03934600	C	1.44413800	1.63622900	-0.00002100
H	1.27456500	2.10273200	-0.93376200	H	1.12357300	2.17839100	-0.89142200
H	1.23197500	2.08473700	0.85124600	H	1.12355800	2.17842300	0.89135600
C	1.41259700	-0.51597800	-1.35783500	C	1.45632200	-0.45107700	-1.26798500
H	0.92433400	-0.04586700	-2.21389000	H	1.07714300	0.07592600	-2.14742500
H	2.49641000	-0.42699200	-1.45203700	H	2.54681200	-0.41319200	-1.25993300
C	1.53697900	-0.64679000	1.15292400	C	1.45631000	-0.45104700	1.26799700
H	2.14197500	0.02056200	1.76741600	H	1.07715100	0.07599500	2.14742300
H	2.16183900	-1.46896100	0.80328400	H	2.54680100	-0.41319500	1.25994100
F	1.10622600	-1.86255400	-1.38061900	F	1.08622400	-1.77263900	-1.38121400
F	2.92417400	1.43799600	-0.00916600	F	2.82900500	1.62097900	-0.00000900
F	0.56999300	-1.18580700	1.97238600	F	1.08617200	-1.77259500	1.38126400

18 TS2<br>Energy (hartree) = -687.196600<br>Lowest harmonic vibrational frequency (cm^{-1}) = -89.49				18 B<br>Energy (hartree) = -687.201801<br>Lowest harmonic vibrational frequency (cm^{-1}) = 38.79			
C	0.70718500	0.19072200	-0.07525700	C	-0.76332200	0.13599700	0.00205300
C	1.33950300	-1.03418000	-0.29756900	C	-1.45489100	-1.07080400	0.14384300
C	2.72048800	-1.13010900	-0.31768900	C	-2.83956800	-1.09871100	0.19902500
C	3.50354700	0.00046600	-0.12165100	C	-3.56997800	0.07845400	0.12115600
C	2.89033300	1.22200900	0.09795800	C	-2.89960700	1.28269600	-0.01334200
C	1.50344200	1.31587000	0.12390600	C	-1.51200100	1.31094500	-0.07104200
H	0.73906800	-1.92365300	-0.44169000	H	-0.90780400	-2.00102800	0.19687700
H	3.18735600	-2.09180000	-0.48622800	H	-3.34873100	-2.04803600	0.30340100
H	4.58294000	-0.07295800	-0.14116800	H	-4.65087700	0.05610200	0.16568000
H	3.48781400	2.11164300	0.24922900	H	-3.45347200	2.21053300	-0.07405100
H	1.06193500	2.28809700	0.29789900	H	-1.03117600	2.27349100	-0.17525800
C	-0.81520000	0.24092900	-0.03425200	C	0.76402500	0.13440500	-0.04795100
C	-1.31585800	1.68422200	-0.03682700	C	1.28893900	1.55158600	-0.28218200
H	-0.99869200	2.22433300	0.85651500	H	0.87608700	1.96663600	-1.20517000
H	-0.97030000	2.21679600	-0.92564300	H	1.02749900	2.20164000	0.55535900
C	-1.31736500	-0.37962800	1.27869500	C	1.30129500	-0.72849800	-1.19351400
H	-0.96447300	0.23011200	2.11425200	H	0.83055200	-0.44016300	-2.13584700
H	-2.40522600	-0.43087700	1.28610900	H	2.38247200	-0.62071900	-1.26269900
C	-1.43564900	-0.48112900	-1.25933900	C	1.26573800	-0.41242100	1.29167900
H	-0.65994400	-0.85546100	-1.92537000	H	0.83018800	-1.39147200	1.48897500
H	-2.08626800	0.19949100	-1.80954800	H	0.99552400	0.27372000	2.09839400
F	-0.82765700	-1.65270800	1.45882400	F	1.03187600	-2.06808600	-0.97667600
F	-2.69916900	1.69091900	-0.04701300	F	2.66156500	1.56255000	-0.40677000
F	-2.21281400	-1.55718300	-0.88094500	F	2.63611000	-0.55919700	1.28855500
18 TS3<br>Energy (hartree) = -687.195663<br>Lowest harmonic vibrational frequency (cm^{-1}) = -125.12				18 C<br>Energy (hartree) = -687.204766<br>Lowest harmonic vibrational frequency (cm^{-1}) = 36.13			
C	0.71661900	-0.12662400	-0.02000200	C	0.62863100	-0.08531100	-0.09916300
C	1.47607700	0.97505900	0.38375700	C	1.39269200	0.98658900	0.36653000
C	2.85713200	0.90403900	0.45776000	C	2.77655600	0.92165200	0.38436400
C	3.51577200	-0.27562500	0.13714800	C	3.43108100	-0.21914100	-0.05923800
C	2.77656100	-1.37888500	-0.25327300	C	2.68621600	-1.29126200	-0.52084000
C	1.39052400	-1.30533300	-0.33206600	C	1.29861500	-1.22418100	-0.54085800
H	0.97992900	1.90299200	0.63575100	H	0.90352700	1.88747000	0.70986000
H	3.42072800	1.77420000	0.76841700	H	3.34558600	1.76712800	0.74868400
H	4.59460900	-0.33323100	0.19660500	H	4.51175100	-0.27108100	-0.04210500
H	3.27408300	-2.30859000	-0.49720500	H	3.18123200	-2.18968800	-0.86597500
H	0.85279500	-2.19417600	-0.63125400	H	0.75380700	-2.08490900	-0.90401200
C	-0.80318700	0.00753400	-0.08816400	C	-0.89207400	0.02664400	-0.12473700
C	-1.45354500	-1.33151000	-0.43688700	C	-1.53414600	-1.27515600	-0.60255200
H	-1.02367100	-1.72871300	-1.35786300	H	-1.24741600	-1.50522600	-1.63097700
H	-1.32996000	-2.05333700	0.37046200	H	-1.26136100	-2.10083500	0.05393300
C	-1.19645700	0.97236500	-1.21333300	C	-1.34654700	1.12287500	-1.09209700
H	-0.75633300	0.65455000	-2.16079900	H	-0.86390600	0.99084000	-2.06235600
H	-2.28144000	1.02219000	-1.30305300	H	-2.43083100	1.10078800	-1.20941300
C	-1.34705600	0.53902600	1.26737000	C	-1.43714800	0.34620200	1.26691100
H	-1.76583600	1.53963700	1.15147400	H	-2.52394000	0.43953800	1.23812500
H	-0.55303100	0.56768600	2.01240200	H	-1.00104900	1.26247700	1.66094700
F	-0.74311500	2.25231400	-0.94606800	F	-1.00864900	2.37799400	-0.61622400
F	-2.80438100	-1.16903600	-0.66933300	F	-2.91307000	-1.15297600	-0.57618300
F	-2.34211900	-0.27477700	1.77109200	F	-1.12168700	-0.68612500	2.12944800
36<br>Energy (hartree) = -389.457708<br>Lowest harmonic vibrational frequency (cm^{-1}) = 58.48				37<br>Energy (hartree) = -488.709007<br>Lowest harmonic vibrational frequency (cm^{-1}) = 56.26			
C	0.075292000	0.031896000	-0.000094000	C	0.414462000	0.084251000	0.098885000
C	0.785110000	-1.172005000	-0.000025000	C	1.038957000	-1.164557000	0.159459000
C	2.169660000	-1.195704000	-0.000023000	C	2.410985000	-1.292838000	0.015238000
C	2.888606000	-0.006912000	-0.000015000	C	3.199113000	-0.168511000	-0.190998000
C	2.202778000	1.195457000	0.000002000	C	2.597189000	1.077294000	-0.251758000
C	0.811857000	1.213136000	-0.000012000	C	1.219825000	1.200845000	-0.111191000
H	0.246843000	-2.112106000	-0.000026000	H	0.447889000	-2.056187000	0.328429000
H	2.690129000	-2.145023000	-0.000004000	H	2.866283000	-2.273522000	0.067091000
H	3.970663000	-0.021442000	0.000009000	H	4.271265000	-0.264978000	-0.301084000
H	2.747540000	2.131079000	0.000071000	H	3.198468000	1.963503000	-0.409673000
H	0.307825000	2.169486000	0.000018000	H	0.779935000	2.186840000	-0.162234000
C	-1.452637000	0.006906000	0.000014000	C	-1.102110000	0.181654000	0.246618000
C	-1.949529000	-0.729350000	-1.251963000	C	-1.557382000	-0.467530000	1.560086000
H	-1.589908000	-1.758246000	-1.281487000	H	-1.119720000	0.058882000	2.409237000
H	-3.041399000	-0.753342000	-1.263972000	H	-2.643378000	-0.420471000	1.642422000
C	-2.054004000	1.412121000	-0.000120000	C	-1.711565000	-0.574969000	-0.934370000
H	-1.756274000	1.976329000	0.885552000	H	-1.413858000	-0.114656000	-1.879656000
H	-1.756631000	1.976020000	-0.886106000	H	-1.412168000	-1.624557000	-0.932748000
C	-1.949344000	-0.729135000	1.252156000	C	-1.611447000	1.622008000	0.209067000

H	-1.605803000	-0.225040000	2.157139000	H	-2.696220000	1.625521000	0.306972000
H	-1.589633000	-1.758006000	1.281860000	H	-1.357819000	2.115622000	-0.730742000
H	-3.142843000	1.340493000	0.000153000	F	-3.095294000	-0.541342000	-0.866187000
H	-1.606029000	-0.225431000	-2.157053000	H	-1.258713000	-1.514136000	1.620414000
H	-3.041212000	-0.753221000	1.264337000	H	-1.192481000	2.204336000	1.031032000
38<br>Energy (hartree) = -587.958887<br>Lowest harmonic vibrational frequency (cm^{-1}) = 54.77							
C	0.538024000	-0.004872000	-0.179748000				
C	1.124664000	1.232037000	0.072805000				
C	2.504580000	1.362079000	0.177903000				
C	3.325134000	0.256010000	0.037158000				
C	2.754195000	-0.985619000	-0.209400000				
C	1.379232000	-1.110033000	-0.316517000				
H	0.517337000	2.118126000	0.196836000				
H	2.934145000	2.335809000	0.374828000				
H	4.399152000	0.356996000	0.120816000				
H	3.381959000	-1.860531000	-0.318528000				
H	0.956793000	-2.088873000	-0.508636000				
C	-0.969819000	-0.194413000	-0.305590000				
C	-1.701056000	1.140421000	-0.201223000				
H	-1.542287000	1.605128000	0.772334000				
H	-1.387617000	1.819466000	-0.996958000				
C	-1.474041000	-1.101545000	0.814109000				
H	-2.554445000	-1.234461000	0.736150000				
H	-0.979647000	-2.073978000	0.777741000				
C	-1.325435000	-0.849802000	-1.646233000				
H	-0.940192000	-0.247382000	-2.469645000				
H	-2.406396000	-0.935757000	-1.753753000				
F	-1.205455000	-0.535294000	2.046082000				
F	-3.063491000	0.933707000	-0.341911000				
H	-0.891153000	-1.845848000	-1.728305000				

Images of NMR spectra for synthesised compounds

2-(Hydroxymethyl)-2-phenylpropane-1,3-diol (16)

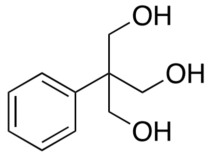

^{1}H NMR (400 MHz, d^6-DMSO)

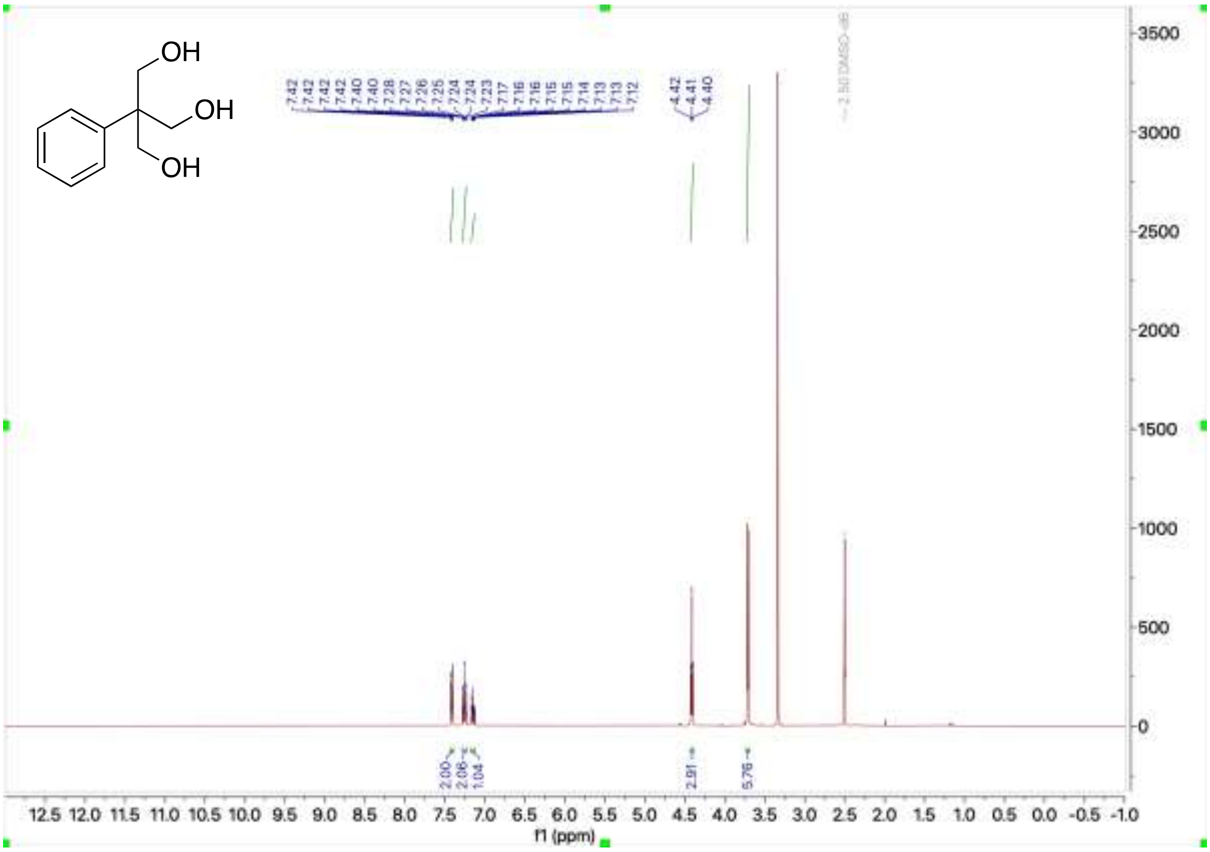

2-Phenyl-2-((tosyloxy)methyl)propane-1,3-diyl bis(4-methylbenzenesulfonate) (17)

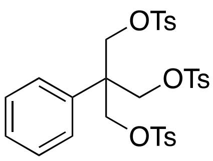

^{1}H NMR (500 MHz, $CDCl_3$):

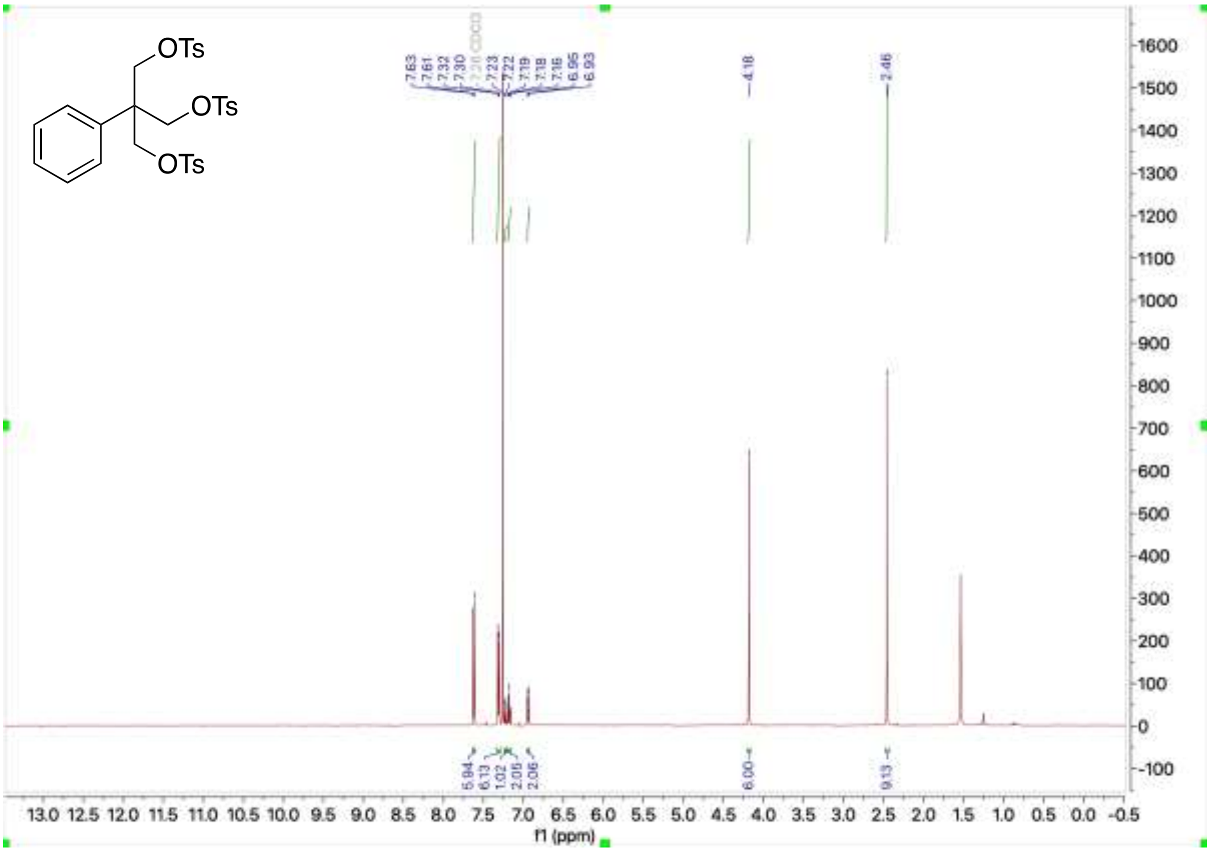

(1,3-Difluoro-2-(fluoromethyl)propan-2-yl)benzene (18)

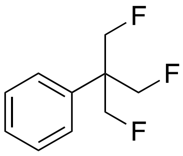

^{1}H NMR (400 MHz, $CDCl_3$):

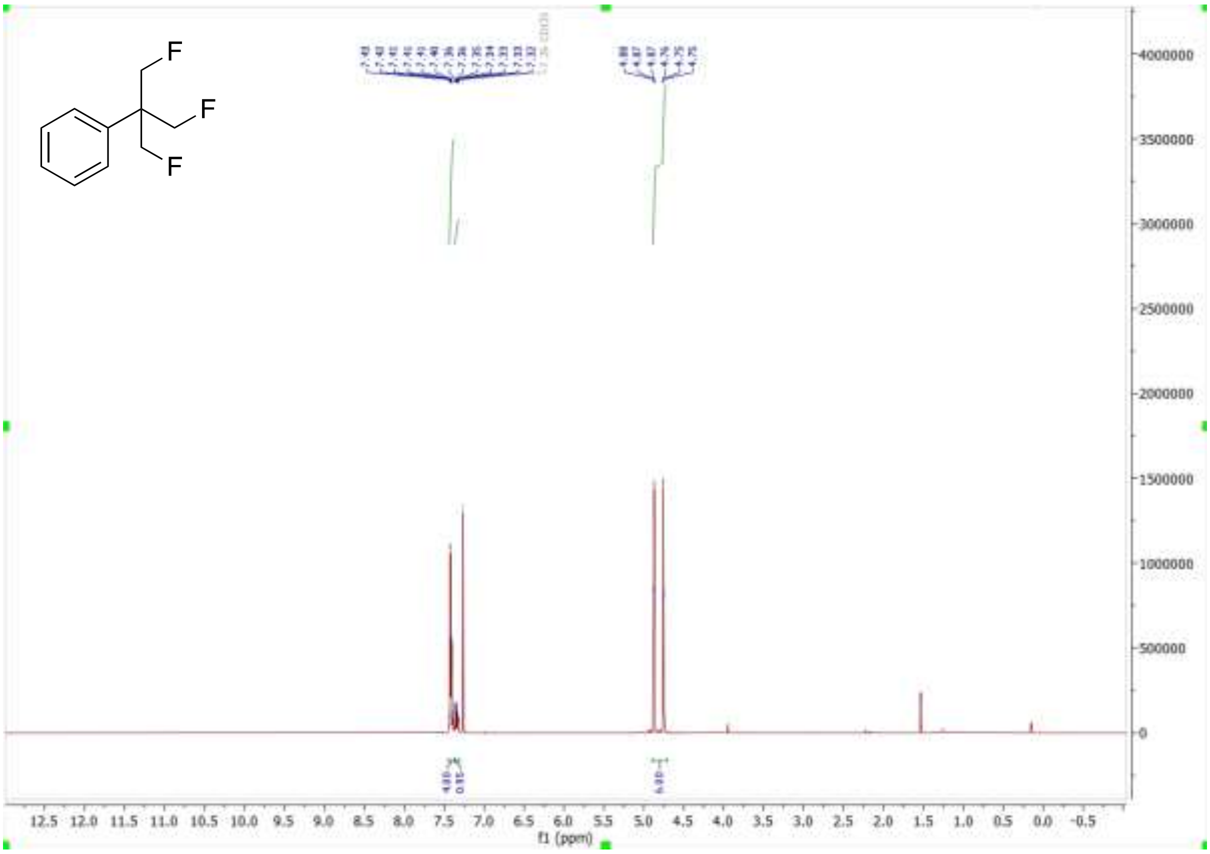

^{19}F NMR (376 MHz, $CDCl_3$):

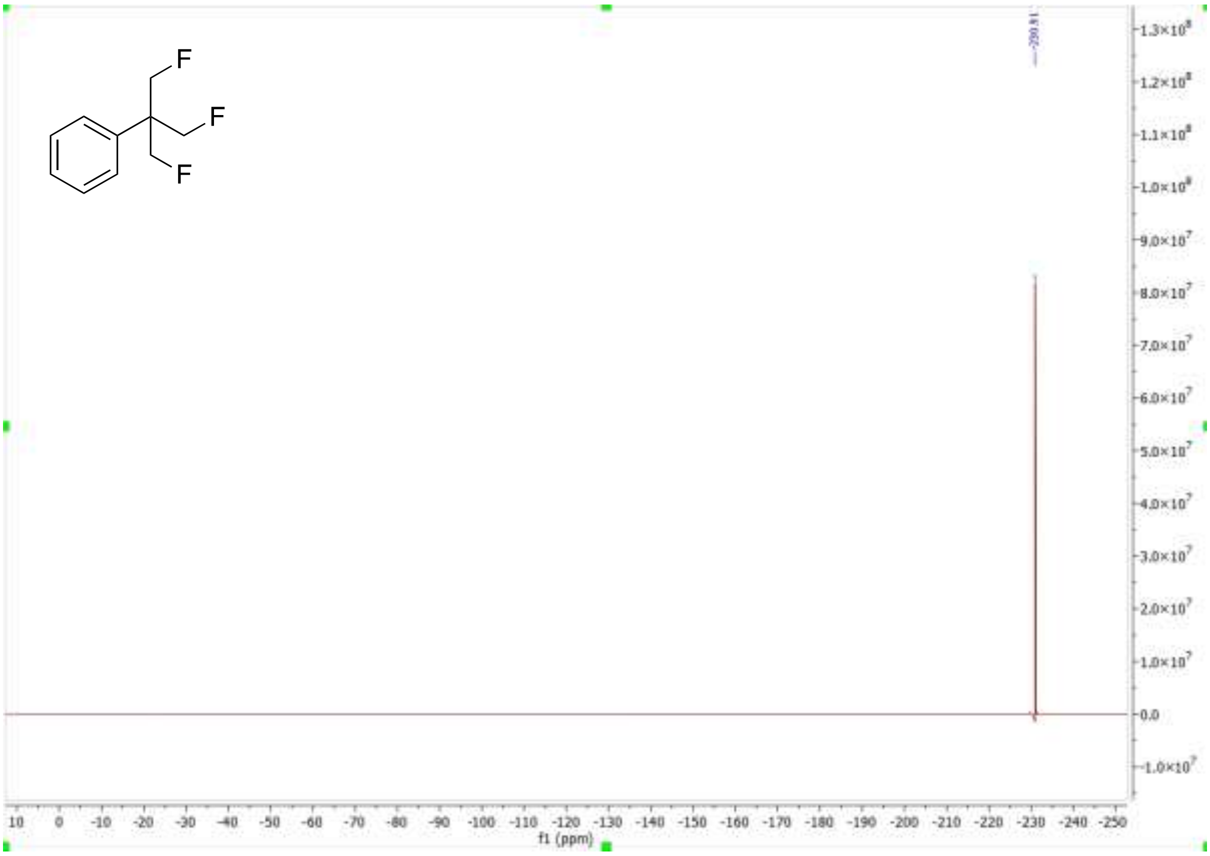

^{13}C NMR (101 MHz, $CDCl_3$):

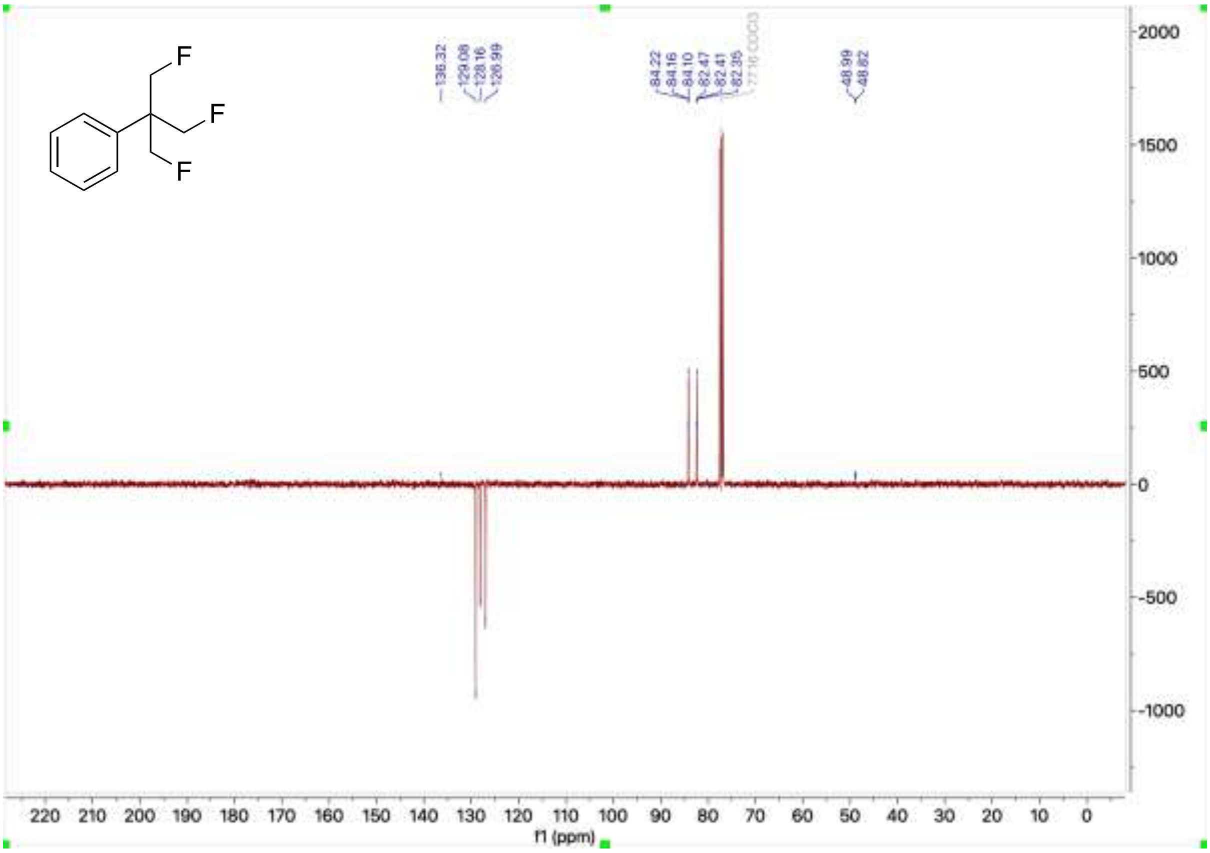

2-(4-Bromophenyl)acetaldehyde (19)

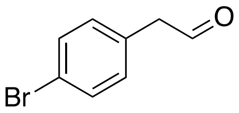

^{1}H NMR (400 MHz, $CDCl_3$):

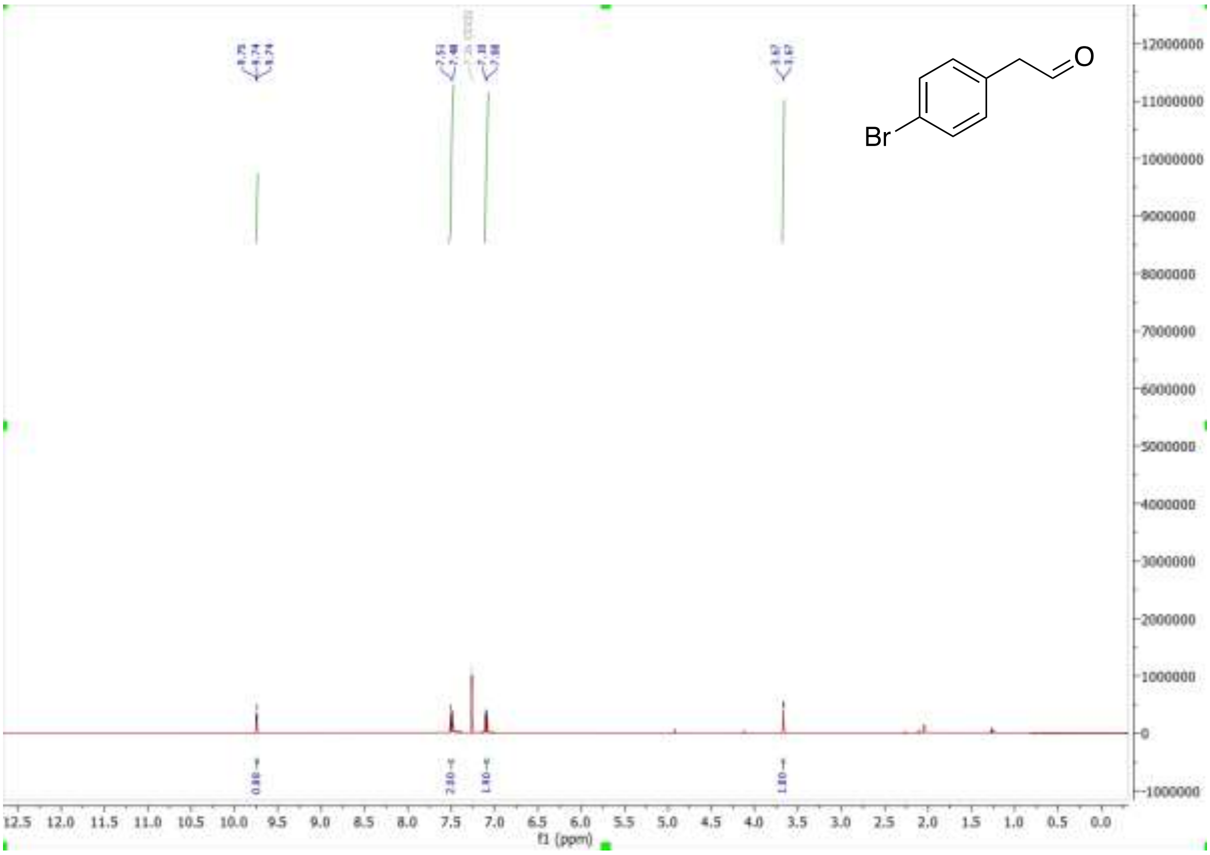

2-(4-Bromophenyl)-2-(hydroxymethyl)propane-1,3-diol (20)

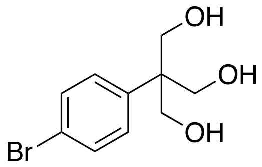

^{1}H NMR (400 MHz, d^6-DMSO):

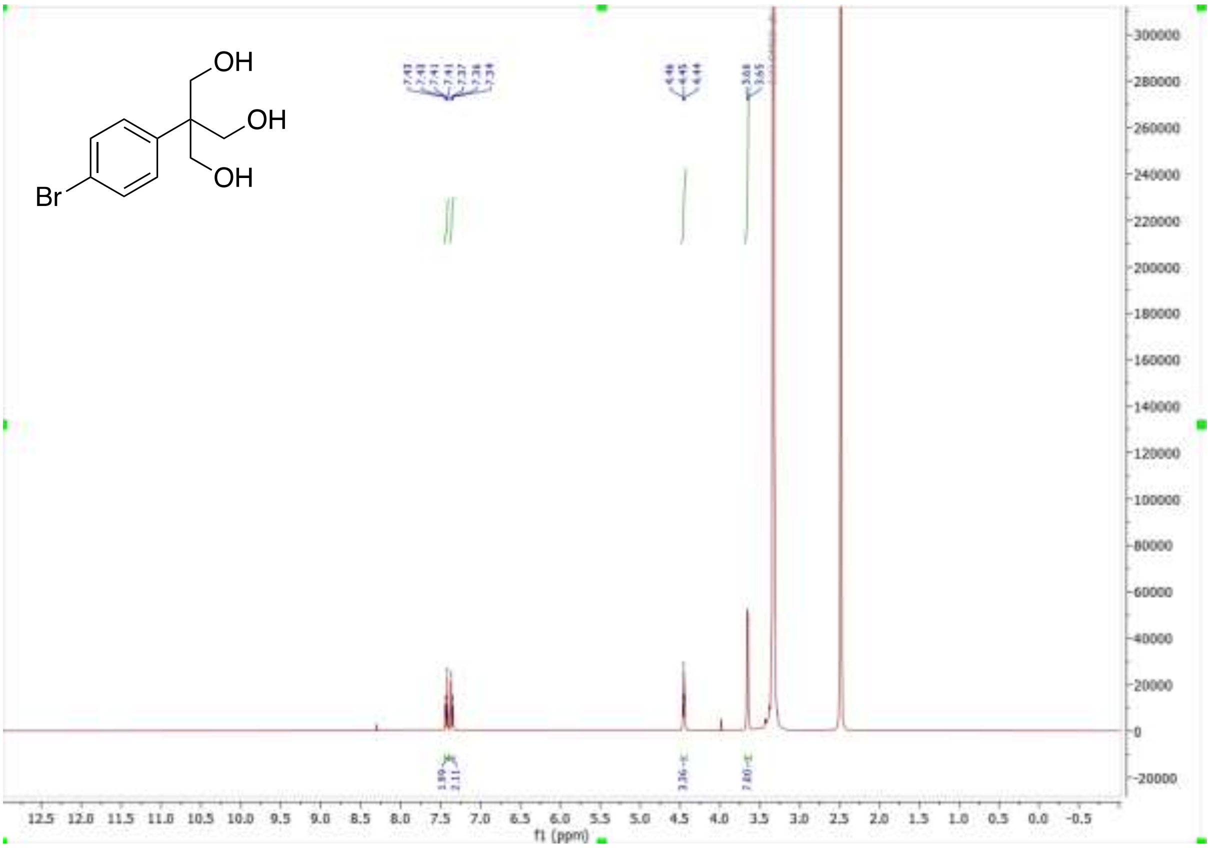

2-(4-Bromophenyl)-2-((tosyloxy)methyl)propane-1,3-diyl bis(4-methylbenzenesulfonate) (21)

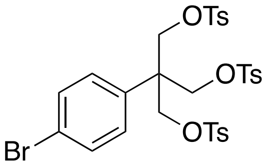

^{1}H NMR (400 MHz, $CDCl_3$):

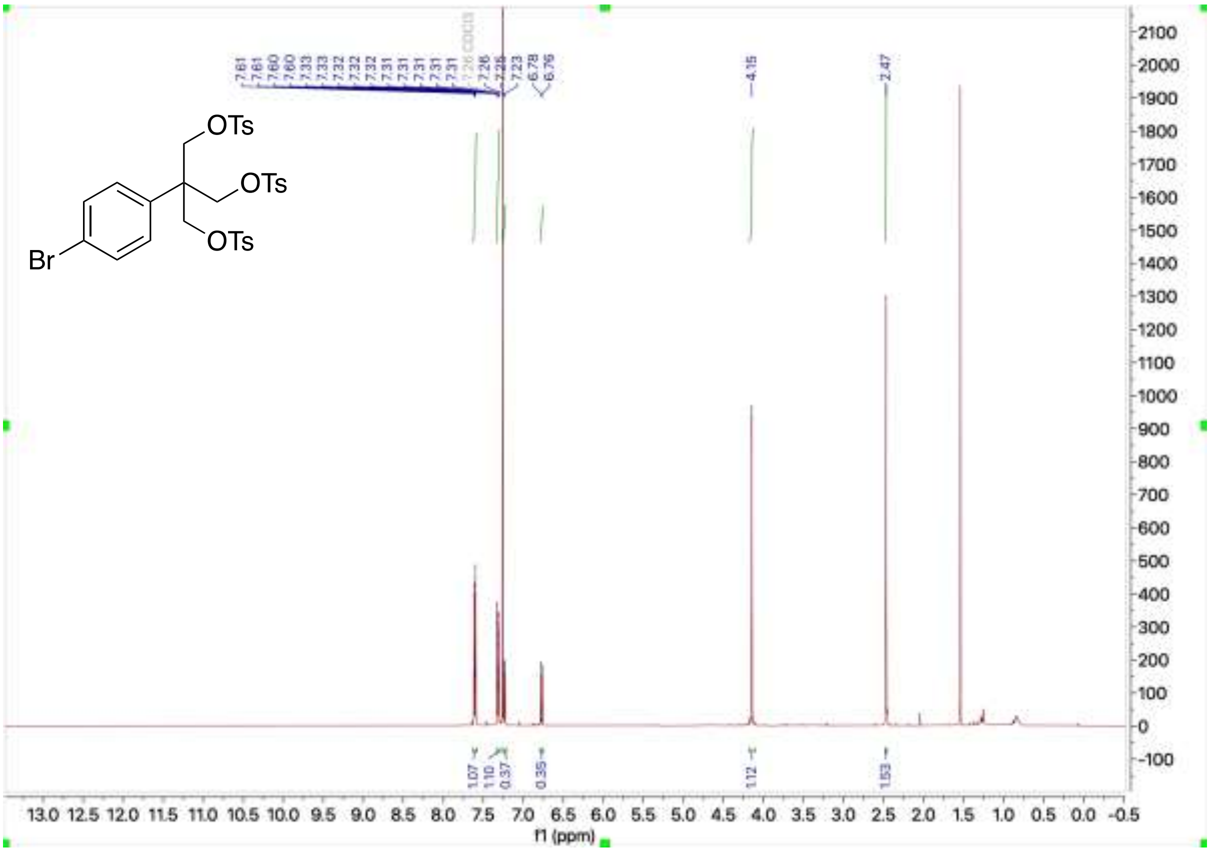

^{13}C NMR (126 MHz, $CDCl_3$):

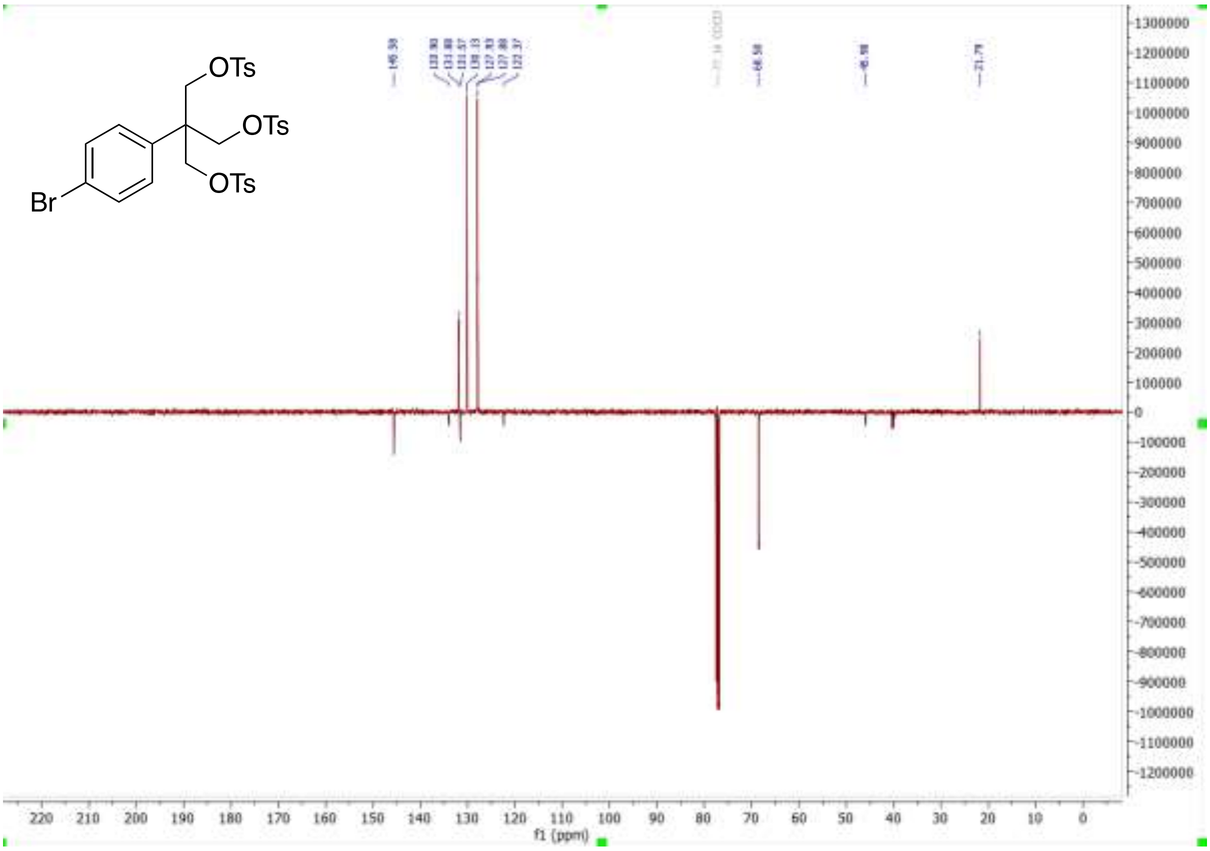

1-Bromo-4-(1,3-difluoro-2-(fluoromethyl)propan-2-yl)benzene (22)

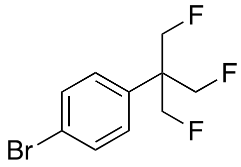

^{1}H NMR (400 MHz, $CDCl_3$):

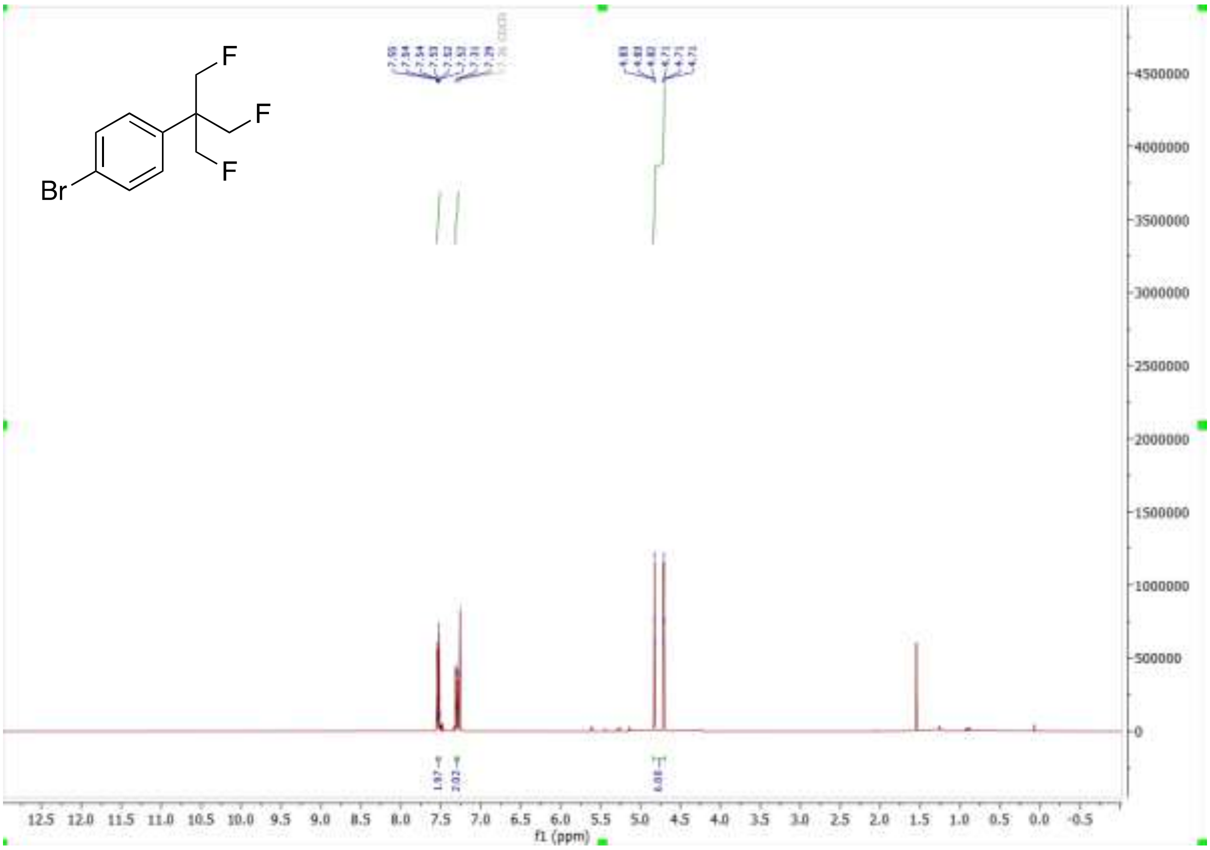

^{19}F NMR (376 MHz, $CDCl_3$):

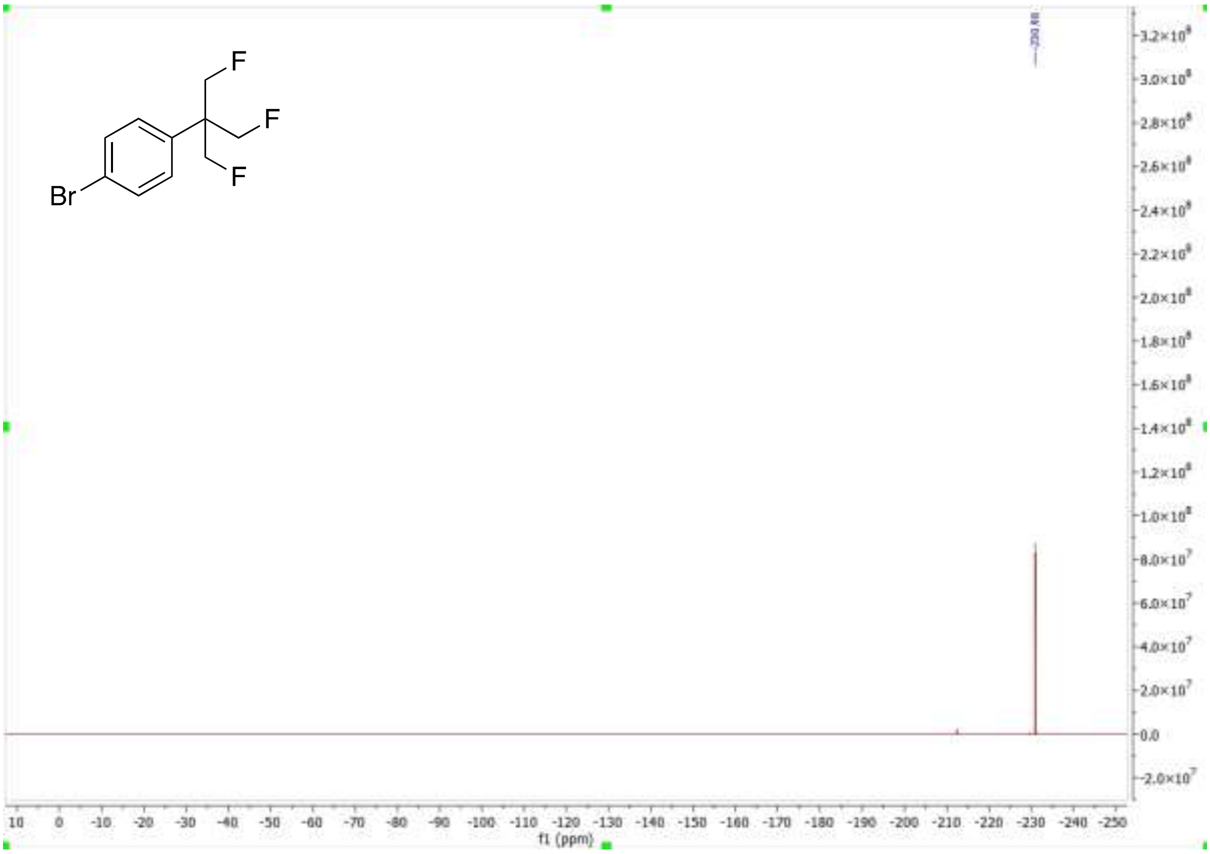

^{13}C NMR (126 MHz, $CDCl_3$):

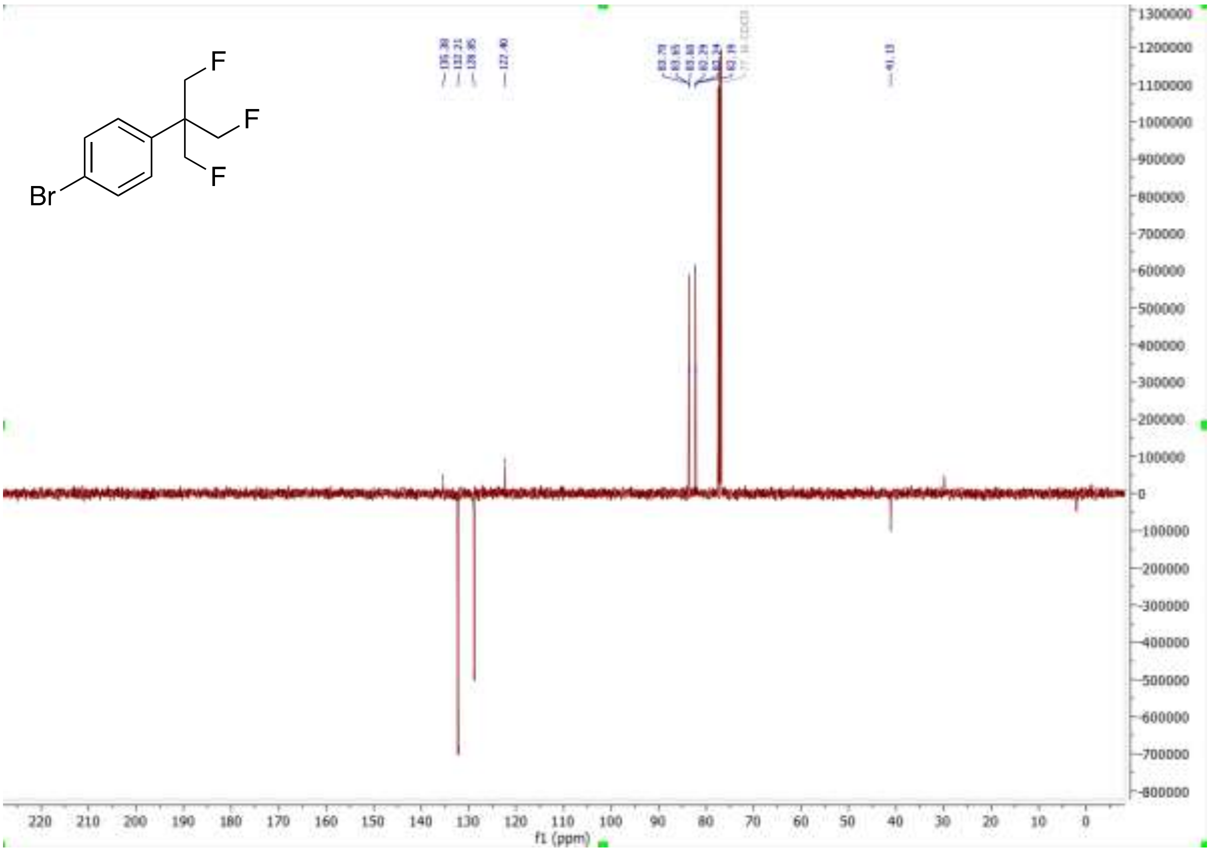

4-(1,3-Difluoro-2-(fluoromethyl)propan-2-yl)-4'-methyl-1,1'-biphenyl (23)

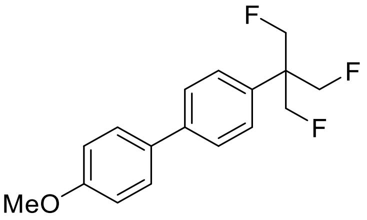

^{1}H NMR (500 MHz, $CDCl_3$):

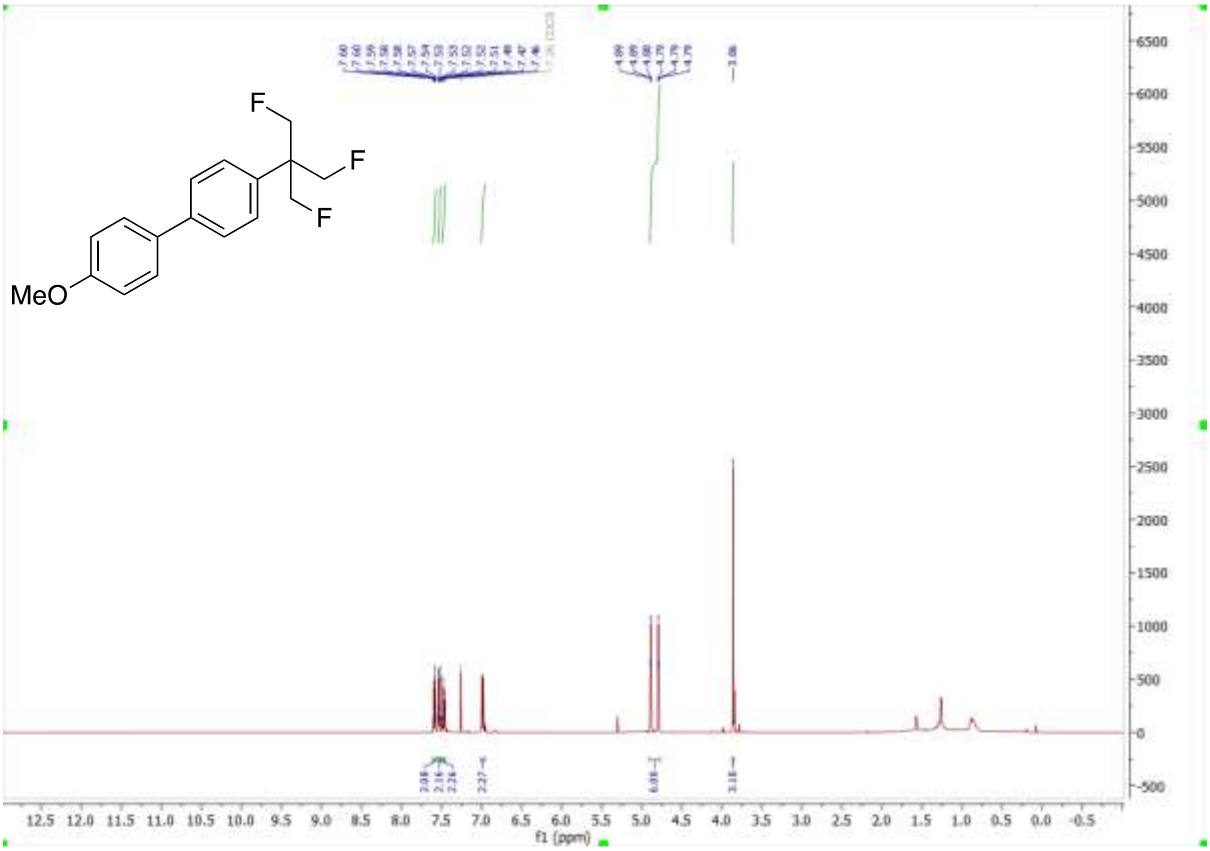

$^{19}F\{^{1}H\}$ NMR (377 MHz, $CDCl_3$):

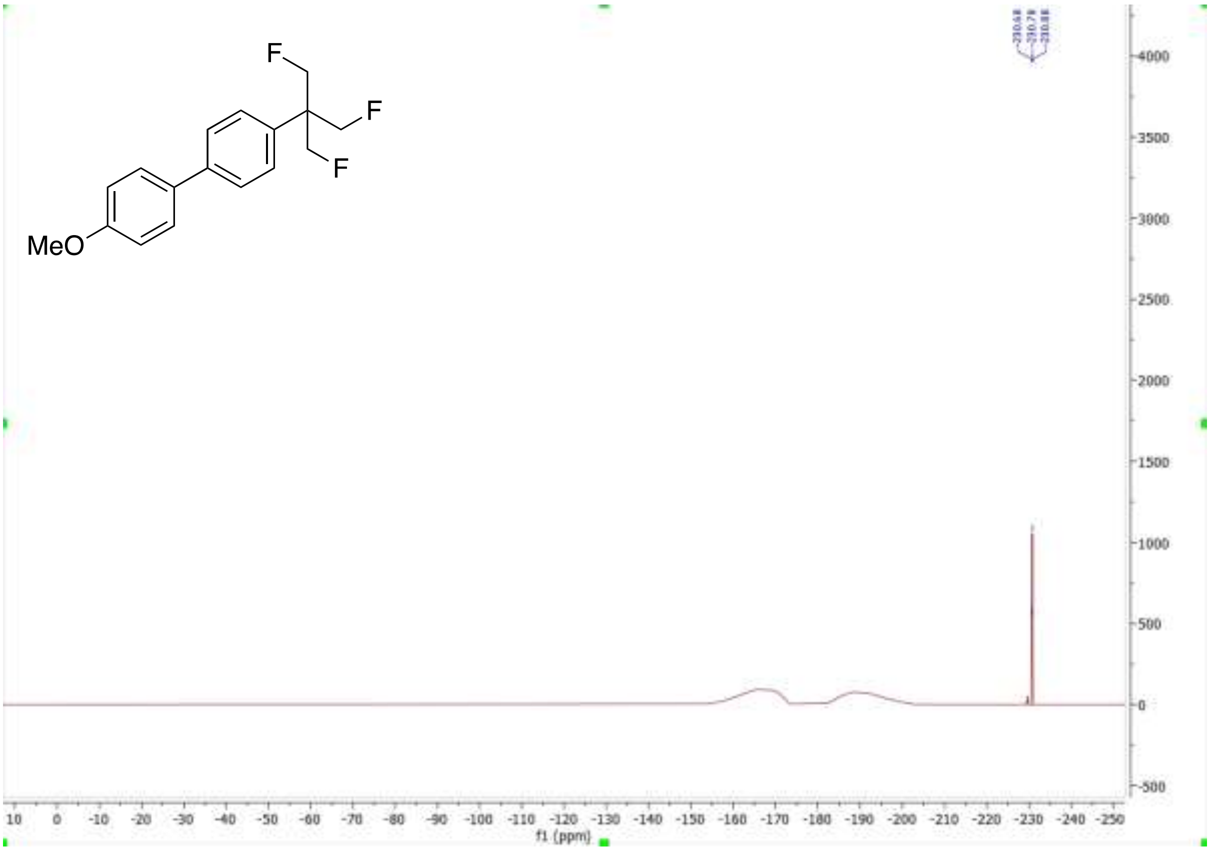

^{13}C NMR (126 MHz, $CDCl_3$):

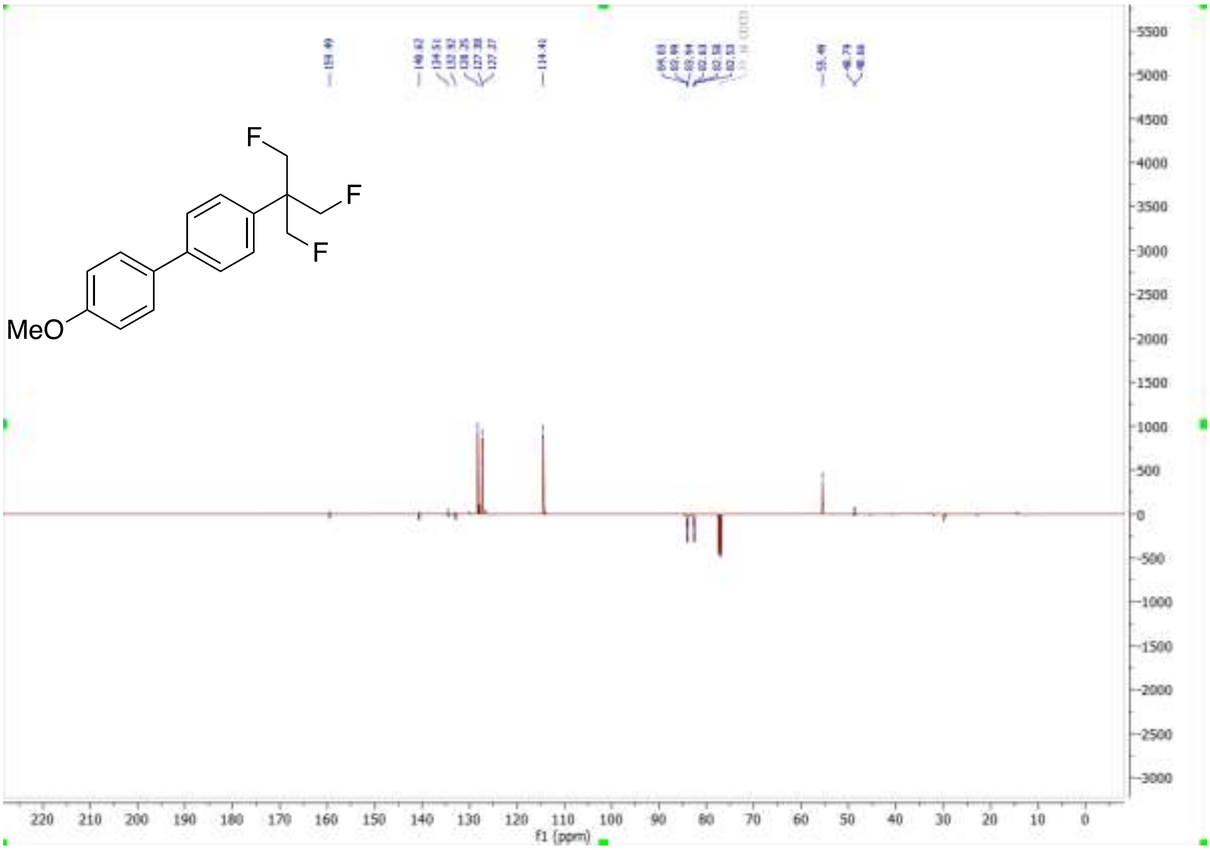

Methyl 4'-(1,3-difluoro-2-(fluoromethyl)propan-2-yl)-[1,1'-biphenyl]-4-carboxylate (24)

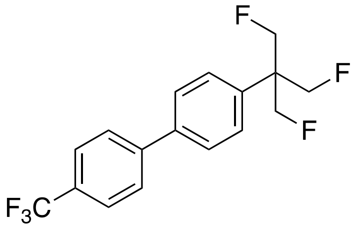

^1H NMR (500 MHz, $CDCl_3$):

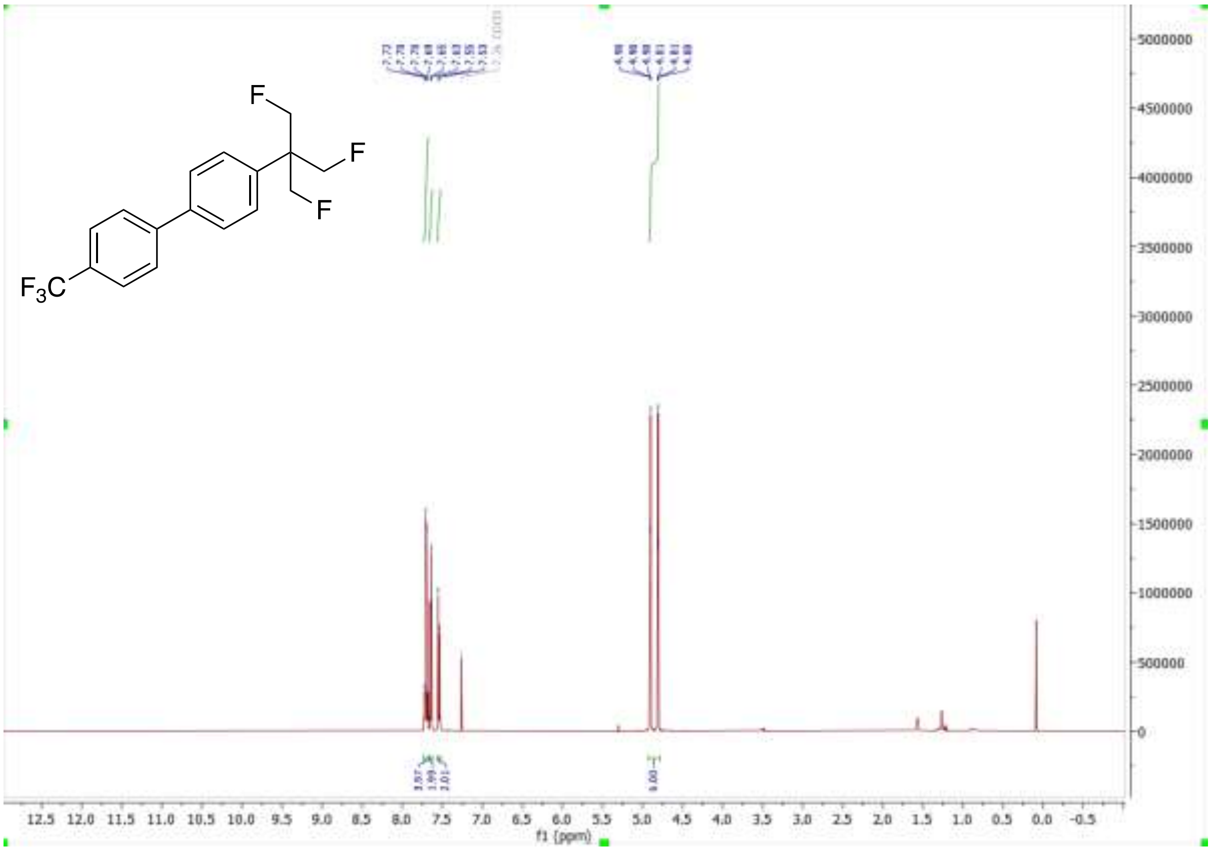

^{19}F NMR (376 MHz, $CDCl_3$):

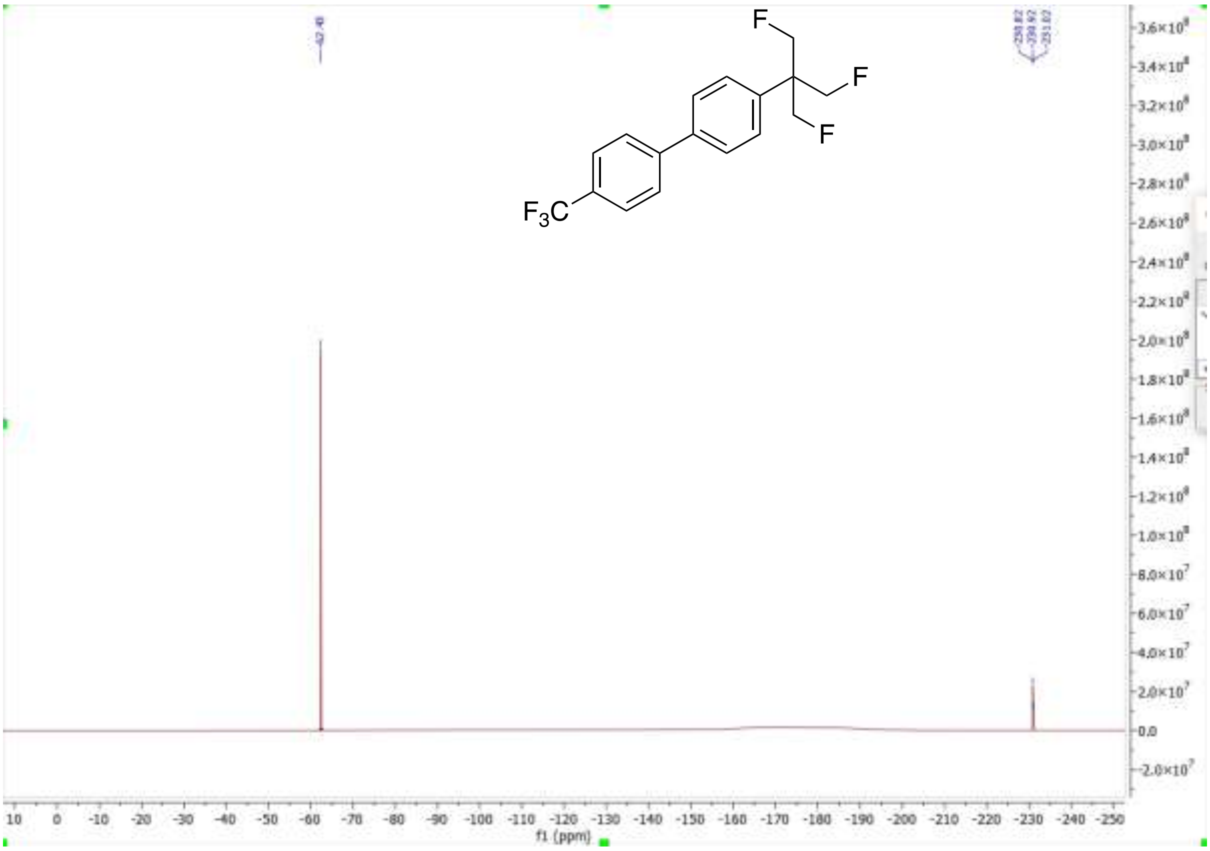

^{13}C NMR (126 MHz, $CDCl_3$):

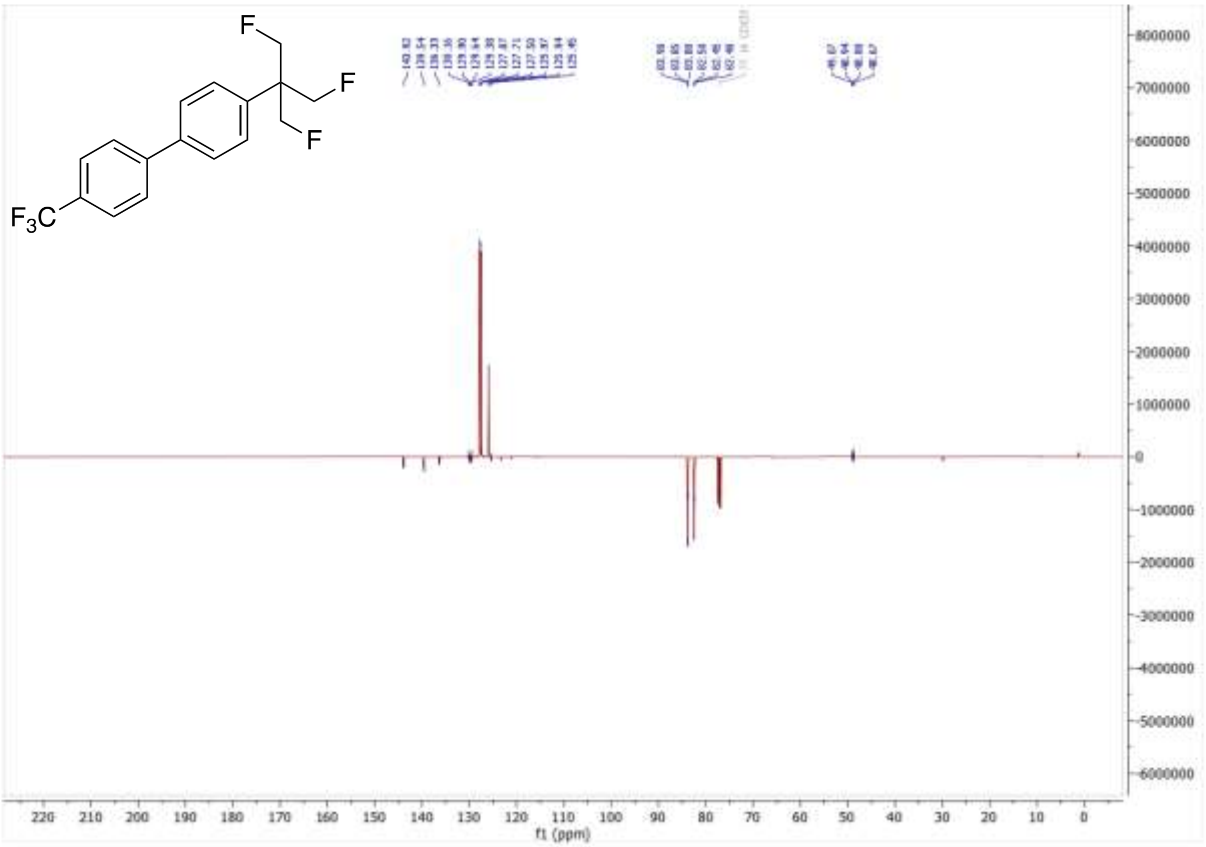

3-(4-(1,3-Difluoro-2-(fluoromethyl)propan-2-yl)phenyl)pyridine (25)

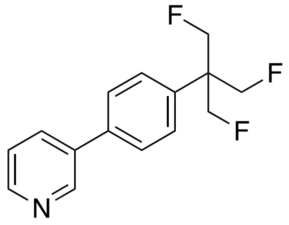

^{1}H NMR (500 MHz, $CDCl_3$):

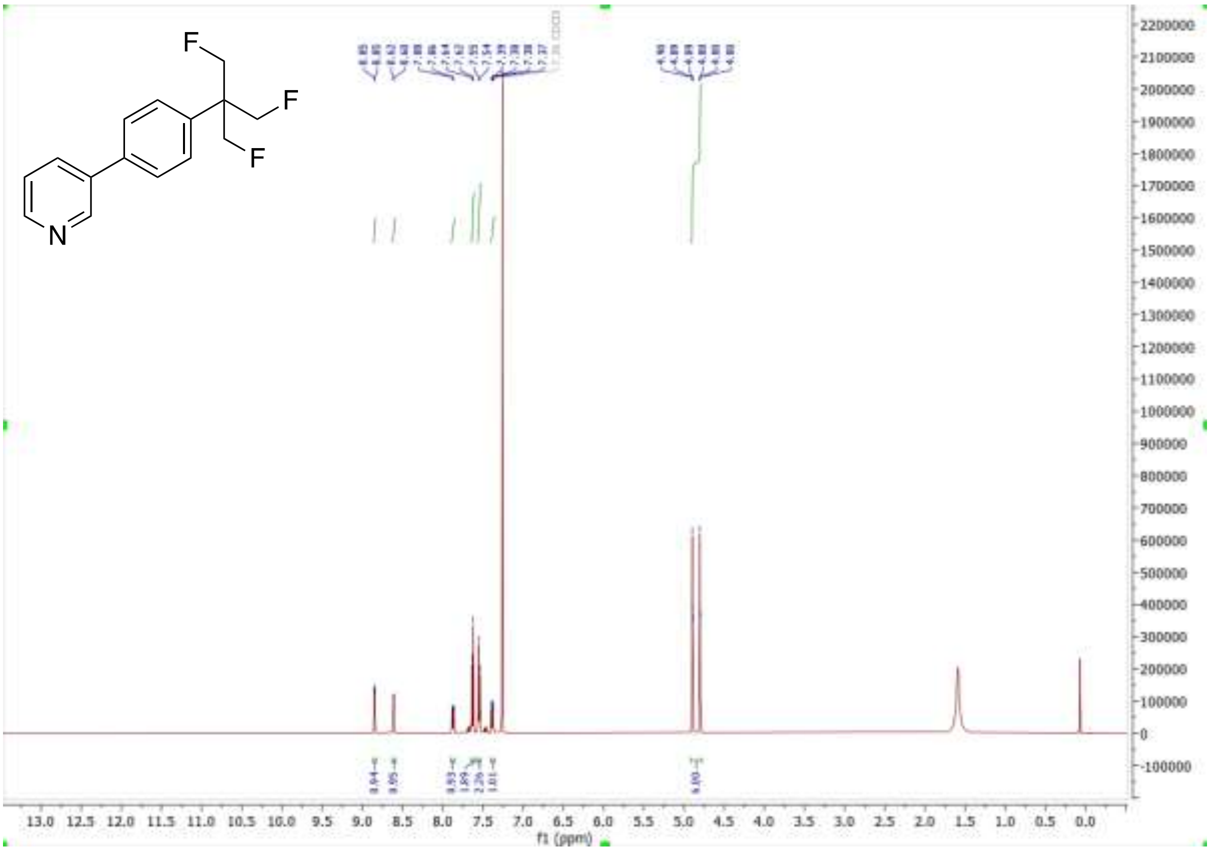

$^{19}F\{^1H\}$ NMR (470 MHz, $CDCl_3$):

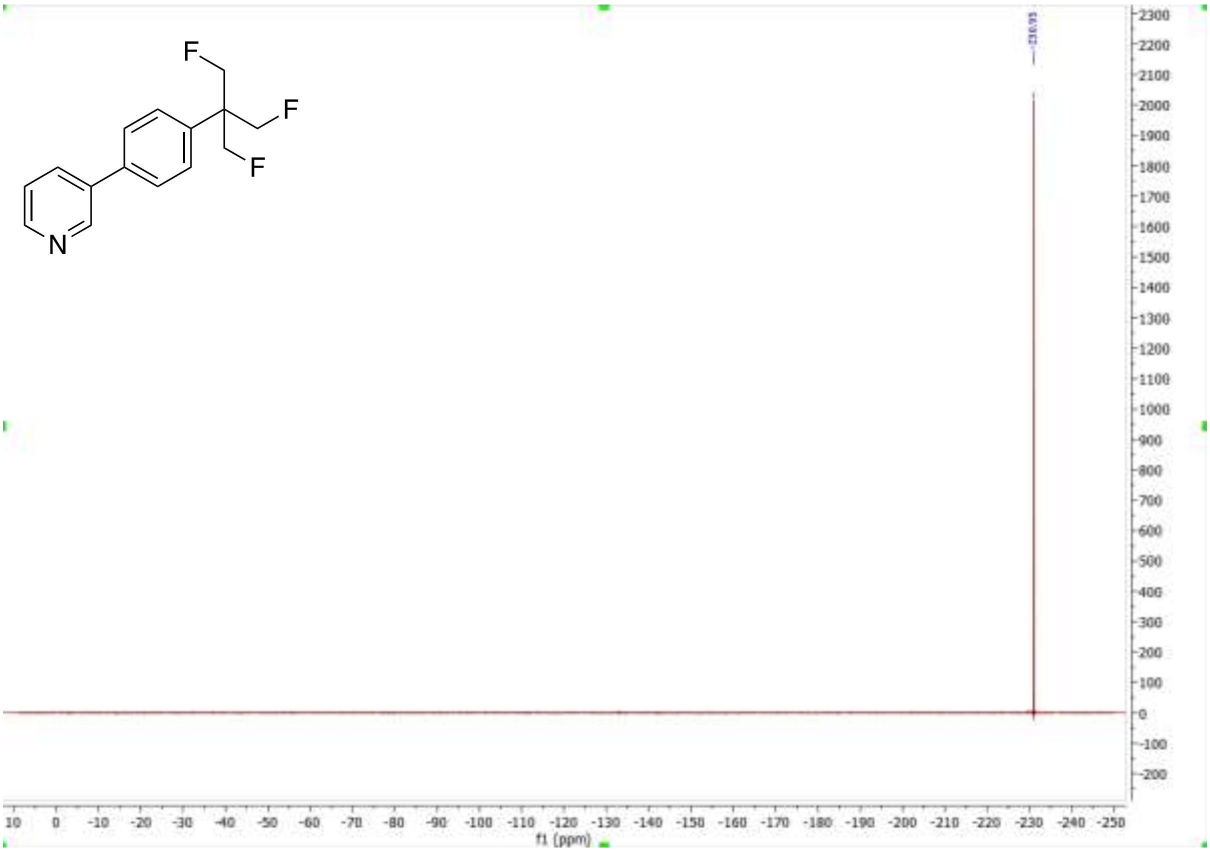

^{13}C NMR (126 MHz, $CDCl_3$):

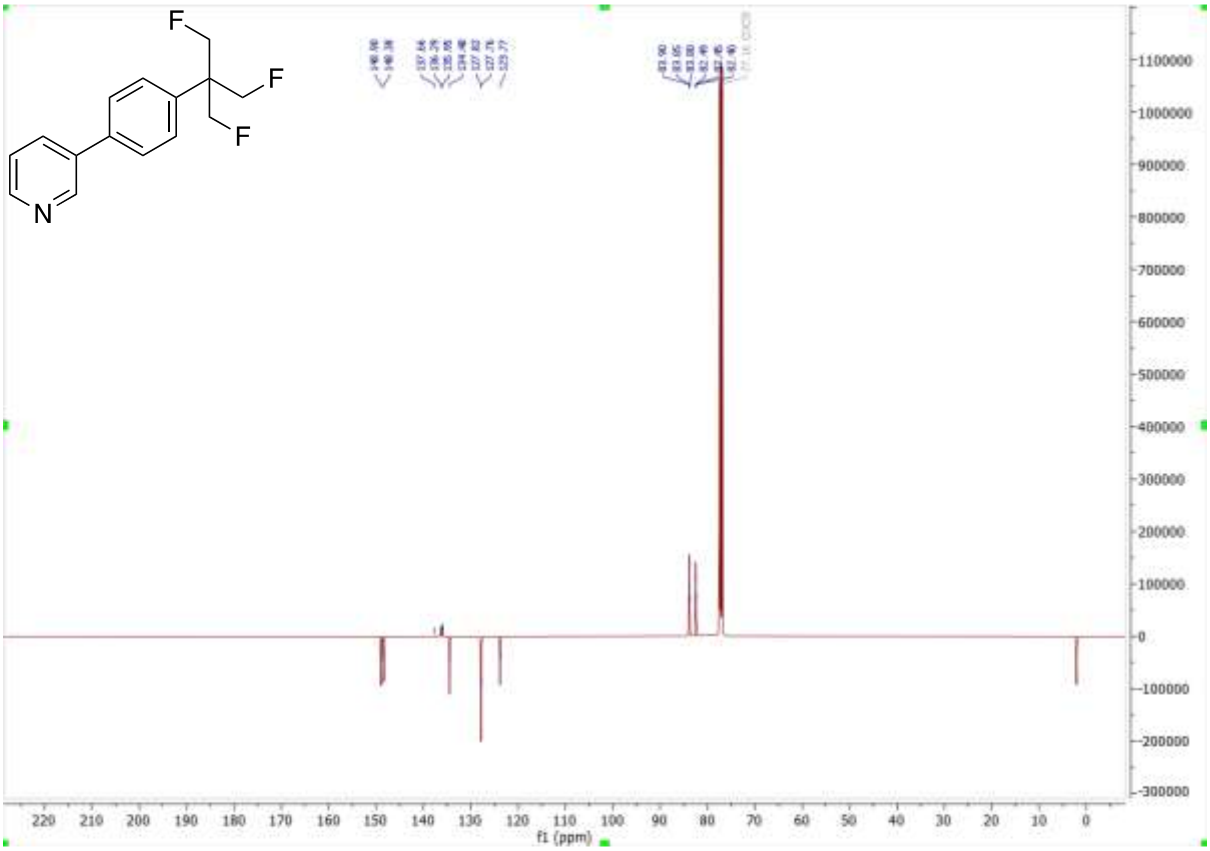

4-(1,3-Difluoro-2-(fluoromethyl)propan-2-yl)-4'-methyl-1,1'-biphenyl (26)

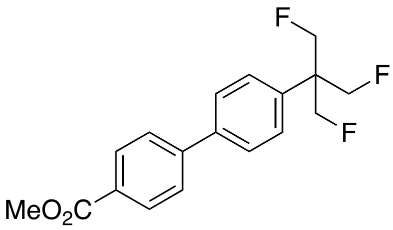

^{1}H NMR (500 MHz, $CDCl_3$):

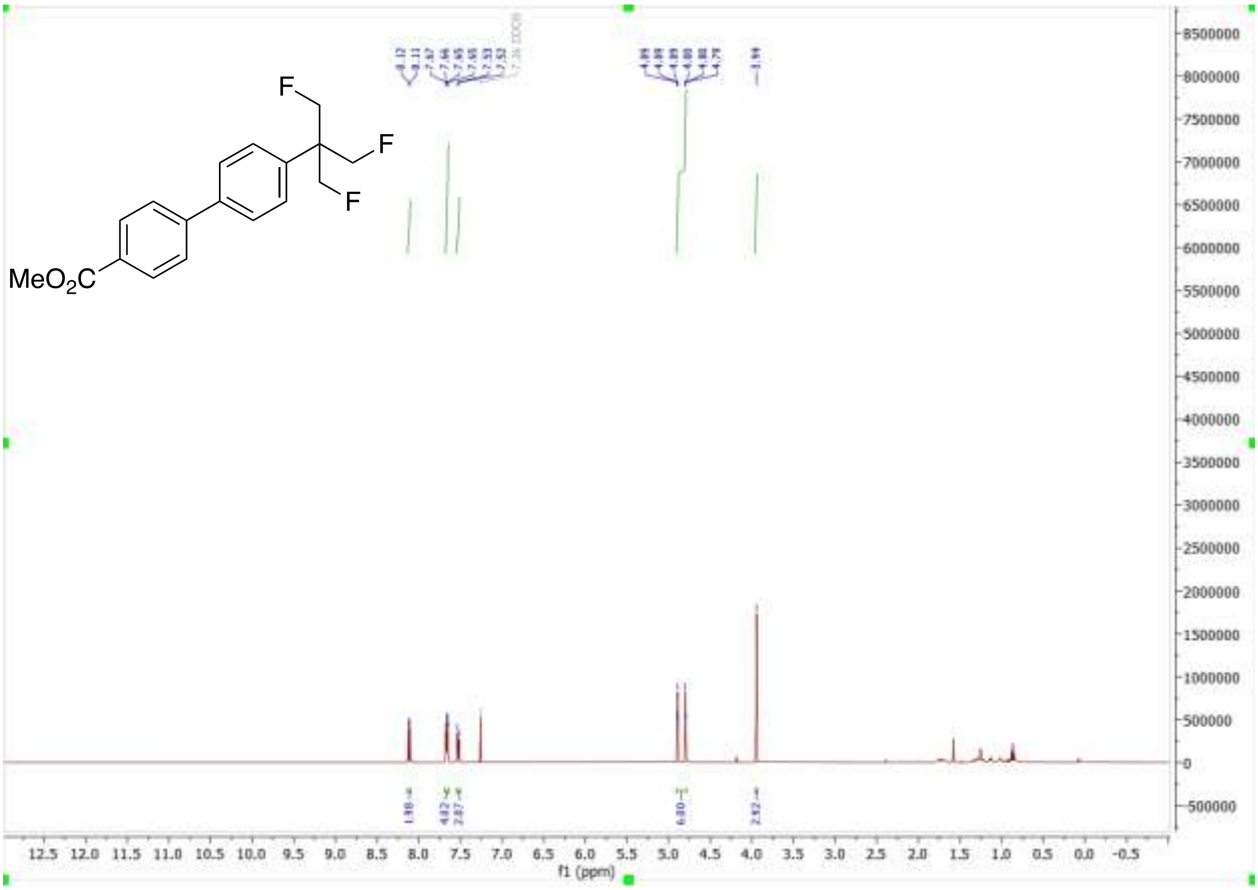

$^{19}F\{^{1}H\}$ NMR (376 MHz, $CDCl_3$):

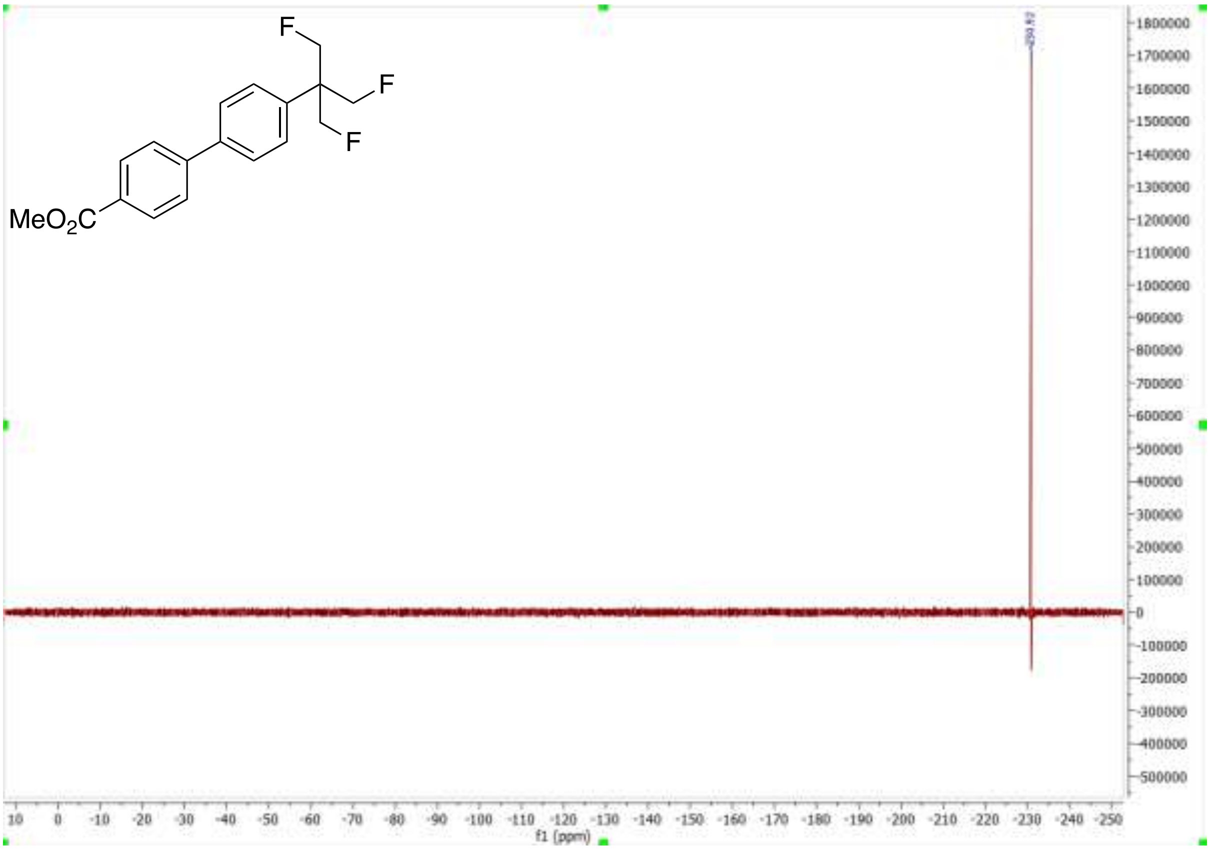

^{13}C NMR (126 MHz, $CDCl_3$):

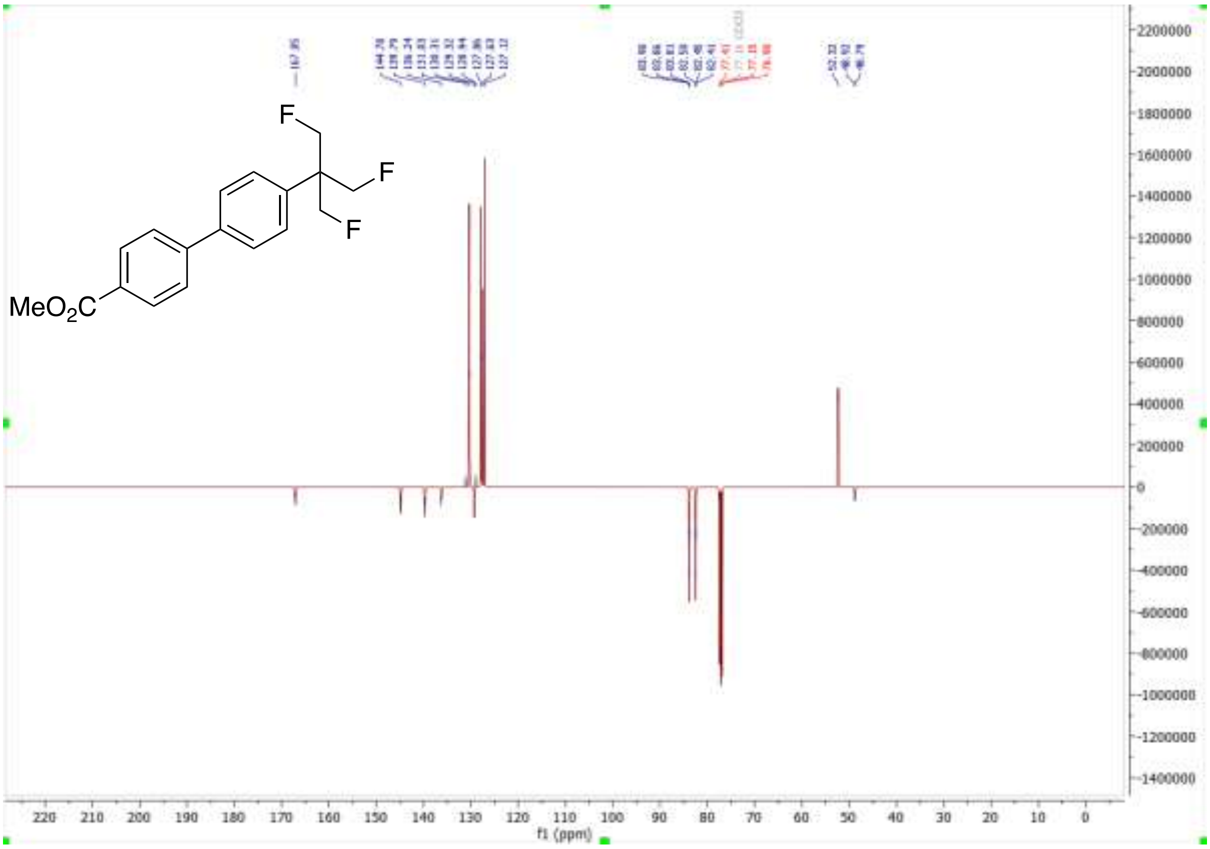

4-(1,3-Difluoro-2-(fluoromethyl)propan-2-yl)-4'-methyl-1,1'-biphenyl (27)

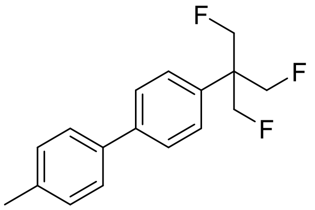

^{1}H NMR (500 MHz, $CDCl_3$):

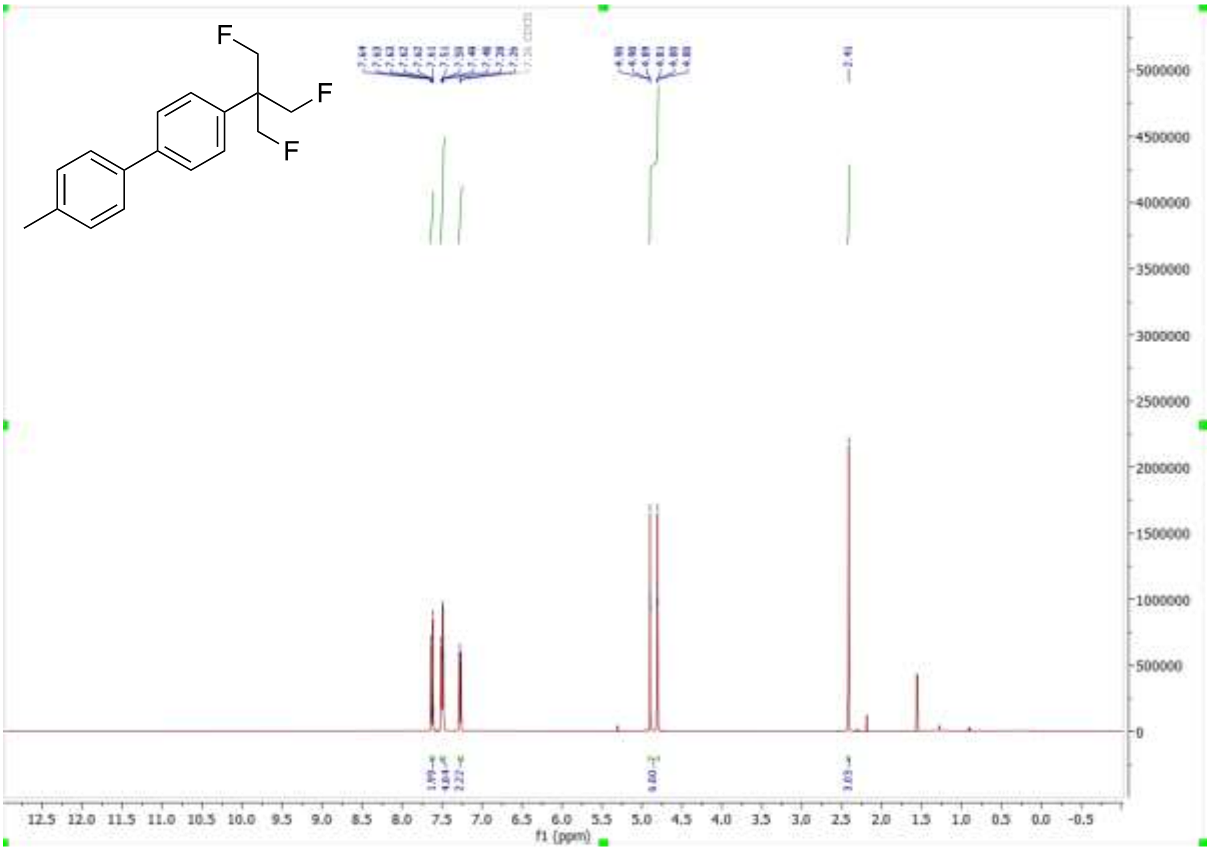

^{13}C NMR (126 MHz, $CDCl_3$):

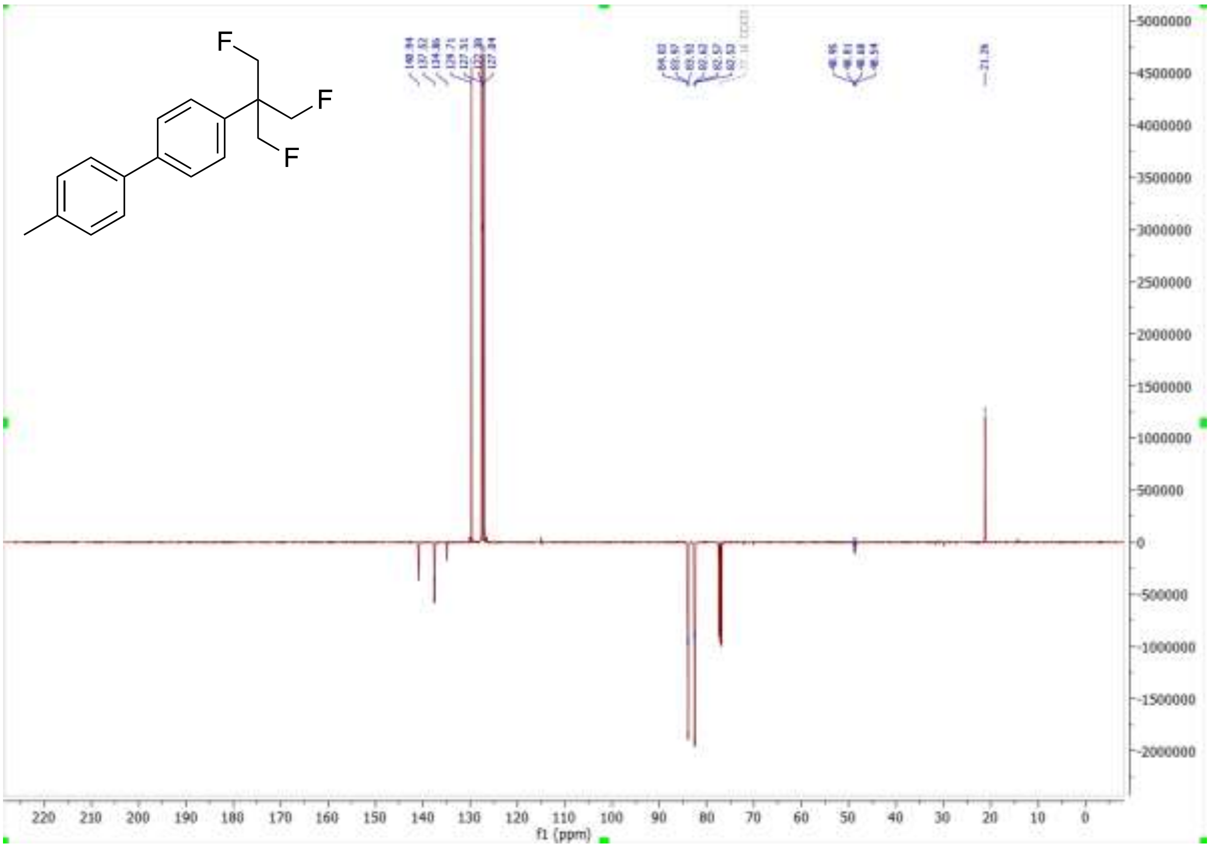

$^{19}F\{^1H\}$ NMR (376 MHz, $CDCl_3$):

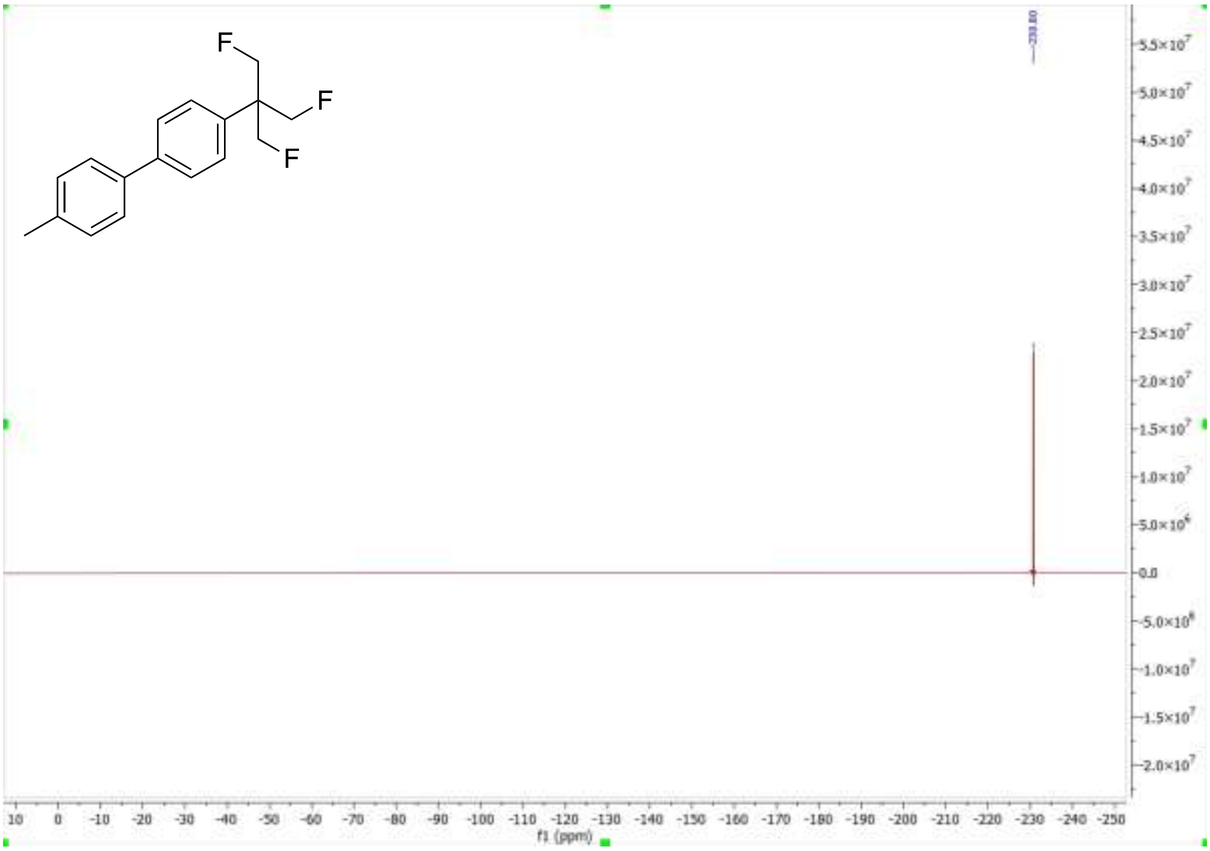

4'-(1,3-Difluoro-2-(fluoromethyl)propan-2-yl)-3,4,5-trifluoro-1,1'-biphenyl (28)

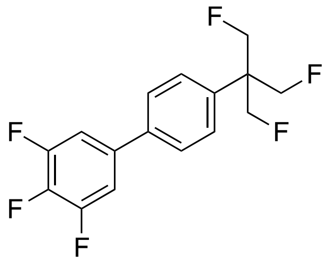

^{1}H NMR (400 MHz, $CDCl_3$) (mixture of product and starting material **22**):

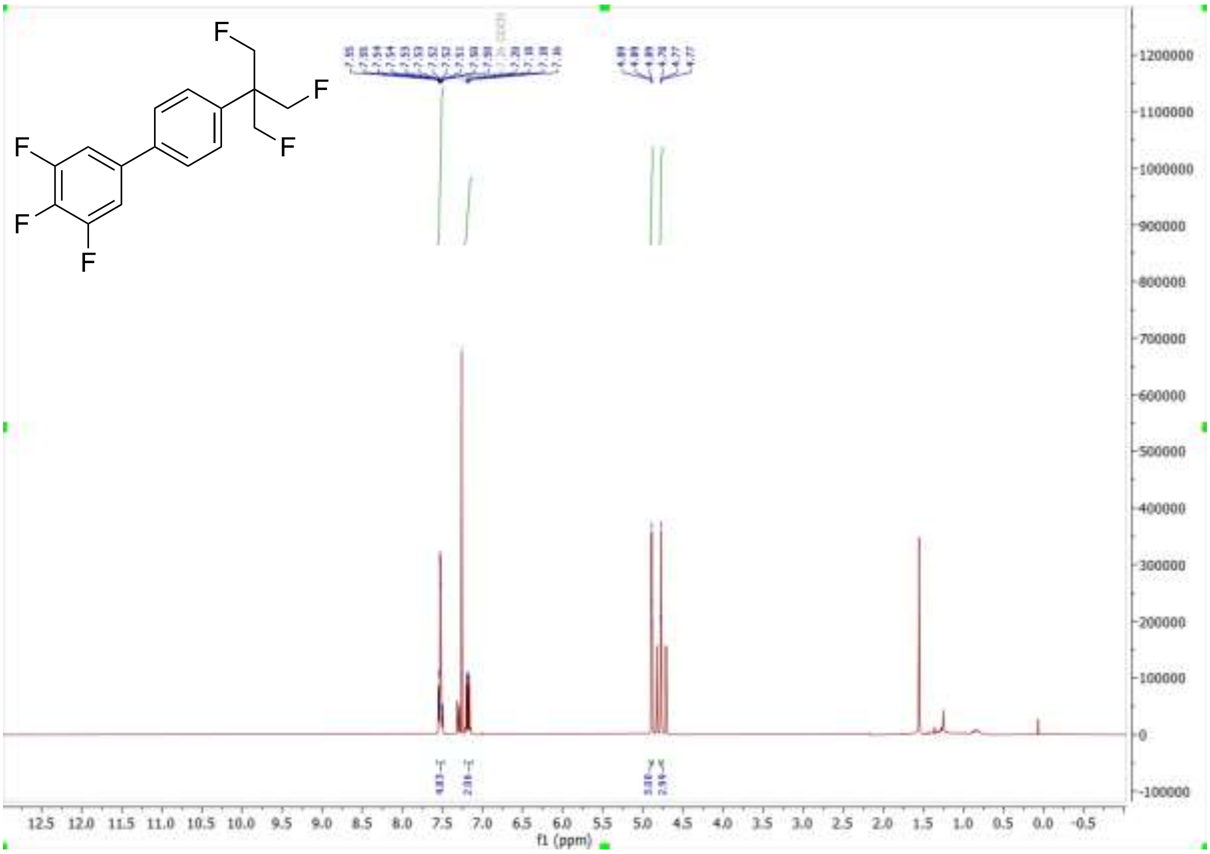

$^{19}F\{^{1}H\}$ NMR (376 MHz, $CDCl_3$):

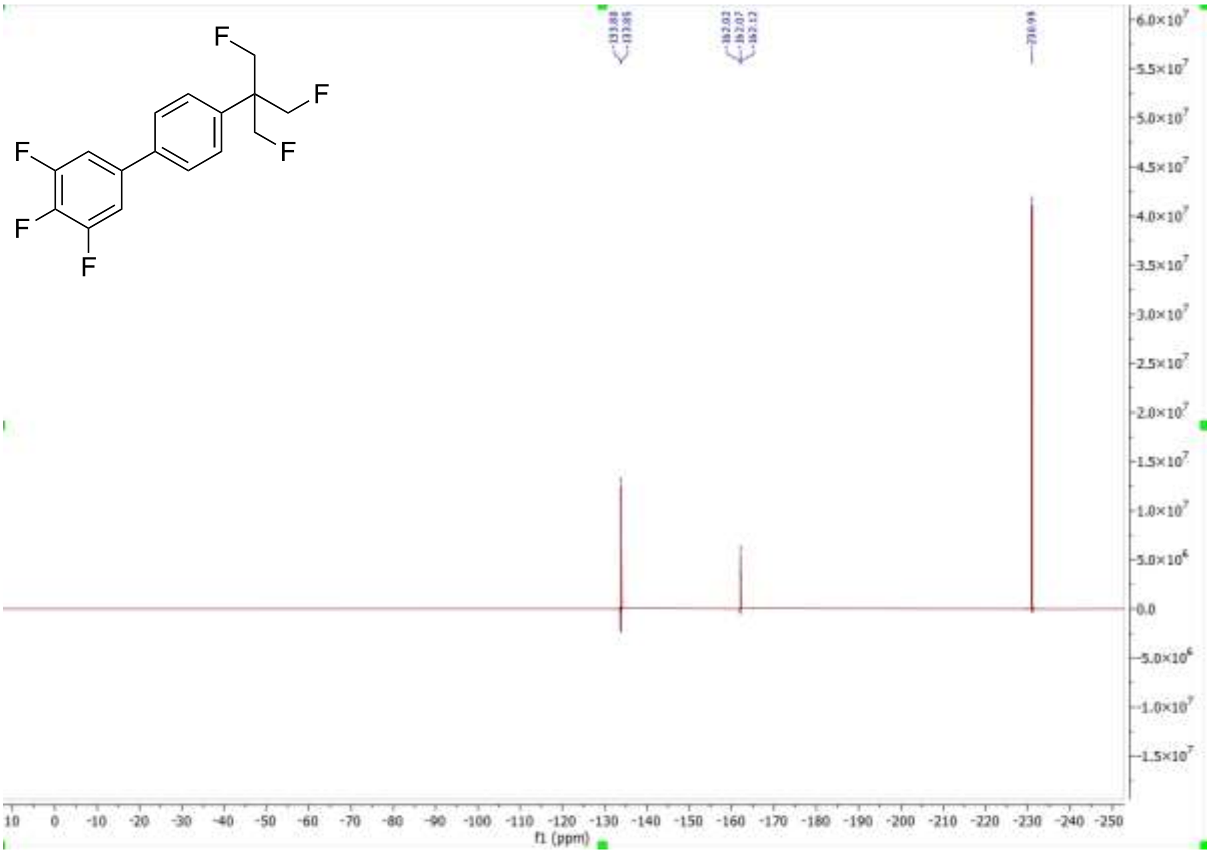

4'-(1,3-Difluoro-2-(fluoromethyl)propan-2-yl)-3-methoxy-1,1'-biphenyl (29)

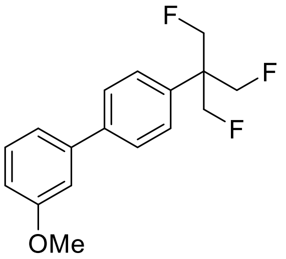

^{1}H NMR (500 MHz, $CDCl_3$):

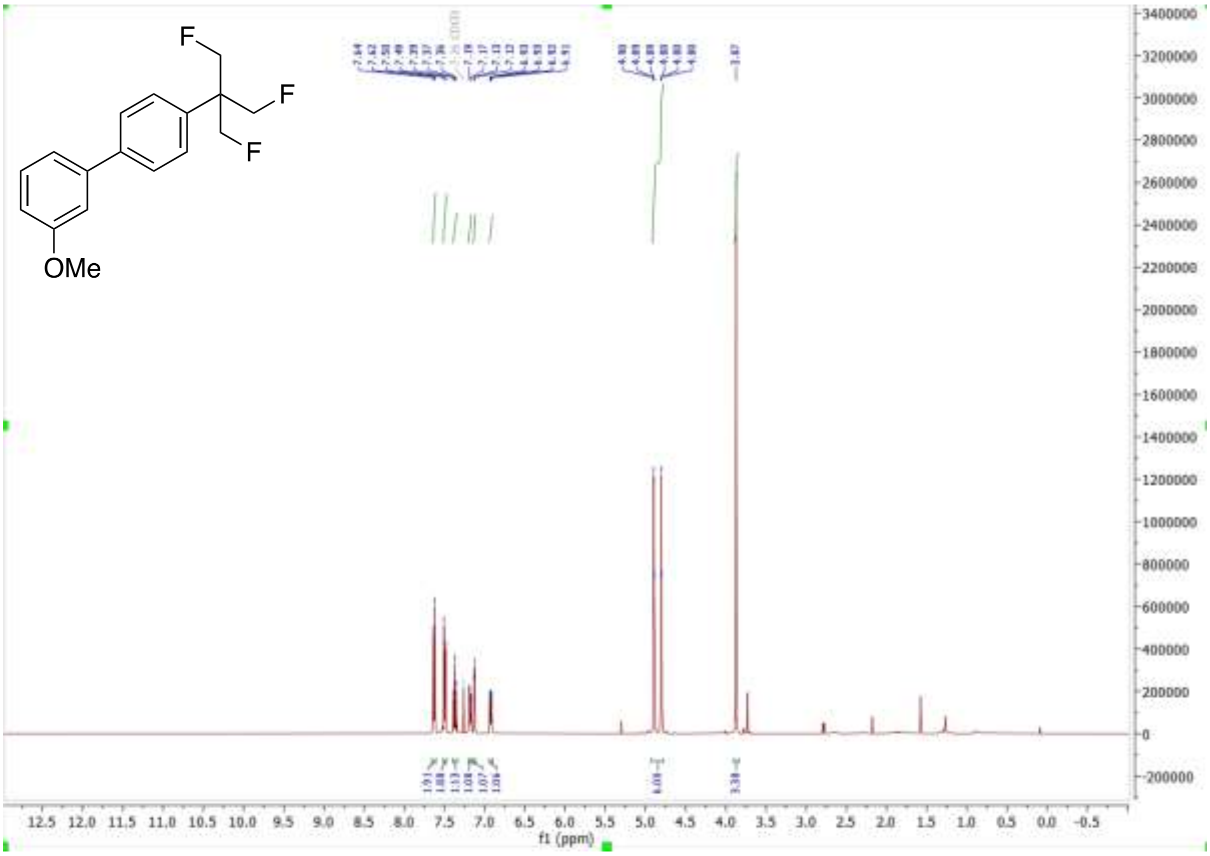

^{13}C NMR (126 MHz, $CDCl_3$):

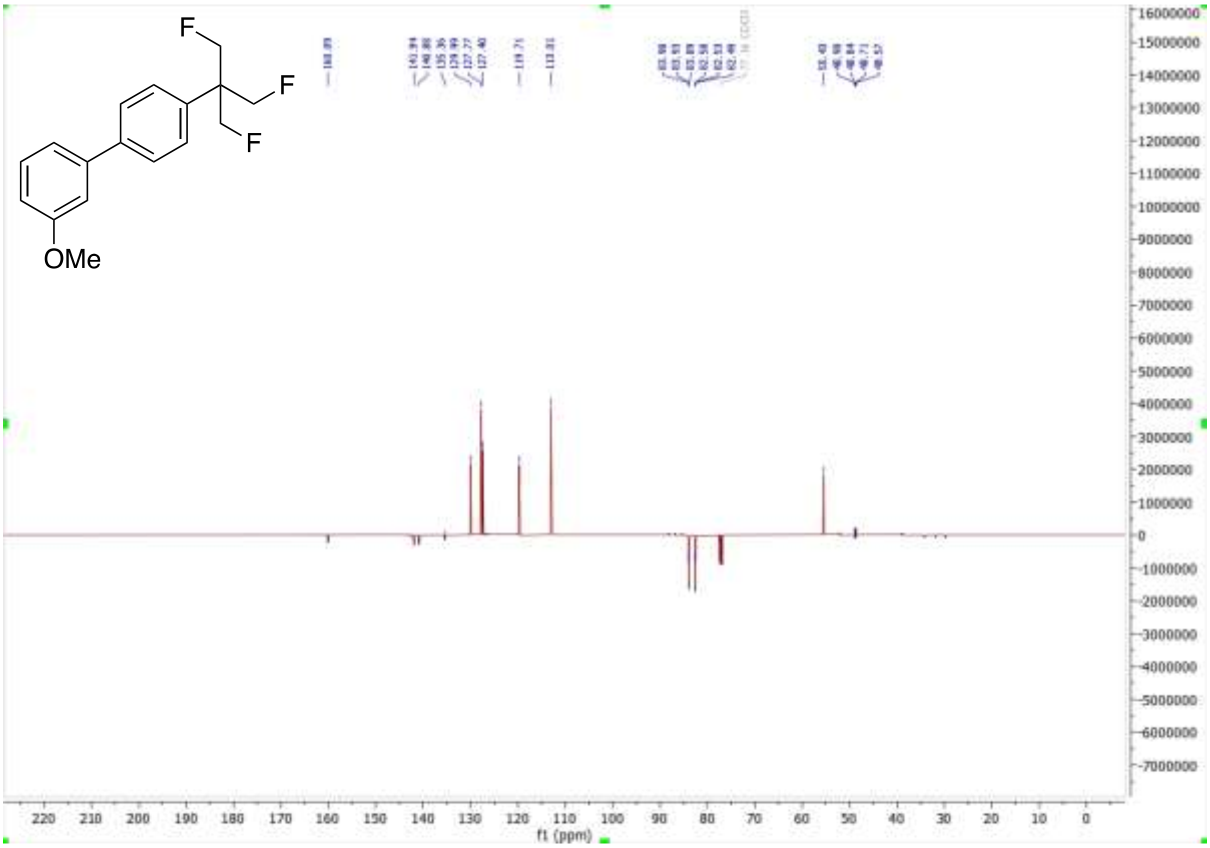

$^{19}F\{^{1}H\}$ NMR (376 MHz, $CDCl_3$):

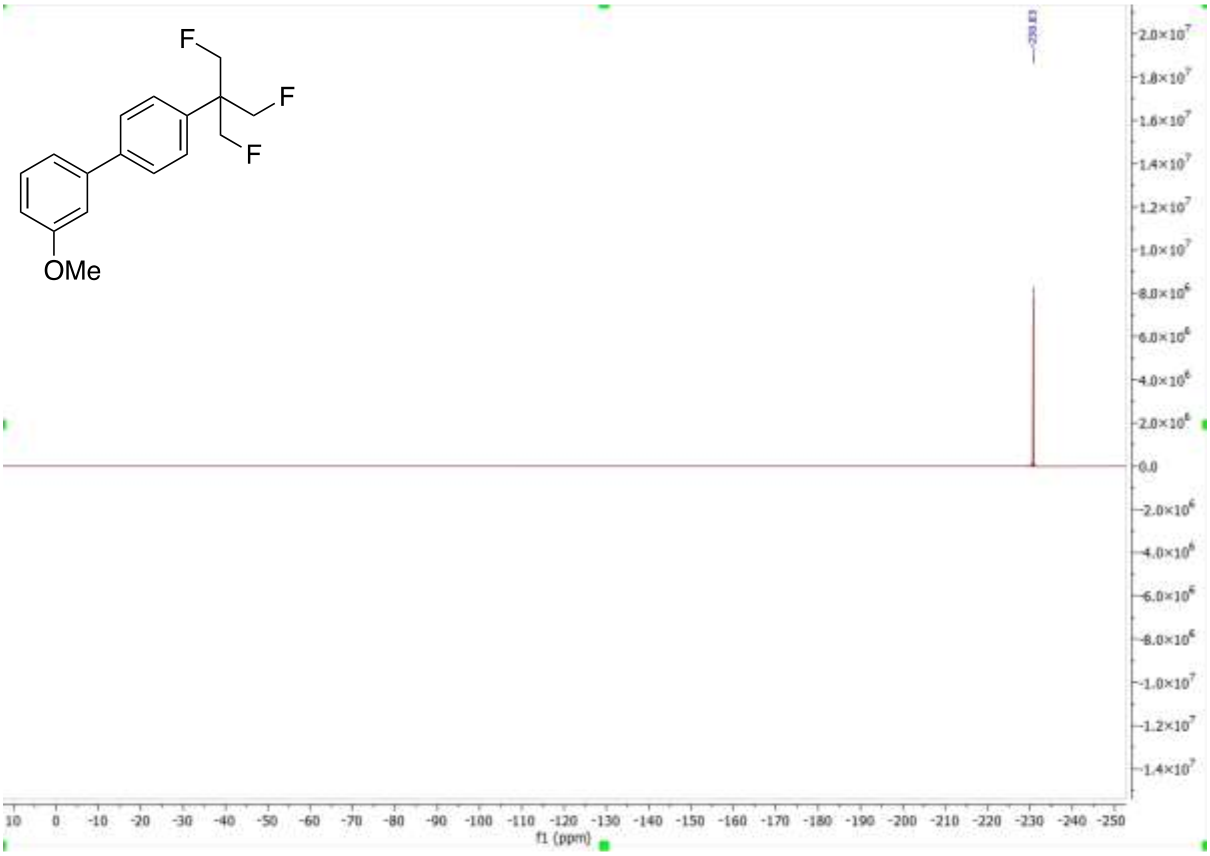

4-(1,3-Difluoro-2-(fluoromethyl)propan-2-yl)-4'-nitro-1,1'-biphenyl (30)

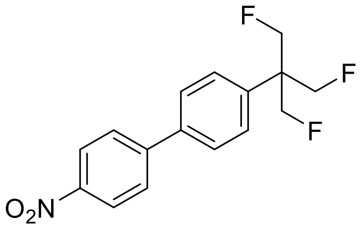

^1H NMR (400 MHz, $CDCl_3$):

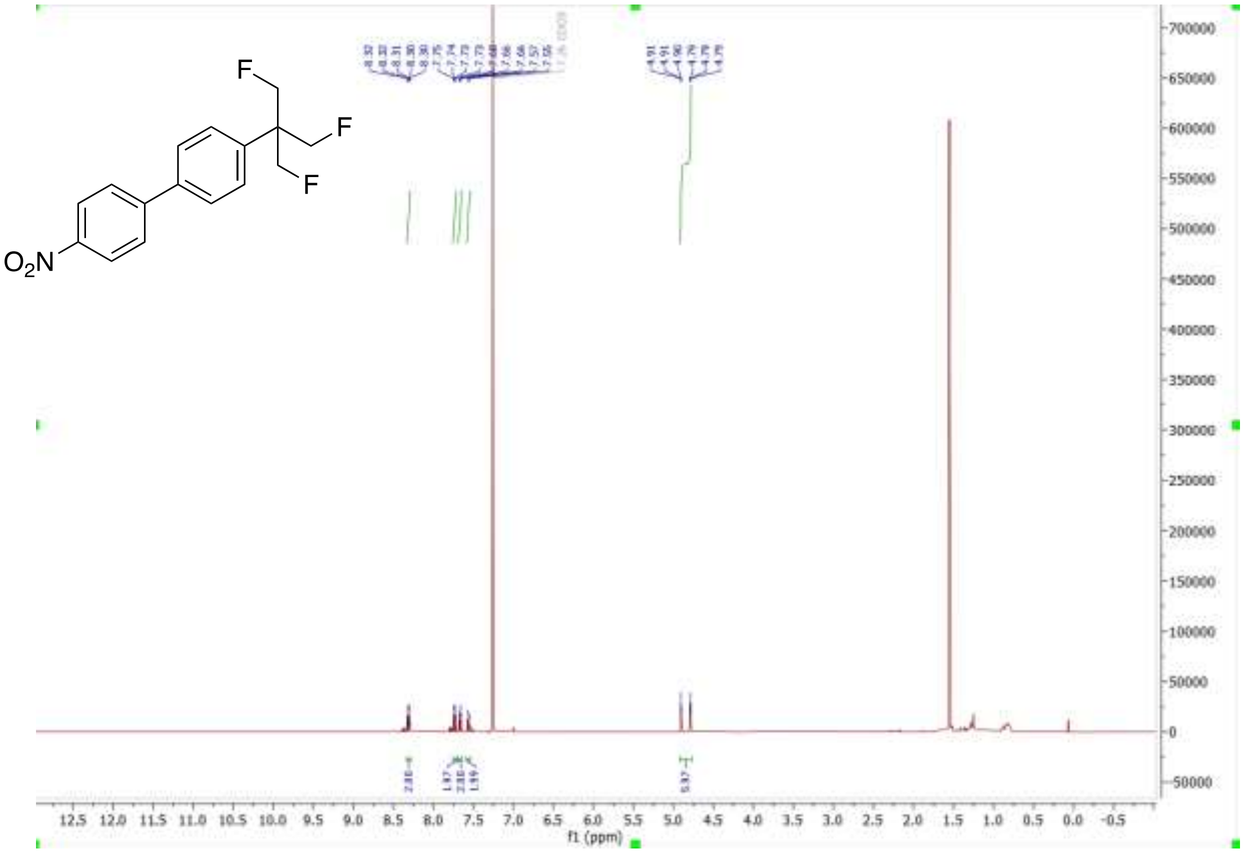

$^{19}F\{^{1}H\}$ NMR (376 MHz, $CDCl_3$):

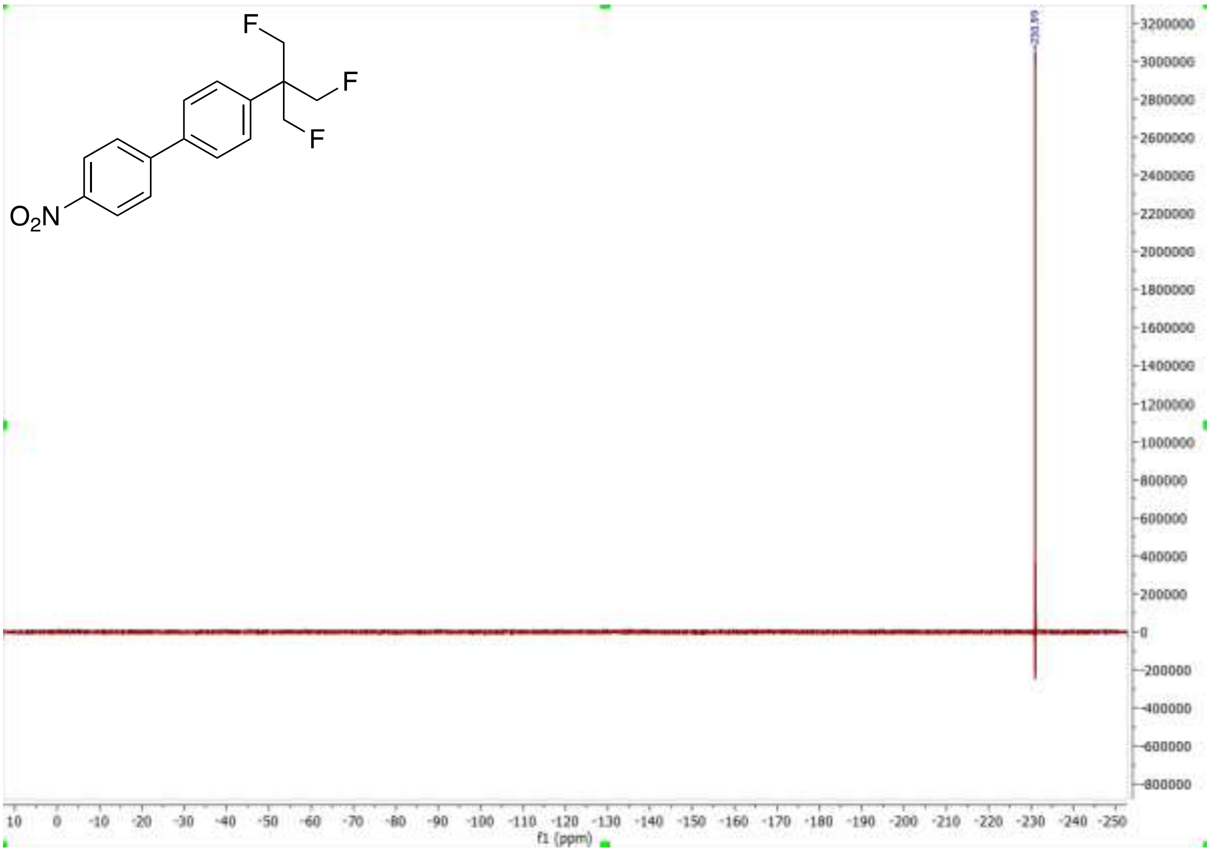

^{13}C NMR (126 MHz, $CDCl_3$):

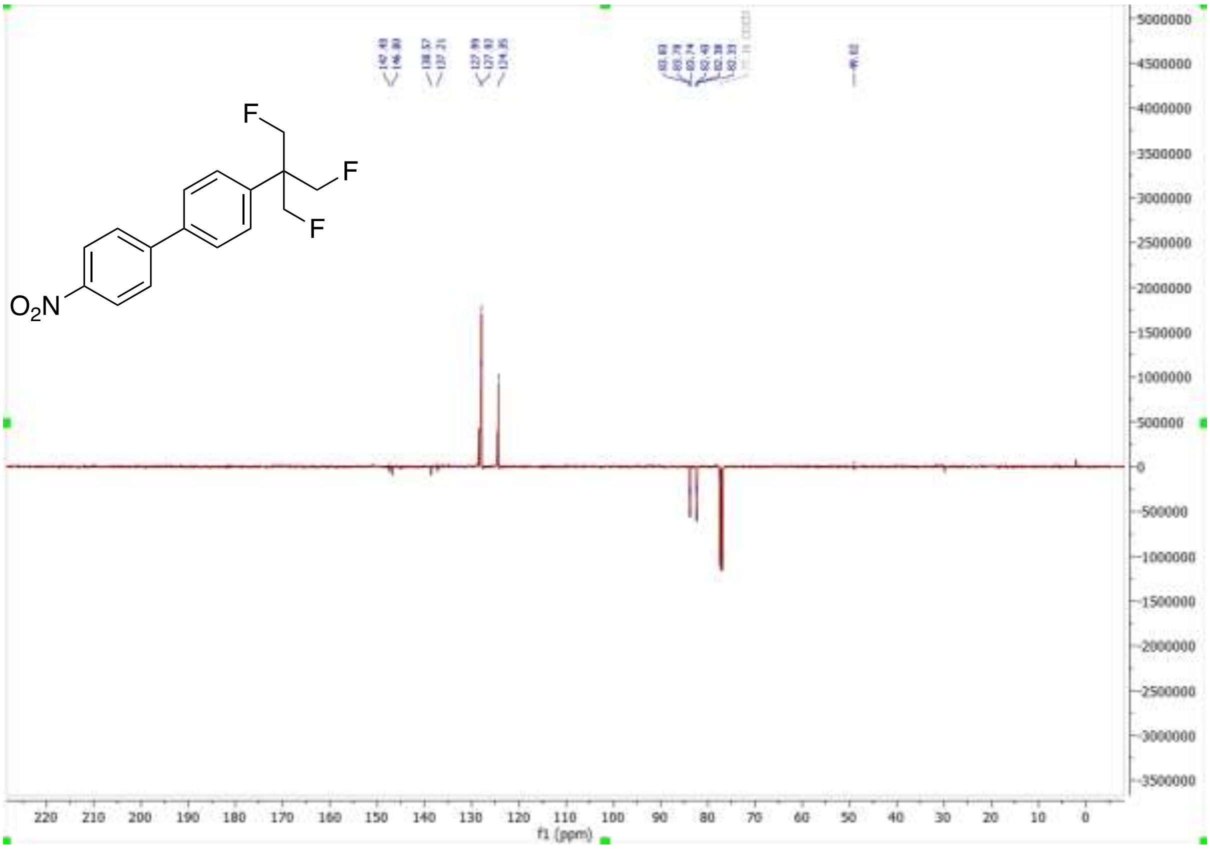

4'-(1,3-Difluoro-2-(fluoromethyl)propan-2-yl)-2-methyl-1,1'-biphenyl (31)

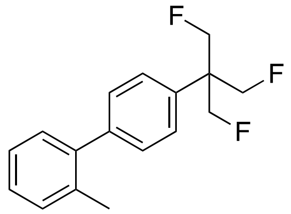

^{1}H NMR (500 MHz, $CDCl_3$):

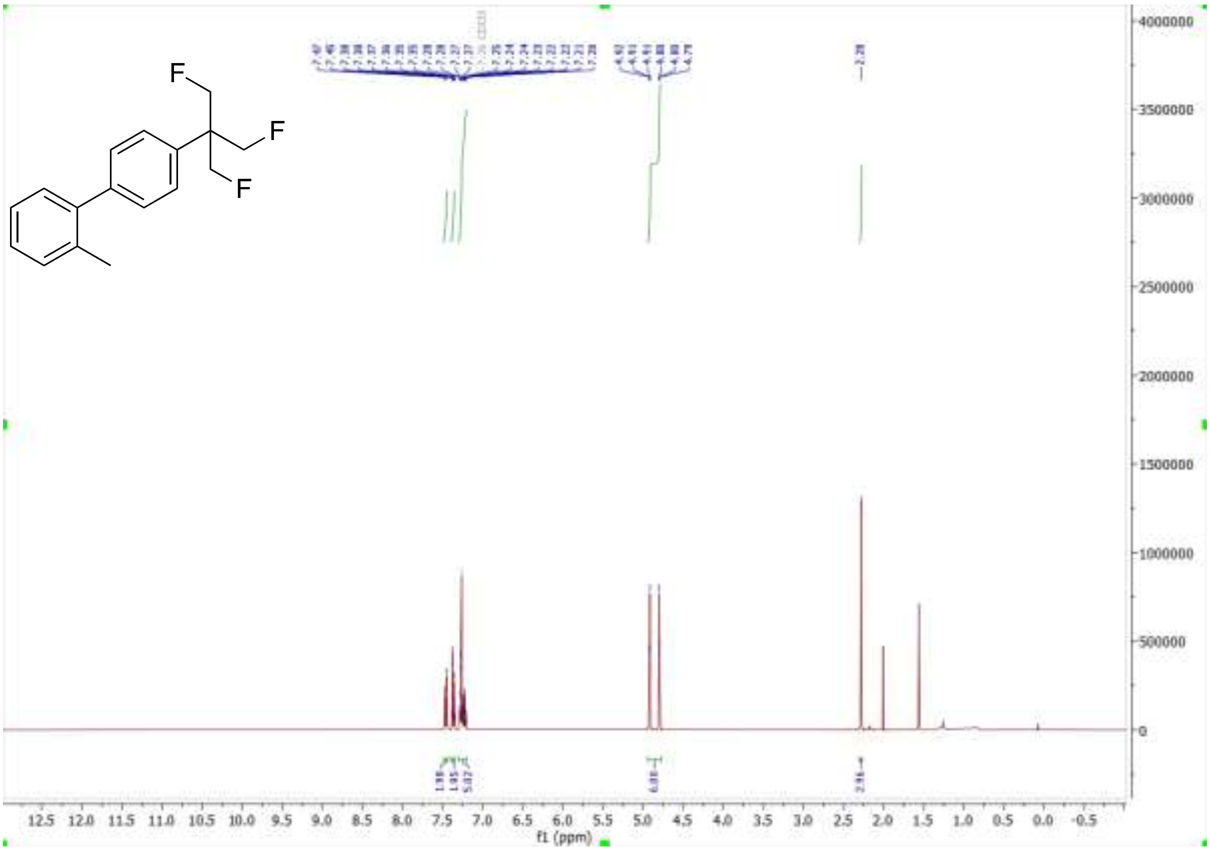

$^{19}F\{^{1}H\}$ NMR (376 MHz, $CDCl_3$):

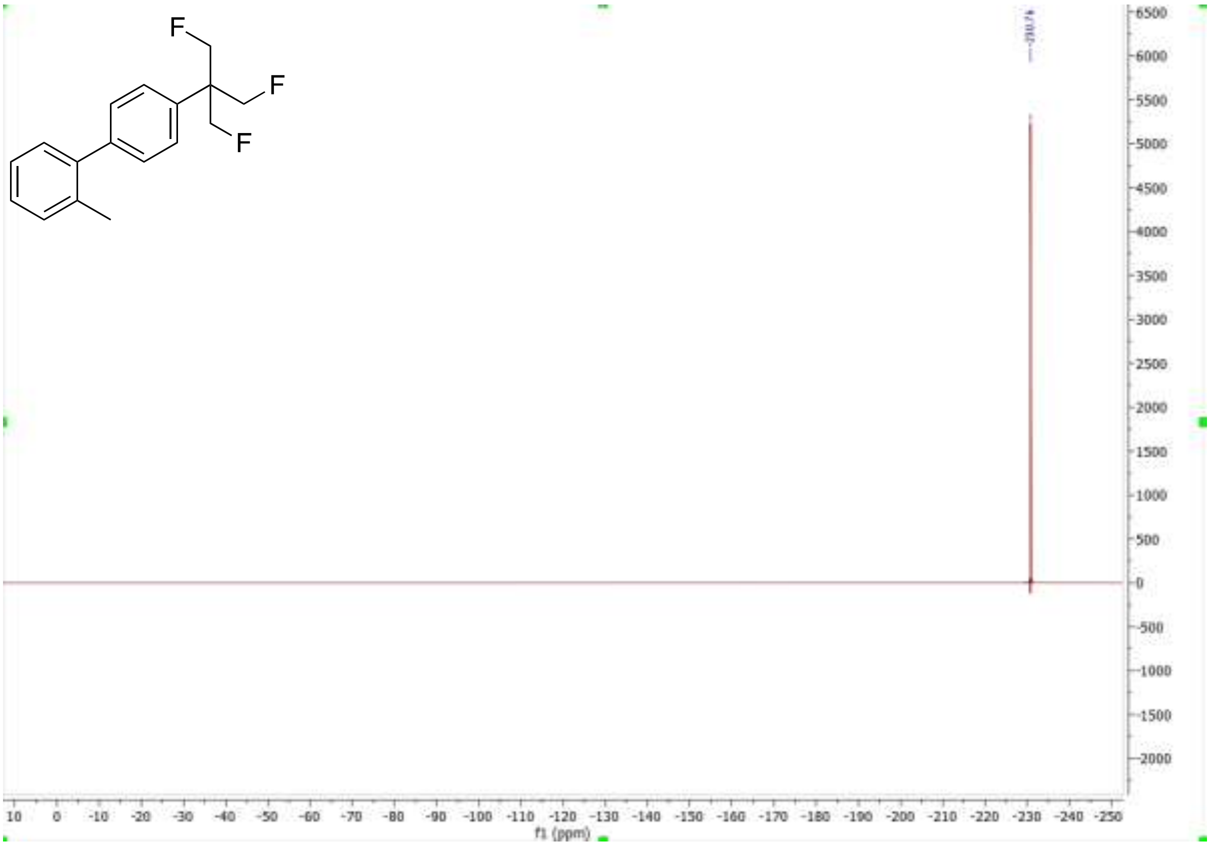

^{13}C NMR (126 MHz, $CDCl_3$):

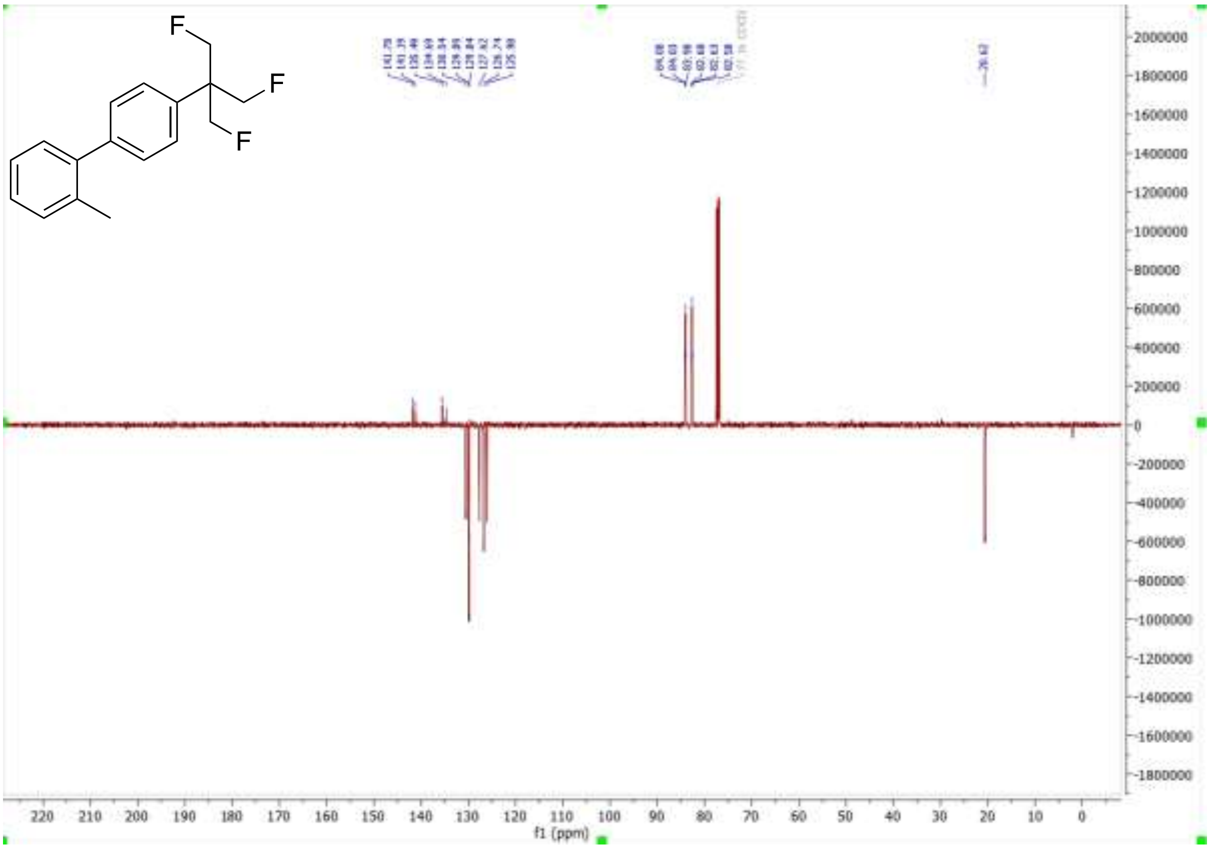

1-(4-(1,3-Difluoro-2-(fluoromethyl)propan-2-yl)phenyl)piperidine (32)

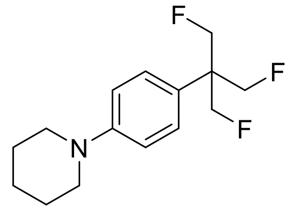

^{1}H NMR (500 MHz, $CDCl_3$):

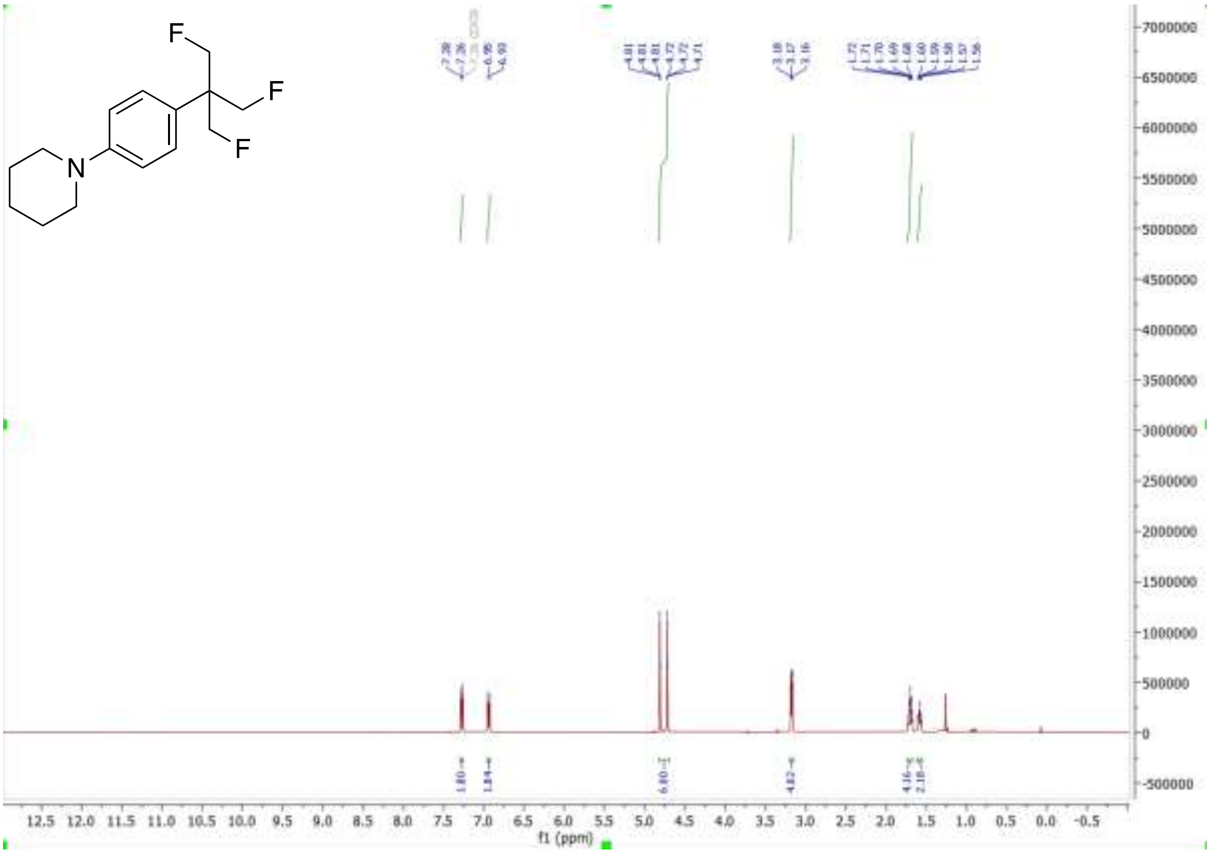

$^{19}F\{^{1}H\}$ NMR (376 MHz, $CDCl_3$):

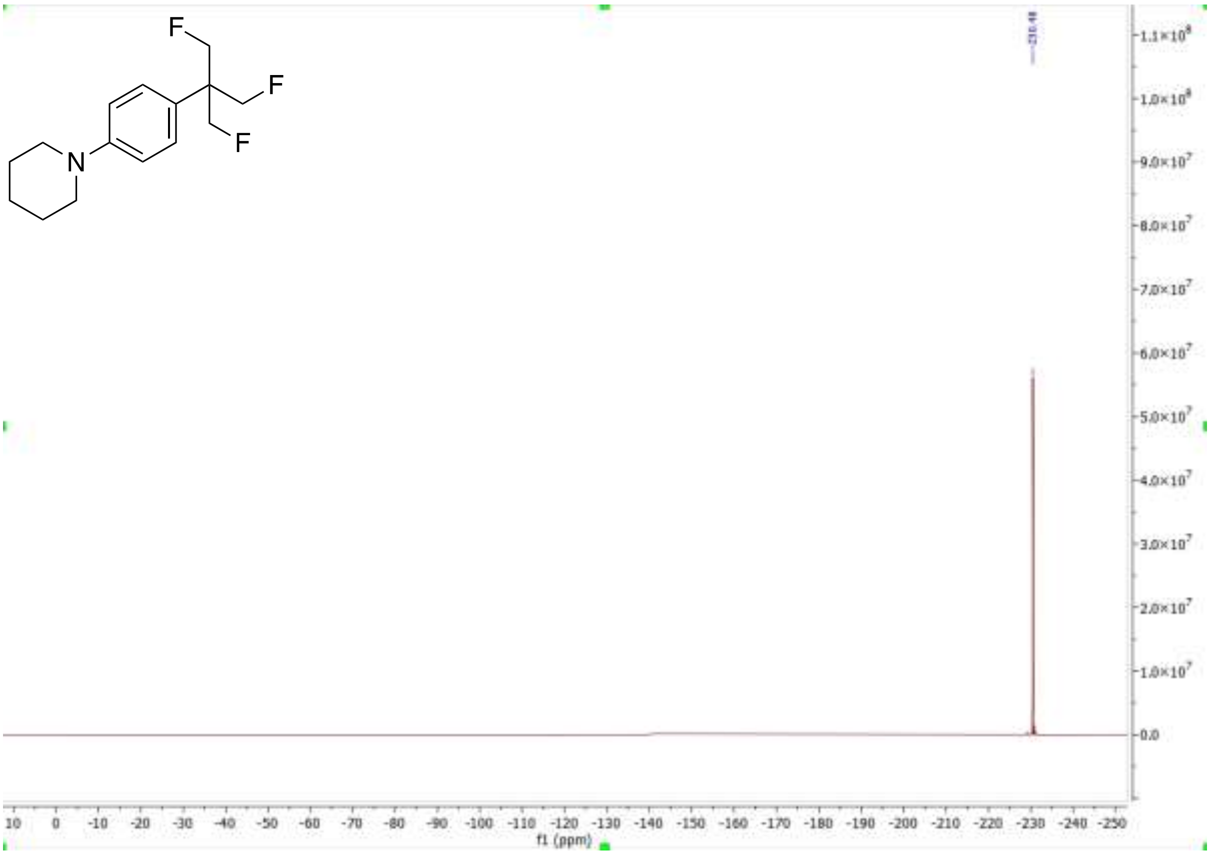

^{13}C NMR (126 MHz, $CDCl_3$):

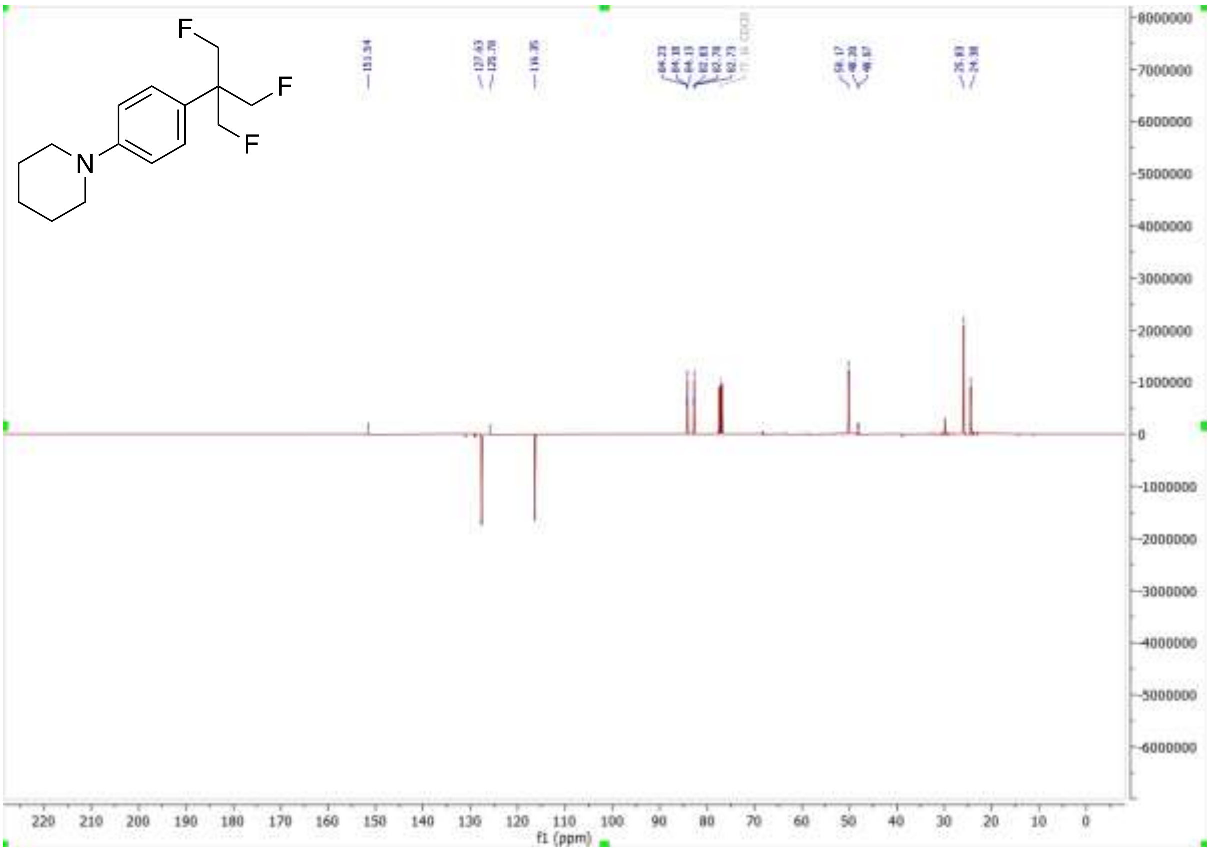

4-(4-(1,3-Difluoro-2-(fluoromethyl)propan-2-yl)phenyl)morpholine (33)

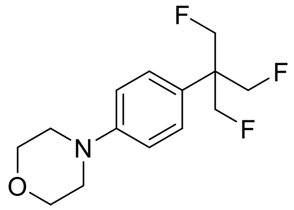

^{1}H NMR (500 MHz, CDCl$_3$):

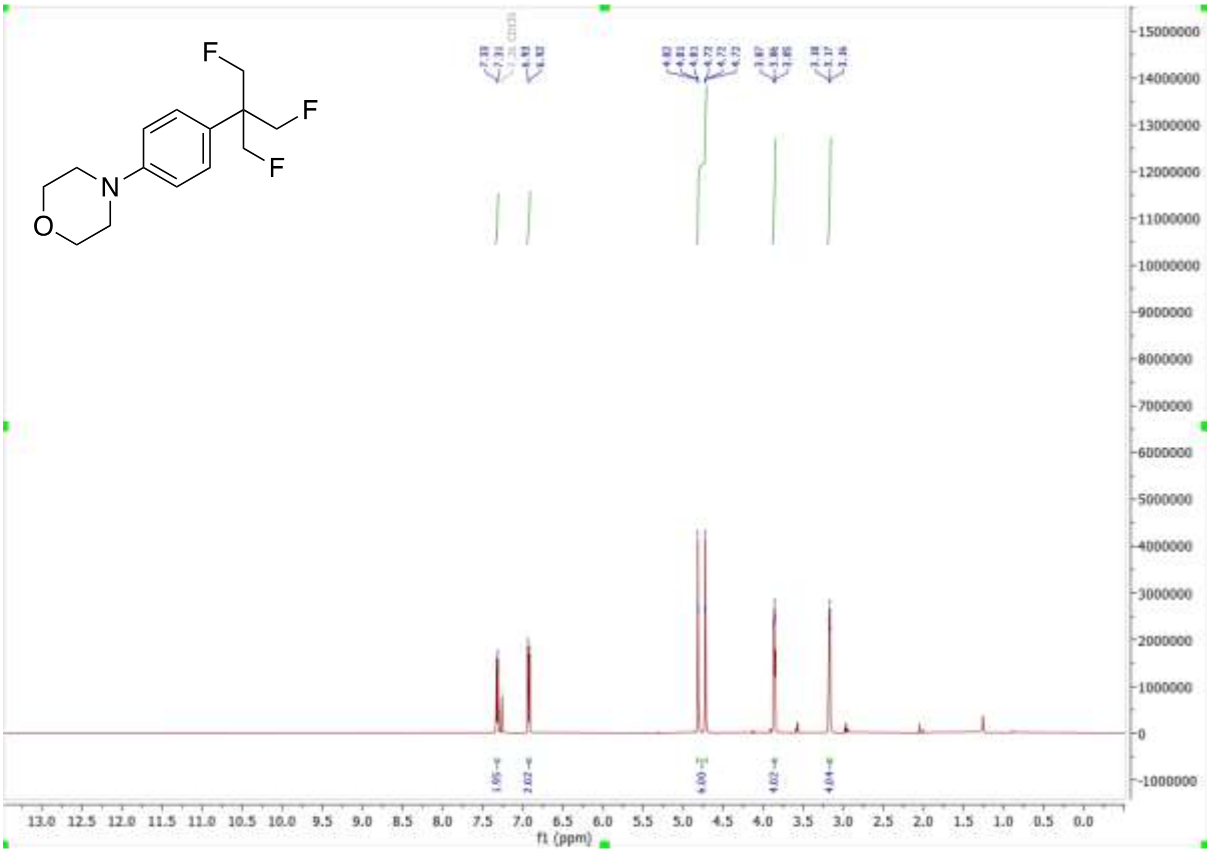

$^{19}F\{^{1}H\}$ NMR (376 MHz, $CDCl_3$):

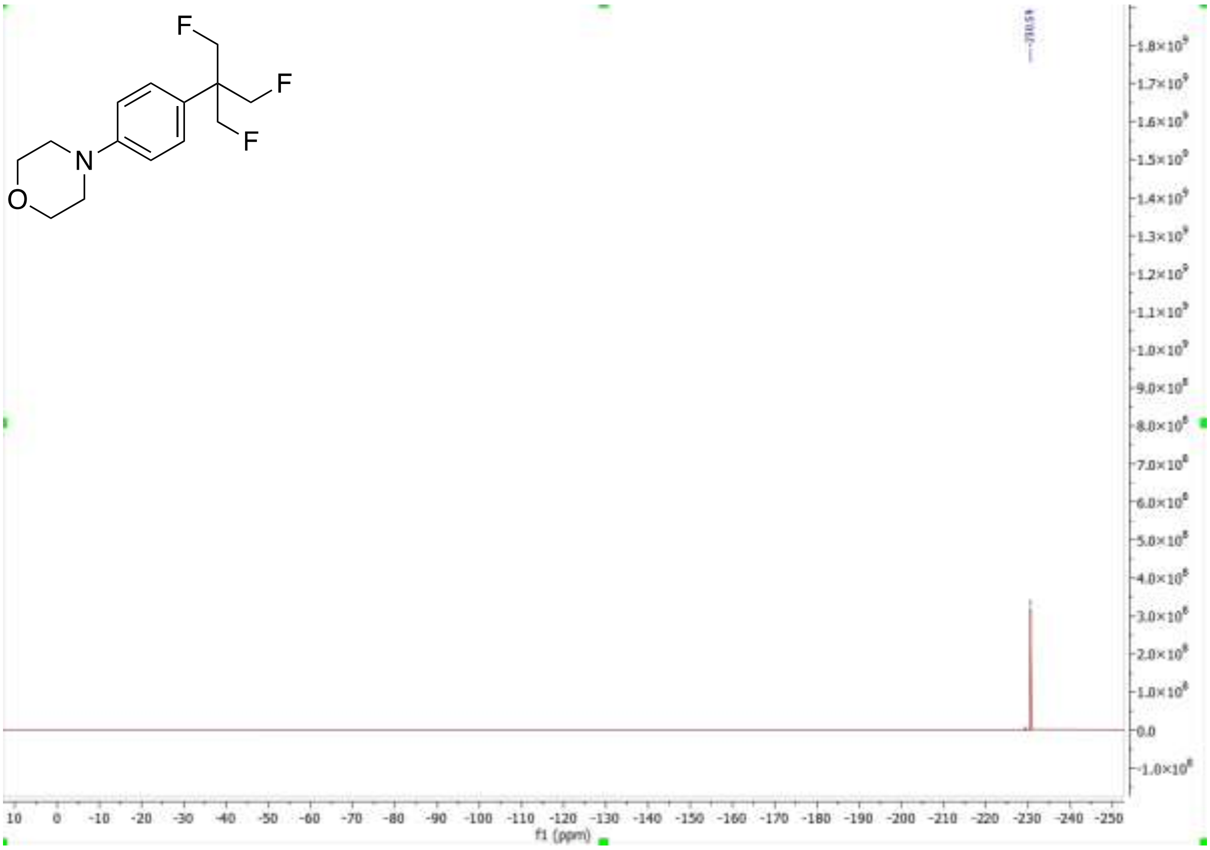

^{13}C NMR (126 MHz, $CDCl_3$):

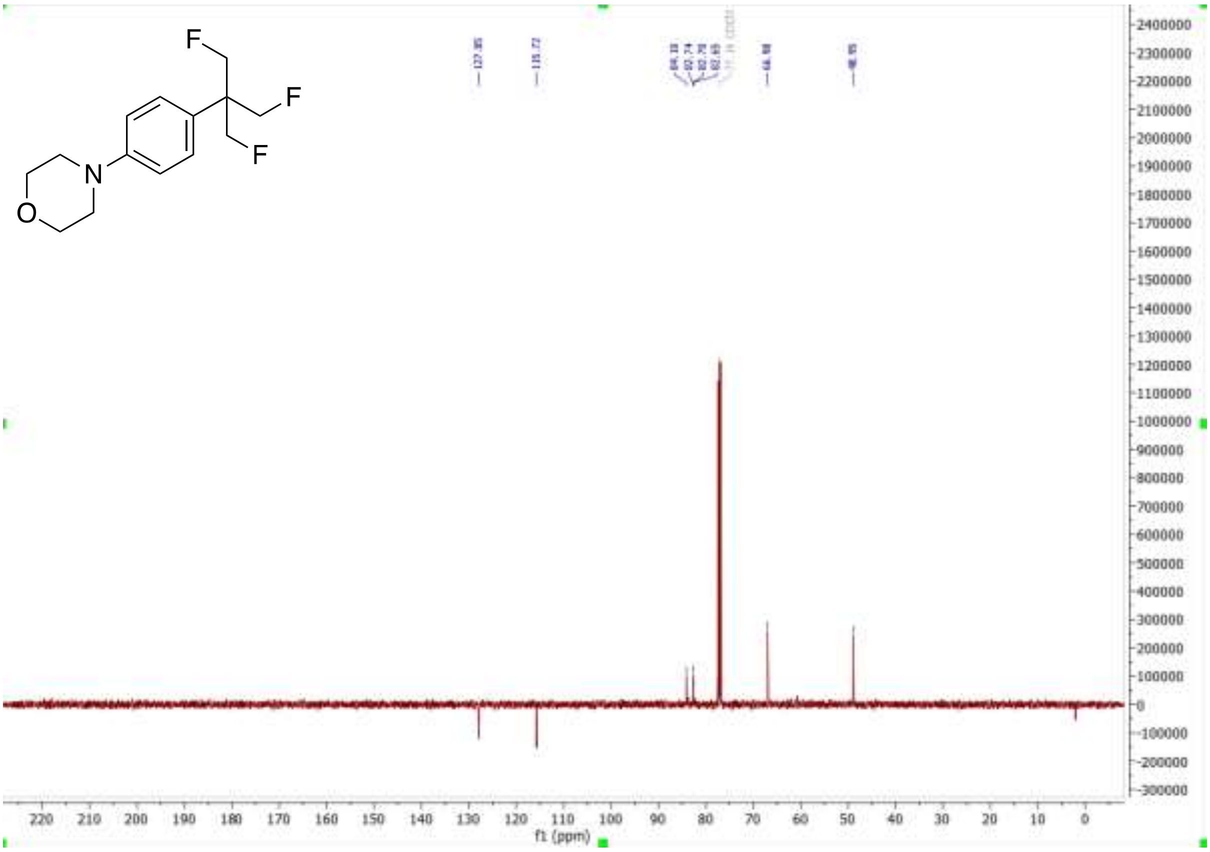

4-(1,3-Difluoro-2-(fluoromethyl)propan-2-yl)-N-phenylaniline (34)

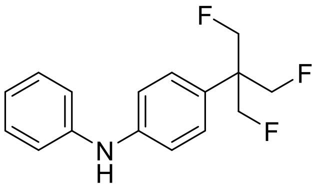

^{1}H NMR (500 MHz, $CDCl_3$):

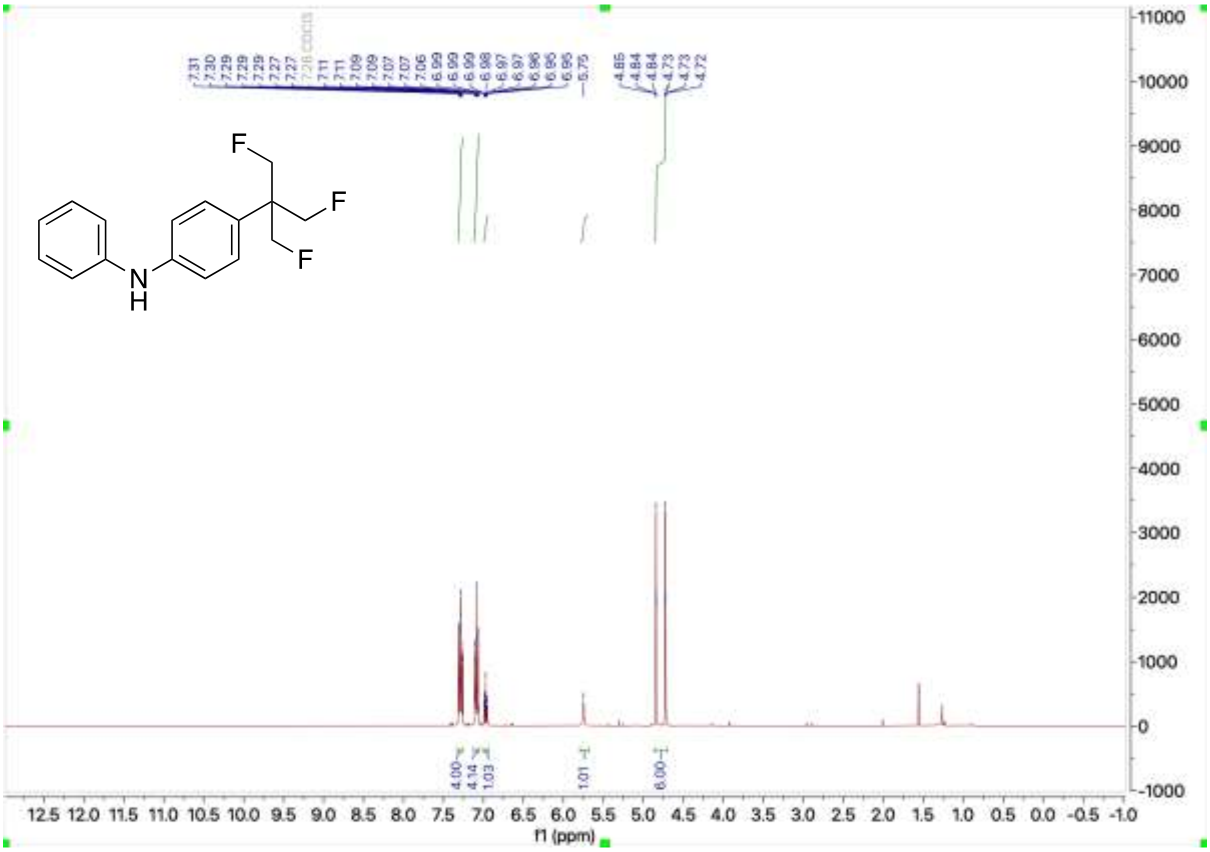

$^{19}F\{^{1}H\}$ NMR (376 MHz, $CDCl_3$):

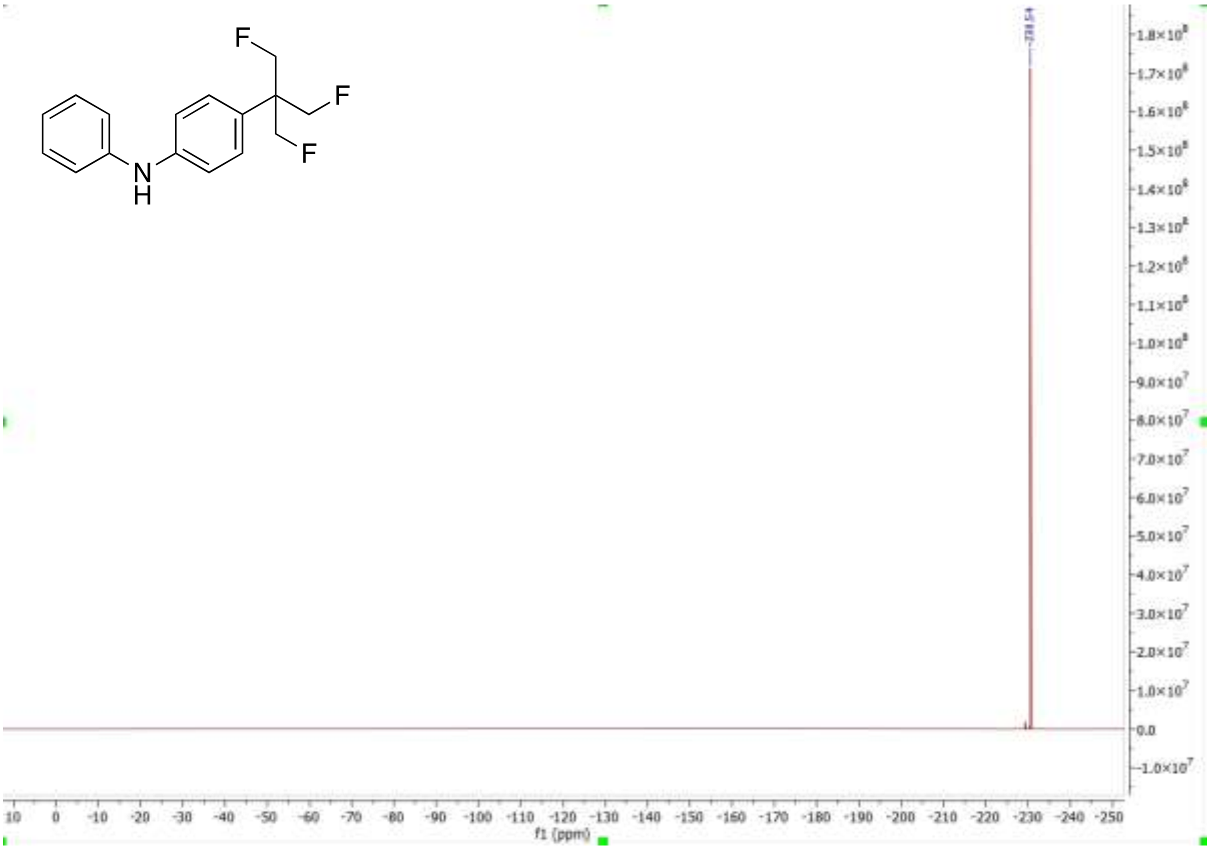

^{13}C NMR (126 MHz, Acetone-d^6):

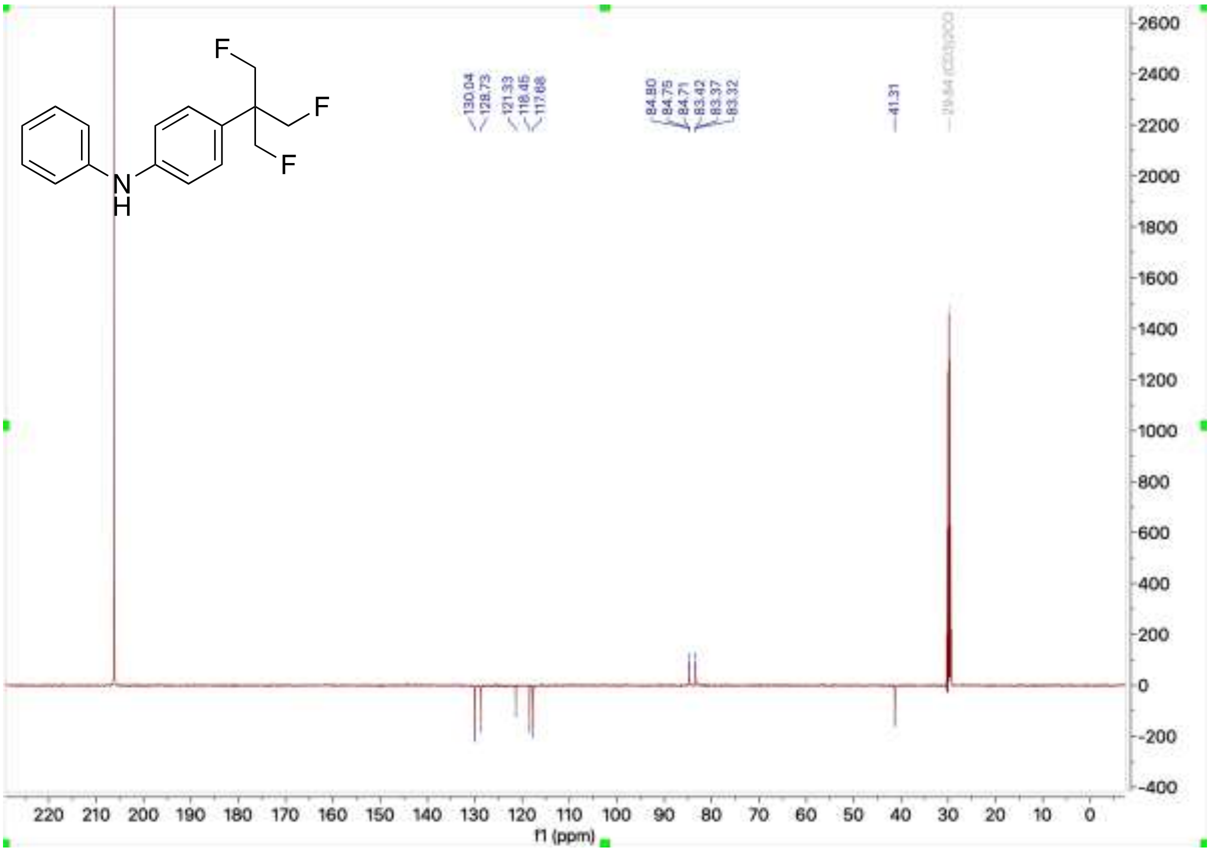

(*R*)-4-(1,3-Difluoro-2-(fluoromethyl)propan-2-yl)-N-(1-(naphthalen-1-yl)ethyl)aniline (35)

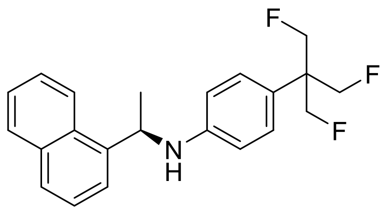

^{1}H NMR (500 MHz, $CDCl_3$):

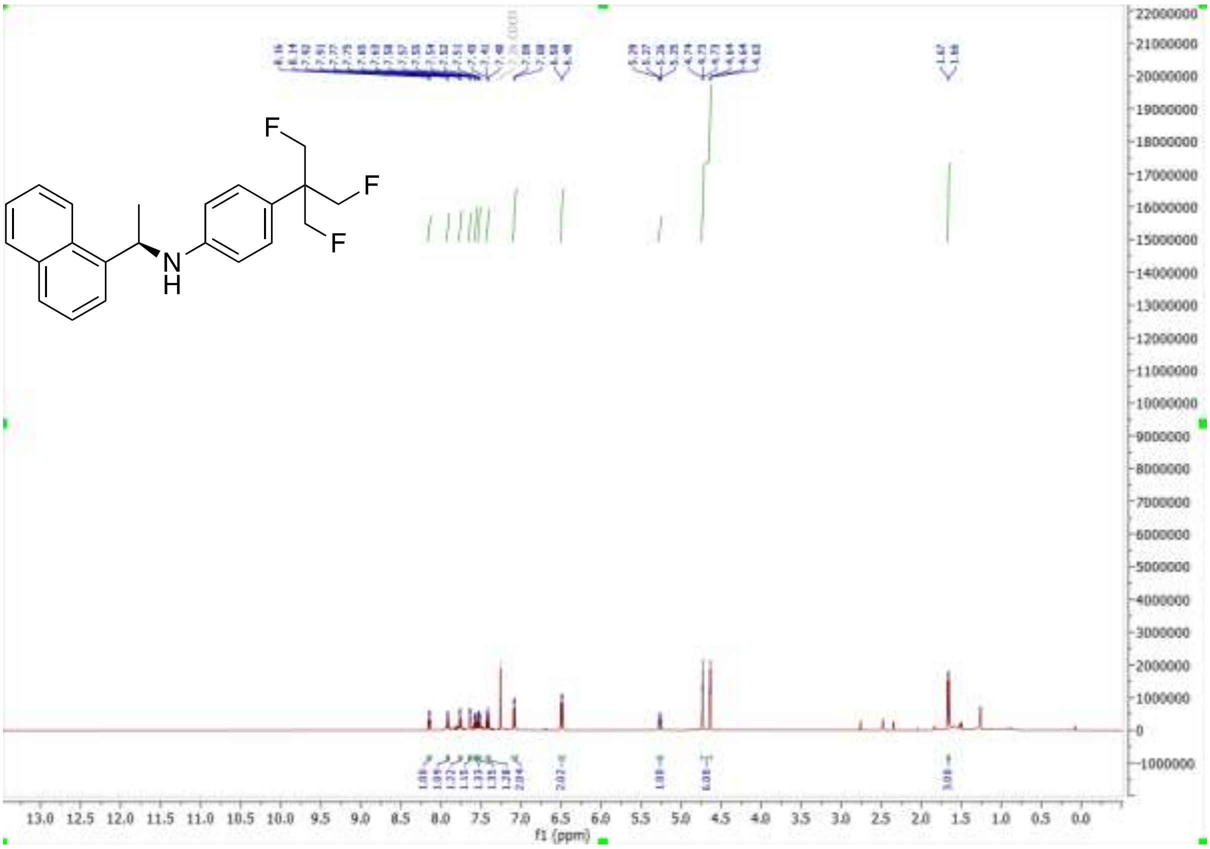

$^{19}F\{^{1}H\}$ NMR (376 MHz, $CDCl_3$):

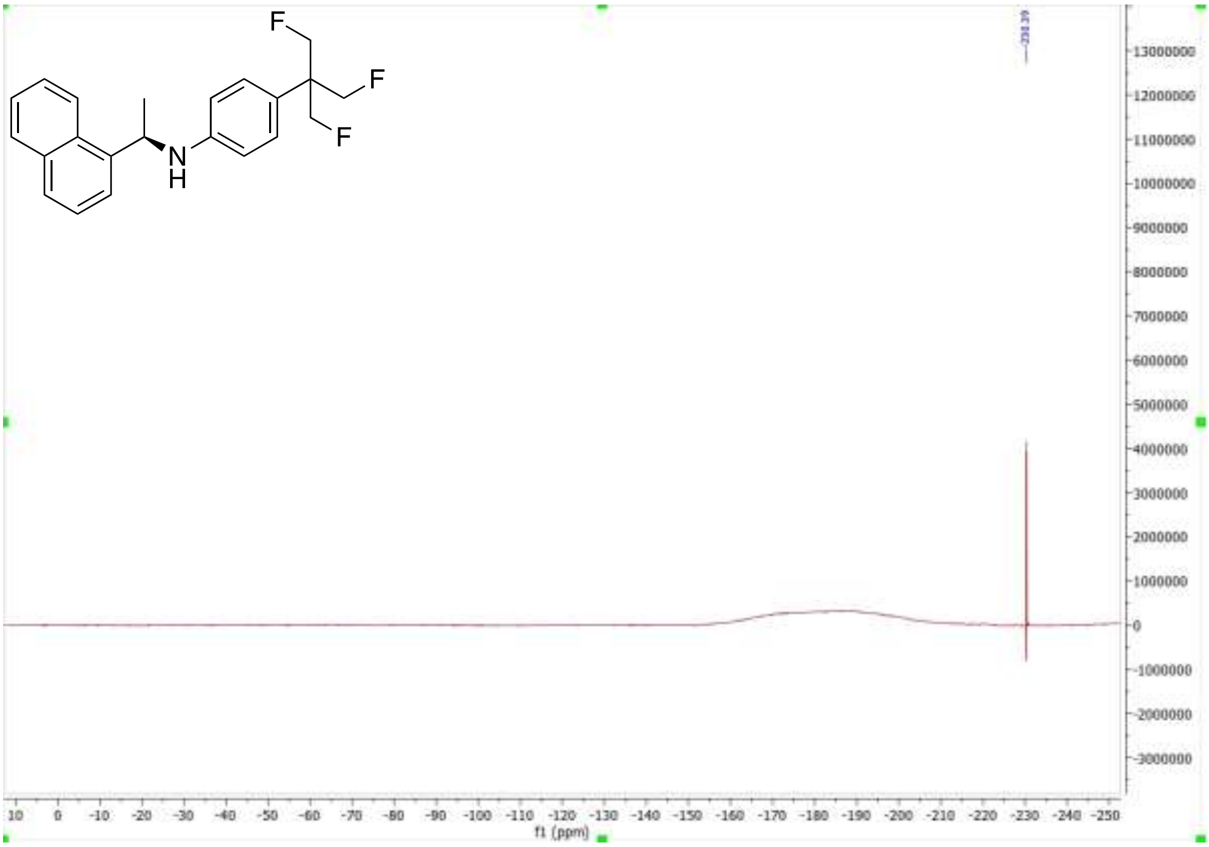

^{13}C NMR (126 MHz, $CDCl_3$):

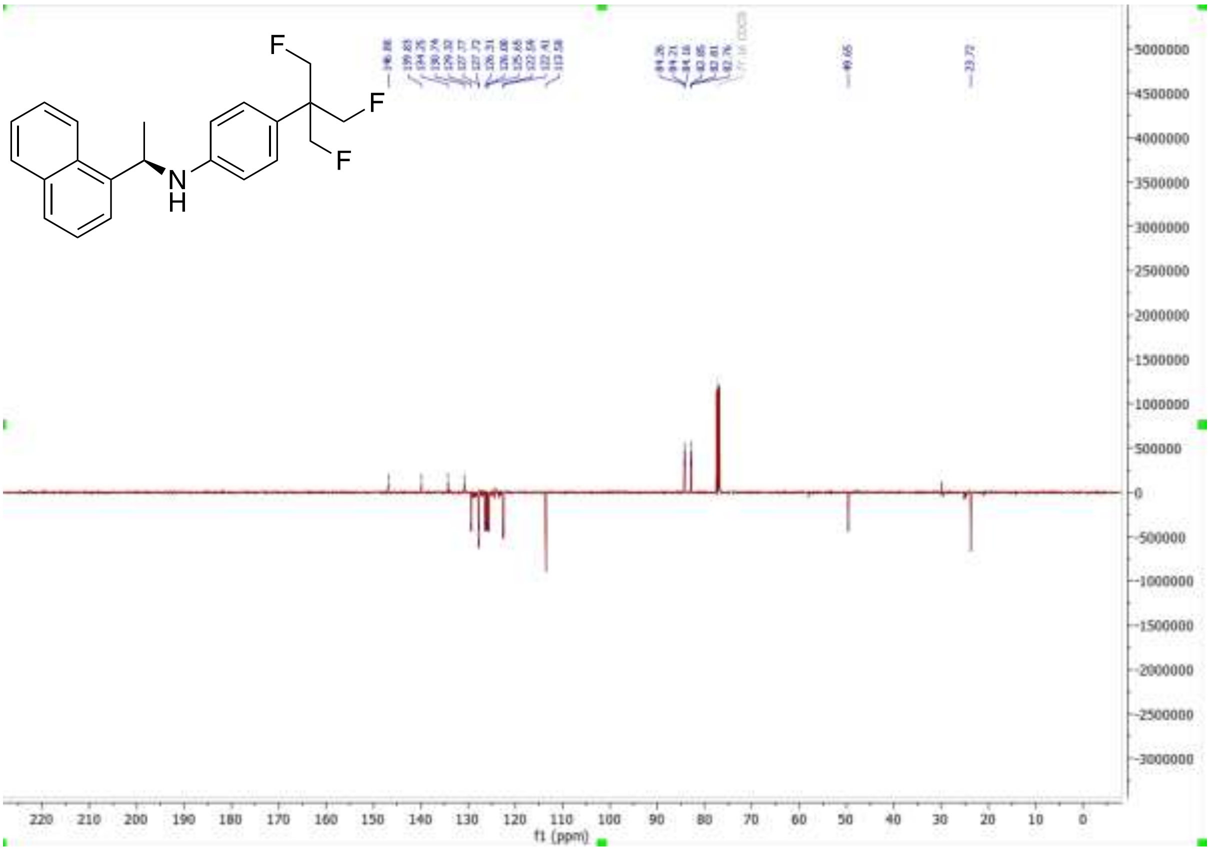

2-(p-Tolyl)acetaldehyde (43)

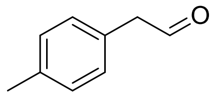

^{1}H NMR (400 MHz, $CDCl_3$):

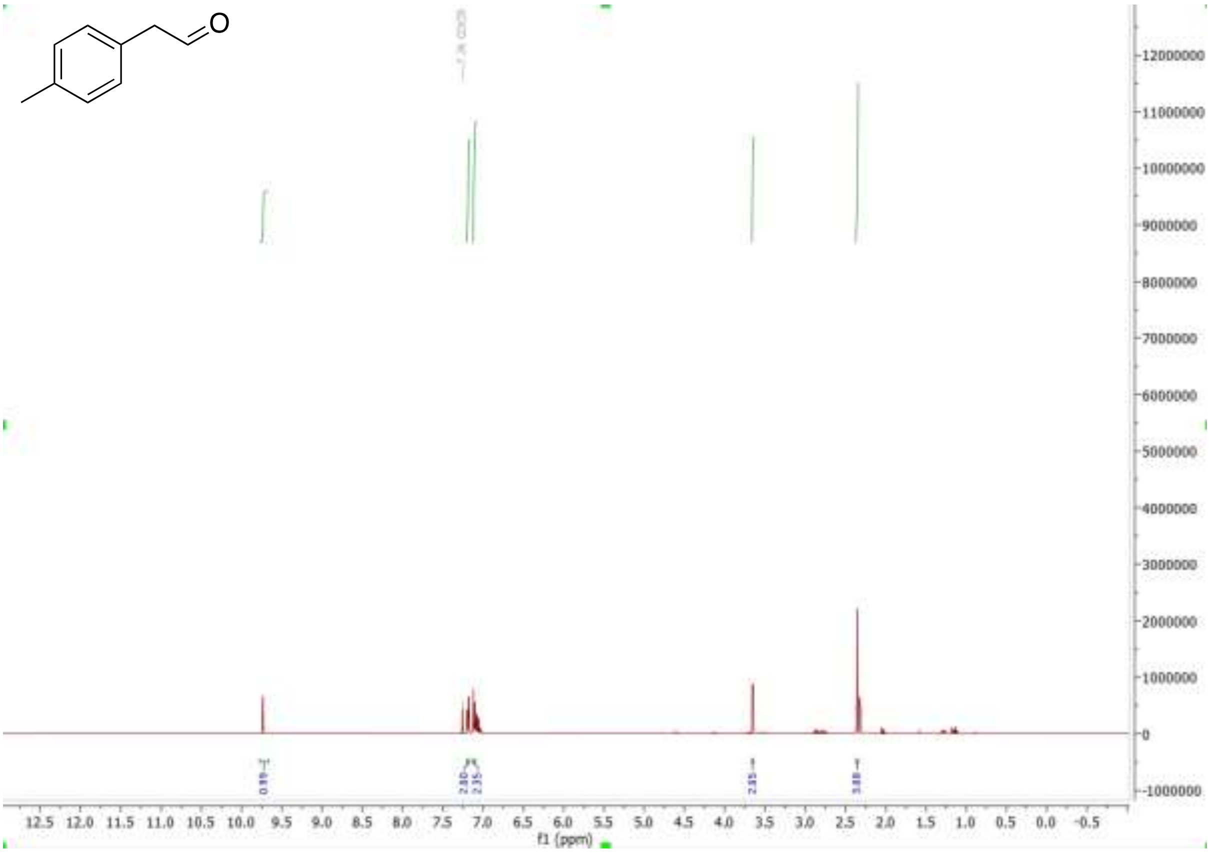

2-(Hydroxymethyl)-2-(p-tolyl)propane-1,3-diol (44)

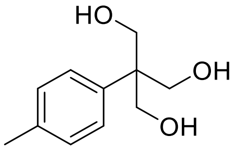

^{1}H NMR (500 MHz, d^6-DMSO):

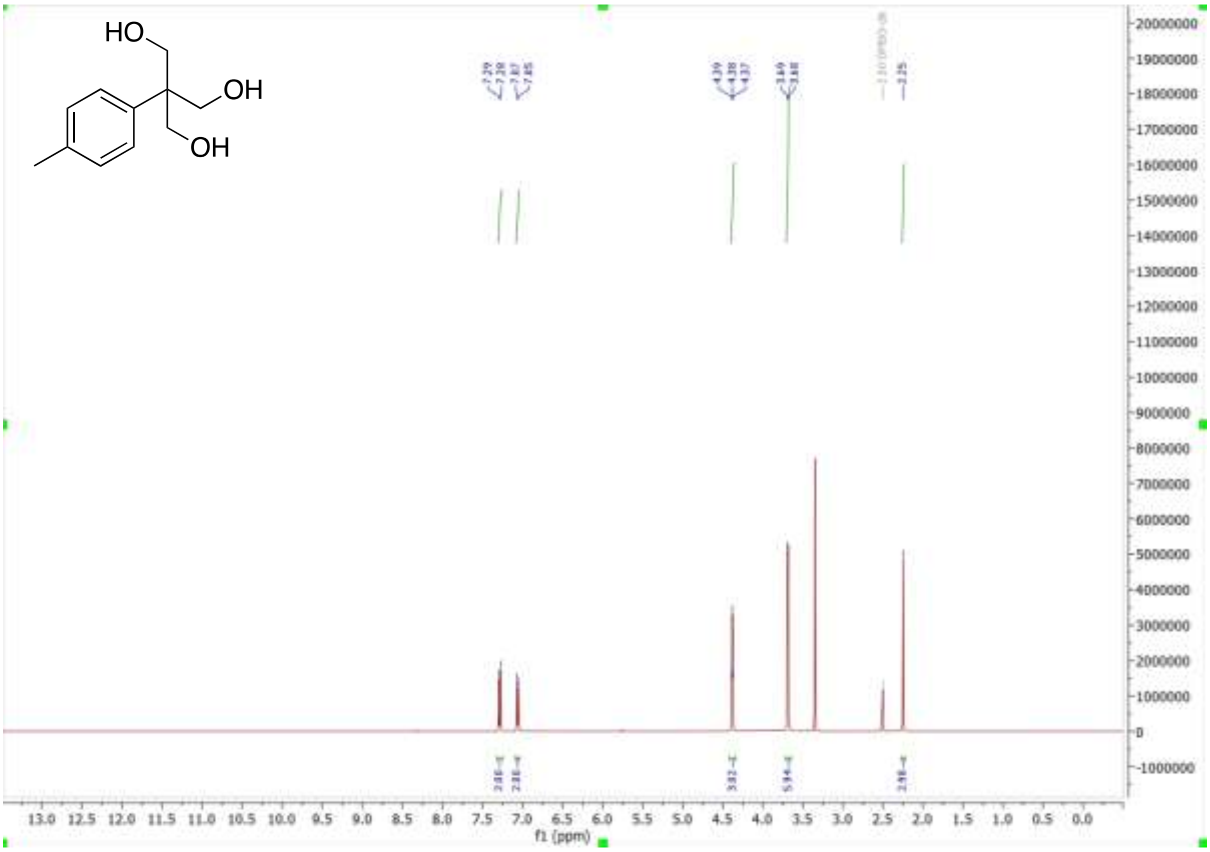

^{13}C NMR (126 MHz, d^6-DMSO):

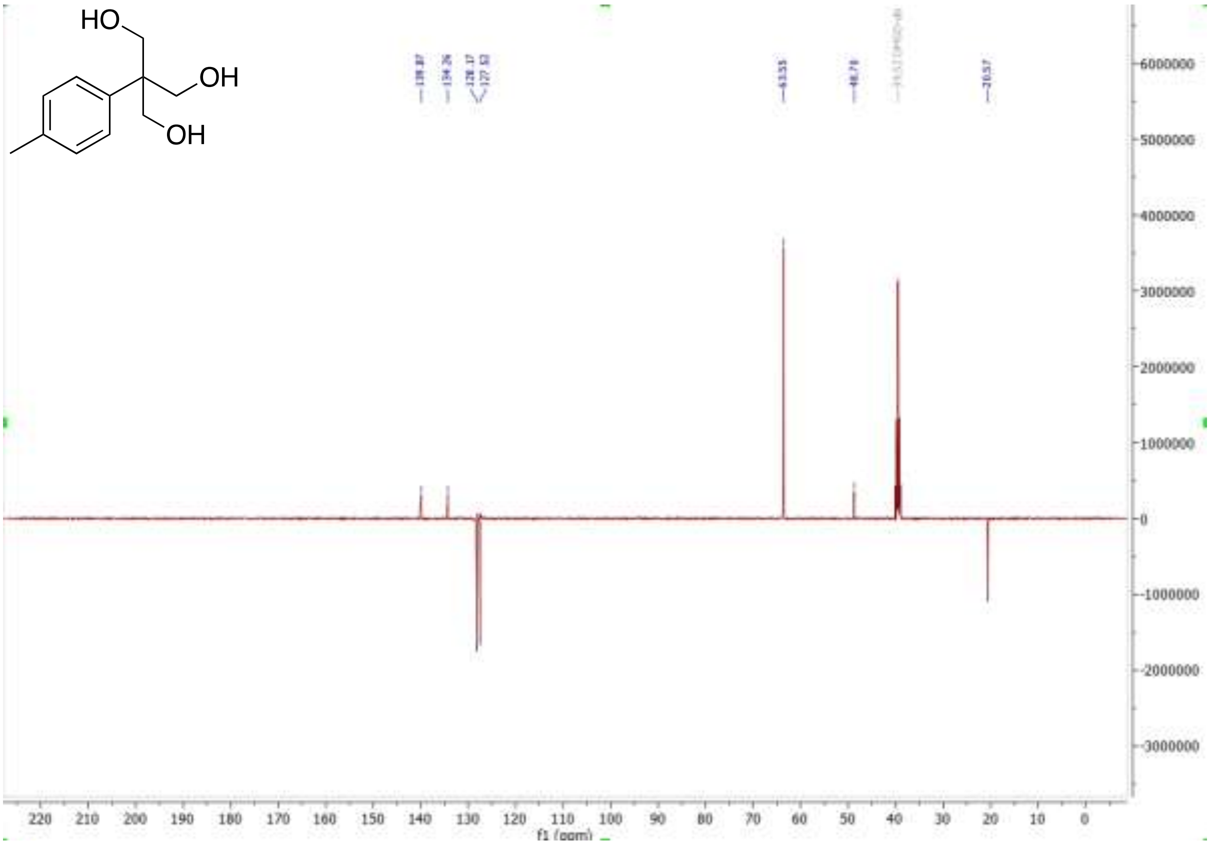

2-(p-Tolyl)-2-((tosyloxy)methyl)propane-1,3-diyl bis(4-methylbenzenesulfonate) (45)

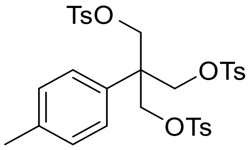

^{1}H NMR (500 MHz, $CDCl_3$):

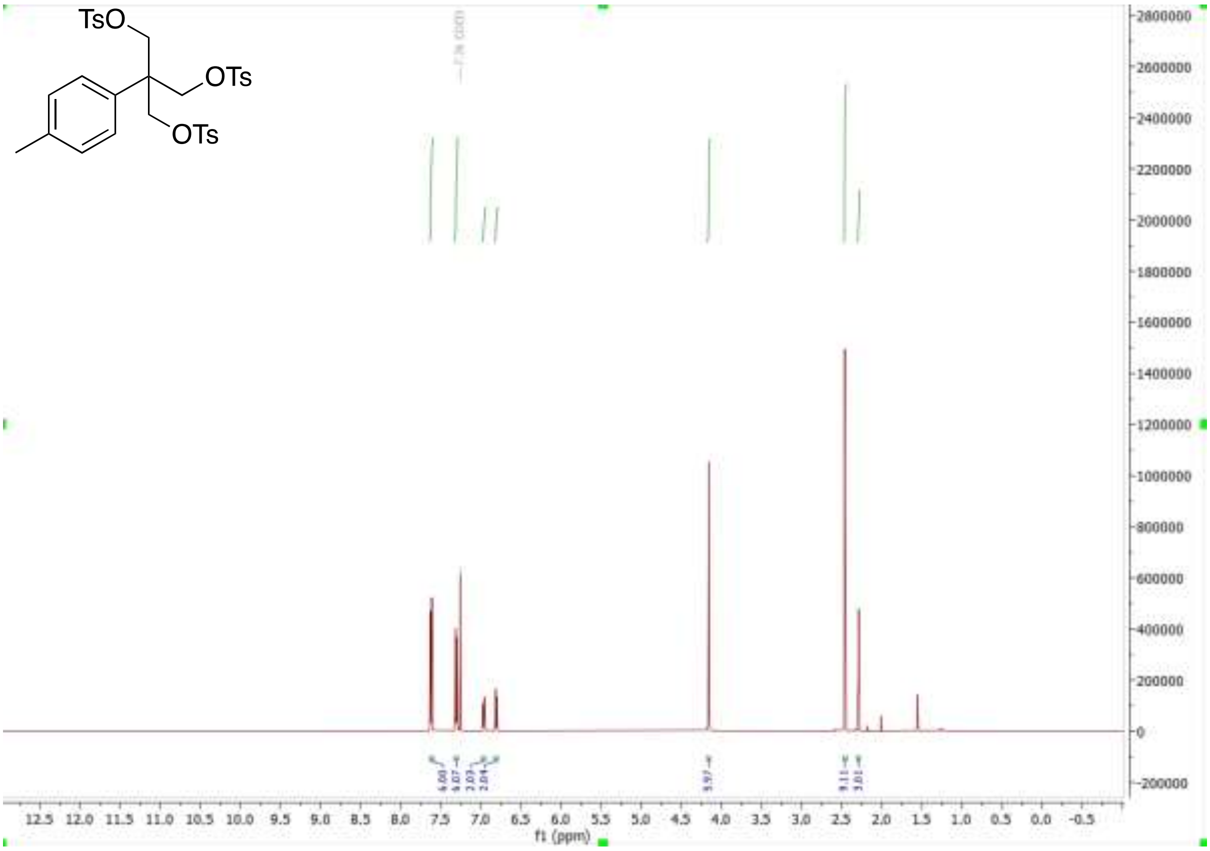

^{13}C NMR (126 MHz, $CDCl_3$):

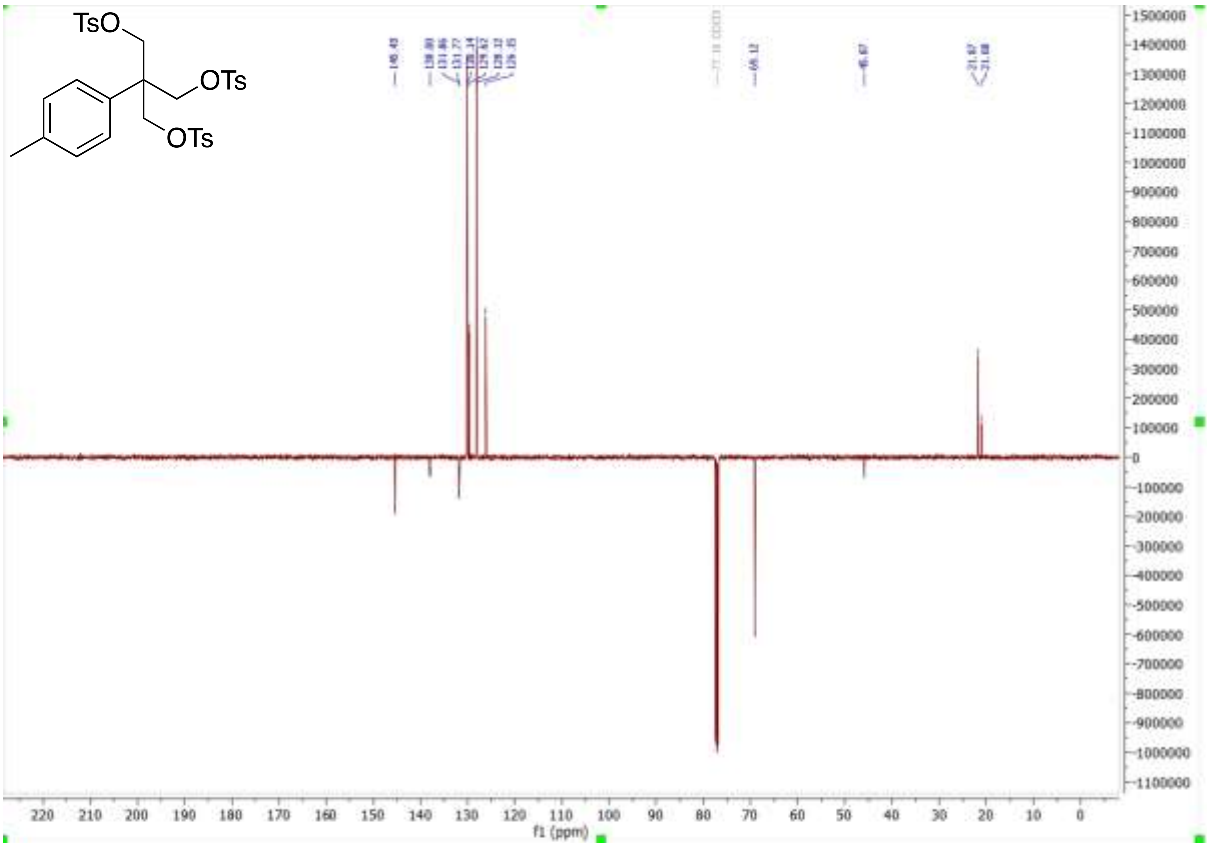

1-(1,3-Difluoro-2-(fluoromethyl)propan-2-yl)-4-methylbenzene (46)

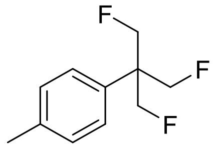

^{1}H NMR (500 MHz, $CDCl_3$):

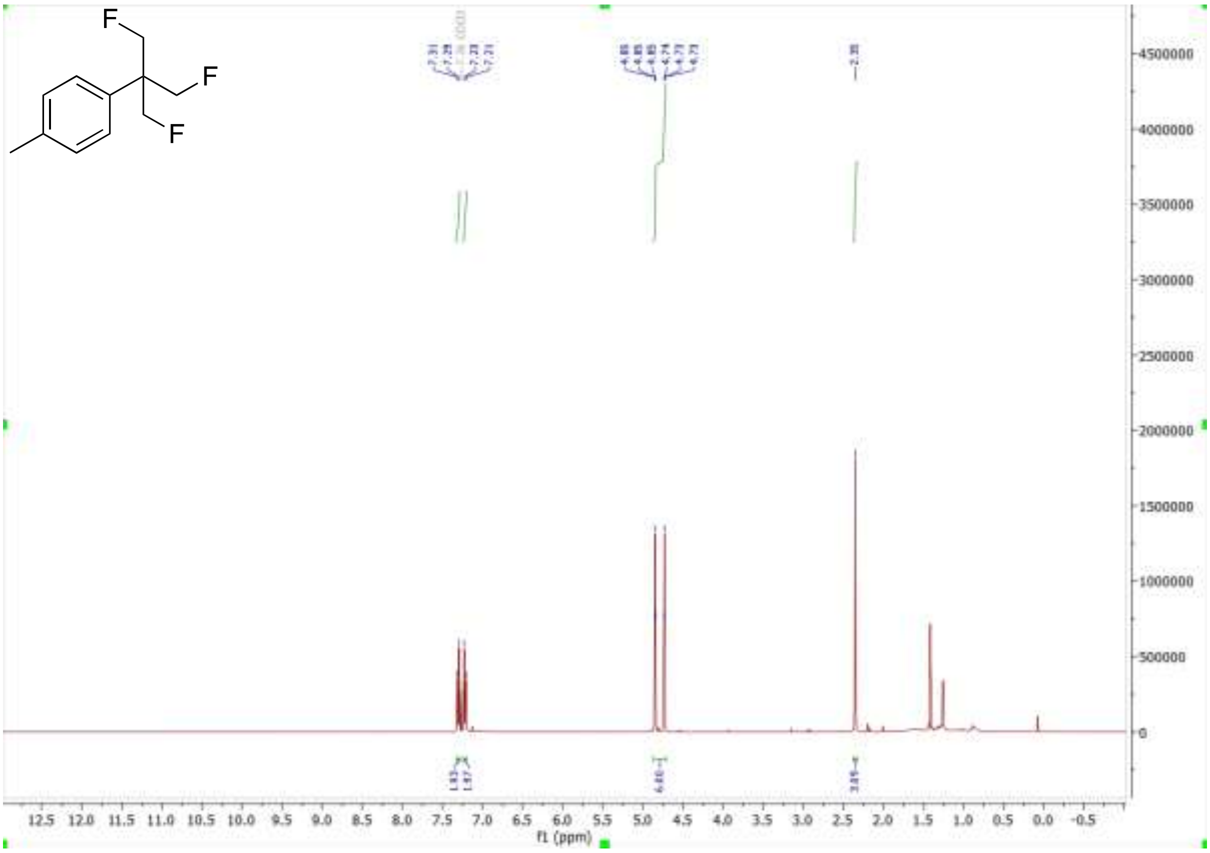

^{13}C NMR (126 MHz, $CDCl_3$):

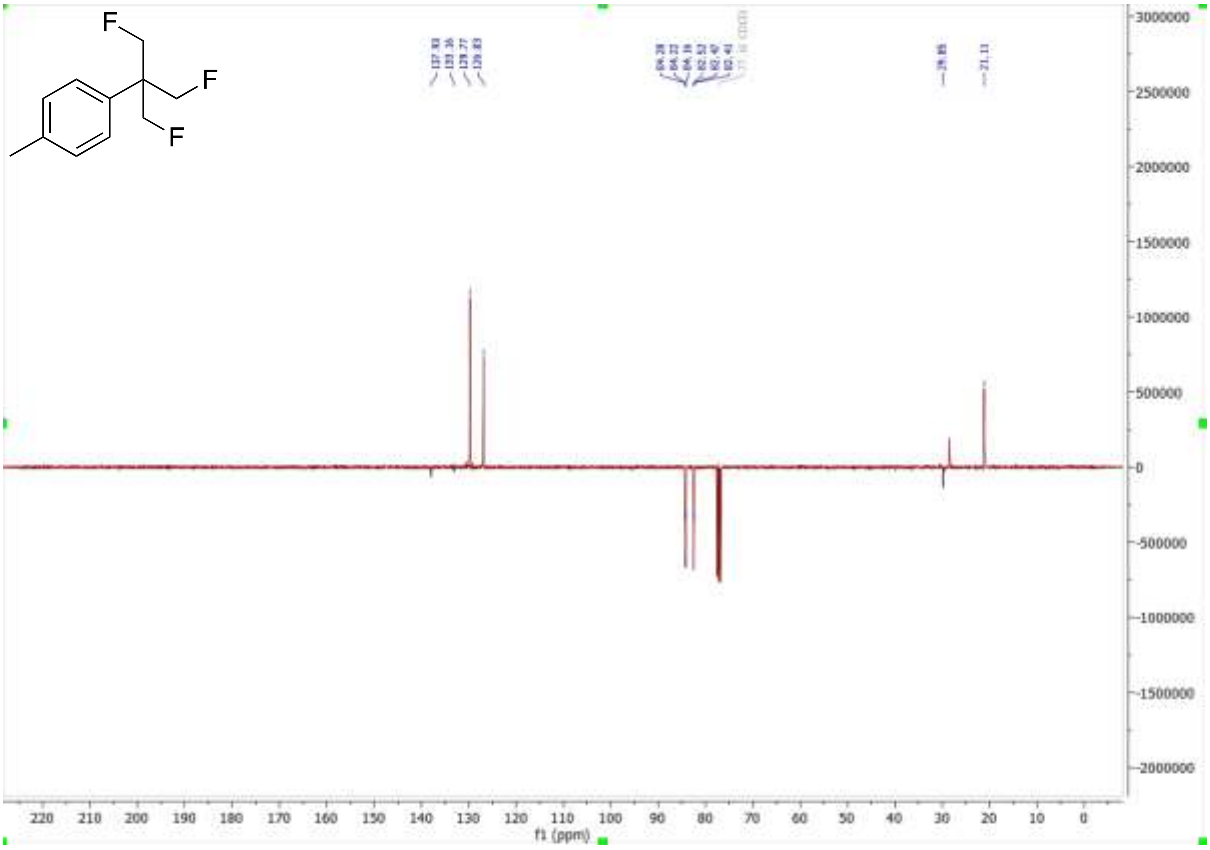

$^{19}F\{^1H\}$ NMR (376 MHz, $CDCl_3$):

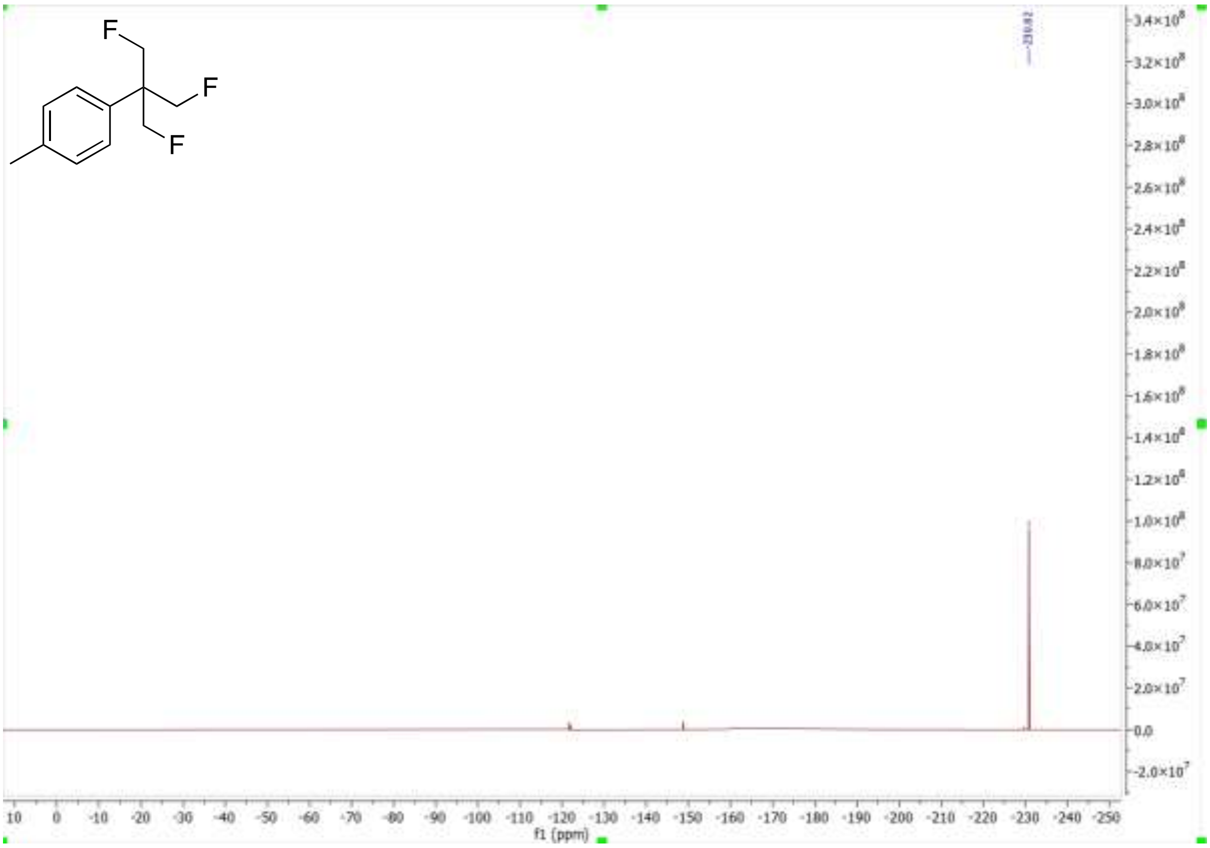

1-(Bromomethyl)-4-(1,3-difluoro-2-(fluoromethyl)propan-2-yl)benzene (47)

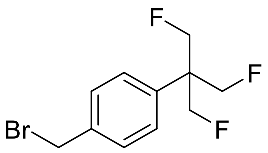

^{1}H NMR (400 MHz, $CDCl_3$):

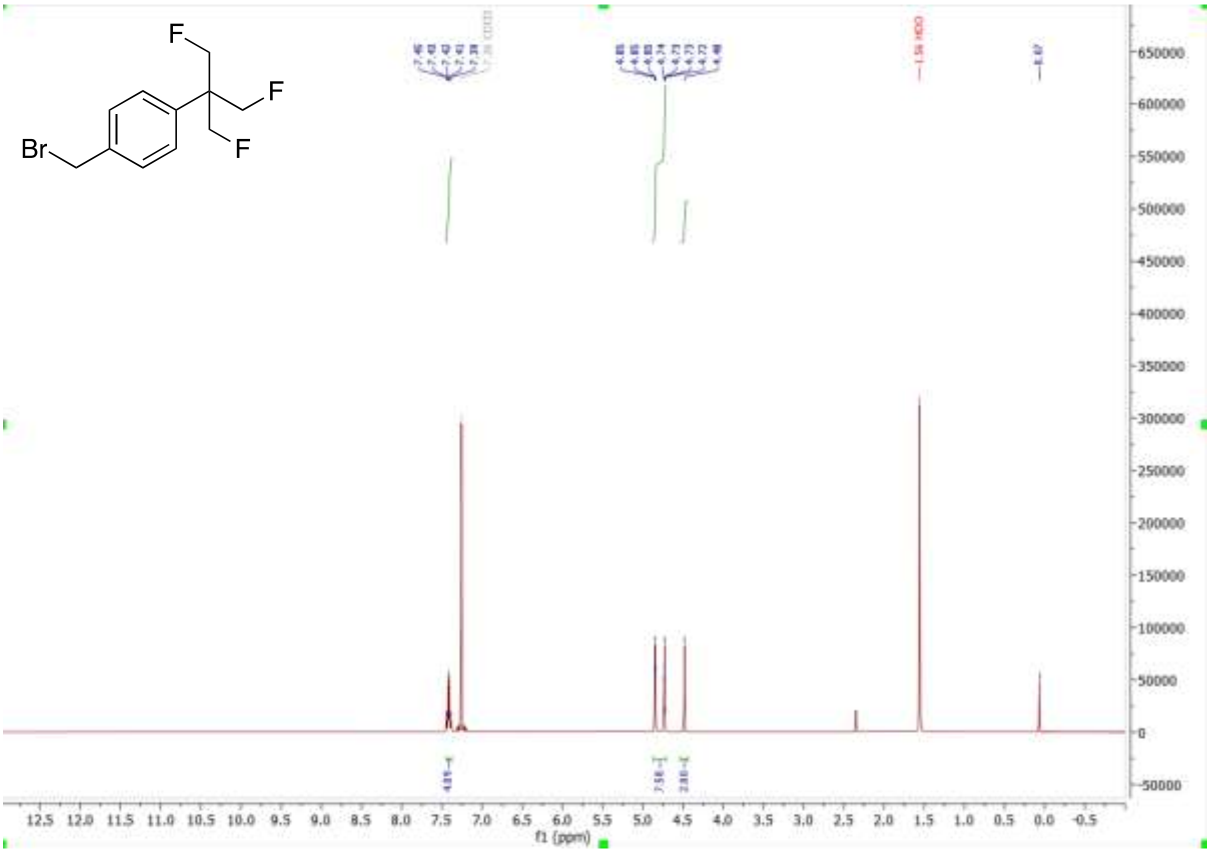

^{19}F NMR (470 MHz, $CDCl_3$):

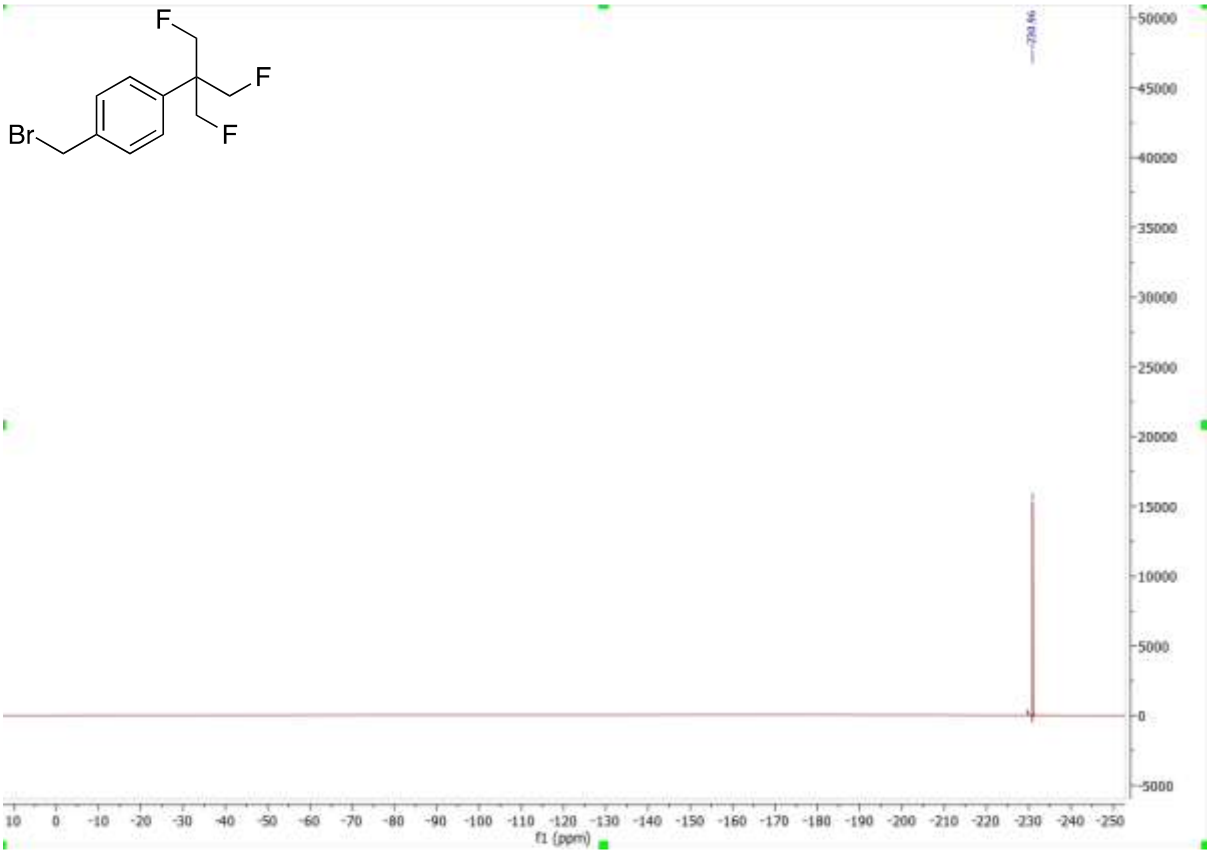

^{13}C NMR (126 MHz, $CDCl_3$):

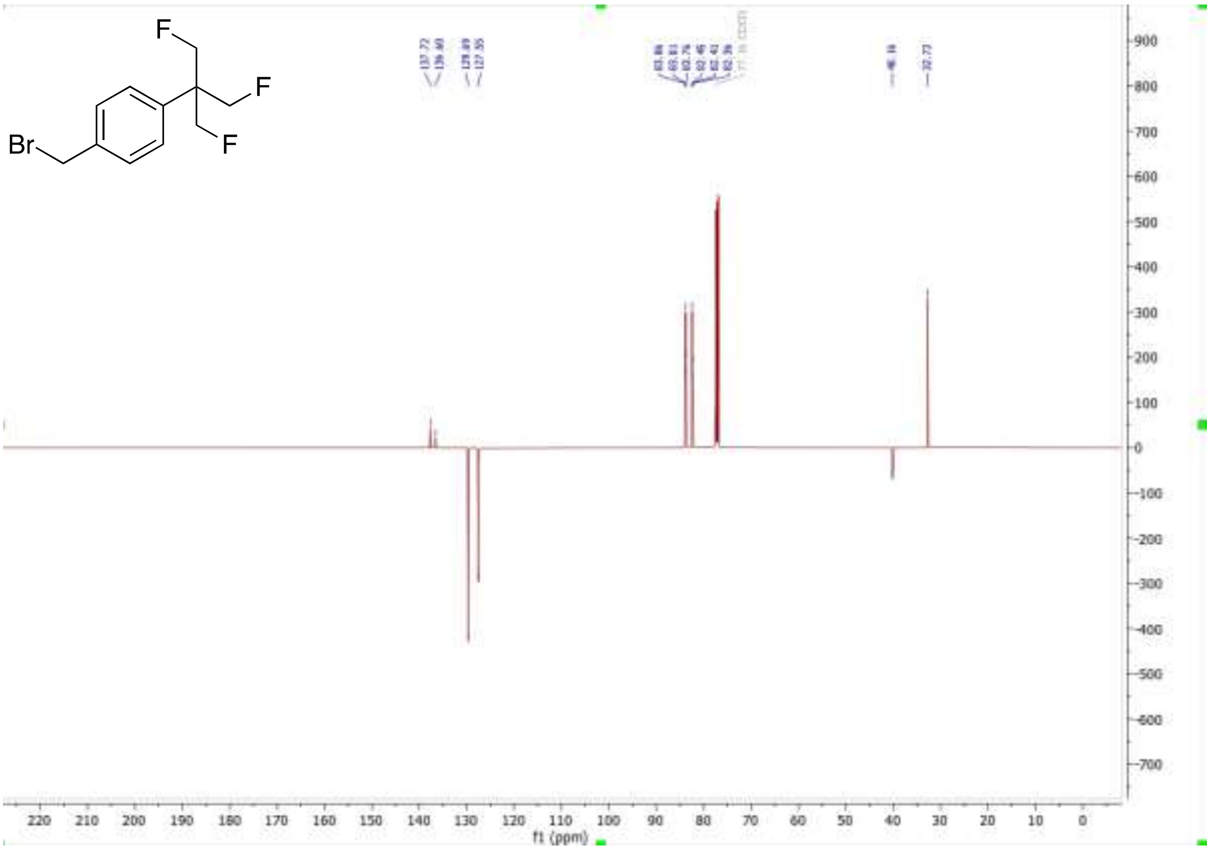

2-(Tert-butyl)-4,5-dichloropyridazin-3(2H)-one

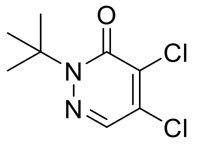

^{1}H NMR (400 MHz, $CDCl_3$):

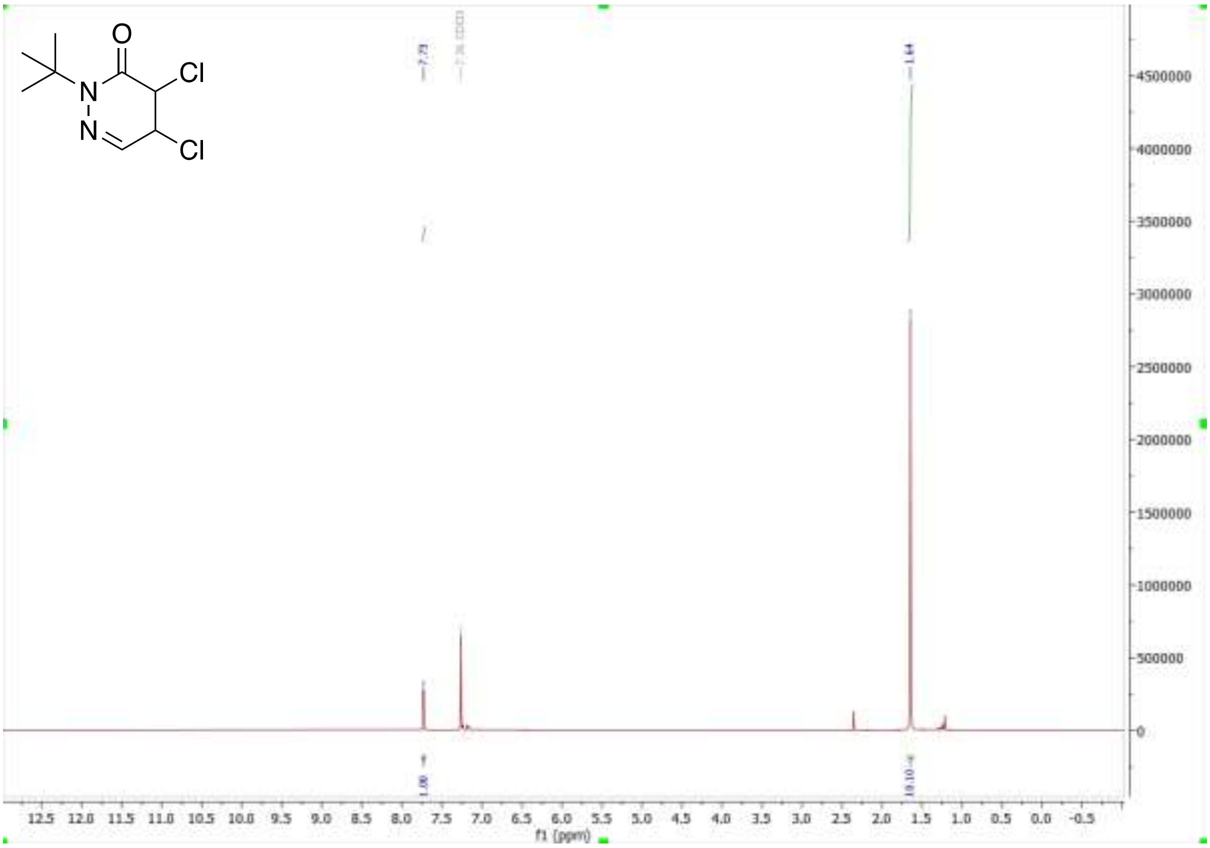

2-(Tert-butyl)-5-chloro-4-mercapto-4,5-dihydropyridazin-3(2H)-one (48)

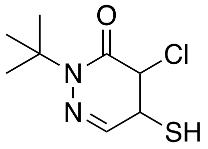

^{1}H NMR (400 MHz, $CDCl_3$):

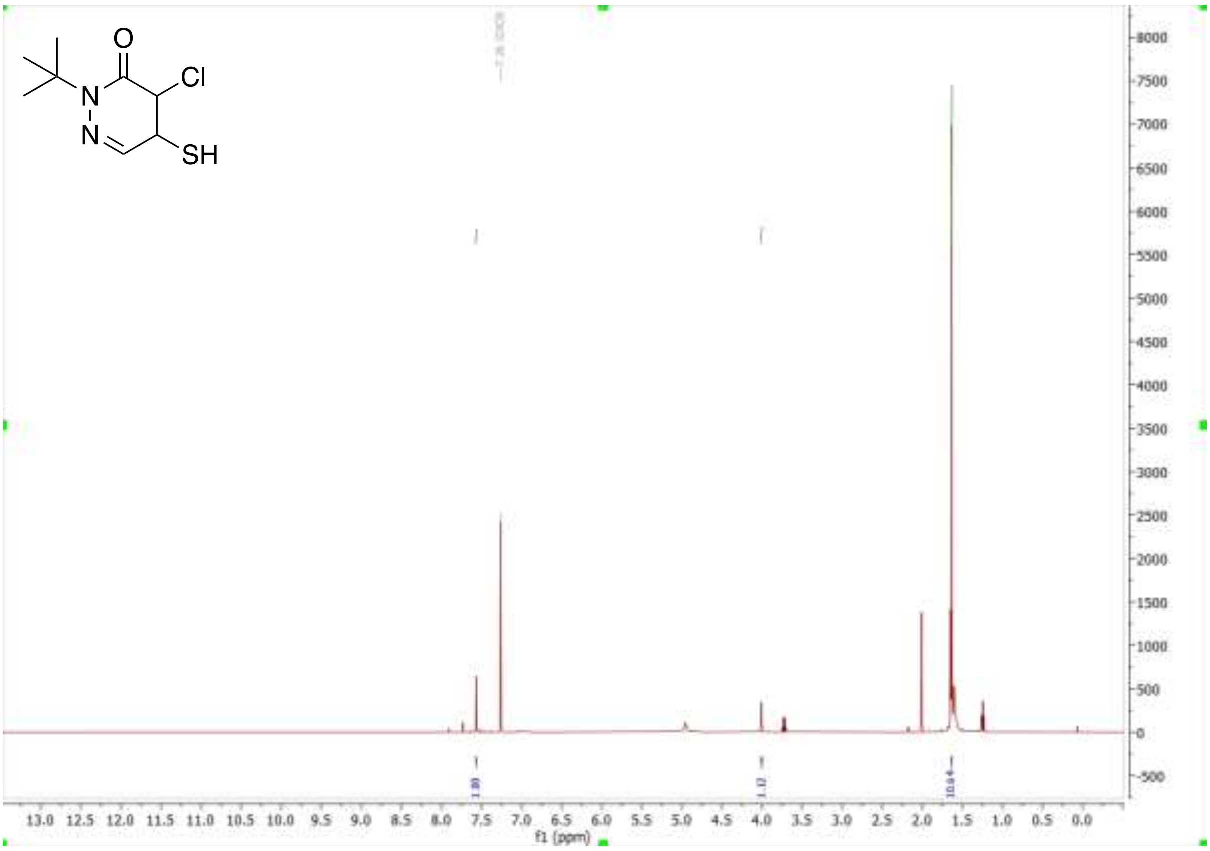

2-(Tert-butyl)-4-chloro-5-((4-(1,3-difluoro-2-(fluoromethyl)propan-2-yl)benzyl)thio)pyridazin-3(2H)-one (49)

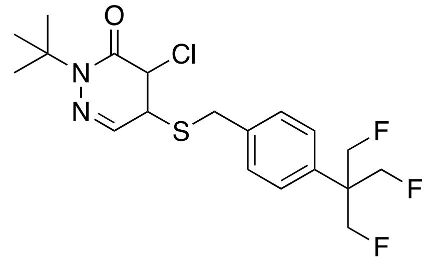

^{1}H NMR (400 MHz, $CDCl_3$):

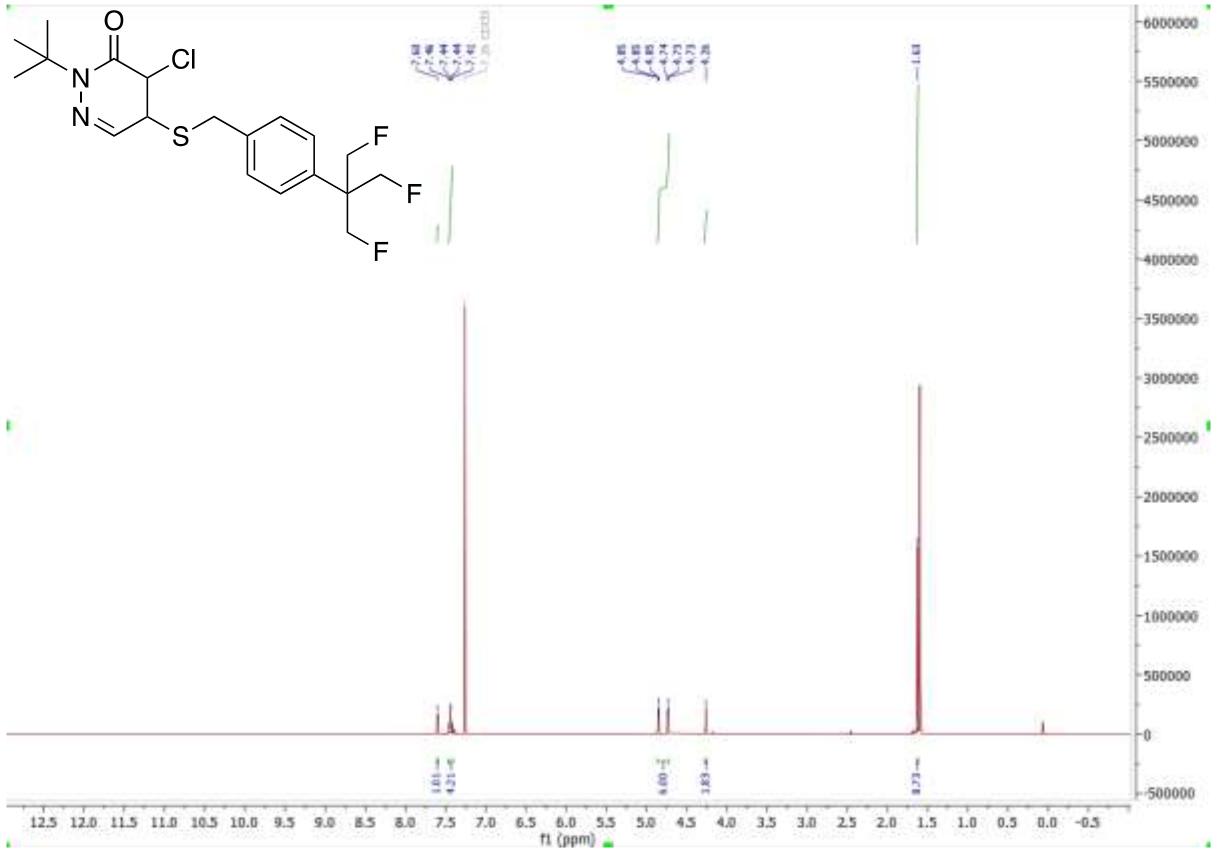

$^{19}F\{^{1}H\}$ NMR (377 MHz, $CDCl_3$):

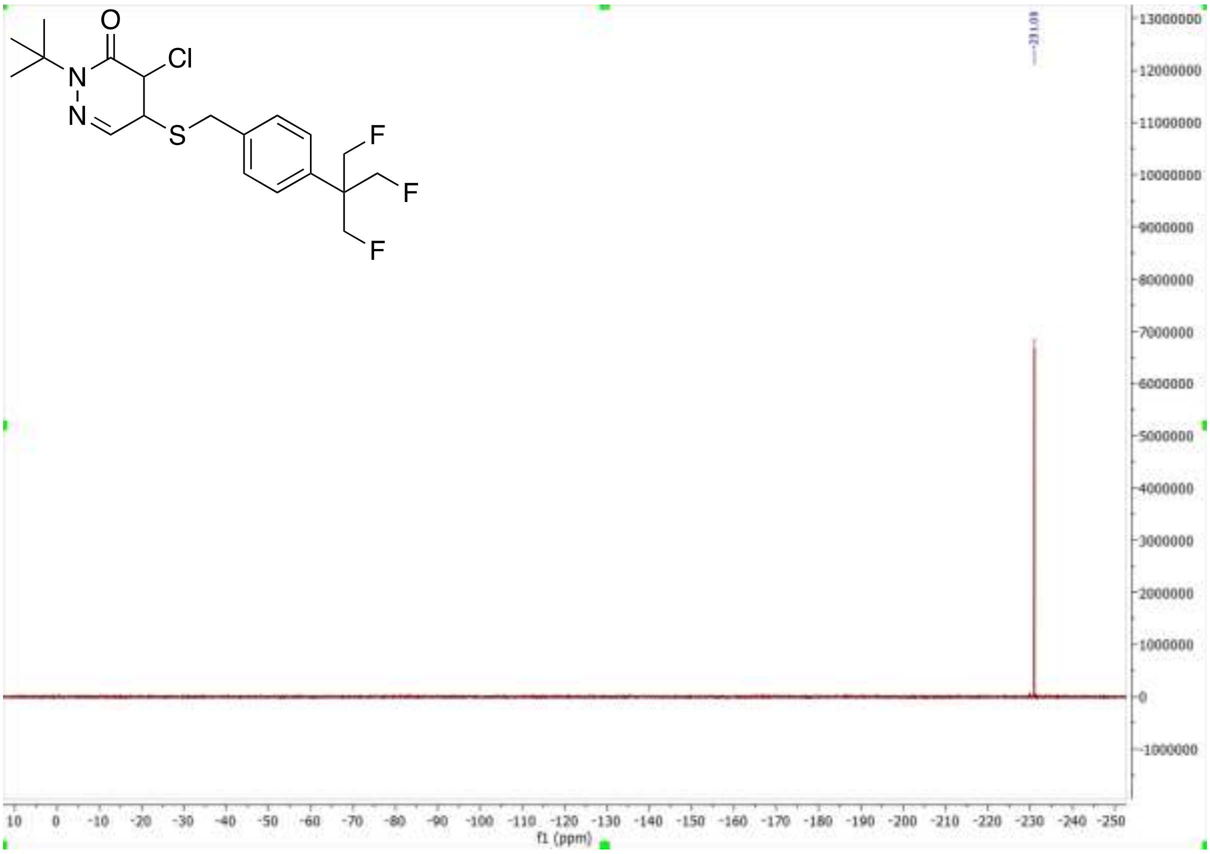

^{13}C NMR (101 MHz, $CDCl_3$):

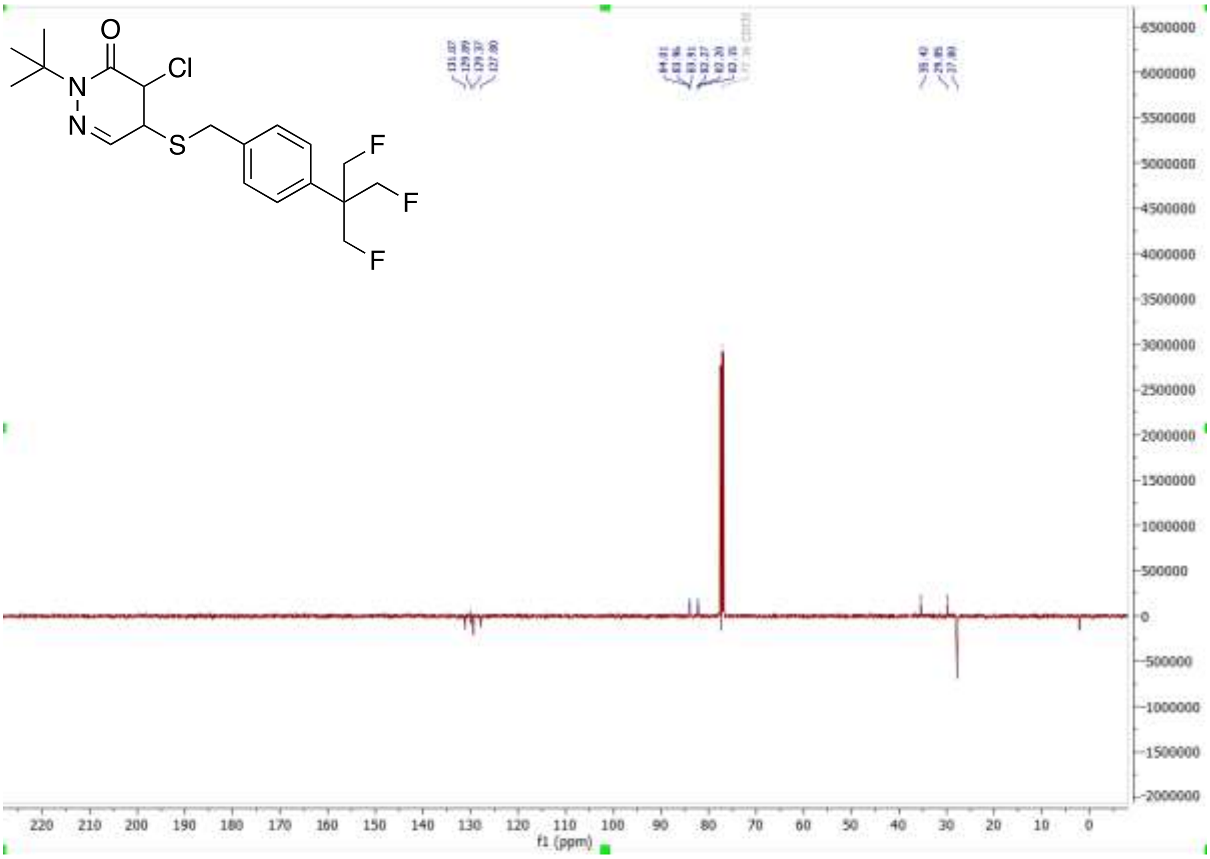

2-((Benzyloxy)methyl)-2-phenylpropane-1,3-diol

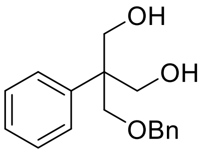

^{1}H NMR (400 MHz, d^{6}-Acetone):

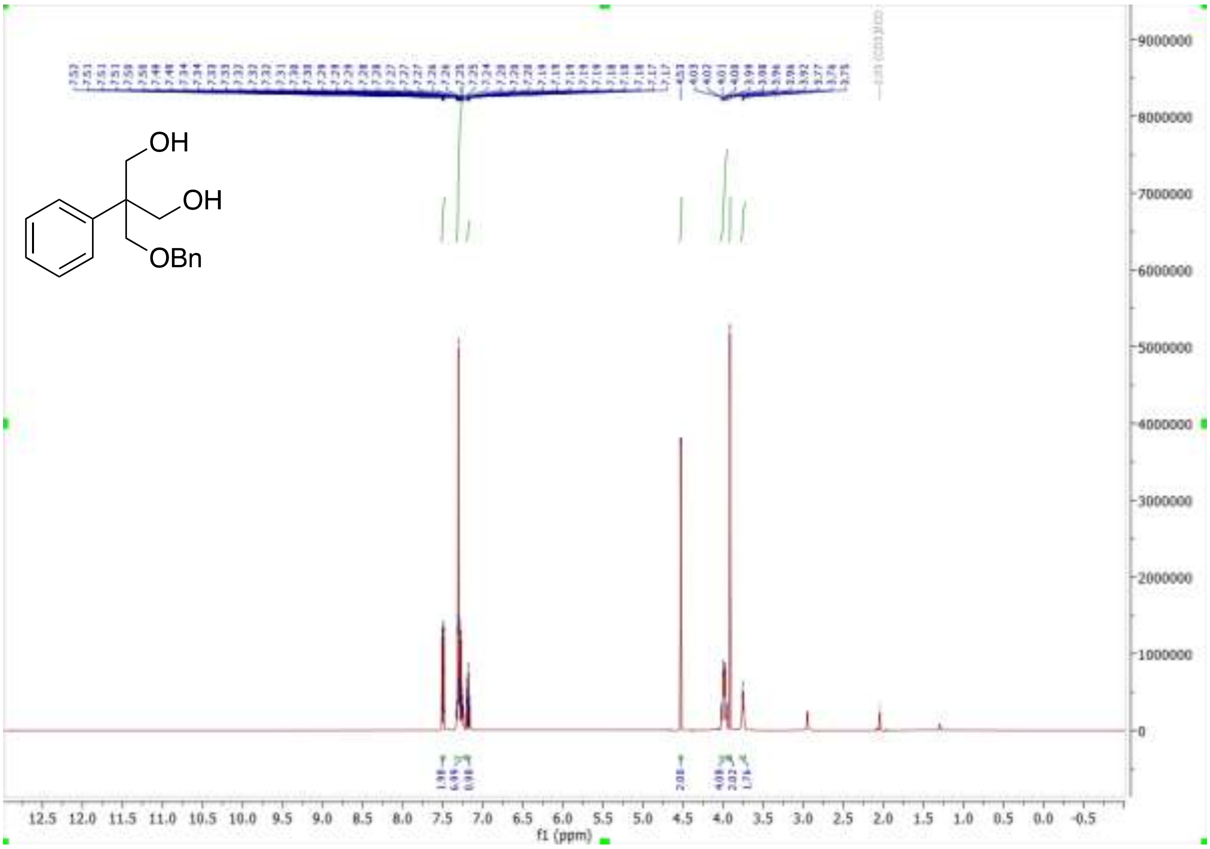

^{13}C NMR (101 MHz, d^{6}-Acetone):

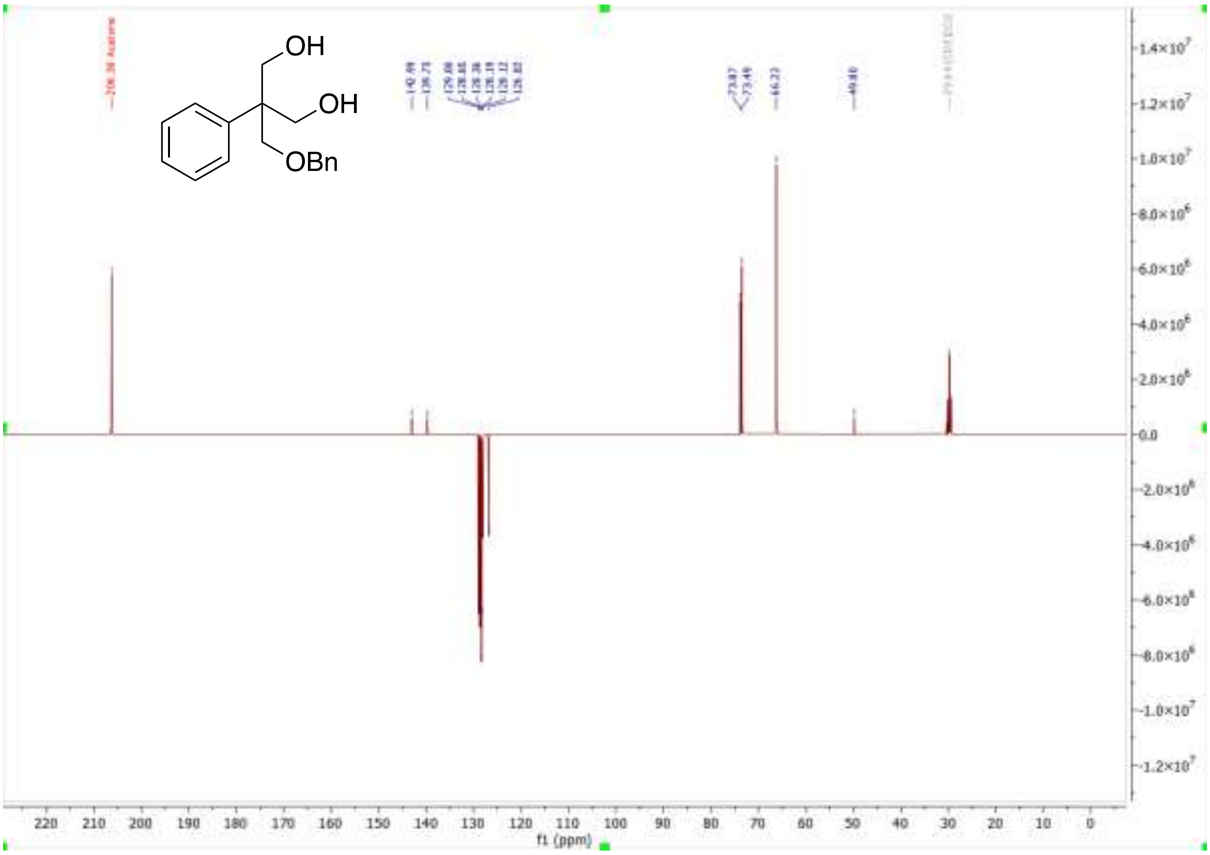

2-((Benzyloxy)methyl)-2-phenylpropane-1,3-diyl dimethanesulfonate

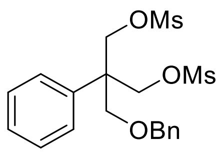

^{1}H NMR (400 MHz, $CDCl_3$):

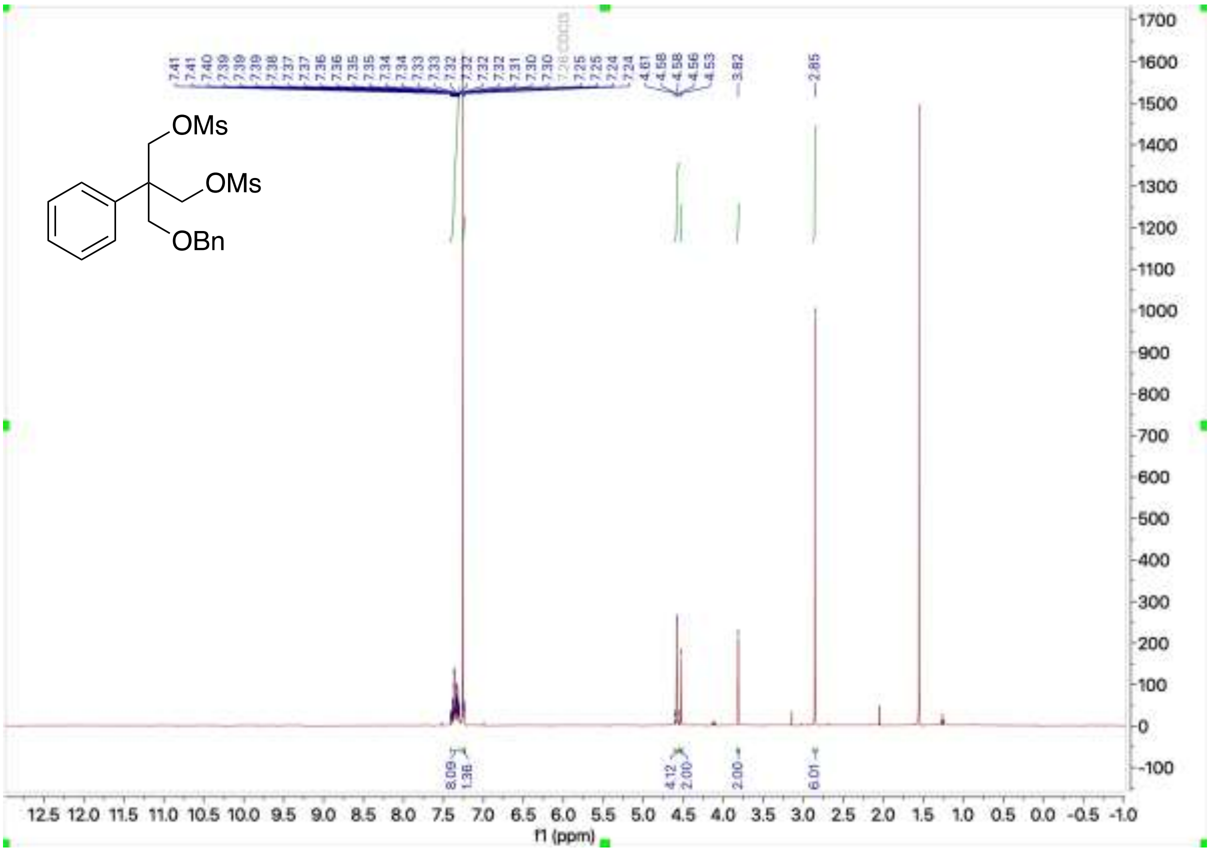

^{13}C NMR (126 MHz, $CDCl_3$):

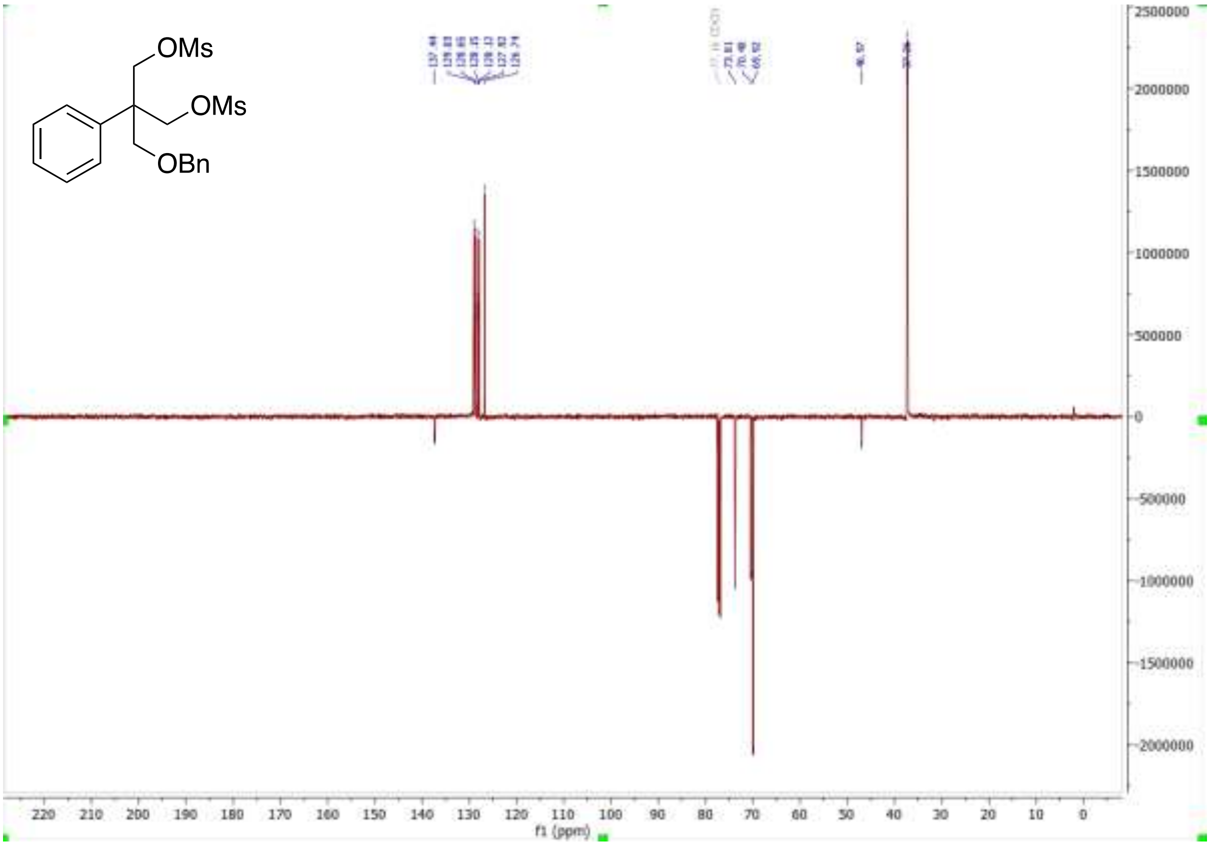

(1-(Benzyloxy)-3-fluoro-2-(fluoromethyl)propan-2-yl)benzene

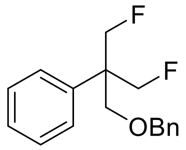

^{1}H NMR (400 MHz, $CDCl_3$):

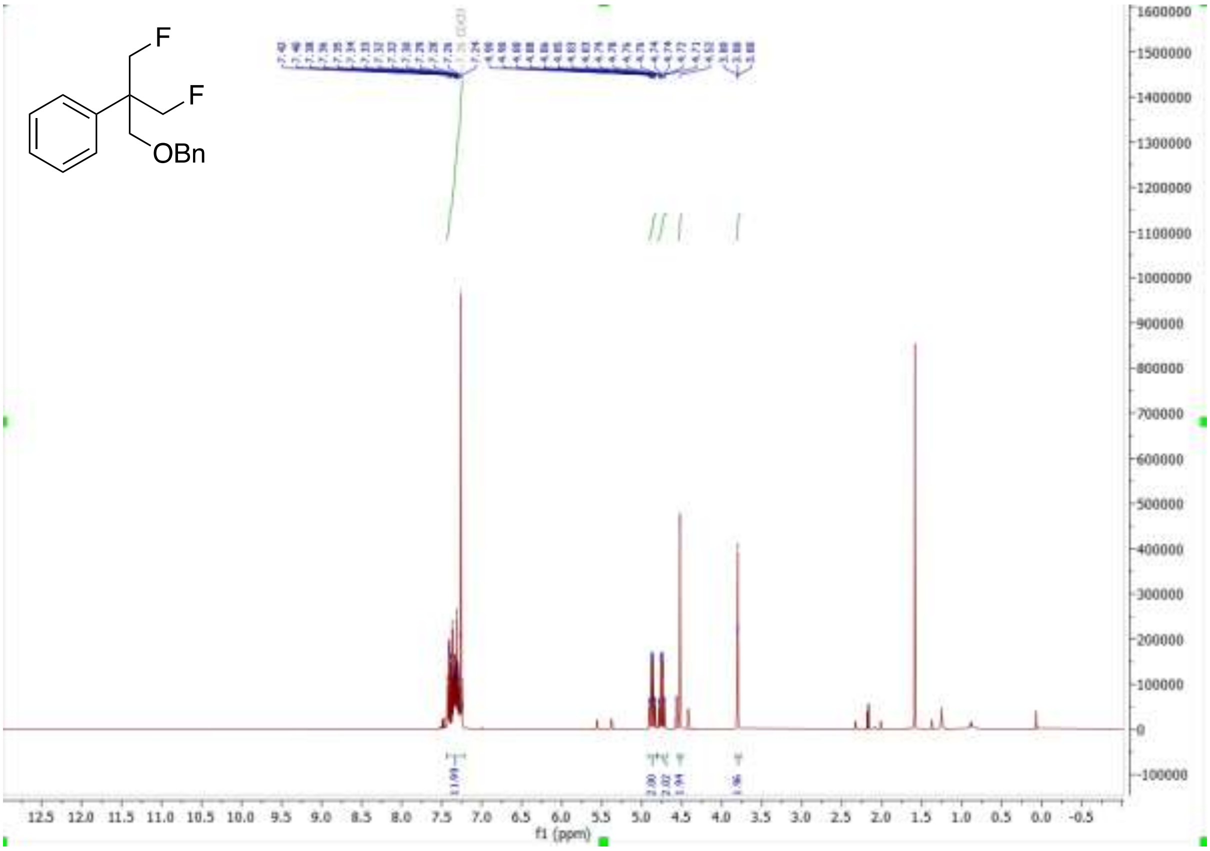

^{19}F NMR (470 MHz, $CDCl_3$):

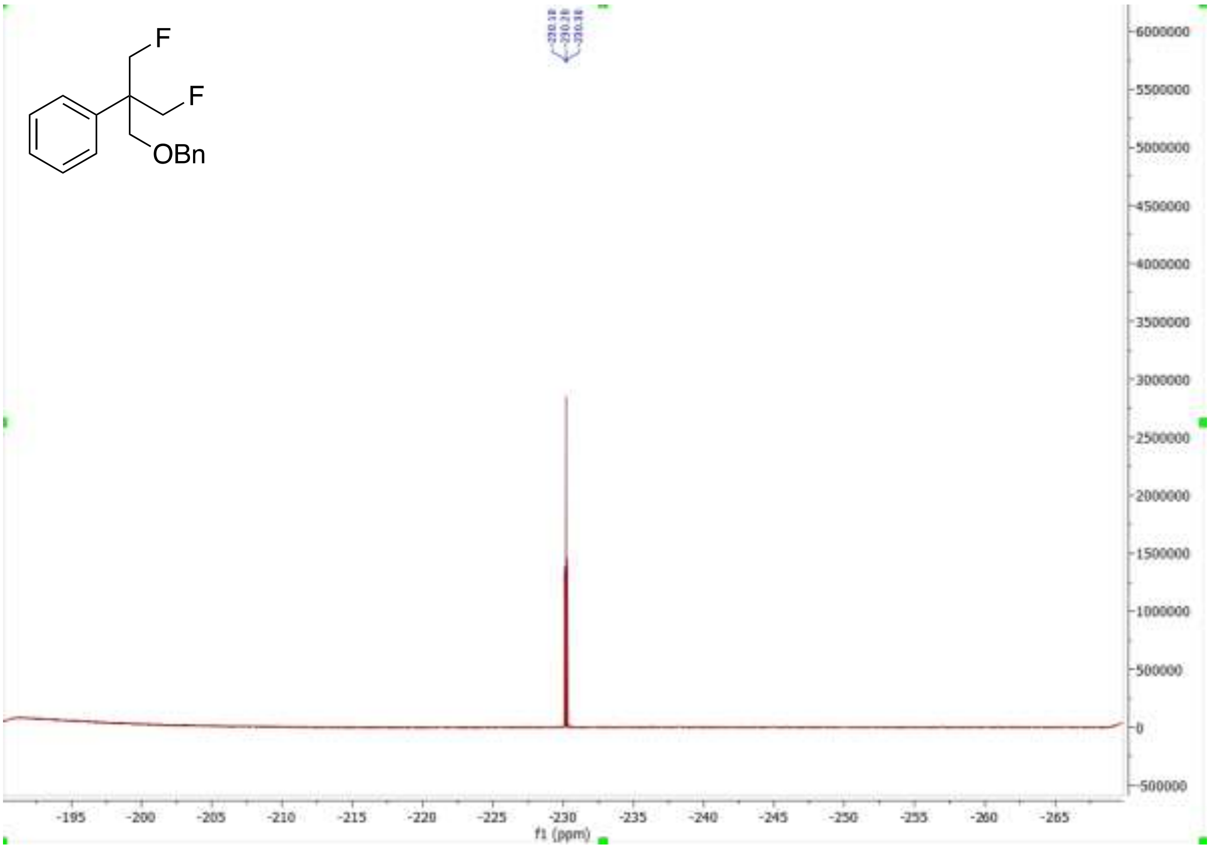

^{13}C NMR (126 MHz, $CDCl_3$):

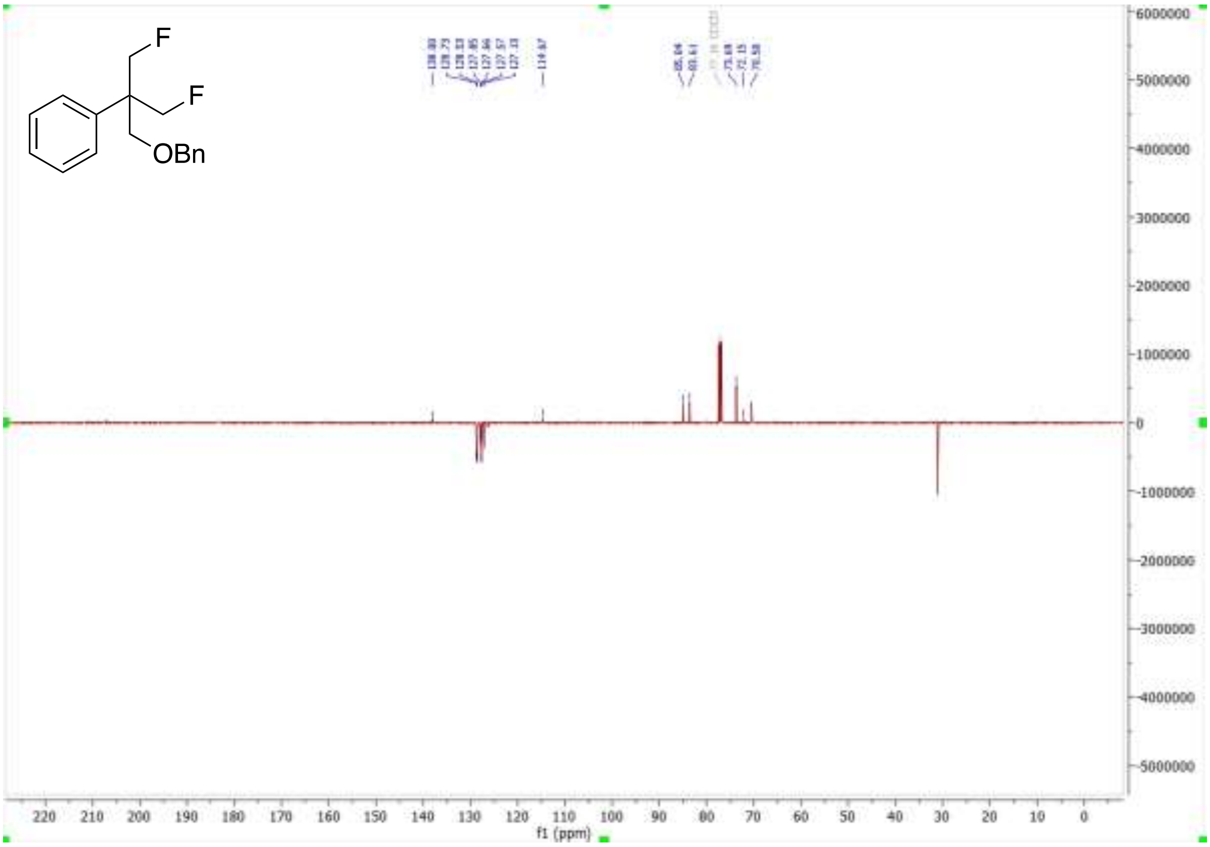

2-Methyl-2-phenylpropan-1-ol

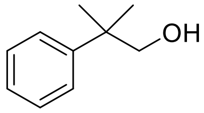

^{1}H NMR (500 MHz, $CDCl_3$):

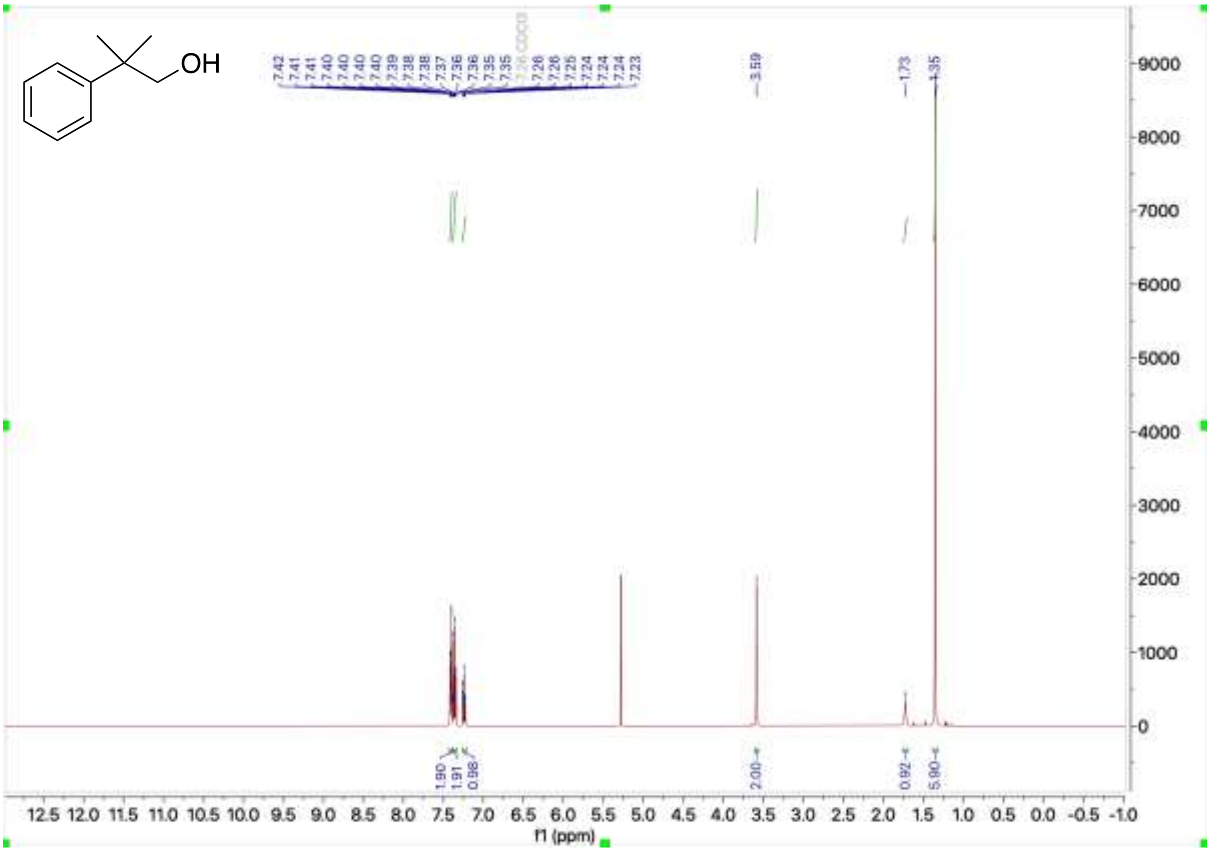

^{13}C NMR (126 MHz, $CDCl_3$):

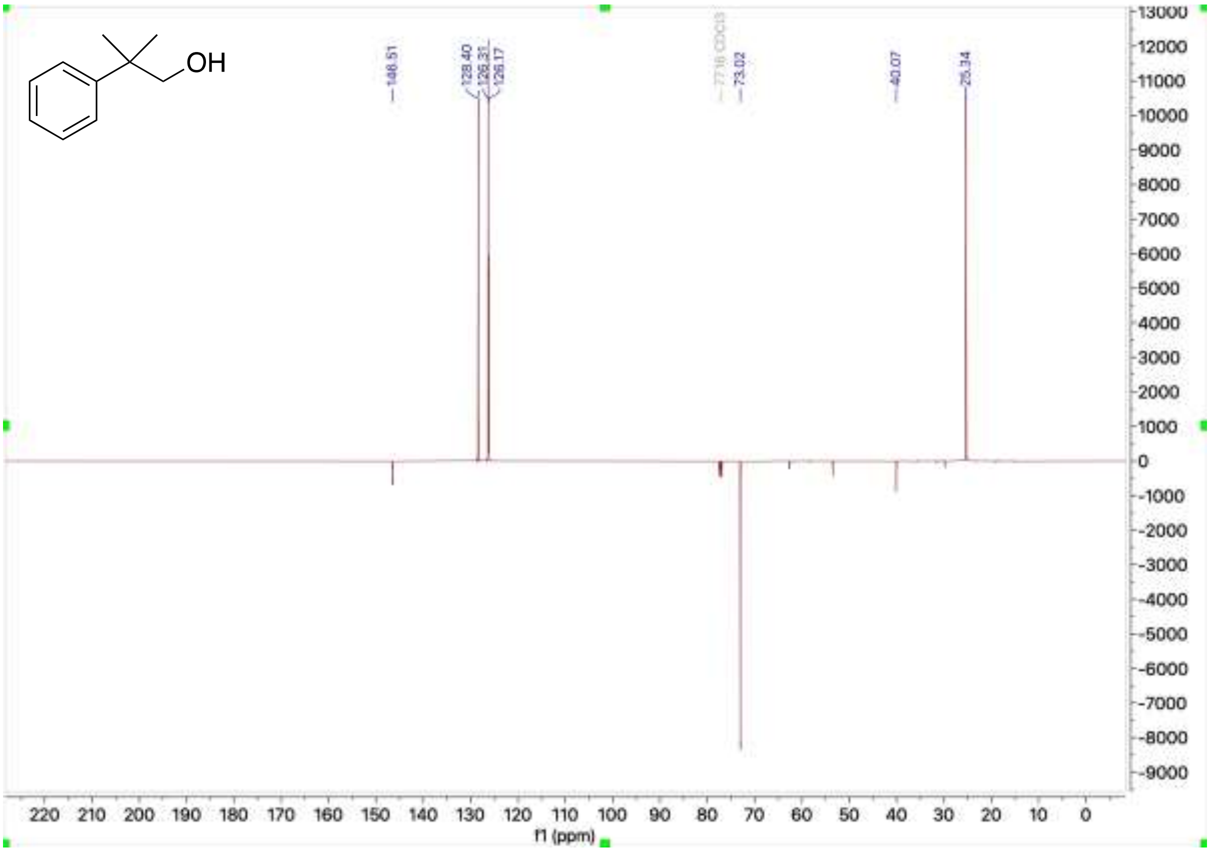

2-Methyl-2-phenylpropyl 4-methylbenzenesulfonate

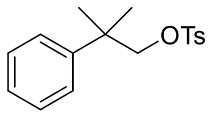

^1H NMR (500 MHz, $CDCl_3$):

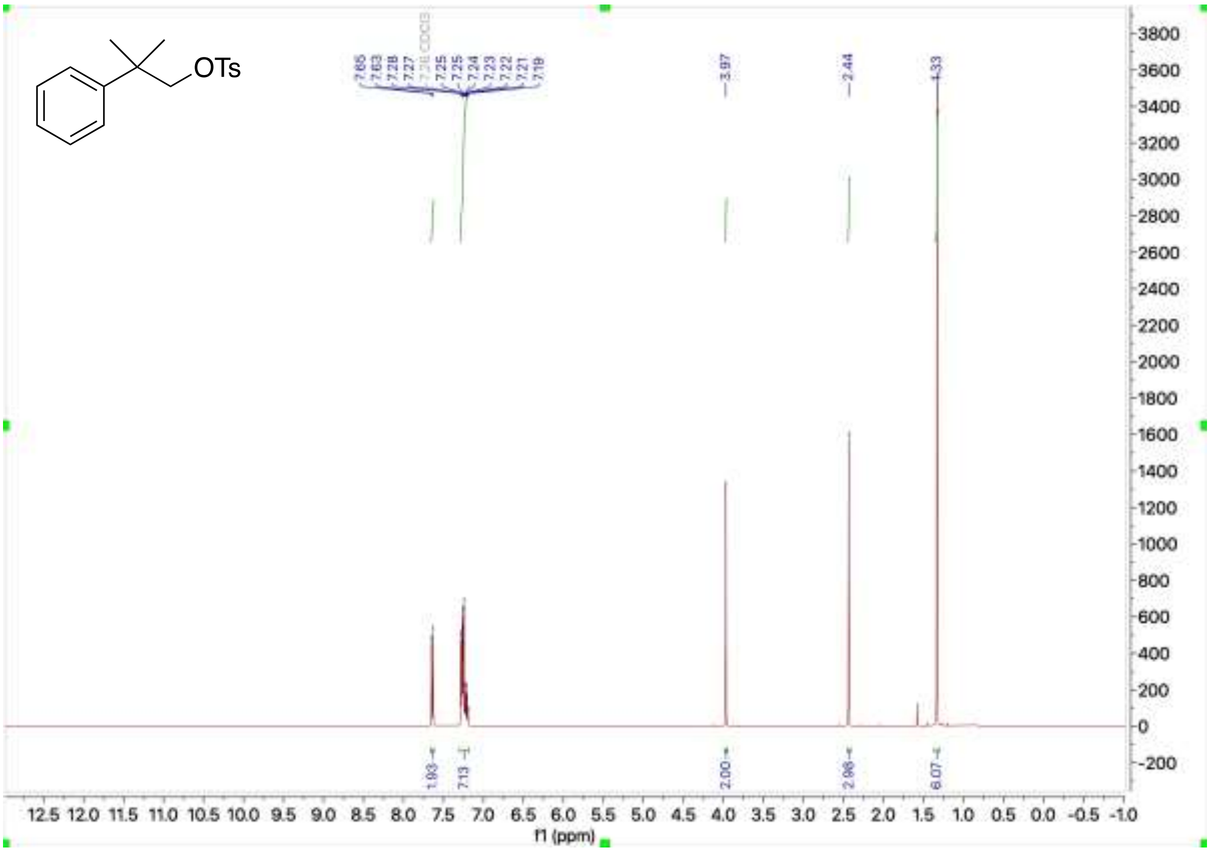

^{13}C NMR (126 MHz, $CDCl_3$):

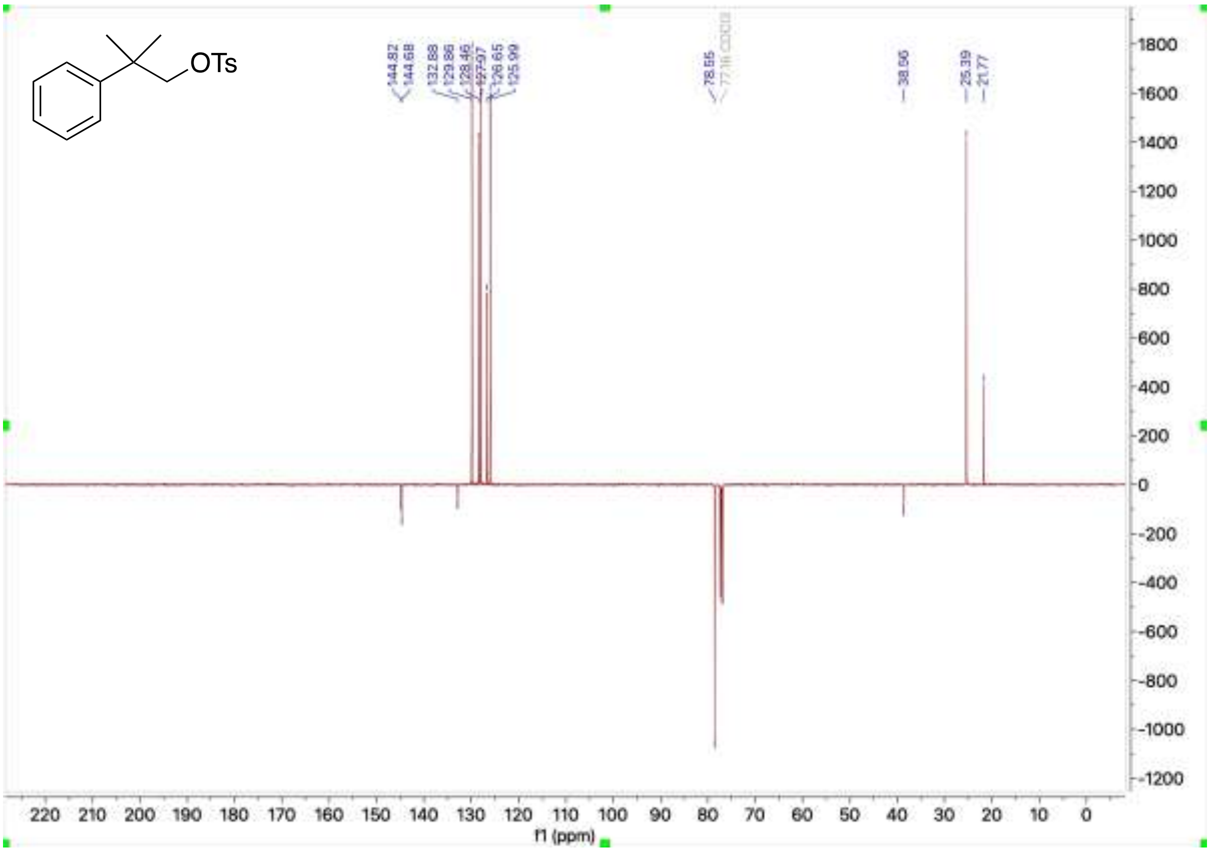

(1-Fluoro-2-methylpropan-2-yl)benzene (37)

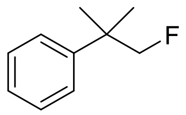

^{1}H NMR (500 MHz, $CDCl_3$):

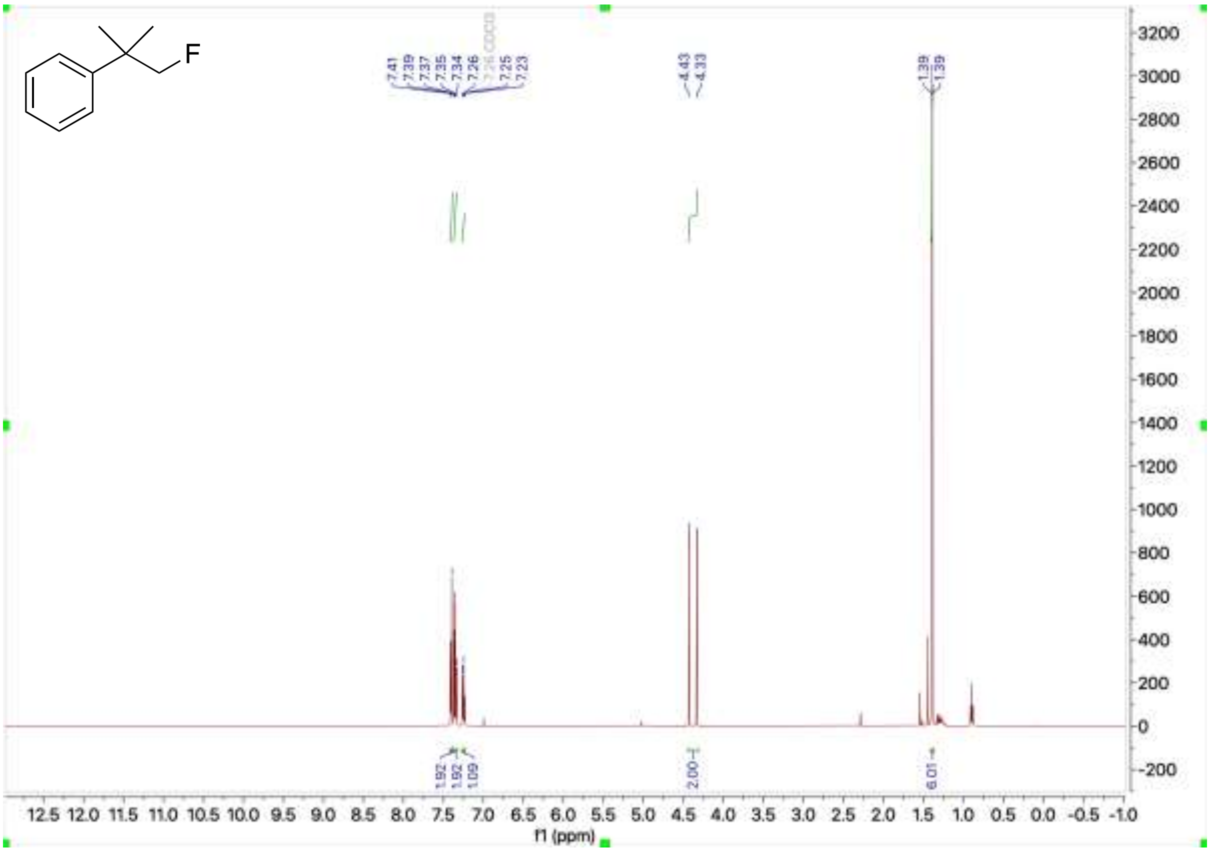

^{13}C NMR (126 MHz, $CDCl_3$):

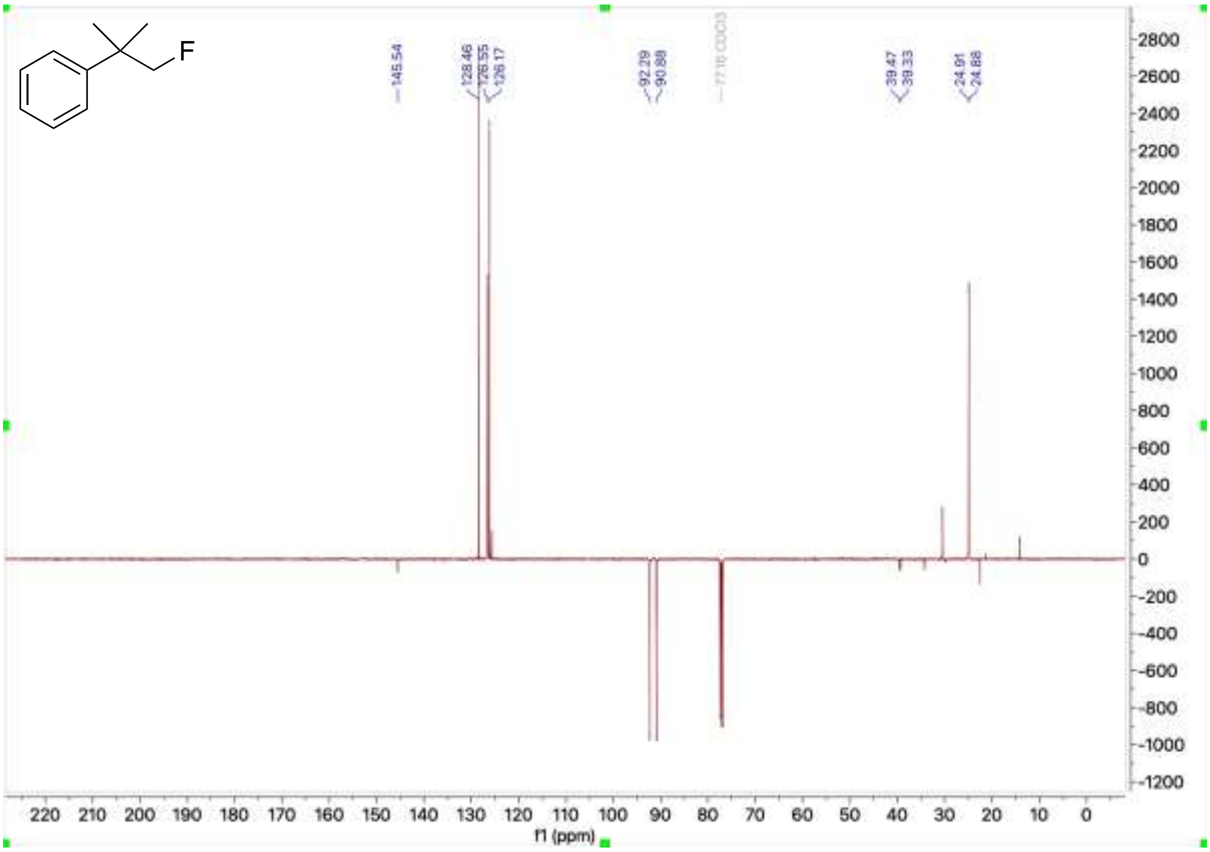

^{19}F NMR (470 MHz, $CDCl_3$):

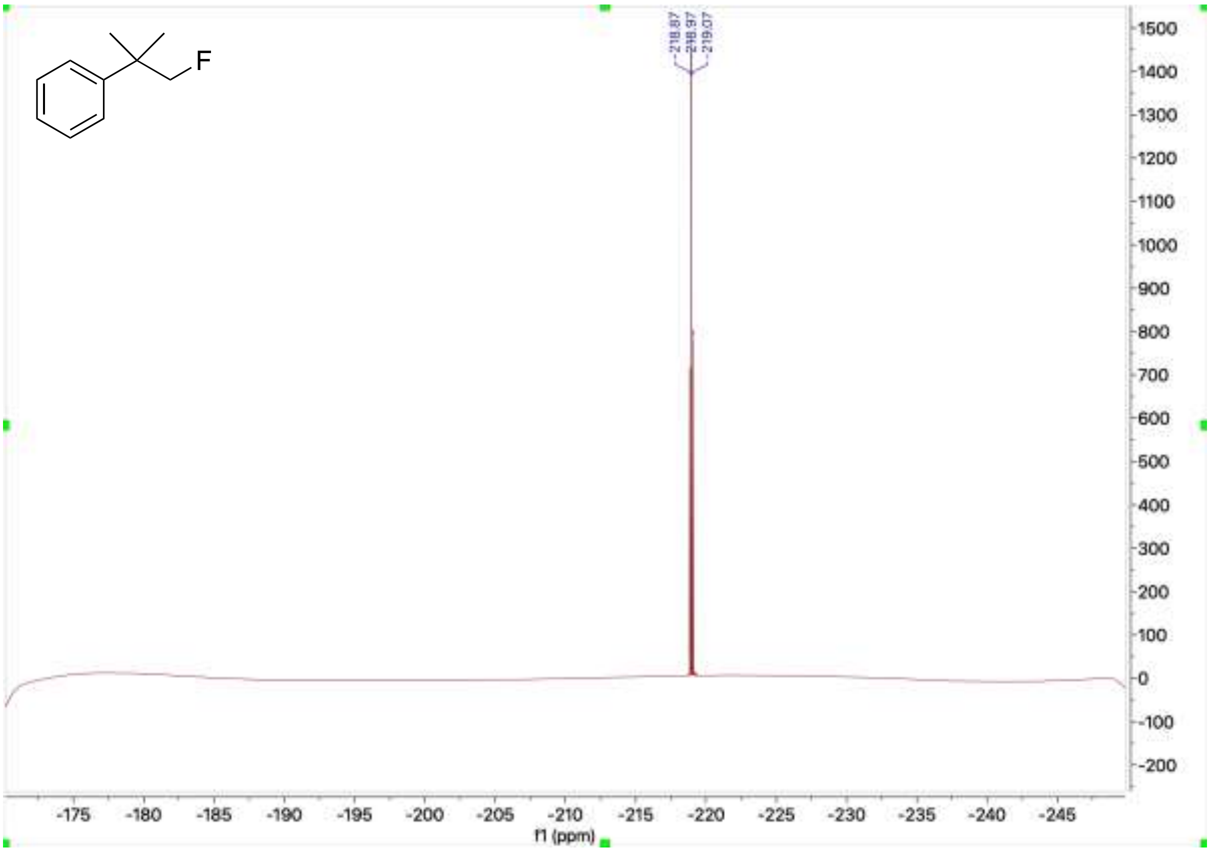

2-Methyl-2-phenylpropane-1,3-diyl bis(4-methylbenzenesulfonate)

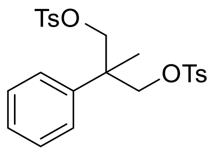

^{1}H NMR (500 MHz, $CDCl_3$):

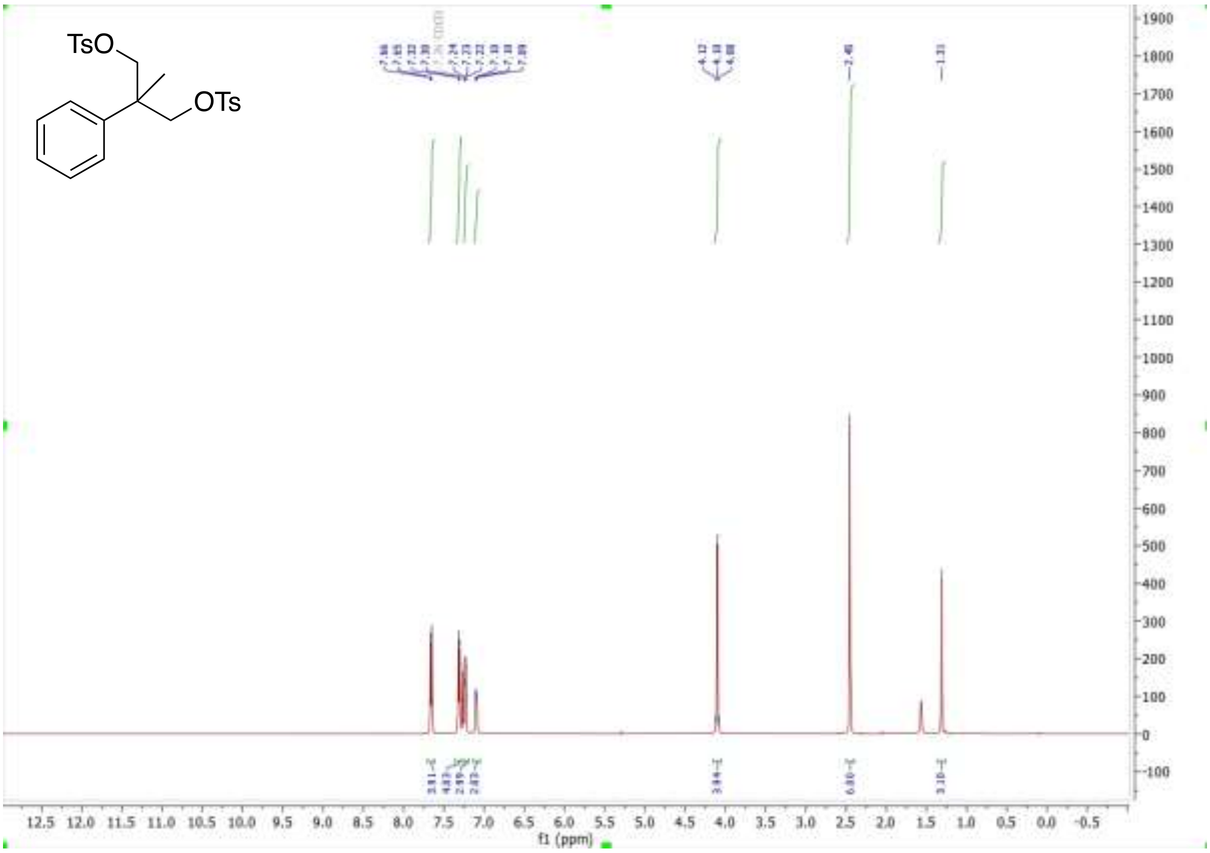

(1,3-Difluoro-2-methylpropan-2-yl)benzene (36)

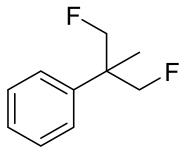

^{1}H NMR (500 MHz, $CDCl_3$):

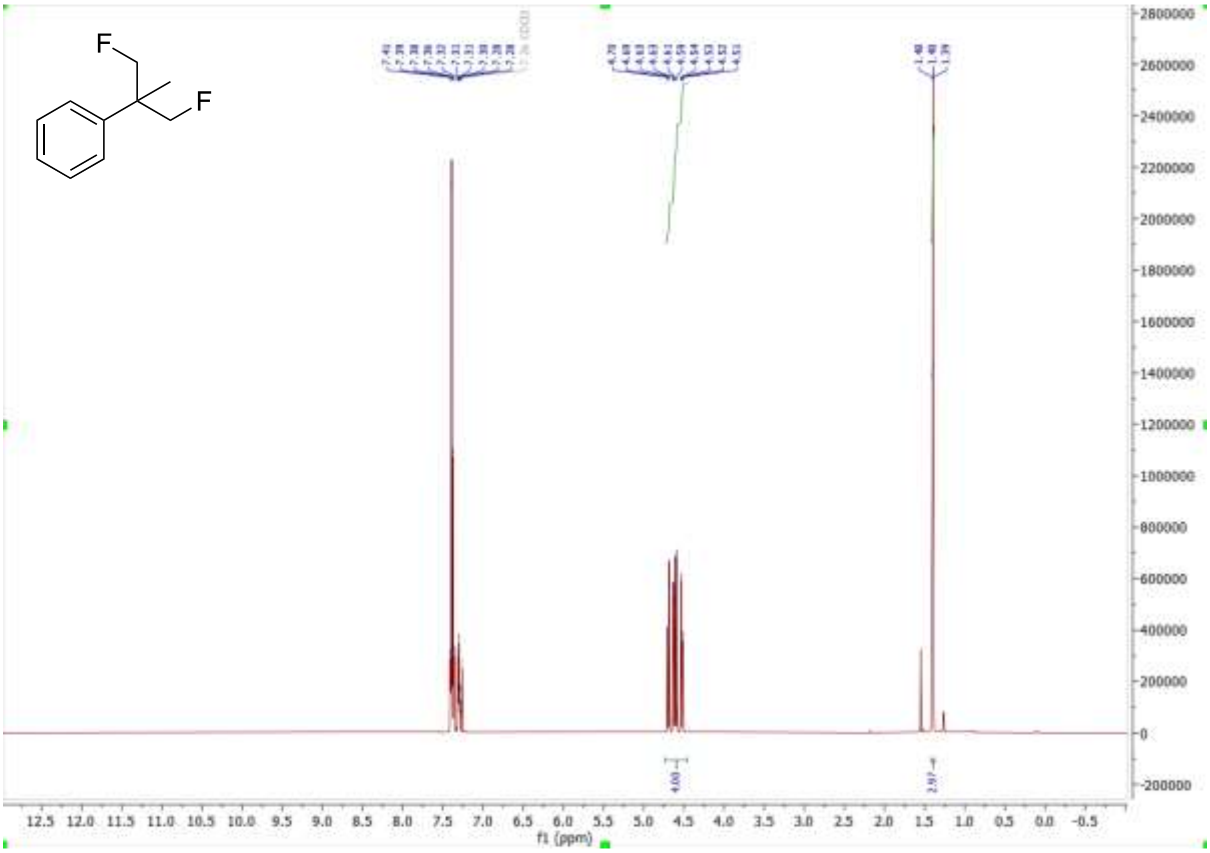

^{19}F NMR (470 MHz, $CDCl_3$):

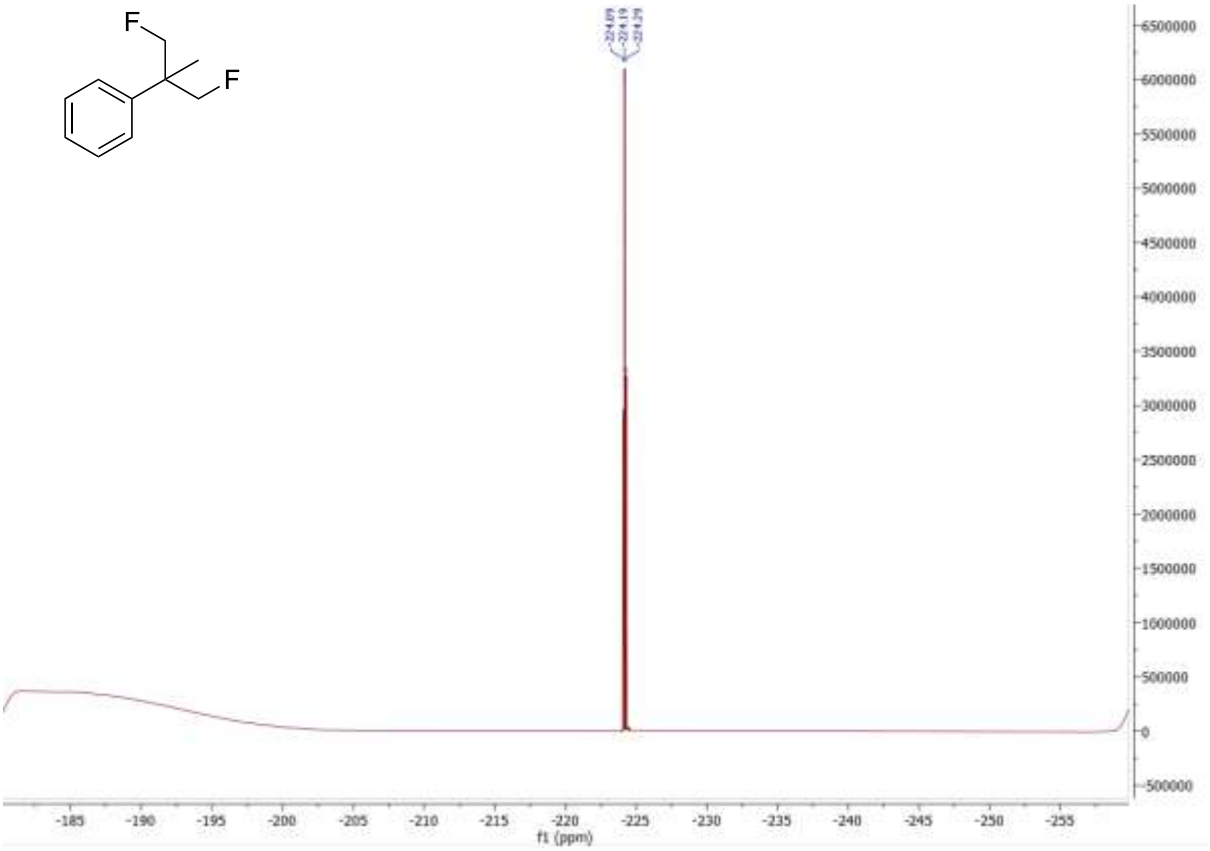

^{13}C NMR (126 MHz, $CDCl_3$):

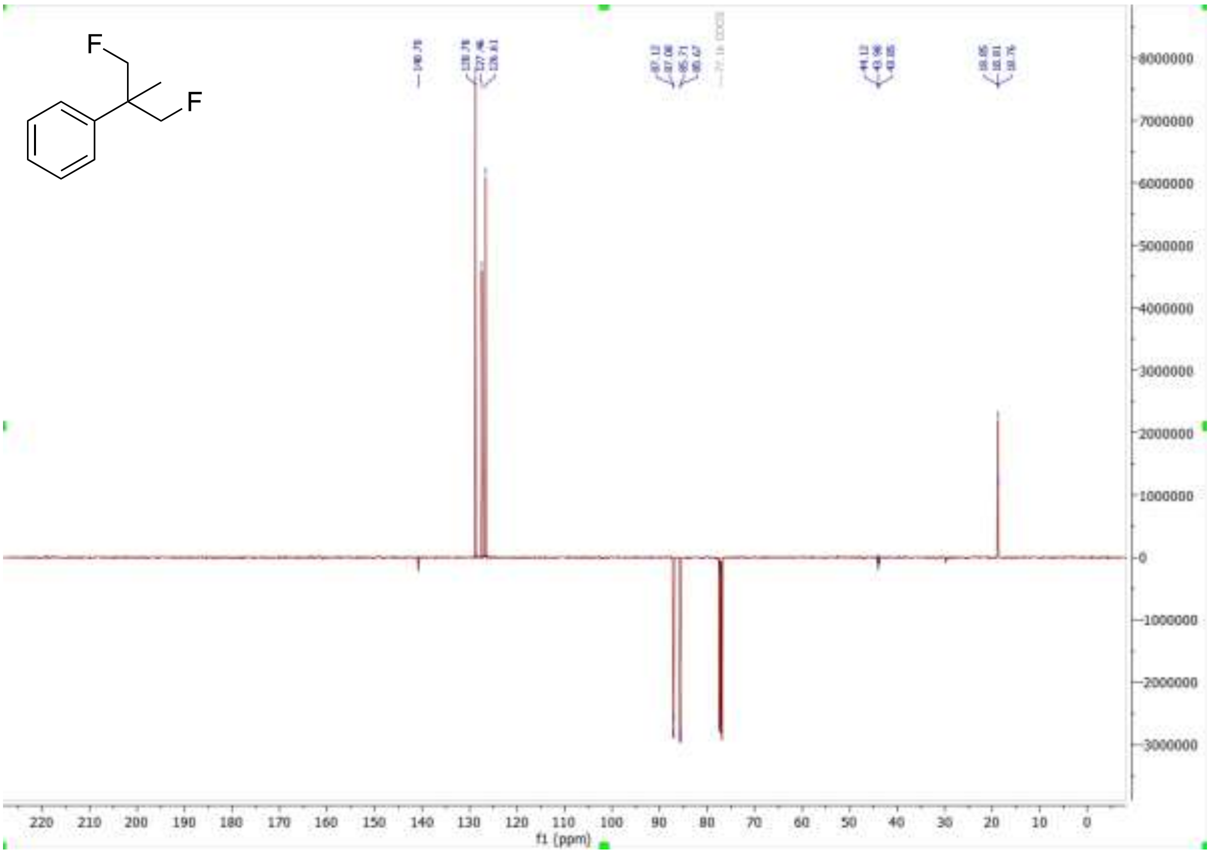

[END]
